# α-Hydroxylactams as Efficient Entries to Diversely Functionalized Ferrociphenols: Synthesis and Antiproliferative Activity Studies

**DOI:** 10.3390/molecules27144549

**Published:** 2022-07-16

**Authors:** Pascal Pigeon, Marie Gaschard, Mohamed Othman, Michèle Salmain, Gérard Jaouen

**Affiliations:** 1 Chimie ParisTech, PSL, 11 rue Pierre et Marie Curie, F-75005 Paris, France; gerard.jaouen@chimieparistech.psl.eu; 2 Sorbonne Université, CNRS, Institut Parisien de Chimie Moléculaire (IPCM), 4 Place Jussieu, F-75005 Paris, France; marie.gaschard_stefanelli@sorbonne-universite.fr (M.G.); michele.salmain@sorbonne-universite.fr (M.S.); 3 Normandie University, URCOM, UNIHAVRE, FR3021, EA 3221, 25 rue P. Lebon BP 540, F-76058 Le Havre, France; mohamed.othman@univ-lehavre.fr

**Keywords:** ferrocifen, hydroxylactam, *N*-acyliminium ion, anticancer activity, ferrocene

## Abstract

The [ferrocene-ene-phenol] motif has been identified as the pharmacophore responsible for the anticancer activity of the family of ferrocene-based molecules coined ferrocifens, owing to its unique redox properties. The addition of imide entities to the historical ferrociphenol scaffold tremendously enhanced the cytotoxic activity of a large panel of cancer cell cultures and preliminary studies showed that the reduction of one of the carbonyl groups of the imide groups to the corresponding α-hydroxylactams only slightly affected the antiproliferative activity. As a continuation to these studies, we took advantage of the facile conversion of α-hydroxylactams to highly electrophilic *N*-acyliminium ions to graft various substituents to the imide motif of phthalimido ferrocidiphenol. Cell viability studies showed that the newly synthesized compounds showed diverse cytotoxic activities on two breast cancer cell lines, while only one compound was significantly less active on the non-tumorigenic cell line hTERT-RPE1.

## 1. Introduction

Breast cancer is a burden for women health, and about 2.1 million were diagnosed in 2018 worldwide. Even if around 75% of early-stage and non-metastatic conditions are now curable, current treatments fail in the case of advanced and metastatic breast cancers [1]. This situation prompts chemists to propose alternatives to current chemotherapeutic agents, among which multifunctional hybrids of ferrocene currently focus particular attention on [2,3,4,5,6,7]. Our group designed a family of ferrocene-based organometallic compounds coined ferrocifens that were initially built up from hydroxytamoxifen, i.e., the main metabolite of tamoxifen, a molecule given for the adjuvant therapy of hormone-dependent breast cancer. Ferrocifens display potent anticancer activity on cell cultures and In vivo on animal models [8] via an original mechanism of action related to the redox properties of the ferrocene entity. Indeed, the [ferrocene-ene-phenol] motif common to ferrocifens has been identified as the pharmacophore owing to its facile oxidation in cellular medium, yielding electrophilic quinone methides [9] that are in turn able to react with nucleophiles in the biological context [10].

**Figure 1:**
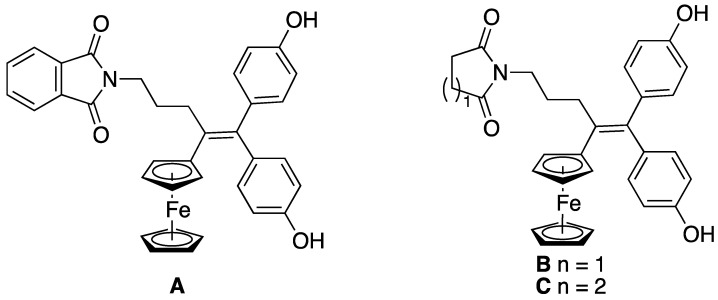
Recently reported imido-ferrocidiphenols **A**–**C** with high anticancer activity.

More recently, we found out that grafting of imide motifs (phthalimide, succinimide, glutarimide) at the end of a propyl chain carried by the central double bond of ferrociphenol (Figure 1) markedly amplified the antiproliferative activity on cell cultures with half maximal inhibitory concentrations (IC_50_) down to 18 nM for compound **A** on the glioblastoma cell model U87 [11] and 145 nM on the triple negative breast cancer cell line MDA-MB-231 [12]. When phthalimide was switched to succinimide or glutarimide (compounds **B** and **C**), the IC_50_ on MDA-MB-231 cells dropped down to respectively 35 and 70 nM, measured after 5 days of incubation [12]. Compound **B** was also tested on the NCI-60 panel of human cancer cells and its mean GI_50_ (half maximal growth inhibition) value was ca. 0.1 µM [13]. Interestingly, an extremely large heterogeneity of response to compound **B** was observed on a panel of 15 glioblastoma patient-derived cell lines, with IC_50_ ranging from 10 nM to ca. 30 µM [14]. In vivo, compound **B** formulated in lipid nanocapsules improved the survival rate and decreased the tumor growth in an orthotopic mouse model of B16F10 melanoma [15].

Mechanistic studies have highlighted that the enhanced cytotoxic activity of imido-ferrocidiphenols was linked to the remarkable stability of the corresponding quinone methides via an unusual lone pair–π interaction between one of the carbonyl groups of the imide and the quinonic cycle [13]. On the whole, the new series of molecules appears as highly promising as anticancer drugs.

Herein we report the synthesis of diversely functionalized ferrocidiphenols derived from compound **A**, using the imide motif as an entry to the grafting of substituents carrying various functional groups via the well-known *N*-acyliminium ions chemistry. Indeed, *N*-acyliminium ions can be generated in situ from α-hydroxyamides using Lewis or Bronsted acids. As powerful electrophiles, these species can be trapped by various nucleophiles inter- [16] or intramolecularly [17,18], yielding α-substituted amides. Standard cell viability assays were performed on the two breast cancer cell lines MCF-7 and MDA-MB-231 and the results were compared with the ones obtained on the non-cancerous cell line hTERT-RPE1.

## 2. Results and Discussion

### 2.1. Synthesis of Ferrocenyl Alkylimides ***3***

**Figure 2:**
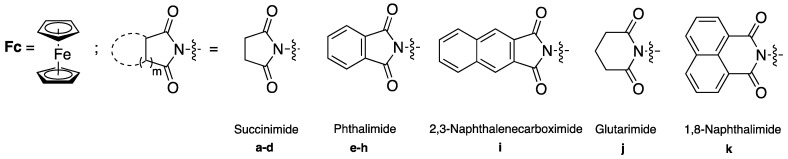
Schematic representation of the substituents cited in this paper and their corresponding numbering.

We carried out a preliminary investigation of the reactivity of *N*-acyliminium ions starting from simple models of the imidopropyl ferrocidiphenols **A**–**C**. Ferrocenyl alkylimides **3** carrying a succinimide, phthalimide, 2,3-naphthalenecarboximide, glutarimide or 1,8-naphthalimide substituent linked to the ferrocene by an alkyl chain of one to four methylene units were prepared with the purpose of investigating the reactivity of the imide groups and the effect of the ferrocenyl group (Figure 2).

**Scheme 1:**
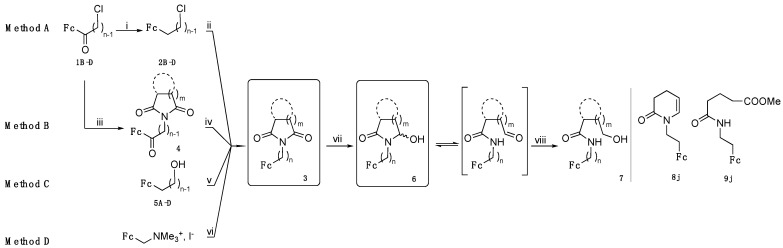
Synthesis of ferrocenyl alkylimides **3** using methods A, B, C, or D; their reduced products α-hydroxylactams **6;** and over-reduced products of amide-alcohols **7**. The enamide **8j** and the amide-ester **9j** were unexpectedly obtained during the reduction of **3j**. Reagents and conditions are i: LiAlH_4_, AlCl_3_, Et_2_O; ii, iii, vi: imide-H, K_2_CO_3_, DMF; iv: HSiEt_3_, TFA, DCM; v: imide-H, PPh_3_, DEAD, THF; vii, viii: NaBH_4_, MeOH.

Ferrocenyl alkylimides of type **3** were synthesized according to four methods A, B, C, and D starting from **1B**–**D** (methods A and B), **5A–D** (method C) or (ferrocenylmethyl)trimethylammonium iodide (method D) as substrates (Scheme 1, Table 1). Ferrocenyl ketones **1B**–**D** were obtained by Friedel-Craft reactions between ferrocene and ω-chloroacyl chlorides [19,20,21]. For method A, ketones **1B***–***D** (with n = 2–4) were first reduced by LiAlH_4_ and AlCl_3_ to afford the ω-chloroalkylferrocenes **2B**–**D** (with n = 2–4) [19,20,21]. Then the imide substituents were introduced by nucleophilic substitution to afford the ferrocenyl alkylimides **3**. On the opposite, for method B, the imide substituents were introduced on compounds **1B**–**D** first by a nucleophilic substitution to yield compounds of type **4**, prior to selective ketone reduction with triethylsilane and TFA.

Method A was the most efficient and general since the ω-chloroalkylferrocene intermediates **2B**–**D** can be prepared in large quantities. On the contrary, yields in ferrocenyl alkylimides **3** synthesized via method B depended on the imide, as shown in Table 1. We previously used ketone reduction to methylene group by triethylsilane and TFA (reaction iv in Scheme 1) for purely organic compounds that were complete in less than 1 day with a good yield (ca. 75%) [22]. Conversely, even if the ketone reduction in compounds **2B**–**D** was selective, since the carbonyl groups of imides were unaffected, the reaction was very slow and incomplete even after 2 weeks. One possible explanation could be the steric hindrance brought by both the ferrocenyl and the imide groups.

Method C, based on the Mitsunobu reaction between ferrocenyl alcohols **5A**–**D** (n = 1–4 [23]), was only applied to prepare ferrocenyl alkylimides **3** carrying phthalimide or succinimide substituents. Ferrocenyl methylimides **3a** and **3e** were also prepared via method D adapted from the literature [24] from the common commercial precursor (ferrocenylmethyl)trimethylammonium iodide using a mixture of phthalimide and potassium carbonate instead of potassium phthalimide.

**Table 1:** Yields in ferrocenyl alkylimides **3** using methods A, B, C, or D depicted in Scheme 1.

Product	n	Method A		Method B		Method C	Method D
		Step i ^a^	Step ii	Step iii	Step iv		
**Succinimide**							
**3a**	1	n.a. ^b^	n.a.	n.a.	n.a.	73	30
**3b**	2	80	92	33	18	66	n.a.
**3c**	3	81	86	33	0	Failed	n.a.
**3d**	4	88	- ^c^	41	7 ^d^	26	n.a.
**Phthalimide**							
**3e**	1	n.a.	n.a.	n.a	n.a	59	77
**3f ^e^**	2	80	79	52	59	53	n.a.
**3g**	3	81	88	45	39	35	n.a.
**3h**	4	88	12	58	38	66	n.a.
**2,3-Naphthalenecarboximide**							
**3i**	2	80	68	-	-	-	n.a.
**Glutarimide**							
**3j**	2	80	80	-	-	-	n.a.
**1,8-Naphthalimide**							
**3k**	2	80	70	-	-	-	n.a.

^a^ yields in compounds **2B**–**D** taken from the literature [19,20,21]; ^b^ not applicable; ^c^ not done; ^d^ value from Ref. [25]; ^e^ compound reported in [26] by a different route.

### 2.2. Synthesis of α-Hydroxylactams ***6***

Reduction of phthalimides **3e–h** with sodium borohydride in methanol afforded the α-hydroxylactams **6e–h** in almost quantitative yield (Scheme 1, Table 2). Conversely, yields were lower for the succinimide and glutarimide derivatives, owing to the known phenomenon of over-reduction. Indeed, in basic medium, α-hydroxylactams **6** are in equilibrium with their amide-aldehyde open form (Scheme 1), whose aldehyde group can be further reduced to the amide-alcohols **7** [27]. Nevertheless, this over-reduction provides a convenient way to prepare compounds of type HO-(CH_2_)_x_-CO-NH-(CH_2_)_y_-R, the imide serving as a protecting group for the final alcohol function. To avoid the over-reduction, Speckamp [27] stated that the reduction of succinimide should be done below 5 °C and glutarimide below −10 °C. In these conditions, reactions were rather slow and we chose to run the reduction reaction at room temperature for all imides for yield comparison (Table 2). For the same reason, we did not add hydrochloric acid to limit the formation of the amide-aldehyde open form and its over-reduction [27]. Of note, the α-methoxylactams **10** were sometimes formed as by-products. We did not attempt to further optimize the reaction conditions for the succinimide derivatives since the α-hydroxylactams were obtained in acceptable yield (33–66%) by this “fast method” and the removal of by-products was easy.

The 2,3-naphthalenecarboximide derivative **3i** was difficult to reduce and the 1,8-naphthalimide **3k** did not react at all. This behavior appears to be a common feature of naphthalimides in these conditions. Furthermore, reduction of the glutarimide derivative **3j** gave a complex mixture, i.e., the expected α-hydroxylactam **6j** and the starting material, **3j** as well as unexpected side products, among which were the enamide **8j** resulting from the dehydration of the α-hydroxylactam, the amide-ester **9j** resulting from glutarimide ring opening by reaction of methanol under basic conditions, and the amide-alcohol **7j** (Scheme 1). Moreover, the α-hydroxylactam **6j** was unstable and not fully characterized. Indeed, after isolation, **6j** was completely decomposed in one night in CDCl_3_, with the appearance of a dark precipitate.

**Scheme 2:**
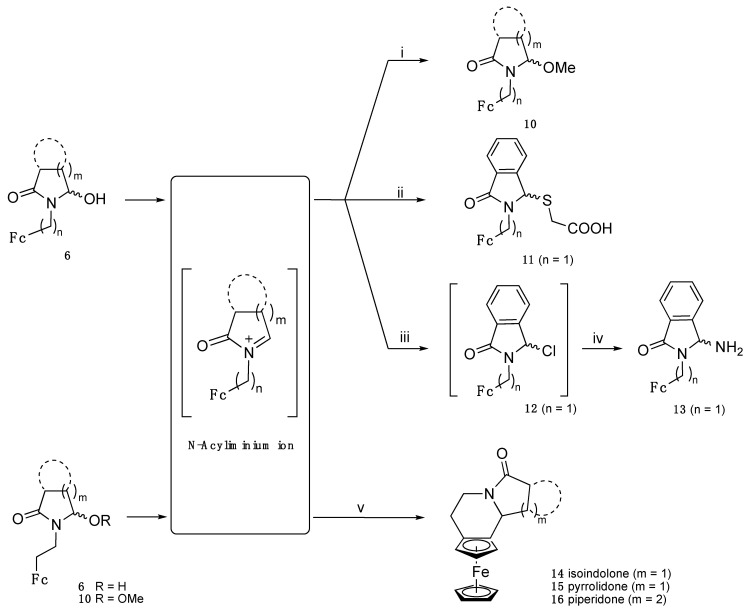
Reactivity of *N*-acyliminium derived from α-hydroxylactams **6**. Reaction conditions are i: TsOH, MeOH, THF; ii: TsOH, thioglycolic acid; iii: ClCOCOCl, DCM; iv: NH_3_; v: TsOH, DCE, 70 °C.

To prevent the formation of this complex mixture and possibly stabilize the unstable **6j**, we tried to convert all the reduced cyclic products into α−methoxylactam **10j** (Scheme 2) by the addition of hydrochloric acid to the reaction medium, 15 min after the addition of sodium borohydride following a method adapted from reference [27]. Indeed, this should hamper basic hydrolysis of the glutarimide and over-reduction of the α-hydroxylactam **6** as stated above. However, the mixture remained still quite complex and the products difficult to separate by chromatography.

### 2.3. Reactivity of N-Acyliminium Ions Derived from α-Hydroxylactams ***6***

α-Hydroxylactams are known precursors of highly electrophilic *N*-acyliminium ions that can be further trapped by various nucleophiles. Herein, the formation of *N*-acyliminium ions by the action of Bronsted acid to α-hydroxylactams **6** was proved via trapping using various nucleophiles. We began by a simple O-nucleophile, namely MeOH, to prepare α−methoxylactams **10** (Scheme 2). However, we noted that the α-hydroxylactams **6** were not very soluble in solvents as DCM or methanol, so they had to be dissolved in THF. Thus, α-methoxylactams **10** were synthesized in mild conditions with a catalytic amount of TsOH at room temperature (Table 2). Most of the reactions went to completion in less than 1 h, if not less than 15 min, except for the α-hydroxylactams **6a** and **6e** (n = 1), where no reaction occurred in 1 day. Compound **10e** was obtained only after heating under reflux for 5–7 h, while an attempt to generate **10a** only gave degradation. The proximity of the ferrocenyl moiety is likely responsible for this lower reactivity. 

Let us note that α−methoxylactams **10** are also precursors of *N*-acyliminium ions with the advantage of being more soluble than α−hydroxylactams **6**, which can be convenient for future reactions (Scheme 2).

The *N*-acyliminium ion derived from the α-hydroxylactam **6e** was also successfully trapped by the S-nucleophile thioglycolic acid under reflux for 8 h to afford **11** in good yield (Scheme 2) following a method adapted from our previous work [28]. Treatment of α-hydroxylactam **6e** with oxalyl chloride gave the moisture-sensitive α-chlorolactam **12** that was immediately converted to α-aminolactam **13** with an overall 59% yield by the addition of ammonia (Scheme 2). Oxalyl chloride was used instead of the more classical reagent thionyl chloride previously employed for the same purpose on a closely related molecule [28] because of the known incompatibility with ferrocenic compounds (S. Top, personal communication).

Electrophilic *N*-acyliminium ions can also be trapped intramolecularly by π-nucleophiles following S_E_Ar reaction to generate polycyclic products [18]. In the ferrocene series, Achari et al. reported the cyclisation of α−hydroxylactam **6f** to the air-sensitive tetracyclic γ-lactam **14** in 79% yield after 2 days in the presence of BF_3_.Et_2_O [26]. In our case, heating of a mixture of α−hydroxylactam **6f** and TsOH to 70 °C in dichloroethane (DCE) also afforded lactam **14** with an 84% yield after only 10 min (Scheme 2). Interestingly the corresponding α-methoxylactam **10f** also afforded lactam **14** in a comparable yield (83%) under the same conditions, even though α−hydroxylactam **6f** was not completely soluble in DCE at 70 °C. ^1^H and ^13^C NMR confirmed the formation of a single diastereoisomer in agreement with the literature [29]. As mentioned, lactam **14** was indeed unstable since its solution in CDCl_3_ turned to green in one night. In the same line, α−methoxylactam **10b** was converted to the tricyclic γ-lactam **15** as a single diastereoisomer (see crystal structure below). Finally, the crude mixture resulting from the reduction of the glutarimide derivative **3j** afforded the tricyclic γ-lactam **16** in 74% yield. This yield was higher than that obtained from **6j** alone, indicating that both α-hydroxylactam **6j** and enamide **8j** contributed to the formation of the *N*-acyliminium ion intermediate. The assumption was confirmed by the disappearance of **8j** observed by TLC of the reaction medium. In the same conditions, and even when eating overnight, **6e** did not give the five-membered ring product. We previously reported that the cyclisation of structurally related compounds (thiophene instead of ferrocene) [30] using TFA at room temperature readily occurred for six-membered ring products but hardly for the five-membered ring ones.

**Figure 3:**
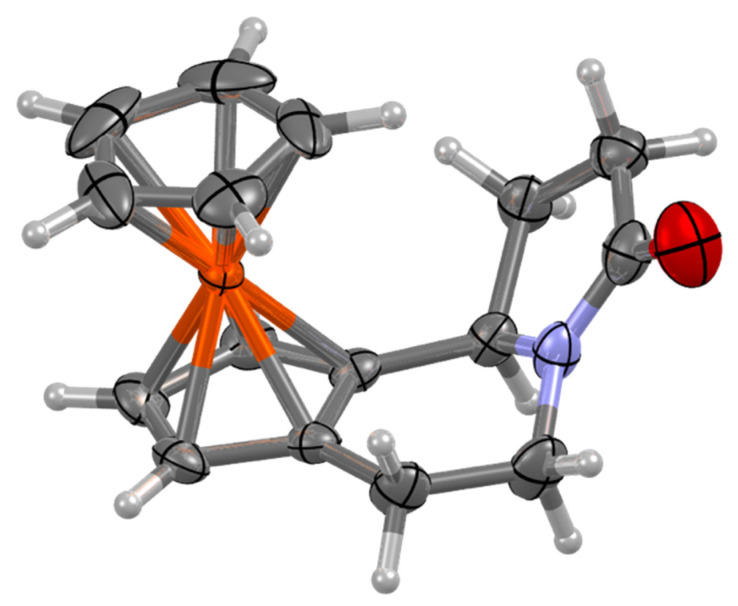
Molecular structure of tricyclic γ-lactam **15**. Ellipsoids drawn at 50% probability.

Single crystals of tricyclic γ-lactam **15** as a racemate were obtained by slow evaporation of a solution of complex in DCM/cyclohexane mixture at room temperature. The X-ray diffraction analysis of **15** confirmed the formation of a single diastereomer (Figure 3).

**Scheme 3:**
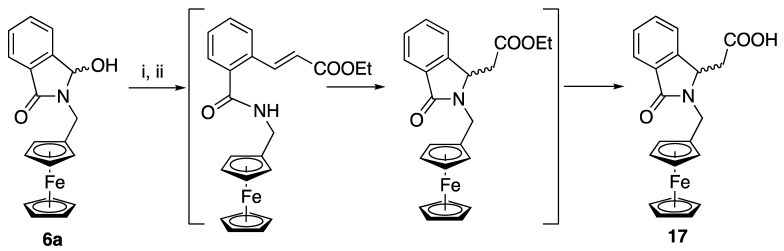
α-hydroxylactam **6a** can also undergo a Wittig reaction. Reagents are i: Ph_3_P = CHCOOEt; ii: K_2_CO_3_.

As mentioned above, α-hydroxylactams **6** are in equilibrium with their corresponding amide-aldehyde open forms. In this respect, the Wittig reaction between **6e** and (carbethoxymethylene) triphenylphosphorane gave the α,β-ethylenic ester that underwent an intramolecular 1,4-Michael addition to form the corresponding cyclic ester, according to a mechanism previously observed for thiophene derivatives [31] that was eventually saponified into the acid **17** in 90% overall yield (Scheme 3).

On the whole, the high electrophilicity of *N*-acyliminium ions derived from imides yielded original ferrocenyl polycyclic γ-lactams as well as various functionalized products, among which compounds **11**, **13**, and **17** than can provide interesting starting points for further conjugation of amino acids or peptides by their C- or N-terminus.

### 2.4. Application to Phthalimido Ferrocidiphenol ***A***

Some of the transformations reported above were applied to ferrocidiphenol **A**. This compound was selected for two reasons. First, reduction of ferrocidiphenol **A** to the corresponding α−hydroxylactam **18** is more efficient as compared to ferrocidiphenol **B**, i.e., 92% vs. 58% yield [25]. Second, we also showed that the IC_50_ of compound **18** on MDA-MB-231 breast cancer cell line was comparable to that of the phthalimide ferrocidiphenol **A** [25].

**Scheme 4:**
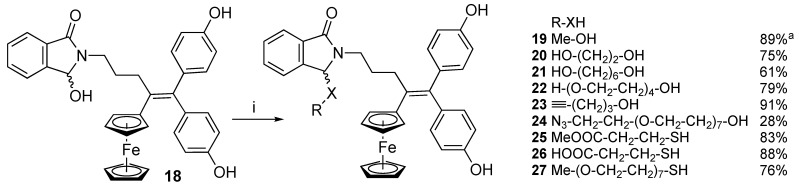
Conversion of α-hydroxylactam **18** to diversely substituted compounds **19–27** via trapping of the *N*-acyliminum ion intermediate with nucleophiles R-XH. Conditions (i): TsOH, R-XH, THF/DCM (THF/methanol for **19**). ^a^ from Ref. [25].

The corresponding *N*-acyliminium ion generated by Brønsted acid treatment of the α−hydroxylactam **18** was readily trapped by methanol to form the α-methoxylactam **19**, as already reported (89% yield) [25]. However, the α−hydroxylactam **18** was recovered by stirring **19** in acetone, with water and hydrochloric acid overnight at room temperature, indicating the reaction was reversible. We extended the range of O-nucleophiles to graft various substituents, for instance linkers carrying useful chemical functions at the other end for future reactions or oligoethylene glycol chains to improve the solubility (Scheme 4). The reaction of **18** with ethylene glycol was performed with 10 eq. diol in the presence of TsOH to prevent double addition to the two hydroxyl groups and displace the equilibrium towards the α−alkyloxylactam. Because of its poor solubility in the THF/DCM mixture, compound **20** precipitated from the reaction medium and was obtained in 75% yield. In the same conditions as for **20**, reaction of **18** with 1,6-hexanediol yielded **21** with a 65% yield. Grafting of the hydroxypentyl chain improved the solubility in organic solvents including DCM.

Due to water insolubility being a known issue for the ferrocidiphenol family, we exploited this strategy to attach substituents that could increase their solubility in a biologically relevant context. Thus, attaching short and monodisperse PEG chains seemed interesting. The tetraethylene glycol chain was readily grafted to **A** to afford **22** in 79% yield at room temperature in less than 3 h. Unfortunately, solubility tests performed by shaking suspensions of compounds **18**, **20**, **22**, **24**, and **27** in water or in PBS overnight at room temperature, followed by RP-HPLC analysis of the supernatants, indicated that none of these compounds were soluble in aqueous medium (see Supplementary Materials). Unexpectedly, compound **20** was poorly soluble in organic solvents, except for DMSO.

For future development in chemical proteomics, we also grafted terminal alkyne and azide functions to ferrocidiphenol **A**. A pentynyl chain was grafted to give compound **23** with a 91% yield. An azide function attached to a short oligoethylene glycol linker was also grafted to the ferrocidiphenol scaffold to increase the water solubility for future bioorthogonal chemistry studies in biologically relevant conditions. The starting azido PEG being expensive, only 1.5 equivalents were added to generate **24**, instead of 10 equivalents for other nucleophiles. Compound **24** was obtained with only a 28% yield as the probable consequence. However, attempts to displace the equilibrium toward the formation of the alkoxylactam by removing water with magnesium sulfate and then adding calcium chloride to the reaction mixture did not improve the conversion. Thus, the relatively low yield is not only due to the equilibrium between the hydroxylactam and the alkoxylactam. An alternative would be to add the α−hydroxylactam **18** in excess to completely consume the expensive azido-PEG-OH, since **18** is easy to recover.

To prevent the reverse hydrolysis reaction, another strategy could consist in replacing the O-nucleophile by an S-nucleophile. Indeed, the formed α-alkylthiolactam should be less likely to hydrolyze. To prove this assumption, we first attempted the reaction with methyl mercaptopropionate or mercaptopropionic acid to form products **25** and **26** in 83% and 88% yield, respectively. In the same line, we grafted a methoxy-terminated short PEG (7 glycol units) to afford compound **27** in 76% yield, using only 1.2 eq. thiol reagent.

**Figure 4:**
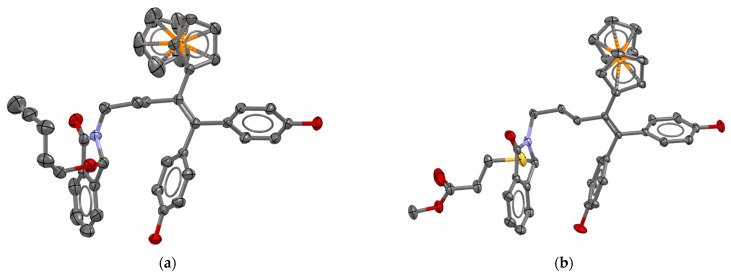
Molecular structures of (**a**) **23** and (**b**) **25** (co-crystallized acetone molecule and hydrogens are omitted for clarity for both compounds). Ellipsoids drawn at 50% probability level.

Single crystals of compounds of **23** and **25** were obtained by slow evaporation of concentrated solutions in acetone-d_6_ at room temperature. X-ray diffraction analysis confirmed the molecular structure of the complexes (Figure 4). A comparison with the previously reported molecular structure of the monophenol analog of **A** [13] showed that the presence of a substituent on the lactam ring did not markedly alter the overall 3D arrangement of the ferrocidiphenol scaffold.

### 2.5. Biological Investigations

#### 2.5.1. Oxidation with HRP and H_2_O_2_

Enzymatic oxidation of **19**, **20**, **22**, **23**, and **25** was performed with a fourfold molar excess of H_2_O_2_ in the presence of HRP at pH 8.1. All compounds were rapidly converted to bright pink adducts (λ_max_ = 571–572 nm, Figure S51). This behavior is reminiscent of that previously observed for a structurally related diphenol complex [32], which lets us conclude that enzymatic oxidation of **19**, **20**, **22**, **23**, and **25** affords the corresponding quinone methide in the anionic phenolate form, regardless of the substituent grafted from the phthalimide ring.

#### 2.5.2. Antiproliferative Activity

A prerequisite to further bioorthogonal modifications is to preserve the biological properties of ferrociphenol compounds. Then, the impact of some of the grafted substituents on the antiproliferative activity was examined by a classical MTT cell viability assay on the two breast cancer cell lines MDA-MB-231 and MCF-7 as well as on the non-tumorigenic cell line hTERT-RPE1 (Table 3). The two cancer cell lines were chosen since most of the previously reported data on ferrocifens were carried out on these cellular models. The experimental protocol is described in Section 3.14., and it is important to emphasize here that the EC_50_ values reported in Table 3 are not to be compared to the IC_50_ values reported in the literature, since the assay conditions are different. For this reason, we included as a reference the ferrocenyl derivative of hydroxytamoxifen **Fc-OH-TAM-3** [8] that is known to be cytotoxic to numerous cell lines. Furthermore, careful analysis of the dose–response curves showed that some of them displayed biphasic features with two inflection points, corresponding to two inhibitory phases at low (<1 µM) and high (>1 µM) concentration ranges. This feature was taken into account when performing data fitting.

Predictably, compound **3h** did not inhibit cell growth for any of the cell models. On the whole, the newly synthesized compounds showed lower EC_50_ values for MDA-MB-231 cells than for MCF-7, except for compound **25** (see below). With respect to **Fc-OH-TAM-3**, grafting different substituents from one of the carbonyl groups of the phthalimide impaired the antiproliferative activity of both cancer cells, especially the hormone-dependent MCF-7 cells. The EC_50_ values of compound **22** were similar for the hTERT-RPE1 and MCF-7 cells but compounds **20** and **25** were twice more toxic than the non-tumorigenic cells. Let us note that the ester group in **25** can be easily hydrolyzed in the biological medium, which might have consequences on its cytotoxicity. Interestingly, compound **23** appears as the most active compound of the series and seems to be selective of cancer cells (6- or 3-fold less toxic on hTERT-RPE1 with respect to MDA-MB-231 and MCF-7, respectively). These results are very promising and provide a good omen for future bioorthogonal reactions and chemical proteomics studies.

## 3. Materials and Methods

### 3.1. General

All ^1^H and ^13^C-NMR spectra were acquired on Bruker 300 and 400 MHz spectrometers. Elemental analysis was performed at the “Service de Micro-analyse ICSN” (Gif sur Yvette, France) or “Service d’Analyses–Chromato-Masse BioCIS-UMR 8076” (Châtenay-Malabry, France). High-resolution mass spectra (HRMS) were performed at the MS^3^ platform of Sorbonne Université. Thin-layer chromatography was performed on silica gel 60 GF254. Purification by column chromatography was performed on the Puriflash 430 system (Interchim) using pre-packed silica gel cartridges (Grace). Ketones **1B**–**D** were synthesized according to [19,20,21]. Ferrocenyl alcohols **5A**–**D** were prepared according to reference [23]. The synthesis of compounds **3d**, **4d**, **18**, and **19** was previously reported in reference [25] and the synthesis of compounds **3f**, **6f** and **14** in reference [26]. Other reagents were obtained from commercial suppliers and used as received.

### 3.2. General Procedure for the Synthesis of Imides ***3*** (Method A) or ***4*** (Method B)

In a flask, a mixture of ferrocenyl compounds **1B**–**D** or **2B**–**D**, imide (3 eq.), potassium carbonate (3 eq.), and DMF (10 mL/mol of substrate) was heated to 80 °C under stirring for 2 days. After cooling to r.t., the mixture was poured into an aqueous solution of sodium hydroxide (3 eq. in 80–100 mL of water/mol substrate) and rapidly extracted (to prevent opening of the imide ring) three times with diethyl ether. Organic layers were combined and washed with water and dried with magnesium sulfate. The solution was concentrated under reduced pressure and the residue was chromatographed on silica gel with DCM/petroleum ether 9:1 mixture for **3b**–**c**,**f**–**k** or dichloromethane for **4b**–**d**,**f**–**h**, affording products as orange-red solids.

### 3.3. General Procedure for the Synthesis of Imides ***3*** by Reduction of ***4*** (Method B)

Imides **4b**–**d**,**f**–**h** were dissolved into dry dichloromethane. Triethylsilane (3.5 eq.) and trifluoroacetic acid (30 eq.) were added and the solution was stirred for 2 weeks at r.t. The solution was slowly poured into an aqueous solution of hydrogen carbonate under stirring, and solid hydrogen carbonate was added until the gas ceased to form. The mixture was extracted three times with dichloromethane and the combined organic layer was washed with water and dried with magnesium sulfate. After concentration under reduced pressure, the crude mixture was chromatographed on silica gel with DCM/petroleum ether 9:1 mixture to afford pure imides **3b**,**d**,**f**–**h** as yellow-orange solids (**3c** failed).

### 3.4. General Procedure for the Synthesis of Imides ***3*** by Mitsunobu Reaction (Method C)

To a mixture of phthalimide or succinimide, triphenylphosphine (1 eq.) and hydroxyalkylferrocene **5A**–**D** (1 eq.) in dry THF (1.5 mL/mmol of imide) were slowly added to diethyl azodicarboxylate (DEAD, 1 eq.) in dry THF (1.5 mL/mmol of imide). The reaction mixture was stirred overnight. The solvent was evaporated under reduced pressure and the residue suspended in Et_2_O. The precipitate was filtered, the solvent was evaporated, and the residue was purified by column chromatography on silica gel with DCM/petroleum ether 9:1 mixture to afford pure imides **3b**–**d**,**f**–**h** as yellow-orange solids.

### 3.5. General Procedure for the Synthesis of Imides ***3*** by Method D

This method is adapted from a procedure described in reference [24], replacing potassium phthalimide by a mixture of phthalimide and potassium carbonate (for **3e**). To (ferrocenylmethyl) trimethylammonium iodide, potassium carbonate and phthalimide or succinimide, DMF was added (10 mL/mmol of iodide), and the mixture was heated to 80 °C overnight. After cooling to room temperature, the mixture was poured into water and was extracted three times with Et_2_O. The combined organic layer was washed with water then dried with magnesium sulfate. After concentration under reduced pressure, the crude mixture was chromatographed on silica gel with a DCM/petroleum ether 9:1 mixture to afford pure imides.

#### 3.5.1. *N*-(2-ferrocenyl-2-oxo-ethyl) Succinimide (**4b**)

*Method B*: From 2-chloro-1-ferrocenyl-1-ethanone **1B** (4 g, 15.24 mmol), potassium carbonate (4.212 g, 30.5 mmol), succinimide (2.265 g, 22.9 mmol). Yield 33%. ^1^H NMR (CDCl_3_): δ 2.83 (s, 4H, succinimide), 4.32 (s, 5H, Cp), 4.56 (t, *J* = 2.0 Hz, 2H, C_5_H_4_), 4.68 (s, 2H, N-CH_2_-CO), 4.81 (t, *J* = 2.0 Hz, 2H, C_5_H_4_). ^13^C NMR (CDCl_3_): δ 28.4 (2CH_2_ succinimide), 45.2 (N-CH_2_-CO), 69.0 (2CH C_5_H_4_), 70.4 (5CH Cp), 72.9 (2CH C_5_H_4_), 75.5 (C C_5_H_4_), 177.0 (CO-N-CO), 194.3 (CO). IR (KBr, ν cm^−1^): 3096, 2965, 2931 (CH, CH_2_), 1703, 1684 (CO). MS (ESI) *m*/*z*: 326 [M + H]^+^. HRMS (ESI, C_16_H_16_FeNO_3_: [M + H]^+^) calcd: 326.0480, found: 326.0468.

#### 3.5.2. *N*-(3-ferrocenyl-3-oxopropyl) Succinimide (**4c**)

*Method B*: from 3-chloro-1-ferrocenyl-propan-1-one **1C** (4.148 g, 15 mmol), potassium carbonate (3.11 g, 22.5 mmol), succinimide (2.973 g, 30 mmol). Yield 33%. ^1^H NMR (CDCl_3_): δ 2.72 (s, 4H, succinimide), 3.04 (t, *J* = 7.5 Hz, 2H, CH_2_CO), 3.90 t, *J* = 7.5 Hz, 2H, CH_2_N), 4.20 (s, 5H, Cp), 4.51 (t, *J* = 2.0 Hz, 2H, C_5_H_4_), 4.76 (t, *J* = 2.0 Hz, 2H, C_5_H_4_). ^13^C NMR (CDCl_3_): δ 28.3 (2CH_2_ succinimide), 34.6 (CH_2_), 36.7 (CH_2_), 69.4 (2CH C_5_H_4_), 70.0 (5CH Cp), 72.7 (2CH C_5_H_4_), 78.5 (C C_5_H_4_), 177.2 (CO-N-CO), 201.4 (CO). IR (KBr, ν cm^−1^): 3094, 3080, 2978, 2952 (CH, CH_2_), 1699, 1658 (CO). MS (ESI) *m*/*z*: 340 [M + H]^+^. HRMS (ESI, C_17_H_18_FeNO_3_: [M + H]^+^) calcd: 340.0636, found: 340.0625.

#### 3.5.3. *N*-(2-ferrocenyl-2-oxoethyl) Phthalimide (**4f**)

*Method B*: from 2-chloro-1-ferrocenyl-1-ethanone **1B** (2.11 g, 8.04 mmol), potassium carbonate (1.111 g, 8 mmol), phthalimide (1.183 g, 8 mmol), yield 52%.

^1^H NMR (CDCl_3_): δ 4.38 (s, 5H, Cp), 4.60 (t, *J* = 2.0 Hz, 2H, C_5_H_4_), 4.87 (t, *J* = 2.0 Hz, 2H, C_5_H_4_), 4.89 (s, 2H, CH_2_), 7.75 (dd, *J* = 5.5 and 3.1 Hz, 2H, phthalimide), 7.90 (dd, *J* = 5.5 and 3.1 Hz, 2H, phthalimide). ^13^C NMR (CDCl_3_): δ 44.7 (CH_2_), 69.1 (2CH C_5_H_4_), 70.5 (5CH Cp), 72.9 (2CH C_5_H_4_), 75.5 (C C_5_H_4_), 123.6 (2CH phthalimide), 132.4 (2C phthalimide), 134.2 (2CH phthalimide), 168.2 (2CO), 195.0 (CO). MS (CI, NH_3_) *m*/*z*: 374 [M + H]^+^. IR (KBr, ν cm^−1^): 1713 (CO), 1684 (CO). HRMS (ESI, C_20_H_16_FeNO_3_: [M + H]^+^) calcd: 374.0480, found: 374.0490.

#### 3.5.4. *N*-(3-ferrocenyl-3-oxopropyl) Phthalimide (**4g**)

*Method B*: from 3-chloro-1-ferrocenyl-propan-1-one **1C** (1.95 g, 7.05 mmol), potassium carbonate (1.949 g, 14.1 mmol), phthalimide (1.556 g, 10.6 mmol), yield 45%. ^1^H NMR (CDCl_3_): δ 2.70 (t, *J* = 7.5 Hz, 2H, CH_2_), 4.11 (t, *J* = 7.5 Hz, 2H, CH_2_), 4.21 (s, 5H, Cp), 4.51 (t, *J* = 2.0 Hz, 2H, C_5_H_4_), 4.78 (t, *J* = 2.0 Hz, 2H, C_5_H_4_), 7.67–7.77 (m, 2H, phthalimide), 7.81–7.92 (m, 2H, phthalimide). ^13^C NMR (CDCl_3_): δ 33.7 (CH_2_), 37.7 (CH_2_), 69.4 (2CH C_5_H_4_), 70.0 (5CH Cp), 72.6 (2CH C_5_H_4_), 78.6 (C C_5_H_4_), 123.4 (2CH phthalimide), 132.2 (2C phthalimide), 134.1 (2CH phthalimide), 168.3 (2CO), 201.4 (CO). IR (ATR, ν cm^−1^): 1712 (CO), 1662 (CO). MS (CI, NH_3_) *m*/*z*: 388 [M + H]^+^, 405 [M + NH_4_]^+^. HRMS (ESI, C_21_H_18_FeNO_3_: [M + H]^+^) calcd: 388.0636, found: 388.0627. Anal. Calcd for C_21_H_17_FeNO_3_(H_2_O)_0.2_: C, 64.53; H, 4.48; N, 3.58. Found: C, 64.56; H, 4.49; N, 3.51.

#### 3.5.5. *N*-(4-ferrocenyl-4-oxobutyl) Phthalimide (**4h**)

*Method B*: from 4-chloro-1-ferrocenyl-1-butanone **1D** (1.19 g, 4.1 mmol), potassium carbonate (0.566 g, 4.1 mmol), phthalimide (0.904 g, 6.1 mmol). Yield 58%. Mp: 137 °C. ^1^H NMR (CDCl_3_): δ 2.00–2.19 (CH_2_), 2.79 (t, *J* = 7.4 Hz, 2H, CH_2_), 3.80 (t, *J* = 7.0 Hz, 2H, CH_2_), 4.18 (s, 5H, Cp), 4.48 (t, *J* = 1.9 Hz, 2H, C_5_H_4_), 4.76 (t, *J* = 1.9 Hz, 2H, C_5_H_4_), 7.66–7.77 (m, 2H, phthalimide), 7.80–7.90 (m, 2H, phthalimide). ^13^C NMR (CDCl_3_): δ 23.6 (CH_2_), 37.0 (CH_2_), 37.8 (CH_2_), 69.4 (2CH C_5_H_4_), 69.9 (5CH Cp), 72.3 (2CH C_5_H_4_), 78.9 (C C_5_H_4_), 123.4 (2CH phthalimide), 132.2 (2C phthalimide), 134.1 (2CH phthalimide), 168.5 (2CO), 203.2 (CO). IR (ATR, ν cm^−1^): 1706 (CO), 1666 (CO). MS (CI, NH_3_) *m*/*z*: 402 [M + H]^+^. HRMS (ESI, C_22_H_20_FeNO_3_: [M + H]^+^) calcd: 402.0793, found: 402.0789. Anal. Calcd for C_22_H_19_FeNO_3_: C, 65.85; H, 4.77; N, 3.49. Found: C, 65.82; H, 4.87; N, 3.49.

#### 3.5.6. *N*-(ferrocenylmethyl) Succinimide (**3a**)

*Method C*: from ferrocenylmethanol **5A** (1.09 g, 5.04 mmol), succinimide (0.5 g, 5 mmol), triphenylphosphine (1.323 g, 5 mmol) and diethyl azodicarboxylate (0.879 g, 0.93 mL, 5 mmol). Yield 73%. *Method D*: from (ferrocenylmethyl)trimethylammonium iodide (3 g, 7.8 mmol), succinimide (1.544 g, 15.6 mmol) and potassium carbonate (2.153 g, 15.6 mmol). Yield 30%. ^1^H NMR (CDCl_3_): δ 2.61 (s, 4H, succinimide), 4.08 (t, *J* = 1.9 Hz, 2H, C_5_H_4_), 4.15 (s, 5H, Cp), 4.31 (t, *J* = 1.9 Hz, 2H, C_5_H_4_), 4.41 (s, 2H, CH_2_). ^13^C NMR (CDCl_3_): δ 28.2 (2CH, succinimide), 38.1 (CH_2_N), 68.4 (2CH, C_5_H_4_), 68.7 (5CH, Cp), 69.9 (2CH, C_5_H_4_), 81.8 (C, C_5_H_4_), 176.8 (2CO). IR (ATR, ν cm^−1^): 1694 (CO). HRMS (ESI, C_15_H_15_FeNO_2_: [M]^+.^) calcd: 297.0447, found: 297.0448.

#### 3.5.7. *N*-(2-ferrocenylethyl) Succinimide (**3b**)

*Method A*: from 2-chloroethylferrocene **5B** (3 g, 12.07 mmol), potassium carbonate (5.005 g, 36.2 mmol), succinimide (3.588 g, 36.2 mmol), yield 92%. *Method B*: from compound **4b** (1.429 g, 4.4 mmol), triethylsilane (1.84 g, 2.53 mL, 15.8 mmol), trifluoroacetic acid (15.535 g, 10.5 mL, 136.2 mmol), yield 18%. *Method C*: from 2-hydroxyethylferrocene (2.301 g, 10 mmol), triphenylphosphine (2.623 g, 10 mmol), diethyl azodicarboxylate (1.742 g, 1.83 mL, 10 mmol), succinimide (0.991 g, 10 mmol). Yield 66%. Mp: 169 °C. ^1^H NMR (CDCl_3_): δ 2.58 (t, *J* = 7.9 Hz, 2H, CH_2_), 2.67 (s, 4H, succinimide), 4.08 (t, *J* = 7.9 Hz, 2H, CH_2_N), 4.08 (s, 4H, C_5_H_4_), 4.13 (s, 5H, Cp). ^13^C NMR (CDCl_3_): δ 27.6 (CH_2_), 28.3 (2CH_2_ succinimide), 38.7 (CH_2_), 67.8 (2CH C_5_H_4_), 68.3 (2CH C_5_H_4_), 68.8 (5CH Cp), 84.5 (C C_5_H_4_), 177.2 (2CO). IR (ATR, ν cm^−1^): 1694 (CO). MS (ESI) *m*/*z*: 311 [M]^+^, 287, 177. HRMS (ESI, C_16_H_17_FeNO_2_: [M]^+^) calcd: 311.0609, found: 311.0622.

#### 3.5.8. *N*-(3-ferrocenylpropyl) Succinimide (**3c**)

*Method A*: from 3-chloropropylferrocene **5B** (0.583 g, 2.22 mmol), potassium carbonate (0.614 g, 4.4 mmol), succinimide (0.44 g, 4.4 mmol). Yield 86%. *Method B*: Failed (starting product **4c** recovered). *Method C*: Failed. ^1^H NMR (CDCl_3_): δ 1.65–1.87 (m, 2H, CH_2_), 2.30 (t, *J* = 7.7 Hz, 2H, CH_2_), 2.60 (s, 4H, CH_2_-CH_2_ succinimide), 3.50 (t, *J* = 7.3 Hz, 2H, CH_2_N), 4.01 (s, 2H, C_5_H_4_), 4.06 (s, 7H, C_5_H_4_ + Cp). ^13^C NMR (CDCl_3_): δ 26.9 (CH_2_), 28.1 (CH_2_-CH_2_ succinimide), 28.6 (CH_2_), 38.8 (CH_2_), 67.2 (2CH C_5_H_4_), 68.0 (2CH C_5_H_4_), 68.6 (5CH Cp), 87.9 (C C_5_H_4_), 177.2 (2CO). IR (ATR, ν cm^−1^): 1688 (CO). HRMS (ESI, C_17_H_19_FeNO_2_: [M]^+.^) calcd: 325.0760, found: 325.076.

#### 3.5.9. *N*-(ferrocenylmethyl) Phthalimide (**3e**)

The spectroscopic data were in agreement with reference [24].

*Method C*: from ferrocenylmethanol **5A** (1 g, 4.63 mmol), triphenylphosphine (1.214 g, 4.6 mmol), diethyl azodicarboxylate (0.806 g, 0.85 mL, 4.6 mmol), phthalimide (0.681 g, 4.6 mmol). Yield 59%. *Method D*: from (ferrocenylmethyl) trimethylammonium iodide (7.5 g, 19.5 mmol), phthalimide (1.91 g, 13 mmol) and potassium carbonate (1.795 g, 13 mmol), yield 77%. MS (EI, 70 eV) *m*/*z*: 345 [M]^+^., 280 [M − Cp]^+^, 202, 158, 121 [CpFe]^+^. IR (ATR, ν cm^−1^): 1705 (CO). Anal. Calcd for C_19_H_15_FeNO_2_: C, 66.11; H, 4.38; N, 4.05. Found: C, 65.77; H, 4.33; N, 4.08.

#### 3.5.10. *N*-(2-ferrocenylethyl) Phthalimide (**3f**)

This compound has been reported in the literature using another pathway but NMR signals were not attributed [26].

*Method A*: from 2-chloroethylferrocene **5B** (3 g, 12.07 mmol), potassium carbonate (5.005 g, 36.2 mmol), phthalimide (5.328 g, 36.2 mmol). Yield 79%. *Method B*: from compound **4f** (1.752 g, 4.69 mmol), triethylsilane (1.638 g, 2.25 mL, 14.1 mmol), trifluoroacetic acid (10.706 g, 7.23 mL, 93.9 mmol). Yield 59%. *Method C*: from 2-hydroxyethylferrocene (1.61 g, 7 mmol), triphenylphosphine (1.835 g, 7 mmol), diethyl azodicarboxylate (1.219 g, 1.28 mL, 7 mmol), phthalimide (1.03 g, 7 mmol). Yield 53%. ^1^H NMR (CDCl_3_): δ 2.70 (t, *J* = 7.8 Hz, 2H, CH_2_), 3.85 (t, *J* = 7.8 Hz, 2H, CH_2_), 4.07 (t, *J* = 1.7 Hz, 2H, C_5_H_4_), 4.11 (t, *J* = 1.7 Hz, 2H, C_5_H_4_), 4.14 (s, 5H, Cp), 7.66–7.77 (m, 2H, phthalimide), 7.80–7.88 (m, 2H, phthalimide). ^13^C NMR (CDCl_3_): δ 28.6 (CH_2_), 38.9 (CH_2_), 67.8 (2CH C_5_H_4_), 68.3 (2CH C_5_H_4_), 68.7 (5CH Cp), 84.7 (C C_5_H_4_), 123.3 (2CH phthalimide), 132.2 (2C phthalimide), 134.0 (2CH phthalimide), 168.3 (2CO). IR (ATR, ν cm^−1^): 1707 (CO). MS (EI, 70 eV) *m*/*z*: 359 [M]^+^, 294 [M - Cp]^+^, 199 [FcCH_2_]^+^, 121 [CpFe]^+^. HRMS (ESI, C_20_H_17_FeNO_2_: [M]^+.^) calcd: 359.0609, found: 359.0608. Anal. Calcd for C_20_H_17_FeNO_2_: C, 66.87; H, 4.77; N, 3.89. Found: C, 66.49; H, 4.78; N, 3.87.

#### 3.5.11. *N*-(3-ferrocenylpropyl) Phthalimide (**3g**)

*Method A*: from 3-chloropropylferrocene **2C** (0.583 g, 2.22 mmol), potassium carbonate (0.614 g, 4.4 mmol), phthalimide (0.653 g, 4.4 mmol), yield 88%. *Method B*: From compound **4g** (4 g, 10.33 mmol), triethylsilane (4.204 g, 5.78 mL, 36.2 mmol), trifluoroacetic acid (35.336 g, 23.88 mL, 309.9 mmol), yield 39%. *Method C*: from 3-hydroxypropylferrocene (1.14 g, 4.67 mmol), triphenylphosphine (1.47 g, 5.6 mmol), diethyl azodicarboxylate (0.976 g, 1.03 mL, 5.6 mmol), phthalimide (0.825 g, 5.6 mmol). Yield 35%. Mp: 99–100 °C. ^1^H NMR (CDCl_3_): δ 1.84–2.00 (m, 2H, CH_2_), 2.40 (t, *J* = 7.4 Hz, 2H, CH_2_), 2.73 (t, *J* = 7.2 Hz, 2H, CH_2_), 4.03 (t, *J* = 1.8 Hz, 2H, C_5_H_4_), 4.05–4.11 (m, 7H, C_5_H_4_ + Cp), 7.66–7.76 (m, 2H, phthalimide), 7.80–7.90 (m, 2H, phthalimide). ^13^C NMR (CDCl_3_): δ 27.0 (CH_2_), 29.8 (CH_2_), 38.1 (CH_2_), 67.7 (2CH C_5_H_4_), 68.4 (2CH C_5_H_4_), 69.0 (5CH Cp), 88.6 (C C_5_H_4_), 123.3 (2CH phthalimide), 132.3 (2C phthalimide), 134.0 (2CH phthalimide), 168.5 (2CO). IR (ATR, ν cm^−1^): 1707 (CO). MS (CI, NH_3_) *m*/*z*: 374 [M + H]^+^, 391 [M + NH_4_]^+^. HRMS (ESI, C_21_H_19_FeNO_2_: [M]^+.^) calcd: 373.0765, found: 373.0777.

#### 3.5.12. *N*-(4-ferrocenylbutyl) Phthalimide (**3h**)

*Method A*: from 4-chlorobutylferrocene **2D** (1.659 g, 6 mmol), potassium carbonate (1.659 g, 12 mmol), phthalimide (1.766 g, 12 mmol). Yield 12%. *Method B*: from compound **4h** (2.711 g, 6.76 mmol), triethylsilane (2.846 g, 3.91 mL, 24.5 mmol), TFA (23.946 g, 16.18 mL, 210 mmol), yield 38%. *Method C*: from 4-hydroxybutylferrocene (1.427 g, 5.53 mmol), triphenylphosphine (1.74 g, 6.6 mmol), diethyl azodicarboxylate (1.156 g, 1.22 mL, 6.6 mmol), phthalimide (0.976 g, 6.6 mmol). Yield 66%. ^1^H NMR (CDCl_3_): δ 1.47–1.62 (m, 2H, CH_2_), 1.64–1.78 (m, 2H, CH_2_), 2.36 (t, *J* = 7.6 Hz, 2H, CH_2_), 3.69 (t, *J* = 7.2 Hz, 2H, CH_2_), 4.02 (t, *J* = 2.0 Hz, 2H, C_5_H_4_), 4.04 (t, *J* = 2.0 Hz, 2H, C_5_H_4_), 4.07 (s, 5H, Cp), 7.64–7.75 (m, 2H, phthalimide), 7.78–7.88 (m, 2H, phthalimide). ^13^C NMR (CDCl_3_): δ 28.4 (CH_2_), 28.5 (CH_2_), 29.2 (CH_2_), 37.9 (CH_2_), 67.8 (2CH C_5_H_4_), 68.8 (2CH C_5_H_4_), 69.3 (5CH Cp), 89.6 (C C_5_H_4_), 123.3 (2CH phthalimide), 132.2 (2C phthalimide), 134.0 (2CH phthalimide), 168.5 (2CO). IR (ATR, ν cm^−1^): 1706 (CO). MS (EI, 70 eV) *m*/*z*: 387 [M]^+.^, 320, 202, 199, 158, 121 [CpFe]^+^, 56. HRMS (ESI, C_22_H_21_FeNO_2_: [M]^+^) calcd: 387.0922, found: 387.0914.

#### 3.5.13. *N*-(2-ferrocenylethyl)-2,3-naphthalenedicarboximide (**3i**)

*Method A*: from 2-chloroethylferrocene **2B** (0.5193 g, 2.09 mmol), potassium carbonate (0.578 g, 4.2 mmol), 2,3-naphthalenedicarboximide (0.412 g, 2.1 mmol), yield 68%. ^1^H NMR (CDCl_3_): δ 2.75 (t, *J* = 7.6 Hz, 2H, CH_2_), 3.92 (t, *J* = 7.6 Hz, 2H, CH_2_), 4.07 (t, *J* = 1.9 Hz, 2H, C_5_H_4_), 4.12 (t, *J* = 1.9 Hz, 2H, C_5_H_4_), 4.14 (s, 5H, Cp), 7.76–7.74 (m, 2H, naphthalene), 7.97–8.09 (m, 2H, naphthalene), 8.31 (s, 2H, naphthalene). ^13^C NMR (CDCl_3_): δ 28.5 (CH_2_), 39.2 (CH_2_), 67.7 (2CH C_5_H_4_), 68.3 (2CH C_5_H_4_), 68.7 (5CH Cp), 84.7 (C C_5_H_4_), 124.7 (2CH naphthalene), 128.0 (2C naphthalene), 129.2 (2CH naphthalene), 130.4 (2CH naphthalene), 135.6 (2C naphthalene), 168.0 (2CO). IR (ATR, ν cm^−1^): 1700 (CO). HRMS (ESI, C_24_H_19_FeNO_2_: [M]^+^) calcd: 409.0760, found: 409.076.

#### 3.5.14. *N*-(2-ferrocenylethyl) Glutarimide (**3j**)

*Method A*: from 2-chloroethylferrocene **2B** (3 g, 12.07 mmol), potassium carbonate (5.005 g, 36.2 mmol), glutarimide (4.096 g, 36.2 mmol). Yield 80%. Mp: 132 °C. ^1^H NMR (CDCl_3_): δ 1.91 (quin, *J* = 6.5 Hz, 2H, CH_2_ glutarimide), 2.52 (t, *J* = 7.9 Hz, 2H, CH_2_), 2.62 (d, t = 6.5 Hz, 4H, glutarimide), 3.93 (t, *J* = 7.9 Hz, 2H, CH_2_N), 4.05 (t, *J* = 1.8 Hz, 2H, C_5_H_4_), 4.08 (t, *J* = 1.8 Hz, 2H, C_5_H_4_), 4.13 (s, 5H, Cp). ^13^C NMR (CDCl_3_): δ 17.2 (CH_2_ glutarimide), 27.7 (CH_2_), 32.9 (2CH_2_ glutarimide), 40.2 (CH_2_N), 67.5 (2CH C_5_H_4_), 68.3 (2CH C_5_H_4_), 68.6 (5CH Cp), 85.1 (C C_5_H_4_), 172.3 (2CO). IR (ATR, ν cm^−1^): 1667 (CO). HRMS (ESI, C_17_H_19_FeNO_2_: [M]^+^) calcd: 325.0760, found: 325.076.

#### 3.5.15. 2-(2-Ferrocenylethyl)-1H-Benzo[de]isoquinoline-1,3(2H)-dione (**3k**)

*Method A*: from 2-chloroethylferrocene **2B** (1.343 g, 5.4 mmol), potassium carbonate (1.494 g, 10.8 mmol), 1,8-naphthalimide (2.131 g, 10.8 mmol), yield 70%. Mp: 193–194 °C. ^1^H NMR (CDCl_3_): δ 2.74 (t, *J* = 8.1 Hz, 2H, CH_2_), 4.09 (t, *J* = 1.7 Hz, 2H C_5_H_4_), 4.16–4.22 (m, 7H, Cp + C_5_H_4_), 4.36 (t, *J* = 8.1 Hz, 2H, CH_2_N), 7.76 (dd, *J* = 8.2 and 7.2 Hz, 2H, naphthalene), 8.21 (dd, *J* = 8.2 and 1.1 Hz, 2H, naphthalene), (dd, *J* = 7.2 and 1.1 Hz, 2H, naphthalene). ^13^C NMR (CDCl_3_): δ 27.9 (CH_2_), 41.2 (CH_2_), 67.6 (2CH C_5_H_4_), 68.4 (2CH C_5_H_4_), 68.8 (5CH Cp), 85.4 (C C_5_H_4_), 122.8 (2C naphthalene), 127.1 (2CH naphthalene), 128.3 (C naphthalene), 131.3 (2CH naphthalene), 131.7 (C naphthalene), 134.1 (2CH naphthalene), 164.2 (2CO). IR (ATR, ν cm^−1^): 1695, 1654 (CO). HRMS (ESI, C_24_H_19_FeNO_2_: [M]^+^) calcd: 409.0760, found: 409.0762.

### 3.6. General Procedure for the Reduction of Imides ***3*** into α-Hydroxylactams ***6***

In a flask, imides **3** were dissolved in a minimum quantity of THF, then methanol (approx. 50 mL/mmol of imide, the flask should not be filled more than 2/3 of its capacity because of gas emission) was added and stirring was started. Solid sodium borohydride was added portionwise (10–15 eq. each 10 min for 40 min, with control of the amount of emitted gas). The mixture was poured into a sodium hydrogen carbonate solution and extracted twice with DCM. The combined organic layer was washed with water and dried with magnesium sulfate. The solution was concentrated under reduced pressure and the residue was chromatographed on silica gel with dichloromethane as eluent, affording the α-hydroxylactams **6** as orange-yellow solids.

#### 3.6.1. 1-(Ferrocenylmethyl)-5-hydroxy-2-pyrrolidone (**6a**)

From **3a**, yield 66%. ^1^H NMR (DMSO-d_6_): δ 1.56–1.74 (m, 1H, CH_2_), 2.01–2.18 (m, 2H, CH_2_), 2.23–2.42 (m, 1H, CH_2_), 3.81 (d, *J* = 14.5 Hz, 1H, CH_2_N), 4.08 (s broad, 2H, C_5_H_4_), 4.15 (s broad, 1H, C_5_H_4_), 4.18 (s, 5H, Cp), 4.25 (s broad, 1H, C_5_H_4_), 4.39 (d, *J* = 14.5 Hz, 1H, CH_2_N), 5.00 (m, 1H, N-CH-O-), 6.02 (d, *J* = 7.2 Hz, 1H, OH). ^13^C NMR (DMSO-d_6_): δ 27.4 (CH_2_), 28.5 (CH_2_), 37.7 (CH_2_N), 67.4 (CH C_5_H_4_), 67.8 (CH C_5_H_4_), 68.4 (5CH Cp), 68.5 (CH C_5_H_4_), 68.9 (CH C_5_H_4_), 80.9 (>N-CH-O-), 83.4 (C C_5_H_4_), 172.7 (CO). IR (ATR, ν cm^−1^): 1646 (CO). HRMS (ESI, C_15_H_17_FeNO_2_: [M]^+^) calcd: 299.0603, found: 299.0604.

#### 3.6.2. *N*-(ferrocenylmethyl)-4-hydroxy-butanamide (**7a**)

This compound was obtained as a by-product during the synthesis of **6a** in 23% yield. ^1^H NMR (DMSO-d_6_): δ 1.67 (m, 2H, CH_2_), 2.13 (t, *J* = 7.5 Hz, 2H, CH_2_CO), 3.38 (m, 2H, CH_2_O), 3.97 (d, *J* = 5.8 Hz, 2H, CH_2_N), 4.07 (t, *J* = 1.8 Hz, 2H, C_5_H_4_), 4.15 (s broad, 7H, Cp + C_5_H_4_), 4.46 (t, *J* = 5.2 Hz, 1H, OH), 7.96 (t, *J* = 5.8 Hz, 1H, NH). ^13^C NMR (DMSO-d_6_): δ 28.7 (CH_2_), 32.1 (CH_2_-CO), 37.4 (CH_2_N), 60.4 (CH_2_O), 67.2 (2CH C_5_H_4_), 67.8 (2CH C_5_H_4_), 68.3 (5CH Cp), 86.4 (C C_5_H_4_), 171.6 (CO). IR (ATR, ν cm^−1^): 1642 (CO). HRMS (ESI, C_15_H_19_FeNNaO_2_: [M + Na]^+^) calcd: 324.065738, found: 324.0654.

#### 3.6.3. 1-(2-Ferrocenylethyl)-5-hydroxy-2-pyrrolidone (**6b**)

From **3b**, yield 33%. ^1^H NMR (DMSO-d_6_): δ 1.46–1.62 (m, 1H, CH_2_), 1.62–1.80 (m, 2H, CH_2_), 1.80–2.04 (m, 1H, CH_2_), 2.10–2.31 (m, 2H, CH_2_), 3.09–3.30 (m, 1H, N-CH_2_), 3.54–3.76 (m, 1H, N-CH_2_), 3.94–4.33 (m, 9H, CpFeC_5_H_4_), 4.62–4.84 (m, 1H, N-CH-O-), 5.85 (d, *J* = 6.4 Hz, 1H, OH). ^13^C NMR (DMSO-d_6_): δ 27.3 (CH_2_), 30.9 (CH_2_), 32.2 (CH_2_), 45.1 (CH_2_N), 66.9 (CH C_5_H_4_), 67.0 (CH C_5_H_4_), 67.5 (CH C_5_H_4_), 67.8 (CH C_5_H_4_), 68.3 (5CH Cp), 78.8 (C C_5_H_4_), 86.0 (>N-CH-O-), 168.7 (CO). 

#### 3.6.4. 1-(3-ferrocenylpropyl)-5-hydroxy-2-pyrrolidone (**6c**)

From **3c**, yield 61%. ^1^H NMR (CDCl_3_): δ 1.60–1.84 (m, 2H, CH_2_), 1.84–1.98 (m, 1H, CH_2_), 1.98–2.16 (m, 1H, CH_2_), 2.17–2.37 (m, 3H, CH_2_), 2.38–2.57 (m, 1H, CH_2_), 3.02–3.19 (m, 1H, N-CH_2_), 3.32–3.56 (m, 1H, N-CH_2_), 3.94–4.19 (m, 9H, CpFeC_5_H_4_), 4.88 (ddd, *J* = 8.1, 6.3, 1.6 Hz, 1H, N-CH-O-). ^13^C NMR (CDCl_3_): δ 24.9 (CH_2_), 27.1 (CH_2_), 28.9 (CH_2_), 29.1 (CH_2_), 40.5 (CH_2_N), 67.2 (CH C_5_H_4_), 67.3 (CH C_5_H_4_), 68.0 (2CH C_5_H_4_), 68.6 (5CH Cp), 88.4 (C C_5_H_4_), 89.2 (>N-CH-O-), 175.0 (CO). IR (ATR, ν cm^−1^): 1650 (CO). HRMS (ESI, C_17_H_21_FeNNaO_2_: [M + Na]^+^) calcd: 350.081388, found: 350.0813.

#### 3.6.5. 2,3-Dihydro-3-hydroxy-2-(ferrocenylmethyl)-1*H*-isoindol-1-one (**6e**)

From **3e**, yield 99%. Mp: 246 °C. ^1^H NMR (DMSO-d_6_): δ 4.04–4.14 (m, 3H, CH_2_ + 2C_5_H_4_), 4.19–4.25 (m, 6H, Cp + C_5_H_4_), 4.29–4.34 (m, 1H, C_5_H_4_), 4.67 (d, *J* = 14.7 Hz, 1H, CH_2_), 5.69 (d, *J* = 8.9 Hz, 1H, CH-O), 6.69 (d, *J* = 8.9 Hz, 1H, OH), 7.46–7.67 (m, 4H, CH_arom_). ^13^C NMR (DMSO-d_6_): δ 37.6 (CH_2_), 67.6 (CH C_5_H_4_), 68.0 (CH C_5_H_4_), 68.5 (5CH Cp), 68.6 (CH C_5_H_4_), 69.0 (CH C_5_H_4_), 79.9 (CH-O), 83.7 (C C_5_H_4_), 122.4 (CH phthalimide), 123.7 (CH phthalimide), 129.4 (CH phthalimide), 131.5 (C phthalimide), 132.0 (CH phthalimide), 144.7 (CH phthalimide), 165.4 (CO). IR (ATR, ν cm^−1^): 1661 (CO). MS (CI, NH_3_) *m*/*z*: 348 [M + H]^+^, 199 [FcCH_2_]^+^, 365 [M + NH_4_]^+^. Anal. Calcd for C_19_H_17_FeNO_2_: C, 65.72; H, 4.93; N, 4.03. Found: C, 65.32; H, 4.77; N, 3.95.

#### 3.6.6. 2,3-Dihydro-3-hydroxy-2-(2-ferrocenylethyl)-1*H*-isoindol-1-one (**6f**)

From **3f**. This compound has been reported in the literature using another method, but NMR signals were not attributed [26]. Yield 99%. ^1^H NMR (DMSO-d_6_): δ 2.43–2.79 (m, 2H, CH_2_), 3.82–3.60 (m, 1H, CH_2_N), 3.70–3.88 (m, 1H, CH_2_N), 4.02–4.22 (m, 9H, CpFeC_5_H_4_), 5.79 (d, *J* = 9.0 Hz, 1H, N-CH-O), 6.62 (d, *J* = 9.0 Hz, 1H, OH), 7.44–7.74 (m, 4H, isoindole). ^13^C NMR (DMSO-d_6_): δ 27.7 (CH_2_), 39.5 (CH_2_), 67.1 (CH C_5_H_4_), 67.2 (CH C_5_H_4_), 67.7 (CH C_5_H_4_), 67.8 (CH C_5_H_4_), 68.4 (5CH Cp), 80.8 (N-CH-O), 85.6 (C C_5_H_4_), 122.4 (CH isoindole), 123.5 (CH isoindole), 129.3 (CH isoindole), 131.8 (CH isoindole), 131.9 (C isoindole), 144.9 (C isoindole), 165.8 (CO). IR (KBr, ν cm^−1^): 1671 (CO). MS (EI, 70 eV) *m*/*z*: 361 [M]^+^, 278, 212, 199, 121 [CpFe]^+^. HRMS (ESI, C_20_H_19_FeNO_2_: [M]^+^) calcd: 361.0765, found: 361.0756. 

#### 3.6.7. 2,3-Dihydro-3-hydroxy-2-(3-ferrocenylpropyl)-1*H*-isoindol-1-one (**6g**)

From **3g**, yield 90%. ^1^H NMR (DMSO-d_6_): δ 1.69–1.96 (m, 2H, CH_2_), 2.32 (t, *J* = 7.8 Hz, 2H, CH_2_), 3.27–3.42 (m, 1H, CH_2_N), 3.52–3.68 (m, 1H, CH_2_N), 3.98–4.19 (m, 9H, CpFeC_5_H_4_), 5.85 (d, *J* = 9.0 Hz, 1H, N-CH-O), 6.60 (d, *J* = 9.0 Hz, 1H, OH), 7.47–7.70 (m, 4H, isoindole). ^13^C NMR (DMSO-d_6_): δ 26.4 (CH_2_), 29.1 (CH_2_), 38.8 (CH_2_), 66.8 (2CH C_5_H_4_), 67.7 (2CH C_5_H_4_), 68.3 (5CH Cp), 80.8 (N-CH-O), 88.4 (C C_5_H_4_), 122.2 (CH isoindole), 123.5 (CH isoindole), 129.3 (CH isoindole), 131.8 (CH isoindole + C isoindole), 145.0 (C isoindole), 166.0 (CO). IR (ATR, ν cm^−1^): 1675 (CO). MS (EI, 70 eV) *m*/*z*: 375 [M]^+^, 310, 292, 266, 264, 121 [CpFe]^+^. HRMS (ESI, C_21_H_21_FeNO_2_: [M]^+^) calcd: 375.0922, found: 375.0925.

#### 3.6.8. 2,3-Dihydro-3-hydroxy-2-(4-ferrocenylbutyl)-1*H*-isoindol-1-one (**6h**)

From **3h**, yield 89%. ^1^H NMR (DMSO-d_6_): δ 1.39–1.74 (m, 4H, CH_2_-CH_2_), 2.24–2.44 (m, 2H, CH_2_), 3.22–3.75 (m, 1H, CH_2_N), 3.51–3.68 (m, 1H, CH_2_N), 3.98–4.12 (m, 9H, CpFeC_5_H_4_), 5.81 (d, *J* = 8.9 Hz, 1H, N-CH-O), 6.59 (d, *J* = 8.9 Hz, 1H, OH), 7.45–7.69 (m, 4H, isoindole). ^13^C NMR (DMSO-d_6_): δ 27.8 (CH_2_), 28.1 (CH_2_), 28.6 (CH_2_), 38.4 (CH_2_), 66.8 (2CH C_5_H_4_), 67.78 (CH C_5_H_4_), 67.83 (CH C_5_H_4_), 68.3 (5CH Cp), 80.6 (N-CH-O), 88.7 (C C_5_H_4_), 122.2 (CH isoindole), 123.5 (CH isoindole), 129.3 (CH isoindole), 131.8 (CH isoindole + C isoindole), 144.9 (C isoindole), 166.0 (CO). MS (EI, 70 eV) *m*/*z*: 389 [M]^+^, 324, 280, 121 [CpFe]^+^. HRMS (ESI, C_22_H_23_FeNO_2_: [M]^+^) calcd: 389.1078, found: 389.1080.

#### 3.6.9. 2-(2-Ferrocenylethyl)-1*H*-3-hydroxy-benzo[f]isoindole-1(2*H*)-one (**6i**)

From **3i**, yield 12%. ^1^H NMR (DMSO-d_6_): δ 2.54–2.67 (m, 1H, CH_2_), 2.67–2.80 (m, 1H, CH_2_), 3.47–3.62 (m, 1H, CH_2_N), 3.47–3.62 (m, 1H, CH_2_N), 3.78–3.91 (m, 1H, CH_2_N), 4.03–4.10 (m, 2H, C_5_H_4_), 4.13 (s, 1H, C_5_H_4_), 4.18 (s, 6H, Cp + C_5_H_4_), 5.95 (d, *J* = 9.0 Hz, 1H, O-CH-N), 6.71 (d, *J* = 9.0 Hz, 1H, OH), 7.54–7.70 (m, 2H, naphthalene), 8.03–8.20 (m, 3H, naphthalene), 8.28 (s, 1H, naphthalene). ^13^C NMR (DMSO-d_6_): δ 27.6 (CH_2_), 39.6 (CH_2_N), 67.10 (CH C_5_H_4_), 67.14 (CH C_5_H_4_), 67.70 (CH C_5_H_4_), 67.77 (CH C_5_H_4_), 68.4 (5CH Cp), 80.8 (O-CH-N), 85.6 (C C_5_H_4_), 122.3 (CH naphthalene), 122.6 (CH naphthalene), 126.7 (CH naphthalene), 127.7 (CH naphthalene), 128.4 (CH naphthalene), 129.4 (CH naphthalene), 129.8 (C naphthalene), 133.1 (CH naphthalene), 134.8 (C naphthalene), 140.8 (CH naphthalene), 165.6 (CO). IR (ATR, ν cm^−1^): 1661 (CO), 1644. HRMS (ESI, C_24_H_21_FeNO_2_: [M]^+^) calcd: 411.0916, found: 411.0912.

#### 3.6.10. 1-(2-Ferrocenylethyl)-6-hydroxy-2-piperidone (**6j**)

From **3j**. This compound was obtained with a yield up to 47% depending on the experiment. It is unstable and degraded before full characterization and only the ^1^H NMR could be obtained. ^1^H NMR (CDCl_3_): δ 1.55–1.71 (m, 1H, CH_2_), 1.71–1.86 (m, 2H, CH_2_), 1.88–2.09 (m, 1H, CH_2_), 2.18–2.51 (m, 2H, CH_2_), 2.62 (t, *J* = 7.3 Hz, 2H, CH_2_), 3.22–3.48 (m, 2H, CH_2_ + OH), 3.61–3.80 (m, 1H, CH_2_), 4.06 (s, 4H, C_5_H_4_), 4.11 (s, 5H, Cp), 4.68 (s broad, 1H, CH-O).

#### 3.6.11. *N*-(2-ferrocenylethyl)-5-hydroxy-pentanamide (**7j**)

This compound was obtained as a by-product during the synthesis of **6j** with a yield up to 46% depending on the experiment. ^1^H NMR (CDCl_3_): δ 1.48–1.65 (m, 2H, CH_2_), 1.65–1.82 (m, 2H, CH_2_), 2.04 (s broad, 1H, OH), 2.20 (t, *J* = 7.1 Hz, 2H, CH_2_), 2.53 (t, *J* = 7.0 Hz, 2H, CH_2_), 3.37 (q, *J* = 6.6 Hz, 1H, CH_2_), 3.64 (t, *J* = 6.1 Hz, 1H), 4.10 (s, 4H, C_5_H_4_), 4.14 (s, 5H, Cp), 5.66 (s broad, 1H, NH). ^13^C NMR (CDCl_3_): δ 21.8 (CH_2_), 29.8 (CH_2_), 32.1 (CH_2_), 36.2 (CH_2_), 40.7 (CH_2_), 62.1 (CH_2_), 67.9 (2CH C_5_H_4_), 68.5 (2CH C_5_H_4_), 69.0 (5CH Cp), 85.7 (C C_5_H_4_), 173.2 (CO). IR (ATR, ν cm-1): 1646 (CO). HRMS (ESI, C_17_H_24_FeNO_2_: [M + H]^+^) calcd: 330.1151, found: 330.1151.

#### 3.6.12. 1-(2-Ferrocenylethyl)-5,6-ene-2-piperidone (**8j**)

This byproduct was obtained during the synthesis of **6j** in a yield up to 23% depending on the experiment. ^1^H NMR (CDCl_3_): δ 2.21–2.32 (m, 2H, CH_2_), 2.39–2.52 (m, 2H, CH_2_), 2.56 (dd, *J* = 8.4, 6.6 Hz, 2H, CH_2_), 3.55 (t, *J* = 7.5 Hz, 2H, CH_2_N), 4.05 (s, 4H, C_5_H_4_), 4.11 (s, 5H, Cp), 5.05 (dt, *J* = 7.6, 4.4 Hz, 1H, =CH), 5.86 (dt, *J* = 7.6, 1.6 Hz, 1H, =CH). ^13^C NMR (CDCl_3_): δ 20.3 (CH_2_), 28.7 (CH_2_), 31.4 (CH_2_), 47.8 (CH_2_N), 67.6 (2CH C_5_H_4_), 68.4 (2CH C_5_H_4_), 68.7 (5CH Cp), 85.1 (C C_5_H_4_), 105.9 (-CH=), 130.3 (N-CH=), 169.4 (CO). IR (ATR, ν cm^−1^): 1622, 1606. HRMS (ESI, C_17_H_20_FeNO: [M + H]^+^) calcd: 310.0889, found: 310.0897.

#### 3.6.13. *N*-(2-ferrocenylethyl)-3-methoxycarbonylbutanamide (**9j**)

This compound is an impurity that was identified during the synthesis of **6j** and was not fully characterized. ^1^H NMR (CDCl_3_): δ 1.85–2.04 (m, 2H, CH_2_), 2.18 (t, *J* = 7.3 Hz, 2H, CH_2_), 2.35 (t, *J* = 7.1 Hz, 2H, CH_2_), 2.49 (t, *J* = 7.0 Hz, 2H, CH_2_), 3.33 (q, *J* = 6.6 Hz, 2H, CH_2_N), 3.65 (s, 3H, OMe), 3.98–4.21 (m, 9H, C_5_H_4_-Fe-Cp), 5.75 (s broad, 1H, NH). ^13^C NMR (CDCl_3_): δ 20.9 (CH_2_), 29.7 (CH_2_), 33.2 (CH_2_), 35.6 (CH_2_), 40.6 (CH_2_), 51.7 (OMe), 68.0 (2CH C_5_H_4_), 68.6 (2CH C_5_H_4_), 69.0 (5CH Cp), 85.9 (C C_5_H_4_), 172.0 (CO), 173.7 (CO). IR (ATR, ν cm^−1^): 1728, 1653 (CO). 

### 3.7. General Procedure for the Synthesis of α-Methoxylactams ***10***

In a flask, α-hydroxylactams **6** were dissolved into a minimum of THF. Methanol (10 mL/mmol of **6**) and a spatula tip of TsOH were added. The mixture was stirred and monitored by TLC until substrate disappearance, then a solution of sodium hydrogen carbonate was added. The mixture was poured into water and extracted twice with dichloromethane. The combined organic layer was washed with water and dried with magnesium sulfate. The solution was concentrated under reduced pressure and the residue was chromatographed on silica gel with DCM/petroleum ether mixture (4:1), affording the α-methoxylactams **10** as orange-yellow solids.

#### 3.7.1. 1-(2-Ferrocenylethyl)-5-methoxy-2-pyrrolidone (**10b**)

From **6b**, yield of 89%. ^1^H NMR (CDCl_3_): δ 1.84–2.16 (m, 2H, -CO-CH_2_-C*H_2_*-CH-O), 2.19–2.36 (m, 1H, -CO-C*H_2_*-CH_2_-CH-O), 2.38–2.55 (m, 1H, -CO-C*H_2_*-CH_2_-CH-O), 2.50–2.71 (m, 2H, >N-CH_2_-C*H_2_*-Fc), 3.09–3.26 (m, 4H, >N-C*H_2_*-CH_2_-Fc + OMe), 3.57–3.74 (m, 1H, >N-C*H_2_*-CH_2_-C=), 3.98–4.16 (m, 9H, CpFeC_5_H_4_), 4.73 (dd, *J* = 6.3 and 1.6 Hz, 1H, >N-CH-O-). ^13^C NMR (CDCl_3_): δ 23.9 (-CO-CH_2_-*CH_2_*-CH-O), 27.9 (>N-CH_2_-*CH_2_*-Fc), 29.1 (-CO-*CH_2_*-CH_2_-CH-O), 41.9 (>N-*CH_2_*-CH_2_-Fc), 52.8 (OMe), 67.5 (CH C_5_H_4_), 67.6 (CH C_5_H_4_), 68.0 (CH C_5_H_4_), 68.4 (CH C_5_H_4_), 68.6 (5CH Cp), 85.5 (C C_5_H_4_), 90.5 (>N-CH-O-), 174.9 (CO). IR (ATR, ν cm^−1^): 1683 (CO). HRMS (ESI, C_17_H_21_FeNNaO_2_: [M + Na]^+^) calcd: 350.081388, found: 350.0809.

#### 3.7.2. 2,3-Dihydro-3-methoxy-2-(ferrocenylmethyl)-1*H*-isoindol-1-one (**10e**)

From **6e**, yield of 90%. ^1^H NMR (CDCl_3_): δ 2.86 (s, 3H, OMe), 3.99 (d, *J* = 13.4 Hz, 1H, CH_2_N), 4.05–4.52 (m, 9H, CpFeC_5_H_4_), 4.78 (d, *J* = 13.4 Hz, 1H, CH_2_N), 5.78 (s, 1H, >N-CH-O-), 7.37–7.62 (m, 3H, isoindole), 7.79 (d, *J* = 7.1 Hz, 1H, isoindole). ^13^C NMR (CDCl_3_): δ 38.8 (CH_2_N), 49.2 (OMe), 68.9 (CH C_5_H_4_), 69.4 (CH C_5_H_4_), 69.5 (5CH Cp), 70.1 (CH C_5_H_4_), 70.3 (CH C_5_H_4_), 83.4 (C C_5_H_4_), 85.6 (>N-CH-O-), 123.5 (CH isoindole), 123.6 (CH isoindole), 129.9 (CH isoindole), 132.0 (CH isoindole), 133.2 (C isoindole), 140.4 (C isoindole), 167.0 (CO). IR (ATR, ν cm^−1^): 1699 (CO). HRMS (ESI, C_20_H_19_FeNO_2_: [M]^+^) calcd: 361.0760, found: 361.0756.

#### 3.7.3. 2,3-Dihydro-3-methoxy-2-(2-ferrocenylethyl)-1*H*-isoindol-1-one (**10f**)

From **6f**, yield of 100%. ^1^H NMR (CDCl_3_): δ 2.55–2.80 (m, 2H, C*H_2_*-Fc), 2.85 (s, 3H, OMe), 3.25–3.42 (m, 1H, CH_2_N), 3.88–4.02 (m, 1H, CH_2_N), 4.16–4.24 (m, 9H, CpFeC_5_H_4_), 5.72 (s, 1H, >N-CH-O-), 7.44–7.62 (m, 3H, isoindole), 7.82 (d, *J* = 7.2 Hz, 1H, isoindole). ^13^C NMR (CDCl_3_): δ 28.2 (*CH_2_*-Fc), 40.7 (CH_2_N), 49.3 (OMe), 67.7 (CH C_5_H_4_), 67.8 (CH C_5_H_4_), 68.2 (CH C_5_H_4_), 68.5 (CH C_5_H_4_), 68.8 (5CH Cp), 85.5 (C C_5_H_4_), 86.6 (>N-CH-O-), 123.4 (2CH isoindole), 130.0 (CH isoindole), 132.0 (CH isoindole), 133.2 (C isoindole), 140.4 (C isoindole), 167.6 (CO). IR (ATR, ν cm^−1^): 1706 (CO). HRMS (ESI, C_21_H_22_FeNO_2_: [M + H]^+^) calcd: 376.0994, found: 376.0995.

#### 3.7.4. 2,3-Dihydro-3-methoxy-2-(3-ferrocenylpropyl)-1*H*-isoindol-1-one (**10g**)

From **6g**, yield of 94%. ^1^H NMR (CDCl_3_): δ 1.78–2.00 (m, 2H, >N-CH_2_-C*H_2_*-CH_2_-Fc), 2.31–2.48 (m, 2H, -C*H_2_*-Fc), 2.87 (s, 3H, OMe), 3.20–3.36 (m, 1H, CH_2_N), 3.71–3.90 (m, 1H, CH_2_N), 3.96–4.16 (m, 9H, CpFeC_5_H_4_), 5.87 (s, 1H, >N-CH-O-), 7.45–7.64 (m, 3H, isoindole), 7.78–7.89 (m, 1H, isoindole). ^13^C NMR (CDCl_3_): δ 27.1 (CH_2_), 29.6 (*CH_2_*-Fc), 39.5 (CH_2_N), 49.2 (OMe), 67.3 (2CH C_5_H_4_), 68.0 (CH C_5_H_4_), 68.1 (CH C_5_H_4_), 68.6 (5CH Cp), 86.3 (>N-CH-O-), 88.2 (C C_5_H_4_), 123.5 (2CH isoindole), 130.0 (CH isoindole), 132.0 (CH isoindole), 133.2 (C isoindole), 140.3 (C isoindole), 167.7 (CO). IR (ATR, ν cm^−1^): 1702 (CO). HRMS (ESI, C_22_H_23_FeNNaO_2_: [M + Na]^+^) calcd: 412.097038, found: 412.0971.

#### 3.7.5. 2,3-Dihydro-3-methoxy-2-(4-ferrocenylbutyl)-1*H*-isoindol-1-one (**10h**)

From **6h**, yield of 79%. ^1^H NMR (CDCl_3_): δ 1.48–1.78 (m, 2H, >N-CH_2_-C*H_2_*-C*H_2_*-CH_2_-Fc), 2.38 (t, *J* = 7.5 Hz, 2H, -C*H_2_*-Fc), 2.87 (s, 3H, OMe), 3.15–3.31 (m, 1H, CH_2_N), 3.72–3.88 (m, 1H, CH_2_N), 3.98–4.14 (m, 9H, CpFeC_5_H_4_), 5.86 (s, 1H, >N-CH-O-), 7.47–7.62 (m, 3H, isoindole), 7.83 (dd, *J* = 8.1 and 1.2 Hz, 1H, isoindole). ^13^C NMR (CDCl_3_): δ 28.0 (CH_2_), 28.7 (CH_2_), 29.4 (*CH_2_*-Fc), 39.4 (CH_2_N), 49.2 (OMe), 67.3 (2CH C_5_H_4_), 68.3 (2CH C_5_H_4_), 68.7 (5CH Cp), 86.3 (>N-CH-O-), 89.0 (C C_5_H_4_), 123.5 (CH isoindole), 123.6 (CH isoindole), 130.1 (CH isoindole), 132.0 (CH isoindole), 133.3 (C isoindole), 140.4 (C isoindole), 167.8 (CO). MS (EI, 70 eV) *m*/*z*: 403 [M]^+^, 338, 306, 199, 186, 121 [CpFe]^+^. IR (KBr, ν cm^−1^): 1705 (CO). HRMS (ESI, C_23_H_25_FeNO_2_: [M]^+^) calcd: 403.1235, found: 403.1223.

### 3.8. 2,3-Dihydro-3-carboxymethylthio-2-(ferrocenylmethyl)-1H-isoindol-1-one (***11***)

In a flask, α-hydroxylactam **6e** was dissolved into a minimum of THF. Dichloromethane (50 mL), thioglycolic acid (0.122 g, 1.3 mmol), and a spatula tip of TsOH were added. The solution was stirred and refluxed for 8 h and monitored by TLC. After cooling, the solution was poured into a solution of sodium hydrogen carbonate and extracted twice with dichloromethane. The combined organic layer was washed with water and dried with magnesium sulfate. The solvent was evaporated under reduced pressure and the residue was dissolved in acetone and concentrated to a minimum volume under reduced pressure. The solution was left overnight in a freezer and the crystals were filtered off, affording compound **11** as an orange-red solid. Yield 76%. ^1^H NMR (DMSO-d_6_): δ 2.57 (d, *J* = 15.4 Hz, 1H, SCH_2_COO), 2.74 (d, *J* = 15.4 Hz, 1H, SCH_2_COO), 4.11 (s, 1H, C_5_H_4_), 4.14 (s, 1H, C_5_H_4_), 4.17 (d, *J* = 14.8 Hz, 1H, NCH_2_), 4.22 (s, 6H, Cp + C_5_H_4_), 4.32 (s, 1H, C_5_H_4_), 4.78 (d, *J* = 14.8 Hz, 1H, NCH_2_), 5.70 (s, 1H, N-CH-S), 7.52 (td, *J* = 7.2, 1.4 Hz, 1H, isoindole), 7.56–7.72 (m, 3H, isoindole), 12.55 (s broad, 1H, OH). ^13^C NMR (CDCl_3_): δ 28.8 (CH_2_), 39.0 (CH_2_), 63.0 (-S-CH-N<), 68.8 (CH C_5_H_4_), 69.5 (6CH Cp + CH C_5_H_4_), 69.8 (CH C_5_H_4_), 70.1 (CH C_5_H_4_), 82.9 (C C_5_H_4_), 123.7 (CH isoindole), 124.0 (CH isoindole), 129.6 (CH isoindole), 132.1 (C isoindole), 132.6 (CH isoindole), 141.9 (C isoindole), 173.7 (CO), 176.1 (CO). IR (ATR, ν cm^−1^): 1732 (CO), 1659 (CO). HRMS (ESI, C_21_H_18_FeNO_3_S: [M-H]^−^) calcd: 420.03568, found: 420.0366.

### 3.9. 2,3-Dihydro-3-amino-2-(ferrocenylmethyl)-1H-isoindol-1-one (***13***)

A solution of ammonia in DCM was prepared by the extraction of 400 mL of concentrated aqueous ammonia solution with 400 mL DCM. The aqueous layer was kept in the funnel for later use and the solution of ammonia in DCM was dried over calcium chloride and filtered. Hydroxylactam **6e** (4.58 g, 13.19 mmol) and oxalyl chloride (1.727 g, 1.19 mL, 13.6 mmol) were stirred in dry DCM until the solids disappeared, then the reaction was continued for 30 min to form compound **12**. This solution was poured into the previous solution of ammonia in DCM and the mixture was stirred for 10 min. The solution was transferred into the funnel containing aqueous ammonia, then the mixture was made more basic with the addition of sodium hydroxide solution. The organic layer was decanted then conc. HCl was added until the yellow precipitate stopped to appear. The precipitate was poured into a solution of NaOH 10 wt% and the compound was extracted twice with DCM. The combined organic layer was concentrated under reduced pressure to afford pure **13** in 59% yield. Mp: 218 °C. ^1^H NMR (DMSO-d_6_): δ 2.65 (s broad, 2H, NH_2_), 4.08 (s, 2H, C_5_H_4_), 4.10 (s, 2H, C_5_H_4_), 4.18 (d, *J* = 14.5 Hz, 1H, CH_2_), 4.18–4.26 (m, 6H, Cp + C_5_H_4_), 4.40(s, 1H, C_5_H_4_), 4.67 (d, *J* = 14.5 Hz, 1H, CH_2_), 5.08 (s broad, 1H, N-CH-N), 7.41–7.52 (m, 1H, isoindole), 7.53–7.65 (m, 3H, isoindole). ^13^C NMR (DMSO-d_6_): δ 37.0 (CH_2_), 67.5 (N-CH-N), 67.9 (CH C_5_H_4_), 68.4 (5CH Cp), 68.6 (2CH C_5_H_4_), 69.0 (CH C_5_H_4_), 84.1 (C C_5_H_4_), 122.1 (CH isoindole), 123.6 (CH isoindole), 128.6 (CH isoindole), 131.5 (CH isoindole), 131.8 (C isoindole), 146.5 (C isoindole), 165.3 (CO). IR (ATR, ν cm^−1^): 1667 (CO). MS (CI, NH_3_) *m*/*z*: 347 [M + H]^+^, 330 [M − NH_3_ + H]^+^, 199 [FcCH_2_]^+^. HRMS (ESI, C_19_H_18_FeN_2_O: [M]^+^) calcd: 346.0763, found: 346.0763.

### 3.10. General Procedure for the Synthesis of Fused Ferrocenyl Lactams ***14***, ***15*** and ***16***

In a flask containing dichloroethane (DCE, 10 mL/mmol of α-hydroxylactam **6** or α-methoxylactam **10**), TsOH (spatula tip) was added and the solution was heated at 70 °C. α-hydroxylactam **6** or α-methoxylactam **10** was rapidly added and the reaction was monitored by TLC until disappearance of substrate. After 15 min, the solution was cooled and was poured into a sodium hydrogen carbonate solution and extracted twice with dichloromethane. The combined organic layer was washed with water and dried with magnesium sulfate. The solution was concentrated under reduced pressure and the residue was chromatographed on silica gel with DCM/petroleum ether 2:1 mixture, affording the fused lactams **14**–**16** as orange-yellow solids. 

#### 3.10.1. Ferroceno[1,2-a]-3,10b-dihydropyrido[2,1-a]isoindol-6(4*H*)-one (**14**)

From **6f**, yield of 84%; from **10f**, yield of 83%. This compound has been reported in the literature using another pathway, but NMR signals were not attributed [26]. ^1^H NMR (CDCl_3_): δ 2.48 (dd, *J* = 15.4, 5.3 Hz, 1H, CH_2_), 3.24–2.89 (m, 2H, CH_2_), 3.45 (s, 5H, Cp), 4.08 (t, *J* = 2.4 Hz, 1H, C_5_H_3_), 4.20 (s broad, 1H, C_5_H_3_), 4.22 (s broad, 1H, C_5_H_3_), 4.62 (dd, *J* = 12.4, 6.0 Hz, 1H, CH_2_), 5.25 (s, 1H, CH-N), 7.50–7.61 (m, 1H, isoindole), 7.62–7.72 (m, 2H, isoindole), 7.99 (d, *J* = 7.5 Hz, 1H, isoindole). ^13^C NMR (CDCl_3_): δ 24.4 (CH_2_), 37.0 (CH_2_), 57.4 (CH), 63.1 (CH C_5_H_3_), 66.0 (CH C_5_H_3_), 66.5 (CH C_5_H_3_), 69.5 (5CH Cp), 81.7 (C C_5_H_3_), 85.6 (C C_5_H_3_), 122.5 (CH isoindole), 123.9 (CH isoindole), 128.5 (CH isoindole), 131.4 (CH isoindole), 132.7 (C isoindole), 146.6 (C isoindole), 167.3 (CO). IR (ATR, ν cm^−1^): 1680 (CO). MS (ESI) *m*/*z*: 343 [M]^+^, 366 [M + Na]^+^, 709 [M_2_ + Na]^+^. HRMS (ESI, C_20_H_17_FeNNaO: [M + Na]^+^) calcd: 366.055173, found: 366.05509.

#### 3.10.2. Ferroceno[1,2-h]-1,2,3,5,6,8a-hexahydroindolizin-3-one (**15**)

From **6b**. Yield of 84%. ^1^H NMR (CDCl_3_): δ 2.23–3.02 (m, 7H, 4CH_2_), 3.94 (s, 1H, C_5_H_3_), 4.06 (s, 1H, C_5_H_3_), 4.08 (s, 5H, Cp), 4.21 (s, 2H, CH_2_N + C_5_H_3_), 4.32 (t, *J* = 7.2 Hz, 1H, CH-N). ^13^C NMR (CDCl_3_): δ 23.3 (CH_2_), 26.0 (CH_2_), 31.5 (CH_2_), 37.5 (CH_2_), 54.7 (CH), 62.2 (CH C_5_H_3_), 65.8 (CH C_5_H_3_), 65.9 (CH C_5_H_3_), 69.5 (5CH Cp), 81.6 (C C_5_H_3_), 91.2 (C C_5_H_3_), 173.4 (CO). IR (ATR, ν cm^−1^): 1673 (CO). HRMS (ESI, C_16_H_18_FeNO: [M + H]^+^) calcd: 296.0732, found: 296.0731. Crystal data: C_16_H_17_FeNO, monoclinic P 2_1_/c, a = 14.6466(7) Å, b = 7.1923(3) Å, c = 12.0545(6) Å, α = γ = 90°, β = 94.662(4)°, V = 1265.65(10) Å^3^, Z = 4, yellow prism 0.37 × 0.27 × 0.06 mm^3^, μ = 9.458 mm^−1^, min/max transmission = 0.17/0.71, T = 200 (1) K, λ = 1.54178 Å, θ range = 6.06° to 66.57°, 9310 reflections measured, 2242 independent, R_int_ = 0.0471, completeness = 0.999, 173 parameters, 0 restraints, final R indices R1 [I > 2σ (I)] = 0.0350 and wR2 (all data) = 0.0904, GOF on F^2^ = 1.025, largest difference peak/hole = 0.25/−0.29 e·Å^−3^.

#### 3.10.3. Ferroceno[8,9-a]octahydro-4H-quinolizin-4-one (**16**)

From the crude mixture of reduction of glutarimide **3j**. Overall yield of 74% starting from glutarimide **3j**. ^1^H NMR (CDCl_3_): δ 1.57–3.02 (m, 9H, 5CH_2_), 3.91 (s, 1H, C_5_H_3_), 3.99 (s, 1H, C_5_H_3_), 4.04 (s, 6H, Cp + CH − N), 4.16 (s, 1H, C_5_H_3_), 4.80 (s broad, 1H, CH_2_). ^13^C NMR (CDCl_3_): δ 19.5 (CH_2_), 23.9 (CH_2_), 30.4 (CH_2_), 33.2 (CH_2_), 40.3 (CH_2_), 54.4 (CH), 62.6 (CH C_5_H_3_), 65.4 (CH C_5_H_3_), 65.6 (CH C_5_H_3_), 69.5 (5CH Cp), 81.2 (C C_5_H_3_), 90.7 (C C_5_H_3_), 169.8 (CO). IR (ATR, ν cm^−1^): 1638 (CO). HRMS (ESI, C_17_H_20_FeNO: [M + H]^+^) calcd: 310.0889, found: 310.0897.

### 3.11. 2,3-Dihydro-3-carboxyethyl-2-(ferrocenylmethyl)-1H-isoindol-1-one (***17***)

A solution of (carbethoxymethylene) triphenylphosphorane (2.926 g, 8.4 mmol), **6e** (2.43 g, 7 mmol) in toluene (30 mL) was stirred under reflux overnight. The solution was concentrated under reduced pressure then potassium carbonate (4.257 g, 30.8 mmol), water (5 mL), and ethanol (20 mL) were added and the mixture was stirred under reflux for 3 h. The mixture was concentrated under reduced pressure then water (200 mL) and DCM (150 mL) were added, and after shaking, the organic layer was discarded. The aqueous layer was washed with DCM and then carefully acidified with hydrochloric acid. Compound **27** was extracted twice with DCM then the combined organic layer was concentrated under reduced pressure. The residue was dissolved into acetone and the solution was partially concentrated under reduced pressure and was left overnight in a freezer. The crystals were filtered off to furnish **17** as red-orange crystals. Yield of 90%.

Mp: 194 °C. ^1^H NMR (DMSO-d_6_): δ 2.65 (dd, *J* = 16.2 and 7.1 Hz, 1H, CH_2_CO), 3.04 (dd, *J* = 16.2 and 4.6 Hz, 1H, CH_2_CO), 4.04–4.15 (m, 3H, 2CH C_5_H_4_ + CH_2_N), 4.15–4.26 (m, 6H, 5CH Cp + CH C_5_H_4_), 4.34–4.42 (m, 1H, CH C_5_H_4_), 4.71 (dd, *J* = 7.1 and 4.6 Hz, 1H, CH), 4.84 (d, *J* = 18.8 Hz, 1H, CH_2_N), 7.43–7.49 (m, 1H, isoindole), 7.49–7.61 (m, 2H, isoindole), 7.65 (d, *J* = 7.4 Hz, 1H, isoindole). ^13^C NMR (DMSO-d_6_): δ 36.5 (CH_2_), 38.8 (CH_2_), 55.3 (CH), 67.7 (CH C_5_H_4_), 68.1 (CH C_5_H_4_), 68.5 (CH C_5_H_4_ + 5CH Cp), 69.1 (CH C_5_H_4_), 83.5 (C C_5_H_4_), 122.8 (CH isoindole), 122.9 (CH isoindole), 128.3 (CH isoindole), 131.5 (C isoindole), 131.6 (CH isoindole), 145.2 (C isoindole), 166.6 (CO), 171.7 (CO). IR (ATR, ν cm^−1^): 1715 (CO), 1640 (CO). MS (CI, NH_3_) *m*/*z*: 390 [M + H]^+^, 199 [FcCH_2_]^+^, 407 [M + NH_4_]^+^. Anal. Calcd for C_21_H_19_FeNO_3_: C, 64.8; H, 4.92; N, 3.59. Found: C, 64.87; H, 4.91; N, 3.41.

### 3.12. General Procedure for the Synthesis of Ferrocidiphenols ***20***–***27***

In a flask, α-hydroxylactam **18** was dissolved into a minimum of THF, and DCM was added. The alcohol or thiol and a spatula tip of TsOH were added. The solution was stirred at room temperature and the reaction was monitored by TLC. When the reaction was complete, the solution was poured into a solution of sodium hydrogen carbonate and extracted twice with DCM. The combined organic layer was washed with water and dried with magnesium sulfate. The solution was concentrated under reduced pressure and the residue was chromatographed on silica gel or precipitated, affording the pure compounds **20–27** as orange-yellow solids.

#### 3.12.1. 2,3-Dihydro-3-(2-hydroxyethyl)-2-[4-ferrocenyl-5,5-bis-(4-hydroxyphenyl)-pent-4-enyl]-1*H*-isoindol-1-one (**20**)

From compound **18** (0.25 g, 0.427 mmol) and ethylene glycol (0.265 g, 4.3 mmol). Precipitation and filtration gave compound **20** in a yield of 75%. ^1^H NMR (DMSO-d_6_): δ 1.60–1.91 (m, 2H, CH_2_), 2.42–2.58 (m, 2H, CH_2_-C=C), 2.94–3.05 (m, 1H, CH_2_-O), 3.05–3.19 (m, 2H, CH_2_N + CH_2_-O), 3.38–3.51 (m, 2H, CH_2_-O), 3.56–3.69 (m, 1H, CH_2_N), 3.72 (s broad, 1H, C_5_H_4_), 3.95 (s broad, 1H, C_5_H_4_), 4.00–4.17 (m, 7H, Cp + 2C_5_H_4_), 5.51 (s, 1H, CH-O), 6.55–6.69 (m, 4H, C_6_H_4_), 6.77 (d, *J* = 8.0 Hz, 2H, C_6_H_4_), 6.94 (d, *J* = 8.0 Hz, 2H, C_6_H_4_), 7.49–7.75 (m, 4H, isoindole), 9.23 (s broad, 2H, OH). ^13^C NMR (DMSO-d_6_): δ 28.5 (CH_2_), 31.7 (CH_2_), 38.7 (CH_2_), 60.0 (CH_2_-O), 65.1 (CH_2_-O), 67.7 (CH C_5_H_4_), 67.8 (CH C_5_H_4_), 68.6 (CH C_5_H_4_), 68.7 (CH C_5_H_4_), 68.9 (5CH, Cp), 85.1 (CH-OH), 86.9 (C C_5_H_4_), 114.99 (2CH C_6_H_4_), 115.04 (2CH C_6_H_4_), 122.5 (CH isoindole), 123.8 (CH isoindole), 129.8 (CH isoindole), 129.9 (2CH C_6_H_4_), 130.4 (2CH C_6_H_4_), 131.99 (CH isoindole), 132.03 (C), 132.9 (C), 135.0 (C), 135.5 (C), 138.2 (C), 141.3 (C), 155.66 (C), 156.70 (C), 166.4 (CO). IR (ATR, ν cm^−1^): 1649 (CO). HRMS (ESI, C_37_H_35_FeNO_5_: [M]^+.^) calcd: 629.1859, found: 629.1864.

#### 3.12.2. 2,3-Dihydro-3-(6-hydroxyhexyl)-2-[4-ferrocenyl-5,5-bis-(4-hydroxyphenyl)-pent-4-enyl]-1*H*-isoindol-1-one (**21**)

From compound **18** (0.2 g, 0.342 mmol) and 1,6-hexanediol (0.404 g, 3.4 mmol). Eluent: DCM; yield of 65%. ^1^H NMR (acetone-d_6_): δ 1.24–1.40 (m, 4H, CH_2_), 1.41–1.59 (m, 4H, CH_2_), 1.74–1.98 (m, 2H, CH_2_), 2.54–2.82 (m, 2H, CH_2_-C=C), 2.84–3.08 (m, 1H, CH_2_-O), 3.08–3.26 (m, 2H, CH_2_N + CH_2_-O), 3.45–3.59 (m, 3H, CH_2_-O + OH), 3.68–3.85 (m, 1H, CH_2_N), 3.79–3.85 (m, 1H, C_5_H_4_), 3.97–4.04 (m, 1H, C_5_H_4_), 4.04–4.15 (m, 7H, Cp + 2C_5_H_4_), 5.45 (s, 1H, CH-O), 6.69 (d, *J* = 8.4 Hz, 2H, C_6_H_4_), 6.73 (d, *J* = 8.4 Hz, 2H, C_6_H_4_), 6.86 (d, *J* = 8.4 Hz, 2H, C_6_H_4_), 7.03 (d, *J* = 8.4 Hz, 2H, C_6_H_4_), 7.50–7.68 (m, 3H, isoindole), 7.71 (d, *J* = 7.3 Hz, 1H, isoindole), 8.24 (s, 1H, OH), 8.31 (s, 1H, OH). ^13^C NMR (acetone-d_6_): δ 26.4 (CH_2_), 26.7 (CH_2_), 29.6 (CH_2_), 30.4 (CH_2_), 33.1 (CH_2_), 33.6 (CH_2_), 39.8 (CH_2_), 62.4 (CH_2_-O), 64.1 (CH_2_-O), 68.6 (CH C_5_H_4_), 68.7 (CH C_5_H_4_), 69.9 (5CH Cp + C_5_H_4_), 70.1 (CH C_5_H_4_), 86.3 (CH-O), 88.5 (C C_5_H_4_), 115.7 (2CH C_6_H_4_), 116.0 (2CH C_6_H_4_), 123.5 (CH isoindole), 124.6 (CH isoindole), 130.4 (CH isoindole), 131.3 (2CH C_6_H_4_), 131.8 (2CH C_6_H_4_), 132.7 (CH isoindole), 133.6 (C), 134.5 (C), 136.8 (C), 137.3 (C), 139.4 (C), 142.8 (C), 156.7 (C), 156.8 (C), 167.6 (CO). IR (ATR, ν cm^−1^): 1678 (CO). HRMS (ESI, C_41_H_43_FeNO_5_: [M]^+^) calcd: 685.2486, found: 685.2506.

#### 3.12.3. O-{2,3-Dihydro-2-[4-ferrocenyl-5,5-bis-(4-hydroxyphenyl)-pent-4-enyl]-1H-isoindol-1-one-3-yl}-12-hydroxy-1,4,7,10-tetraoxadodecane (**22**)

From compound **18** (0.25 g, 0.427 mmol) and tetraethylene glycol (0.829 g, 4.3 mmol). Eluent: cyclohexane/ethyl acetate 1: 2; yield of 79%. ^1^H NMR (acetone-d_6_): δ 1.74–1.96 (m, 2H, CH_2_), 2.55–2.80 (m, 2H, CH_2_-C=C), 3.12–3.25 (m, 2H, CH_2_N + CH_2_-O), 3.26–3.37 (m, 1H, CH_2_-O), 3.48–3.68 (m, 14H, CH_2_-O), 3.68–3.80 (m, 1H, CH_2_N), 3.80–3.85 (m, 1H, C_5_H_4_), 3.99–4.04 (m, 1H, C_5_H_4_), 4.04–4.14 (m, 7H, Cp + 2C_5_H_4_), 5.49 (s, 1H, CH-O), 6.69 (d, *J* = 8.5 Hz, 2H, C_6_H_4_), 6.72 (d, *J* = 8.5 Hz, 2H, C_6_H_4_), 6.86 (d, *J* = 8.5 Hz, 2H, C_6_H_4_), 7.03 (d, *J* = 8.5 Hz, 2H, C_6_H_4_), 7.51–7.61 (m, 1H, isoindole), 7.61–7.67 (m, 2H, isoindole), 7.71 (d, *J* = 7.3 Hz, 1H, isoindole), 8.17 (s, 1H, OH), 8.20 (s, 1H, OH). ^13^C NMR (acetone-d_6_): δ 29.6 (CH_2_), 33.1 (CH_2_), 39.7 (CH_2_), 62.0 (CH_2_-O), 64.1 (CH_2_-O), 68.6 (CH C_5_H_4_), 68.7 (CH C_5_H_4_), 69.8 (5 CH Cp + 1 CH C_5_H_4_), 70.1 (CH C_5_H_4_), 70.9 (CH_2_-O), 71.1 (CH_2_-O), 71.2 (2CH_2_-O), 71.3 (CH_2_-O), 73.5 (CH_2_-O), 86.5 (CH-OH), 88.5 (C C_5_H_4_), 115.7 (2CH C_6_H_4_), 116.0 (2CH C_6_H_4_), 123.5 (CH isoindole), 124.8 (CH isoindole), 130.5 (CH isoindole), 131.3 (2CH C_6_H_4_), 131.8 (2CH C_6_H_4_), 132.7 (CH isoindole), 133.6 (C), 134.6 (C), 136.8 (C), 137.2 (C), 139.4 (C), 142.6 (C), 156.66 (C), 156.72 (C), 167.7 (CO). IR (ATR, ν cm^−1^): 1679 (CO). HRMS (ESI, C_43_H_47_FeNNaO_8_: [M + Na]^+^) calcd: 784.254328, found: 784.2544.

#### 3.12.4. 2,3-Dihydro-3-(pent-4-ynyl)-2-[4-ferrocenyl-5,5-bis-(4-hydroxyphenyl)-pent-4-enyl]-1*H*-isoindol-1-one (**23**)

From compound **18** (0.25 g, 0.427 mmol) and 4-pentyn-1-ol (0.359 g, 4.3 mmol). Eluent: cyclohexane/ethyl acetate 2: 1. Yield of 91%. ^1^H NMR (acetone-d_6_): δ 1.61–1.76 (m, 2H, CH_2_), 1.77–1.96 (m, 2H, CH_2_), 2.19–2.32 (m, 3H, -CH_2_-alkyn-H), 2.54–2.82 (m, 2H, CH_2_-C=C), 3.04–3.22 (m, 2H, CH_2_N + CH_2_O), 3.23–3.36 (m, 1H, CH_2_O), 3.70–3.84 (m, 1H, CH_2_N), 3.81 (s, 1H, C_5_H_4_), 4.01 (s, 1H, C_5_H_4_), 4.09 (s, 7H, Cp + C_5_H_4_), 5.45 (s, 1H, N-CH-O), 6.69 (d, *J* = 8.5 Hz, 2H, C_6_H_4_), 6.73 (d, *J* = 8.5 Hz, 1H, C_6_H_4_), 6.86 (d, *J* = 8.5 Hz, 1H, C_6_H_4_), 7.03 (d, *J* = 8.5 Hz, 1H, C_6_H_4_), 7.51–7.68 (m, 3H, isoindole), 7.72 (dt, *J* = 7.3, 1.1 Hz, 1H, isoindole), 8.18 (s, 1H, OH), 8.22 (s, 1H, OH). ^13^C NMR (acetone-d_6_): δ 15.3 (CH_2_), 29.2 (CH_2_), 29.4 (CH_2_), 32.9 (CH_2_), 39.5 (CH_2_), 62.0 (CH_2_), 68.6 (CH C_5_H_4_), 68.7 (CH C_5_H_4_), 69.9 (CH C_5_H_4_ + 5CH Cp), 70.1 (CH C_5_H_4_), 70.3 (C alkyne), 83.9 (CH alkyne), 86.2 (N-CH-O), 88.5 (C C_5_H_4_), 115.7 (2CH C_6_H_4_), 116.0 (2CH C_6_H_4_), 123.5 (CH isoindole), 124.6 (CH isoindole), 130.5 (CH isoindole), 131.3 (2CH C_6_H_4_), 131.8 (2CH C_6_H_4_), 132.7 (CH isoindole), 133.6 (C), 134.6 (C), 136.9 (C), 137.3 (C), 139.4 (C), 142.6 (C), 156.67 (C), 156.73 (C), 167.6 (CO). IR (ATR, ν cm^−1^): 2118 (alkyne), 1670 (CO). HRMS (ESI, C_40_H_38_FeNO_4_: [M + H]^+^) calcd: 652.2145, found: 652.2143. Crystal data: C_41.5_H_40_FeNO_4.5_, monoclinic P 2_1_/n, a = 9.8118(3) Å, b = 17.2893(6) Å, c = 20.2760(6) Å, α = γ = 90°, β = 91.557(2)°, V = 3438.33(19) Å^3^, Z = 4, orange prism 0.2 × 0.1 × 0.05 mm^3^, μ = 3.874 mm^−1^, min/max transmission = 0.65/0.90, T= 200(1) K, λ = 1.54178 Å, θ range = 4.37° to 66.47°, 26640 reflections measured, 6066 independent, R_int_ = 0.0505, completeness = 0.998, 455 parameters, 27 restraints, final R indices R1 [I > 2σ (I)] = 0.0382 and wR2 (all data) = 0.0982, GOF on F^2^ = 1.031, largest difference peak/hole = 0.27/−0.34 e·Å^−3^.

#### 3.12.5. O-{2,3-Dihydro-2-[4-ferrocenyl-5,5-bis-(4-hydroxyphenyl)-pent-4-enyl]-1*H*-isoindol-1-one-3-yl}-24-azido-1,4,7,10,13,16,19,22-octaoxatetracosane (**24**)

From compound **18** (0.22 g, 0.376 mmol) and O-(2-azidoethyl)heptaethylene glycol (0.223 g, 0.564 mmol). Eluent: cyclohexane/ethyl acetate 1: 2. Yield of 28%. ^1^H NMR (acetone-d_6_): δ 1.76–1.95 (m, 2H, CH_2_), 2.53–2.78 (m, 2H, CH_2_-C=C), 2.82–3.01 (m, 1H, CH_2_N), 3.12–3.43 (m, 4H, CH_2_-O + CH_2_-N_3_), 3.48–3.72 (m, 28H, CH_2_-O), 3.68–3.81 (m, 1H, CH_2_N), 3.80–3.85 (m, 1H, C_5_H_4_), 3.98–4.04 (m, 1H, C_5_H_4_), 4.04–4.15 (m, 7H, Cp + 2C_5_H_4_), 5.49 (s, 1H, CH-O), 6.69 (d, *J* = 8.4 Hz, 2H, C_6_H_4_), 6.73 (d, *J* = 8.4 Hz, 2H, C_6_H_4_), 6.86 (d, *J* = 8.4 Hz, 2H, C_6_H_4_), 7.03 (d, *J* = 8.4 Hz, 2H, C_6_H_4_), 7.51–7.61 (m, 1H, isoindole), 7.61–7.68 (m, 2H, isoindole), 7.71 (d, *J* = 7.3 Hz, 1H, isoindole), 8.20 (s, 2H, OH). ^13^C NMR (CDCl_3_): δ 28.7 (CH_2_), 32.1 (CH_2_), 39.3 (CH_2_), 50.8 (CH_2_-N_3_), 63.1 (CH_2_-O), 68.2 (4CH C_5_H_4_), 69.4 (5CH Cp), 69.9–71.3 (14CH_2_-O), 85.9 (CH-OH), 87.8 (C C_5_H_4_), 115.3 (2CH C_6_H_4_), 115.7 (2CH C_6_H_4_), 123.4 (CH isoindole), 123.9 (CH isoindole), 130.0 (CH isoindole), 130.5 (2CH C_6_H_4_), 131.2 (2CH C_6_H_4_), 132.2 (CH isoindole), 132.5 (C), 133.7 (C), 136.2 (C), 136.7 (C), 138.6 (C), 141.0 (C), 154.9 (C), 155.2 (C), 167.9 (CO). IR (ATR, ν cm^−1^): 2105 (azide), 1678 (CO). HRMS (ESI, C_51_H_62_FeN_4_NaO_11_: [M + Na]^+^) calcd: 985.36567, found: 983.3664.

#### 3.12.6. 2,3-Dihydro-3-(2-methoxycarbonylethylthio)-2-[4-ferrocenyl-5,5-bis-(4-hydroxyphenyl)-ent-4-enyl]-1*H*-isoindol-1-one (**25**)

From compound **18** (0.305 g, 0.52 mmol) and methyl 3-mercaptopropionate (0.219 g, 1.8 mmol), reaction time of 4 h. Eluent: cyclohexane/ethyl acetate 2: 1. Yield of 83%. ^1^H NMR (acetone-d_6_): δ 1.79–1.96 (m, 2H, CH_2_), 1.99–2.28 (m, 4H, SCH_2_CH_2_COO), 2.48–2.76 (m, 2H, CH_2_-C=C), 3.19–3.33 (m, 1H, CH_2_N), 3.56 (s, 3H, OMe), 3.74–3.78 (m, 1H, C_5_H_4_), 3.90–4.15 (m, 2H, CH_2_N + C_5_H_4_), 4.05–4.16 (m, 7H, 2C_5_H_4_ + Cp), 4.91 (CH-S), 6.68 (d, *J* = 8.5 Hz, 2H, C_6_H_4_), 6.72 (d, *J* = 8.5 Hz, 2H, C_6_H_4_), 6.85 (d, *J* = 8.5 Hz, 2H, C_6_H_4_), 7.01 (d, *J* = 8.5 Hz, 2H, C_6_H_4_), 7.49–7.59 (m, 1H, isoindole), 7.63–7.77 (m, 3H, isoindole), 8.23 (s, 1H, OH), 8.35 (s, 1H, OH). ^13^C NMR (acetone-d_6_): δ 22.2 (CH_2_), 29.4 (CH_2_), 32.8 (CH_2_), 34.5 (CH_2_), 39.4 (CH_2_), 51.8 (OCH_3_), 63.1 (CH-S), 68.6 (CH C_5_H_4_), 68.7 (CH C_5_H_4_), 69.80 (CH C_5_H_4_), 69.85 (5CH Cp), 70.1 (CH C_5_H_4_), 88.4 (C C_5_H_4_), 115.7 (2CH C_6_H_4_), 116.1 (2CH C_6_H_4_), 123.5 (CH isoindole), 124.8 (CH isoindole), 129.7 (CH isoindole), 131.3 (2CH C_6_H_4_), 131.8 (2CH C_6_H_4_), 132.8 (CH isoindole), 132.9 (C), 134.6 (C), 136.8 (C), 137.2 (C), 139.3 (C), 144.7 (C), 156.69 (C), 156.74 (C), 167.6 (CO), 172.2 (COO). IR (ATR, ν cm^−1^): 1714 (CO), 1660 (CO). HRMS (ESI, C_39_H_37_FeNO_5_S: [M]^+^) calcd: 687.1736, found: 687.1736. Crystal data: C_42_H_43_FeNO_6_S, triclinic P -1, a = 10.7220(5) Å, b = 13.2436(6) Å, c = 15.3329(6) Å, α = 97.196(3)°, β = 103.916(4)°, γ = 113.797(4)°, V = 1872.10(16) Å^3^, Z = 2, orange plate 0.3 × 0.2 × 0.1 mm^3^, μ = 0.507 mm^−1^, min/max transmission = 0.57/1.00, T = 200 K, λ = 0.71073 Å, θ range = 1.74° to 28.28°, 25312 reflections measured, 9219 independent, R_int_ = 0.0892, completeness = 0.997, 471 parameters, 0 restraints, final R indices R1 [I > 2σ (I)] = 0.0630 and wR2 (all data) = 0.1676, GOF on F^2^ = 1.102, largest difference peak/hole = 1.52/−0.59 e·Å^−3^.

#### 3.12.7. 2,3-Dihydro-3-(2-carboxyethylthio)-2-[4-ferrocenyl-5,5-bis-(4-hydroxyphenyl)-pent-4-enyl]-1*H*-isoindol-1-one (**26**)

From compound **18** (0.3 g, 0.512 mmol) and 3-mercaptopropionic acid (0.19 g, 0.16 mL, 1.8 mmol), time overnight. Eluent: ethyl acetate. Yield of 88%. ^1^H NMR (DMSO-d_6_): δ 1.51–2.18 (m, 6H, CH_2_ + SCH_2_-CH_2_COO), 2.34–2.59 (m, 2H, CH_2_-C=C), 3.02–3.25 (m, 1H, CH_2_N), 3.63–3.69 (m, 1H, C_5_H_4_), 3.71–3.86 (m, 1H, CH_2_N), 3.95–4.16 (m, 8H, 3C_5_H_4_ + Cp), 5.04 (CH-S), 6.59 (d, *J* = 8.3 Hz, 2H, C_6_H_4_), 6.62 (d, *J* = 8.3 Hz, 2H, C_6_H_4_), 6.75 (d, *J* = 8.3 Hz, 2H, C_6_H_4_), 6.91 (d, *J* = 8.3 Hz, 2H, C_6_H_4_), 7.51–7.74 (m, 4H, isoindole). ^13^C NMR (DMSO-d_6_): δ 21.0 (CH_2_), 28.1 (CH_2_), 31.3 (CH_2_), 33.6 (CH_2_), 38.3 (CH_2_), 61.8 (CH-S), 67.6 (CH C_5_H_4_), 67.7 (CH C_5_H_4_), 68.5 (CH C_5_H_4_), 68.6 (CH C_5_H_4_), 68.9 (5CH Cp), 86.7 (C C_5_H_4_), 114.8 (2CH C_6_H_4_), 115.0 (2CH C_6_H_4_), 122.3 (CH isoindole), 123.7 (CH isoindole), 128.8 (CH isoindole), 129.8 (2CH C_6_H_4_), 130.2 (2CH C_6_H_4_), 131.1 (C), 132.0 (CH isoindole), 132.8 (C), 134.9 (C), 135.3 (C), 137.5 (C), 143.3 (C), 155.5 (2C), 166.2 (CO), 172.3 (COO). IR (ATR, ν cm^−1^): 1704 (CO), 1639 (CO). HRMS (ESI, C_38_H_34_FeNO_5_S: [M-H]^-^) calcd: 672.1513, found: 672.1511.

#### 3.12.8. S-{2,3-Dihydro-2-[4-ferrocenyl-5,5-bis-(4-hydroxyphenyl)-pent-4-enyl]-1*H*-isoindol-1-one-3-yl}-1-thia-4,7,10,13,16,19,22-heptaoxatricosane (**27**)

From compound **18** (0.3422 g, 0.584 mmol) and O-(2-mercaptoethyl)-O′-methyl-hexa(ethylene glycol) (0.25 g, 0.701 mmol). Eluent: cyclohexane/ethyl acetate 1: 2. Yield of 76%. ^1^H NMR (acetone-d_6_): δ 1.72–1.96 (m, 2H, CH_2_), 1.98–2.16 (m, 2H, CH_2_-S), 2.48–2.78 (m, 2H, CH_2_-C=C), 3.16–3.69 (m, 30H, CH_2_N + CH_2_(OCH_2_CH_2_)_6_-OCH_3_), 3.74–3.81 (m, 1H, C_5_H_4_), 3.88–4.03 (m, 2H, CH_2_N + C_5_H_4_), 4.03–4.20 (m, 7H, Cp + 2C_5_H_4_), 4.96 (s, 1H, CH-S), 6.69 (d, *J* = 8.5 Hz, 2H, C_6_H_4_), 6.74 (d, *J* = 8.5 Hz, 2H, C_6_H_4_), 6.85 (d, *J* = 8.5 Hz, 2H, C_6_H_4_), 7.02 (d, *J* = 8.5 Hz, 2H, C_6_H_4_), 7.54 (td, *J* = 7.2, 1.6 Hz, 1H, isoindole), 7.61–7.77 (m, 3H, isoindole), 8.35 (s broad, 2H, OH). ^13^C NMR (acetone-d_6_): δ 27.3 (CH_2_), 29.4 (CH_2_), 32.8 (CH_2_), 39.5 (CH_2_), 58.8 (OCH_3_), 63.1 (CH-S), 68.6 (CH C_5_H_4_), 68.7 (CH C_5_H_4_), 69.79 (CH C_5_H_4_), 69.84 (5CH Cp), 70.1 (CH C_5_H_4_), 70.7 (CH_2_-O), 70.8 (CH_2_-O), 71.0 (2CH_2_-O), 71.2 (8CH_2_-O), 72.6 (CH_2_-O), 88.4 (C C_5_H_4_), 115.8 (2CH C_6_H_4_), 116.1 (2CH C_6_H_4_), 123.4 (CH isoindole), 124.9 (CH isoindole), 129.6 (CH isoindole), 131.3 (2CH C_6_H_4_), 131.8 (2CH C_6_H_4_), 132.76 (CH isoindole), 132.84 (C), 134.5 (C), 136.8 (C), 137.1 (C), 139.3 (C), 144.9 (C), 156.6 (C), 156.7 (C), 167.6 (CO). IR (ATR, ν cm^−1^): 1669 (CO). HRMS (ESI, C_50_H_61_FeNNaO_10_S: [M + Na]^+^) calcd: 946.32578, found: 946.3258.

### 3.13. X-ray Crystal Structure Determination

Single crystals were selected, mounted, and transferred into a cold nitrogen gas stream. Intensity data was collected with a Bruker Kappa-APEX2 system using micro-source Cu-Kα radiation (**15**, **23**) or a home-made diffractometer using a Rigaku MM007HF Mo-Kα source (**25**). Unit-cell parameters determination, data collection strategy, integration, and absorption correction were carried out with the Bruker APEX2 (**15**, **23**) or CrysAlisPro (**25**) suites of programs. The structures were solved with SHELXT and refined anisotropically by full-matrix least-squares methods with SHELXL using WinGX (**15**, **23**) or Olex2 (**25**). The structures were deposited at the Cambridge Crystallographic Data Centre with numbers CCDC 2166261 (**15**), 2166262 (**23**), and 2166263 (**25**), and can be obtained free of charge via www.ccdc.cam.ac.uk.

### 3.14. Cell Culture and Viability Assay

MDA-MB-231, MCF-7, and hTERT-RPE1 cells were obtained from ATCC. Cells were maintained in a monolayer culture in DMEM with phenol red with Glutamax I (MDA-MB-231) or 2 mM L-glutamine (MCF-7) or 15 mM HEPES (hTERT-RPE1) supplemented with 10% FBS at 37 °C in a humidified atmosphere and 5% CO_2_. Stock solutions of the complexes were prepared in DMSO. Cells were seeded in 96-well plates at a density of 4000 cells/well. After overnight attachment, a dilution series of the compounds were added in the medium, and cells were incubated for a further 72 h. The percentage of DMSO in the culture medium did not exceed 1%. Cell viability was evaluated by using a colorimetric method based on the tetrazolium salt MTT [3-(4,5-dimethylthiazol-2-yl)-2,5-diphenyltetrazolium bromide], which is reduced by viable cells to yield purple formazan crystals. After 72 h, the medium was removed and the cells were incubated with MTT solution in PBS (10 μL of a 5 mg/mL) for 2–3 h of incubation. The formed purple formazan crystals were dissolved in 100 μL DMSO by thorough shaking, and the absorbance at 560 nm was read using a microplate reader (FLUOstar OPTIMA, BMG Labtech, Ortenberg, Germany). Each test was performed with at least three replicates and repeated at least three times. EC_50_ were determined using *Dr Fit* software [33].

## 4. Conclusions

Our aim was to design a versatile strategy to graft various functionalities to the scaffold of imido-ferrocidiphenols and evaluate their impact on their biological activity. The presence of the imide group provided us with an efficient entry to achieve this aim. Ferrocenyl alkylimides were initially used as simple models to investigate the conversion of imides to α-hydroxylactams by selective reduction of one of their carbonyl groups. These α-hydroxylactams are precursors of highly electrophilic *N*-acyliminium ions that were efficiently trapped by O-, S-, and π-nucleophiles to afford lactams in good to excellent yield. Translation of this strategy to phthalimido ferrocidiphenol enabled the grafting of various substituents carrying useful chemical functions such as carboxylic acid, alcohol, azide, or terminal alkyne. Cell viability assays on breast cancer cells showed that grafting of various substituents on the phthalimide moiety had diverse effects on the overall antiproliferative activity with respect to the reference **Fc-OH-TAM-3**. The chemistry of α-hydroxylactams/*N*-acyliminium ions then appears as an efficient synthetic route to connect functional groups of interest to ferrocidiphenol, such as azide or terminal alkyne. As a short-term perspective, these groups will provide starting points to conjugate bioligands for targeting purposes or for click-based chemical proteomics with the final aim to uncover the protein targets of ferrociphenols. 

## Figures and Tables

**Table 2:** Yields in α-hydroxylactams **6** and α-methoxylactams **10** generated according to reactions depicted in Scheme 1 and Scheme 2.

n	α−Hydroxylactam 6		α−Methoxylactam 10	
	Product	Yield%	Product	Yield%
**Succinimide derivative (pyrrolidone series)**				
1	**6a**	66	**10a**	failed
2	**6b**	33	**10b**	89
3	**6c**	61	- ^a^	-
**Phthalimide derivative (isoindolone series)**				
1	**6e**	99	**10e**	90
2	**6f ^b^**	99	**10f**	100
3	**6g**	90	**10g**	94
4	**6h**	89	**10h**	79
**2,3-Naphthalenecarboximide derivative (benzo[f]isoindolone)**				
2	**6i**	12	-	-
**Glutarimide derivative (piperidone)**				
2	**6j**	47 (unstable)	-	-
**1,8-Naphthalimide derivative (Benzo[de]isoquinolinone)**				
2	**6k**	No reaction		

^a^ not done; ^b^ reported in Ref. [26].

**Table 3:** Half-maximal effective concentrations (EC_50_) of selected ferrocidiphenols on tumorigenic and non-tumorigenic cell lines.

Compound	MDA-MB-231	MCF-7	HTERT-RPE1
	EC_50_ (µM) ^a^		
**Fc-OH-TAM-3**	1.9 ± 0.3	1.8 ± 0.2	3.5 ± 0.2
**3h**	>50	>50	>50
**20**	6.3 ± 2.1	10.6 ± 0.3	5.0 ± 0.2
**22**	8.8 ± 2.5	29.9 ± 1.3	24.7 ± 1.1
**23**	2.4 ± 0.5	4.2 ± 0.7	12.9 ± 3.3
**25**	18.0 ± 0.4	4.7 ± 1.4	9.1 ± 0.4

^a^ measured on 96-well plates seeded with 4000 cells/well; cell viability measured after 3 days by MTT assay; the means and standard deviations result from three independent experiments.

## Data Availability

Data is contained within the article or Supplementary Material.

## Supplementary Materials

The following supporting information can be downloaded at: https://www.mdpi.com/article/10.3390/molecules27144549/s1, Table S1: crystallographic data of compounds **15**, **23**, and **25**; Figures S1–S49: spectroscopic data of newly synthesized compounds; Figure S50: solubility studies of **18**, **20**, **22**, **24**, and **27**; Figure S51: Enzymatic oxidation of **19**, **22**, **23,** and **25**; Figure S52: MTT cell viability assay for compounds **Fc-OH-TAM-3**, **3h**, **20**, **22**, **23,** and **25**; Figure S53: RP-HPLC traces of **3h**, **20**, **22**, **23**, and **25**; CIF files for compounds **15**, **23**, and **25**.

## Sample Availability

Samples of the compounds **19**–**27**. are available from the authors upon request.

## References

[1] Harbeck N., Penault-Llorca F.D.R., Cortes J., Gnant M., Houssami N., Poortmans P., Ruddy K., Tsang J., Cardoso F. (2019). Breast cancer. Nat. Rev. Dis. Primers.

[2] Kowalski K. (2018). Recent developments in the chemistry of ferrocenyl secondary natural product conjugates. Coord. Chem. Rev..

[3] Sijongesonke P., Aderibigbe B.A. (2019). Ferrocene-Based Compounds with Antimalaria/Anticancer Activity. Molecules.

[4] Wang R., Chen H., Yan W., Zheng M., Zhang T., Zhang Y. (2020). Ferrocene-containing hybrids as potential anticancer agents: Current developments, mechanisms of action and structure-activity relationships. Eur. J. Med. Chem..

[5] Singh A., Lumb I., Mehra V., Kumar V. (2019). Ferrocene-Appended Pharmacophores: An exciting approach for modulating biological potential of organic scaffolds. Dalton Trans..

[6] Sharma B., Kumar V. (2021). Has Ferrocene Really Delivered Its Role in Accentuating the Bioactivity of Organic Scaffolds?. J. Med. Chem..

[7] Raičević V., Radulović N., Sakač M. (2022). Toward Selective Anticancer Agents: Ferrocene-Steroid Conjugates. Eur. J. Inorg. Chem..

[8] Jaouen G., Vessières A., Top S. (2015). Ferrocifen type anti cancer drugs. Chem. Soc. Rev..

[9] Vessières A., Wang Y., McGlinchey M.J., Jaouen G. (2020). Multifaceted chemical behaviour of metallocene (M = Fe, Os) quinone methides. Their contribution to biology. Coord. Chem. Rev..

[10] Citta A., Folda A., Bindoli A., Pigeon P., Top S., Vessières A., Salmain M., Jaouen G., Rigobello M.P. (2014). Evidence for Targeting Thioredoxin Reductases with Ferrocenyl Quinone Methides. A Possible Molecular Basis for the Antiproliferative Effect of Hydroxyferrocifens on Cancer Cells. J. Med. Chem..

[11] Najlaoui F., Pigeon P., Abdelkafi Z., Leclerc S., Durand P., El Ayeb M., Marrakchi N., Rhouma A., Jaouen G., Gibaud S. (2015). Phthalimido-ferrocidiphenol cyclodextrin complexes: Characterization and anticancer activity. Int. J. Pharm..

[12] Pigeon P., Wang Y., Top S., Najlaoui F., Garcia Alvarez M., Bignon J., McGlinchey M.J., Jaouen G. (2017). A new series of succinimido-ferrociphenols and related heterocyclic species induce strong antiproliferative effects, especially against ovarian cancer cells resistant to cisplatin. J. Med. Chem..

[13] Wang Y., Pigeon P., Top S., García J., Troufflard C., Ciofini I., McGlinchey M.J., Jaouen G. (2019). Atypical lone pair-π interaction with quinone methides in a series of imido-ferrociphenol anticancer drug candidates. Angew. Chem. Int. Ed..

[14] Vessières A., Quissac E., Lemaire N., Alentorn A., Domeracka P., Pigeon P., Sanson M., Idbaih A., Verreault M. (2021). Heterogeneity of Response to Iron-Based Metallodrugs in Glioblastoma Is Associated with Differences in Chemical Structures and Driven by FAS Expression Dynamics and Transcriptomic Subtypes. Int. J. Mol. Sci..

[15] Topin-Ruiz S.N., Mellinger A.L., Lepeltier E., Bourreau C., Fouillet J., Riou J.R.M., Jaouen G., Martin L., Passirani C., Clere N. (2020). p722 ferrocifen loaded lipid nanocapsules improve survival of murine xenografted-melanoma via a potentiation of apoptosis and an activation of CD8+ T lymphocytes. Int. J. Pharm..

[16] Yazici A., Pyne S.G. (2009). Intermolecular Addition Reactions of N-Acyliminium Ions (Part II). Synthesis.

[17] Yazici A., Pyne S.G. (2009). Intermolecular Addition Reactions of N-Acyliminium Ions (Part I). Synthesis.

[18] Wu P., Nielsen T.E. (2017). Scaffold Diversity from N-Acyliminium Ions. Chem. Rev..

[19] Hwang H.J., Carey J.R., Brower E.T., Gengenbach A.J., Abramite J.A., Lu Y. (2005). Blue Ferrocenium Azurin: An Organometalloprotein with Tunable Redox Properties. J. Am. Chem. Soc..

[20] Elecko P., Foltinova P., Salisova M., Solcaniova E., Toma S. (1974). Synthesis, proton magnetic resonance spectra, and biological activity of haloacylferrocenes. Chem. Zvesti.

[21] Wilbert G., Traud S., Zentel R. (1997). Liquid crystalline main-chain polymers containing the ferrocene unit as a side group. Part 2. Variation of the spacer length. Macromol. Chem. Phys..

[22] Pigeon P., Decroix B. (1997). Tetracyclic systems: Synthesis of isoindolo[1,2-b]thieno[2,3(3,2 or 3,4)-e][1,3]thiazocines and isoindolo[2,1-a]thieno[2,3(3,2 or 3,4)-f][1,4] and -[1,5]diazocines. J. Heterocycl. Chem..

[23] Davis W.L., Shago R.F., Langner E.H.G., Swarts J.C. (2005). Synthesis and electrochemical properties of a series of ferrocene-containing alcohols. Polyhedron.

[24] Dol G.C., Kamer P.C.J., Hartl F., Nolte P.W.N.M.V. (1998). Electrochemical and binding properties of a novel ferrocene containing redox-active basket-shaped host molecule. J. Chem. Soc. Dalton Trans..

[25] Wang Y., Pigeon P., Li W., Jiangkun Y., Dansette P.M., Othman M., McGlinchey M.J., Jaouen G. (2022). Diversity-oriented synthesis and bioactivity evaluation of N-substituted ferrocifen compounds as novel antiproliferative agents against TNBC cancer cells. Eur. J. Med. Chem..

[26] Achari K.M.M., Ramanathan C.R. (2017). Conformationally rigid chiral ferrocene derivative: Synthesis, resolution and stereochemical assignment. Tetrahedron Asymmetry.

[27] Speckamp W.N., Hiemstra H. (1985). Intramolecular reactions of N-acyliminium intermediates. Tetrahedron.

[28] Pigeon P., Decroix B. (1997). Introduction of a carboxymethylamino(or oxy or thio) group in the 3 position of 2-aryl(or arylmethyl)isoindol-1-ones from a-hydroxylactams. Synth. Commun..

[29] Osante I.A., Collado M.I., Lete E., Sotomayor N. (2001). Stereodivergent Synthesis of Hetero-Fused Isoquinolines by Acyliminium and Metallation Methods. Eur. J. Org. Chem..

[30] Othman M., Pigeon P., Decroix B. (1997). Acyliminium ion cyclizations: Synthesis of thieno[2’,3’:3,4]pyrrolo[2,1-a]isoindolone and benzo[a]thieno[2,3(3,2 or 3,4)-g]indolizinones. Tetrahedron.

[31] Pigeon P., Decroix B. (1996). Synthesis of thieno[2’,3’(3’,4’ or 3’,2’):5,6]azepino[2,1-a]isoindolediones from N-thienyl-2(3)-ylmethylphthalimides. J. Heterocycl. Chem..

[32] Tonolo F., Salmain M., Scalcon V., Top S., Pigeon P., Folda A., Caron B., McGlinchey M.J., Toillon R.A., Bindoli A. (2019). Small Structural Differences between Two Ferrocenyl Diphenols Determine Large Discrepancies of Reactivity and Biological Effects. ChemMedChem.

[33] Di Veroli G.Y., Fornari C., Goldlust I., Mills G., Boon Koh S., Bramhall J.L., Richards F.M., Jodrell I. (2015). An automated fitting procedure and software for dose-response curves with multiphasic features. Sci. Rep..

