# Supplementary material for: α-Hydroxylactams as Efficient Entries to Diversely Functionalized Ferrociphenols: Synthesis and Antiproliferative Activity Studies
Source: Molecules. 2022 Jul 16;27(14):4549. doi: 10.3390/molecules27144549 (PMC9324693; doi:10.3390/molecules27144549)
Supplement: Supplementary file 1 [file molecules-27-04549-s001.zip › molecules-1705428-supplementary.pdf]

Supplementary Materials

α-Hydroxylactams as efficient entries to diversely functionalized ferrociphenols: Synthesis and anticancer activity studies

Pascal Pigeon, Marie Gaschard, Mohamed Othman, Michèle Salmain and Gérard Jaouen

Table of contents

Figure S1: Structural data for compound **3a** 1
Figure S2: Structural data for compound **3b** 3
Figure S3: Structural data for compound **3c** 5
Figure S4: Structural data for compound **3d** 7
Figure S5: Structural data for compound **3e** 8
Figure S6: Structural data for compound **3f** 10
Figure S7: Structural data for compound **3g** 12
Figure S8: Structural data for compound **3h** 14
Figure S9: Structural data for compound **3i** 16
Figure S10: Structural data for compound **3j** 18
Figure S11: Structural data for compound **3k** 20
Figure S12: Structural data for compound **4b** 22
Figure S13: Structural data for compound **4c** 24
Figure S14: Structural data for compound **4d** 26
Figure S15: Structural data for compound **4f** 28
Figure S16: Structural data for compound **4g** 30
Figure S17: Structural data for compound **4h** 32
Figure S18: Structural data for compound **6a** 34
Figure S19: Structural data for compound **7a** 37
Figure S20: Structural data for compound **6b** 40
Figure S21: Structural data for compound **6c** 41
Figure S22: Structural data for compound **6e** 44
Figure S23: Structural data for compound **6f** 46
Figure S24: Structural data for compound **6g** 48
Figure S25: Structural data for compound **6h** 50
Figure S26: Structural data for compound **6i** 52
Figure S27: Structural data for compound **6j** 54
Figure S28: Structural data for compound **7j** 55
Figure S29: Structural data for compound **8j** 57
Figure S30: Structural data for compound **9j** 60
Figure S31: Structural data for compound **10b** 62
Figure S32: Structural data for compound **10e** 65
Figure S33: Structural data for compound **10f** 67
Figure S34: Structural data for compound **10g** 69
Figure S35: Structural data for compound **10h** 71
Figure S36: Structural data for compound **11** 73
Figure S37: Structural data for compound **13** 76
Figure S38: Structural data for compound **14** 78
Figure S39: Structural data for compound **15** 80
Figure S40: Structural data for compound **16** 83
Figure S41: Structural data for compound **17** 86
Figure S42: Structural data for compound **20** 88
Figure S43: Structural data for compound **21** 90
Figure S44: Structural data for compound **22** 93
Figure S45: Structural data for compound **23** 96
Figure S46: Structural data for compound **24** 99

Figure S47: Structural data for compound **25** 102
Figure S48: Structural data for compound **26** 105
Figure S49: Structural data for compound **27** 108
Table S1: Crystallographic data for **15**, **23** and **25** 111
Figure S50: Solubility studies for compounds **18**, **20**, **22**, **24** and **27** 112
Figure S51: Conversion to quinone methide: UV-VIS spectra of **19**, **20**, **22**, **23** and **25** 114
Figure S52: MTT cell viability assay for compounds **Fc-OH-TAM-3**, **3h**, **20**, **22**, **23** and **25** ..115
Figure S53: RP-HPLC traces for compounds **Fc-OH-TAM-3**, **3h**, **20**, **22**, **23** and **25**........ 118

Figure S1: ^{1}H (in CDCl3), ^{13}C (in CDCl3) NMR, HR-MS and IR data for compound **3a**

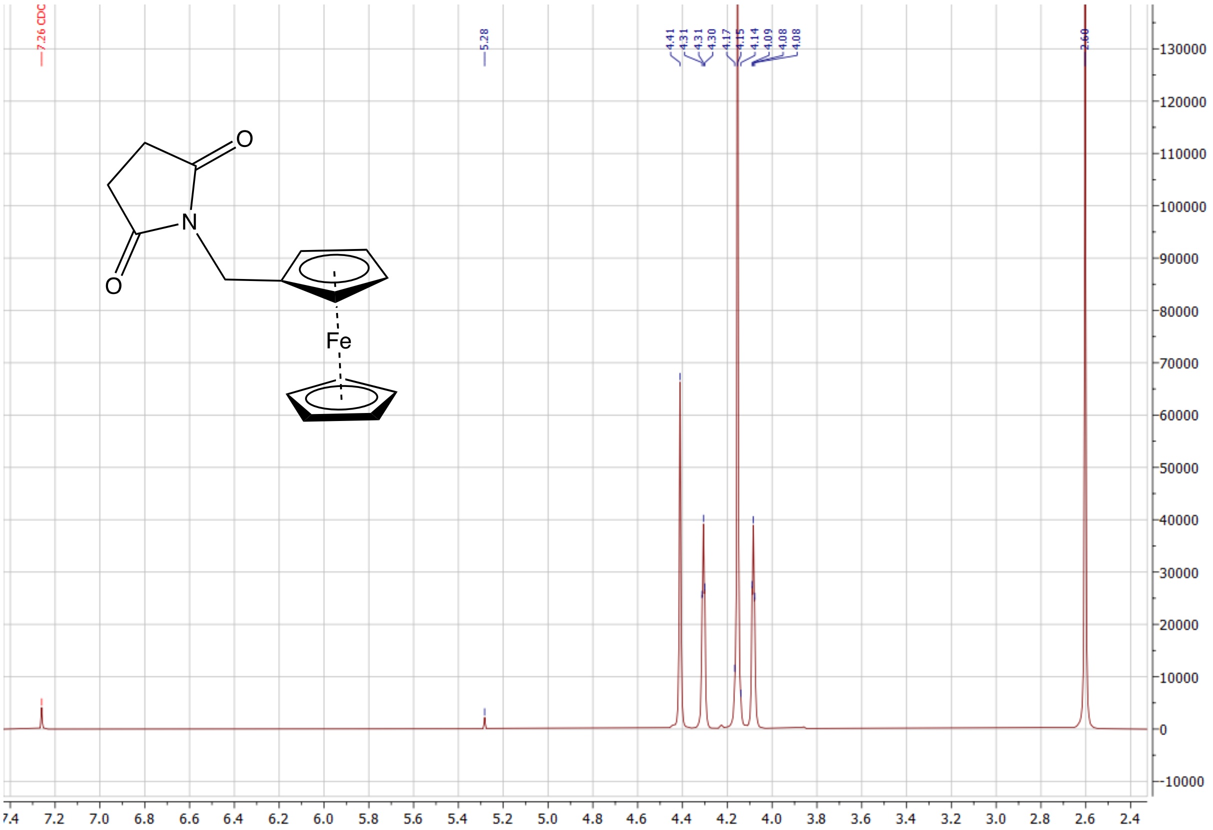

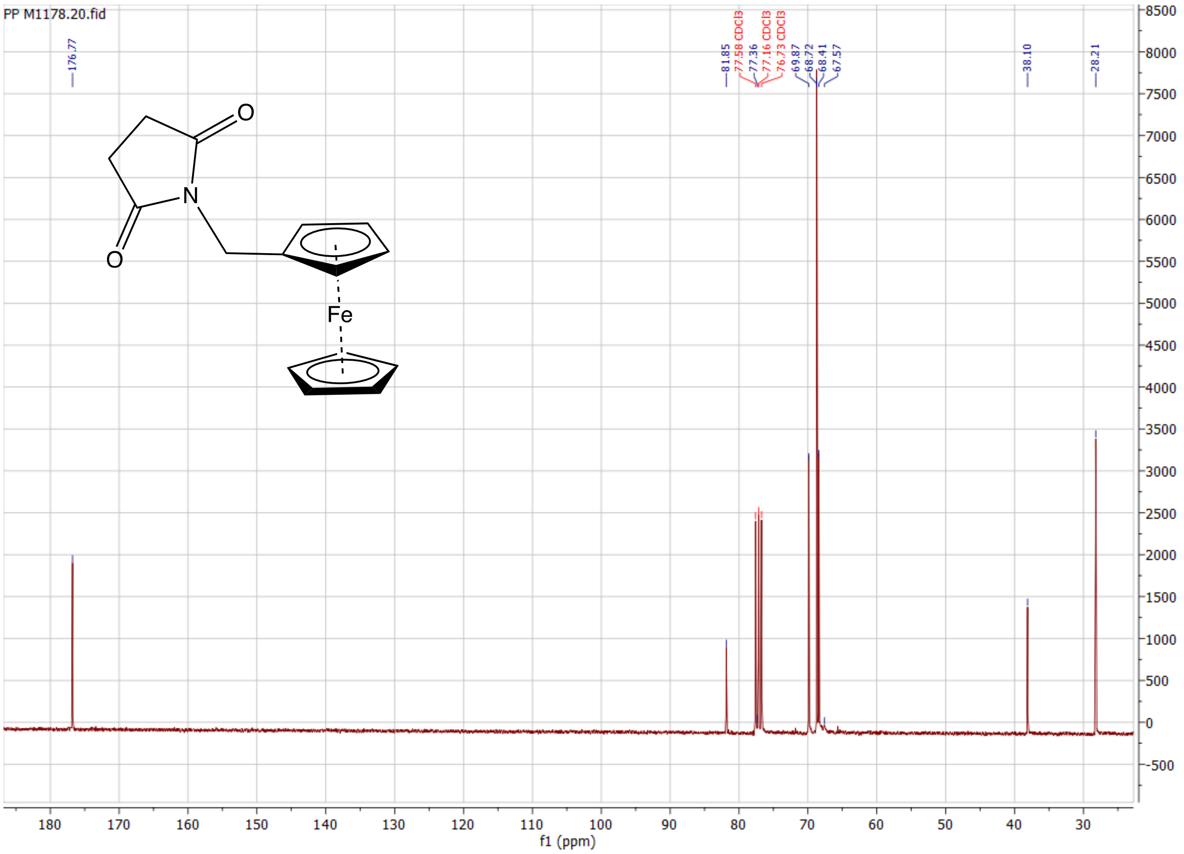

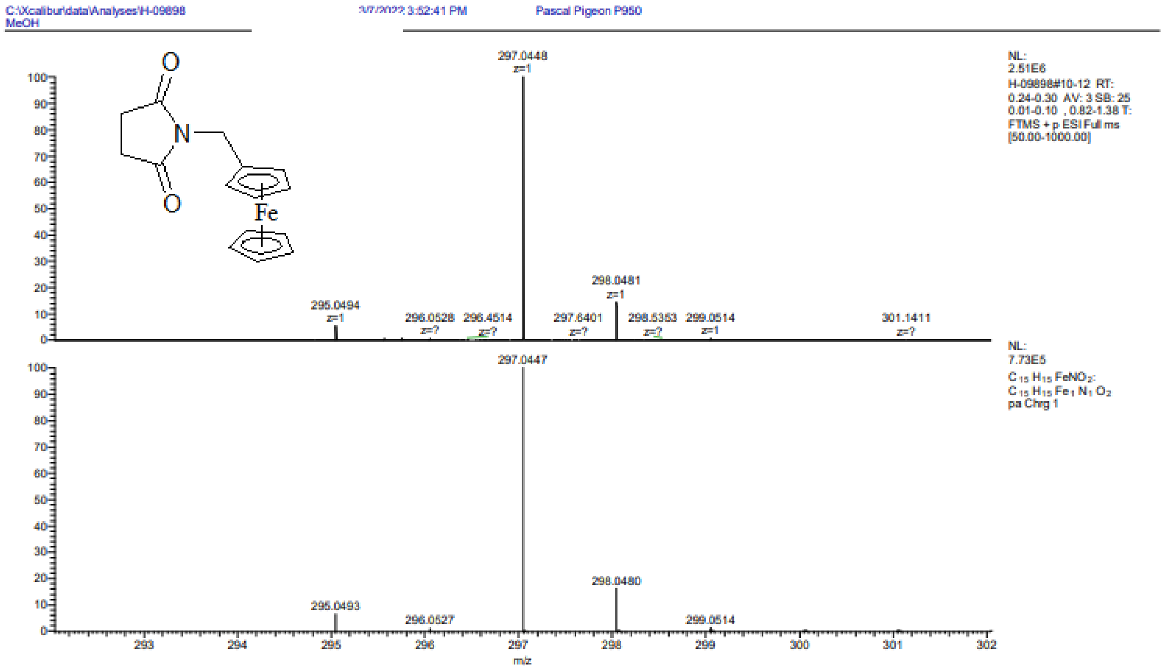

Experimental/theoretical isotopic pattern MS spectrum

Error = 0.4 ppm; Relative Intensity (%) 100

HRMS (ESI) m/z: [M]+ Calcd for C15H15FeNO2 297.0447 . Found 297.0448; (Error: 0.4 ppm).

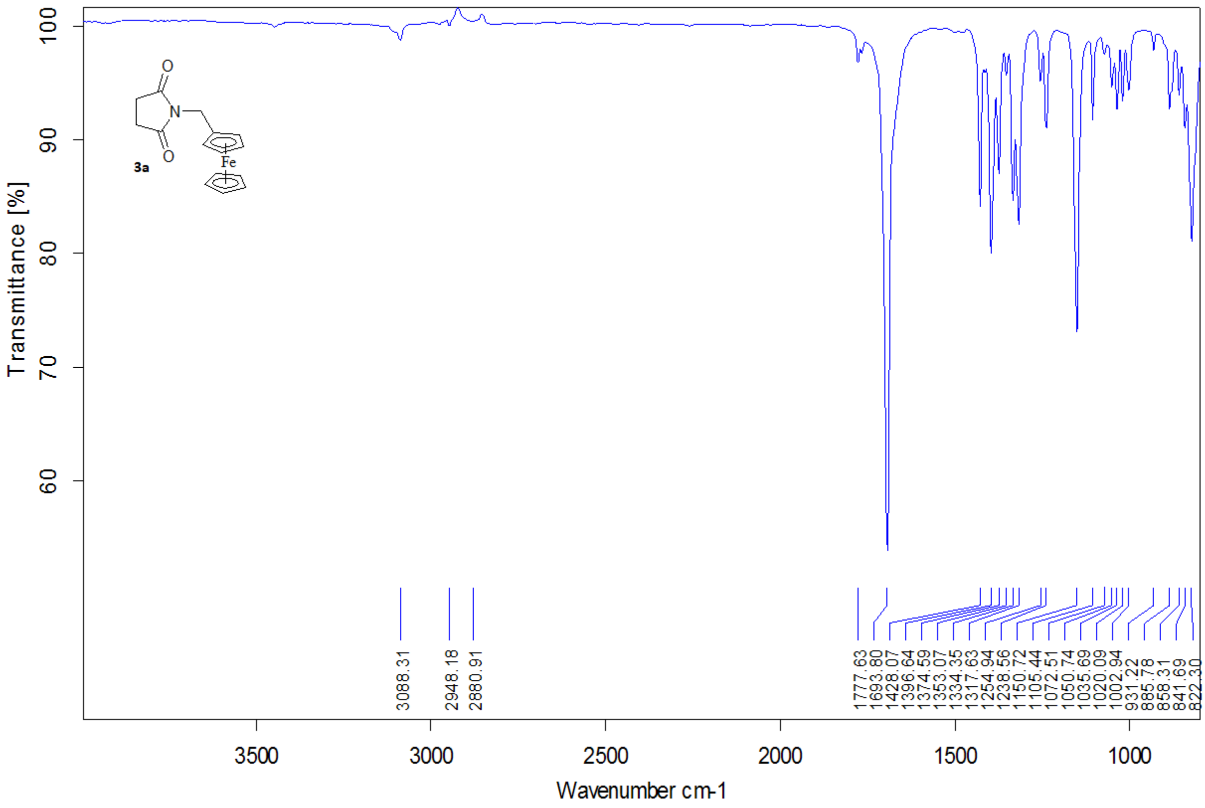

Figure S2: ^{1}H (in $CDCl_3$), ^{13}C (in $CDCl_3$) NMR, HR-MS and IR data for compound **3b**

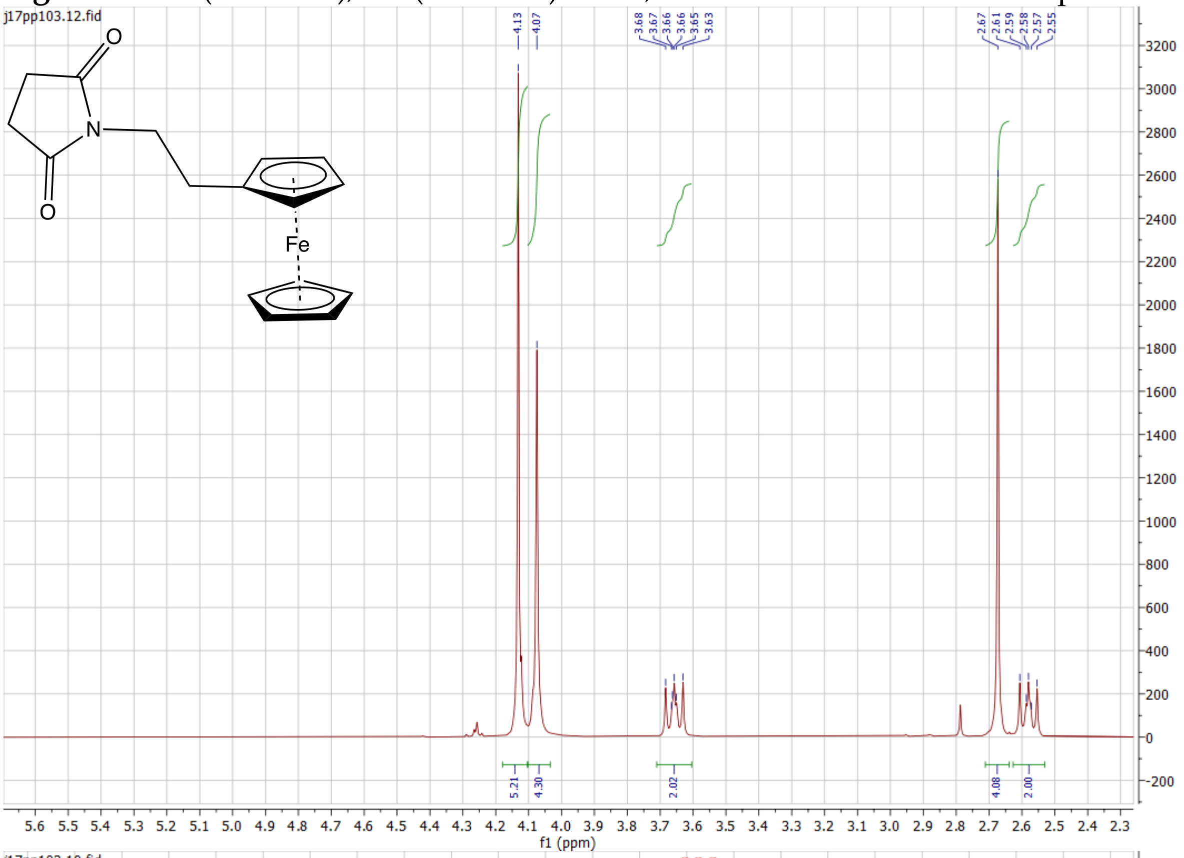

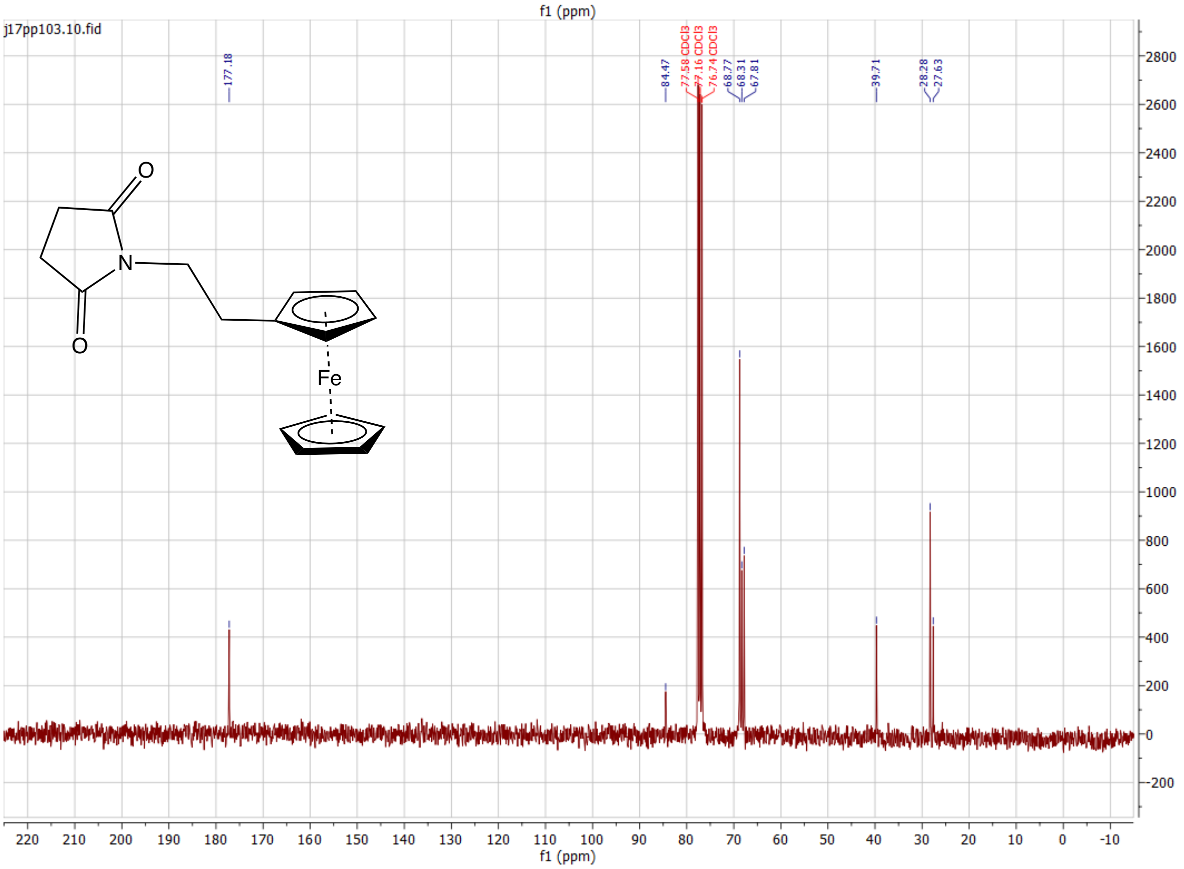

Single Mass Analysis
Tolerance = 5.0 PPM / DBE: min = -1.5, max = 100.0
Element prediction: Off
Number of isotope peaks used for i-FIT = 9

Monoisotopic Mass, Odd Electron Ions
291 formula(e) evaluated with 1 results within limits (all results (up to 1000) for each mass)
Elements Used:
C: 0-100 H: 1-120 N: 0-10 O: 0-10 Fe: 1-1

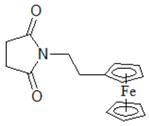

06-Sep-2013 5::7::2
ENSCP_P729 21 (0.572) Cm (18:24)
MeOH
LCT Premier XE KE483
1: TOF MS ES+
2.33e+004

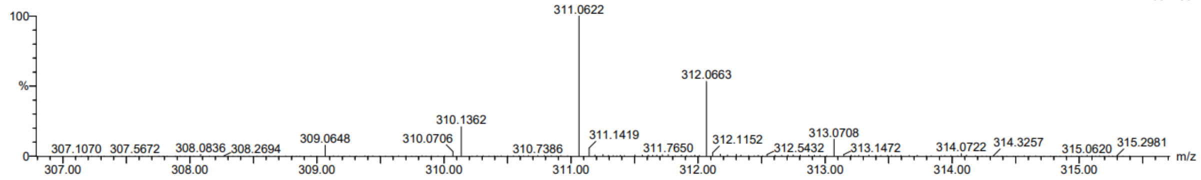

Minimum:				-1.5			
Maximum:		5.0	5.0	100.0			
Mass	Calc. Mass	mDa	PPM	DBE	i-FIT	i-FIT (Norm)	Formula
311.0622	311.0609	1.3	4.2	9.0	724.2	0.0	C16 H17 N O2 Fe

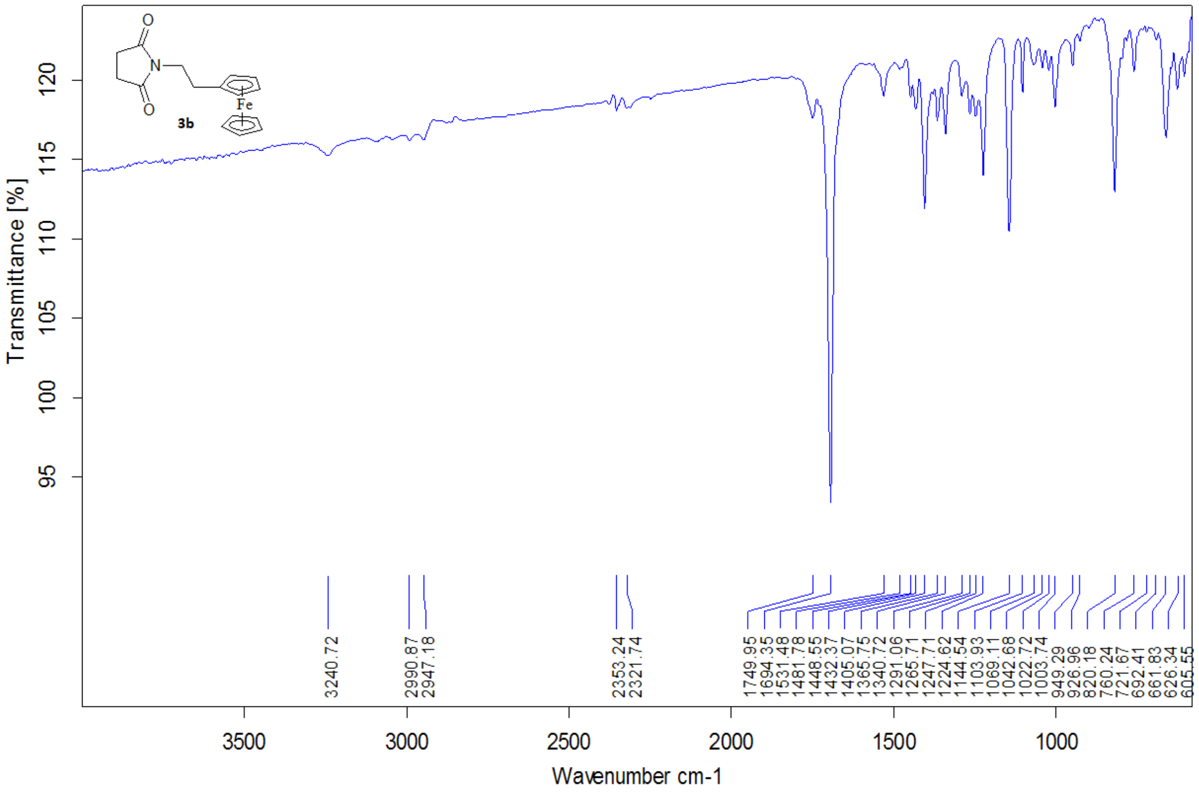

Figure S3: ^{1}H (in $CDCl_3$), ^{13}C (in $CDCl_3$) NMR, HR-MS and IR data for compound **3c**

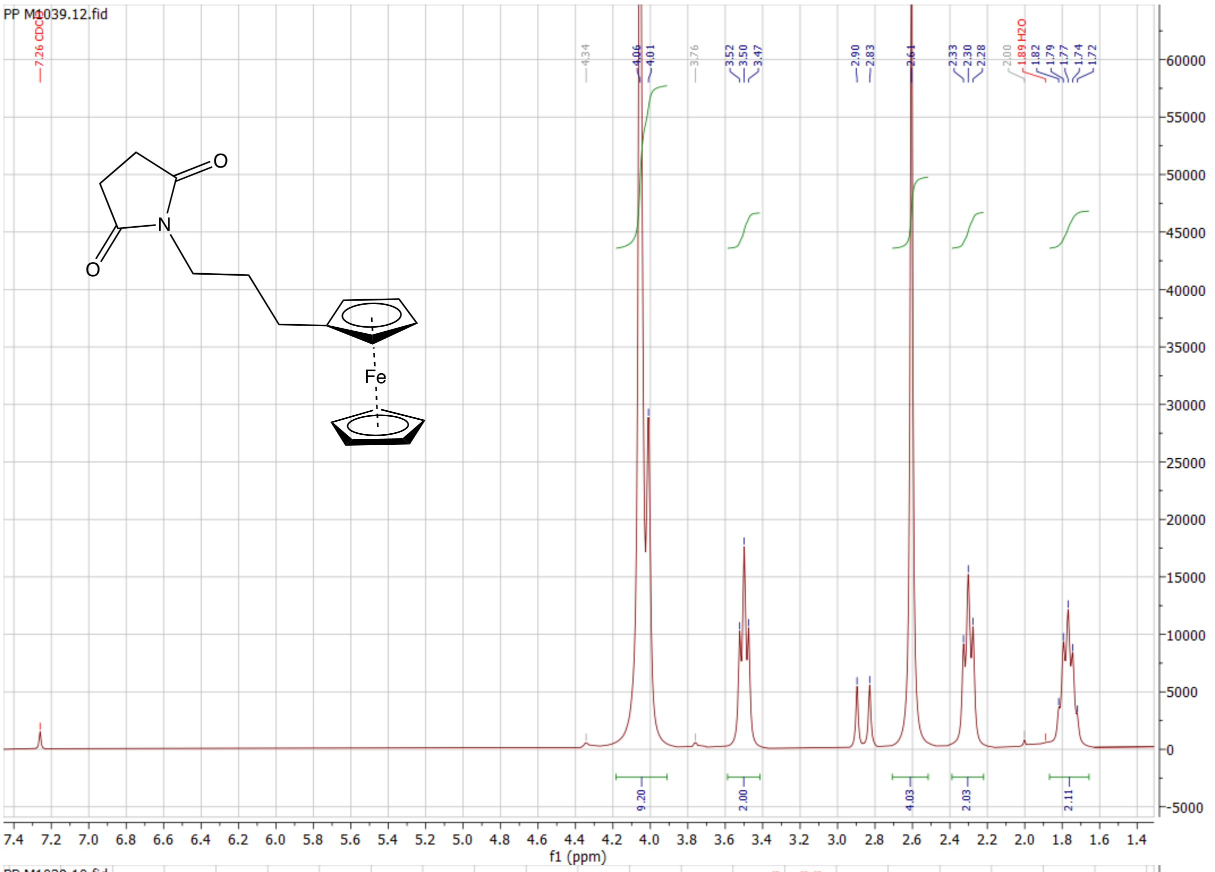

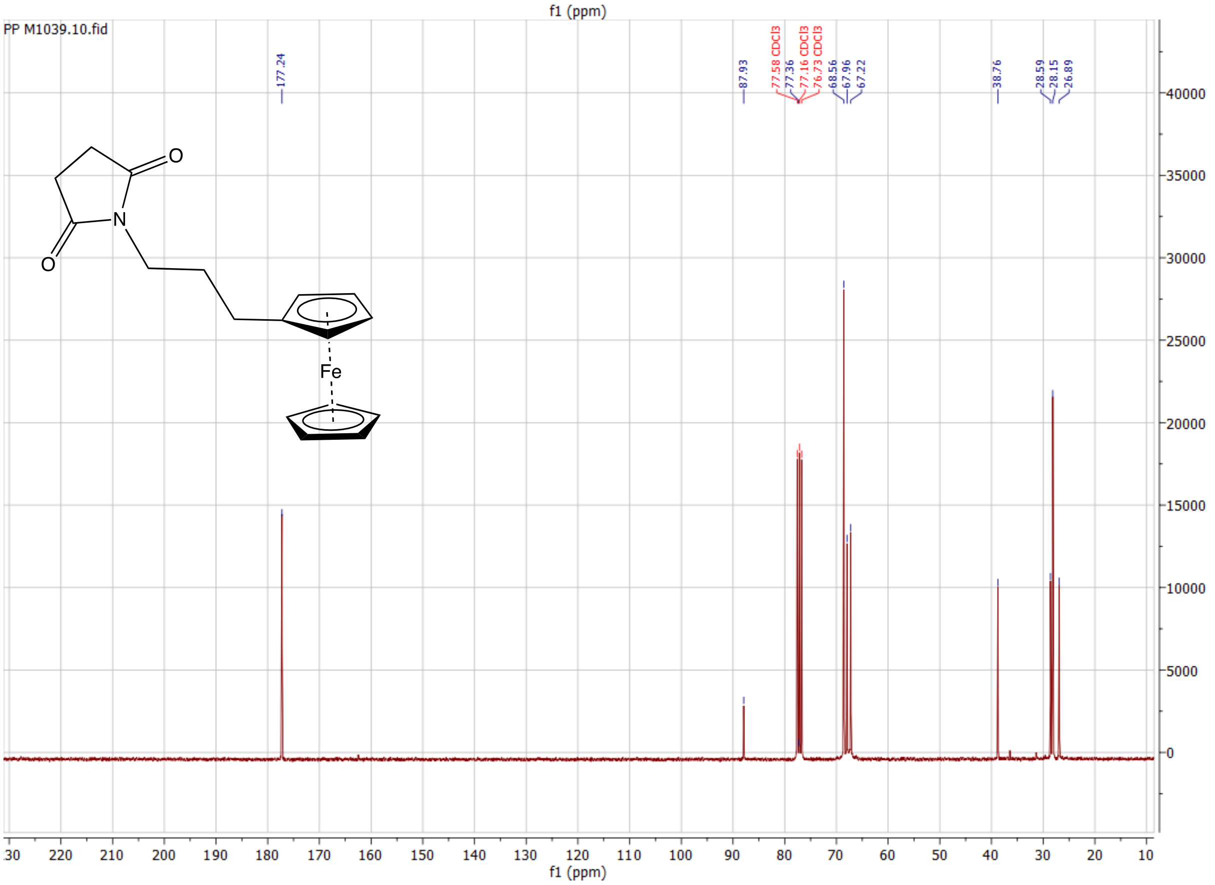

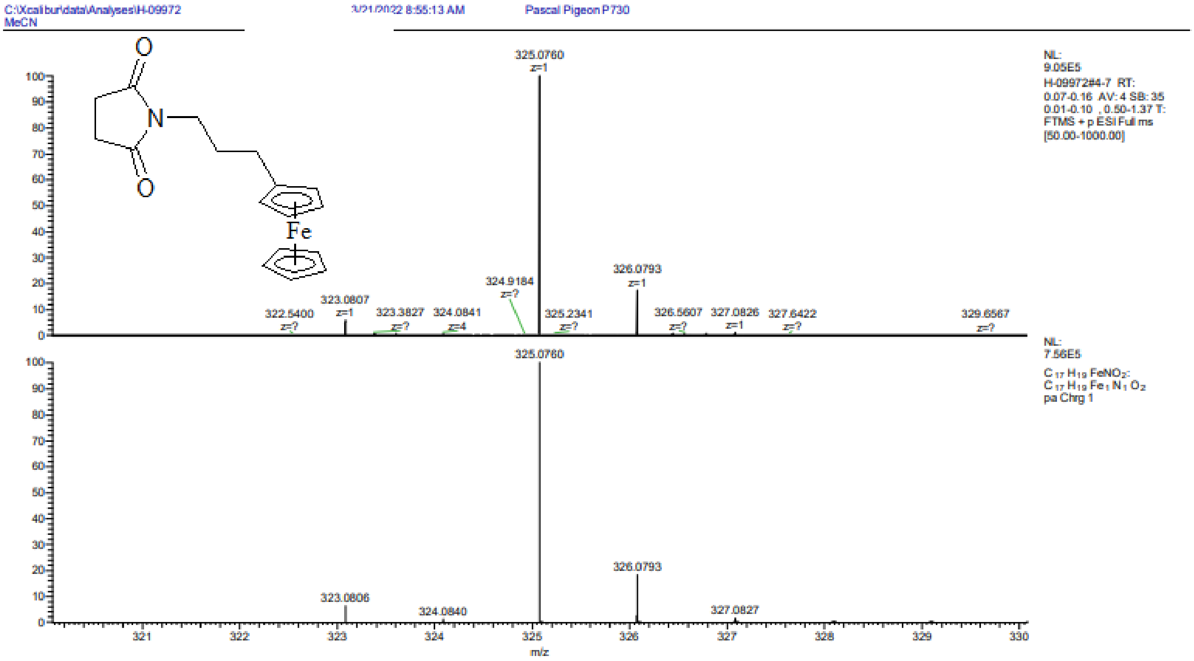

Experimental/theoretical isotopic pattern MS spectrum

Error = 0.1 ppm; Relative Intensity (%) 100

HRMS (ESI) m/z: [M]+ Calcd for C17H19FeNO2 325.0760 . Found 325.076; (Error: 0.1 ppm).

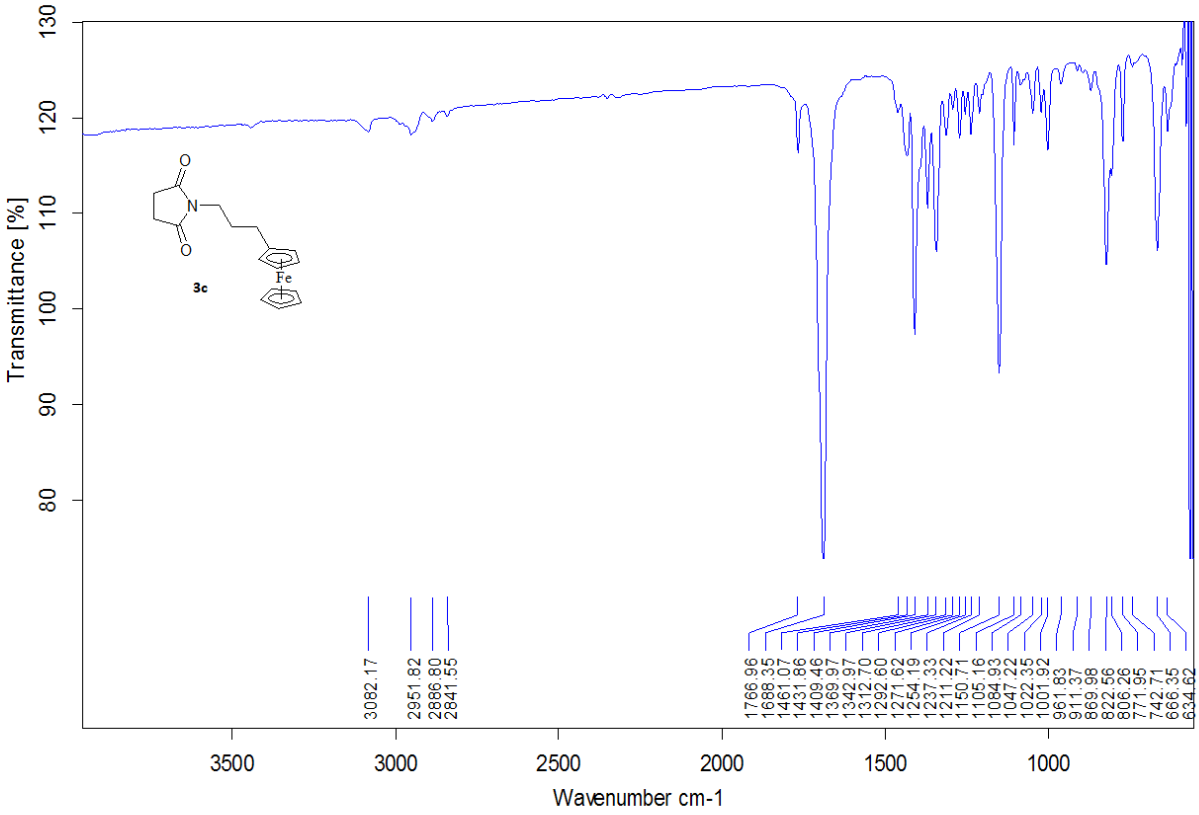

Figure S4: HR-MS and IR data for compound **3d**

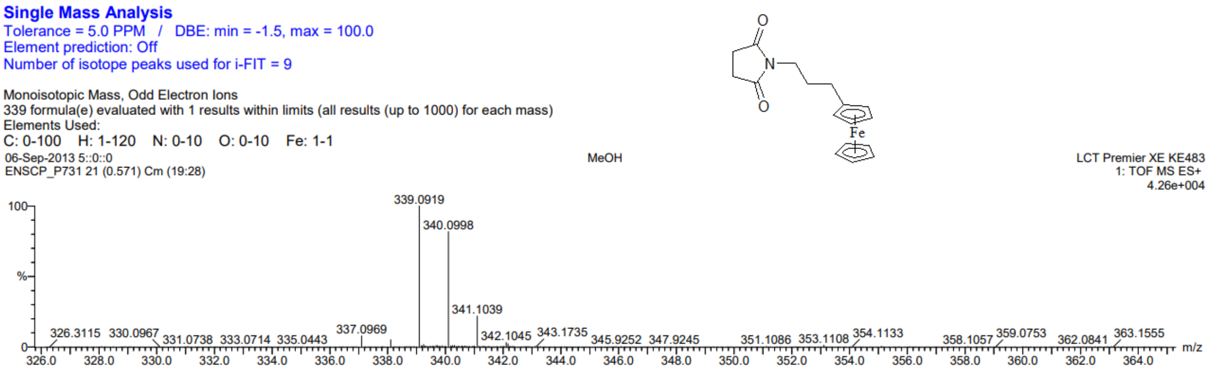

Minimum:				-1.5			
Maximum:		5.0	5.0	100.0			
Mass	Calc. Mass	mDa	PPM	DBE	i-FIT	i-FIT (Norm)	Formula
339.0919	339.0922	-0.3	-0.9	9.0	1253.4	0.0	C18 H21 N O2 Fe

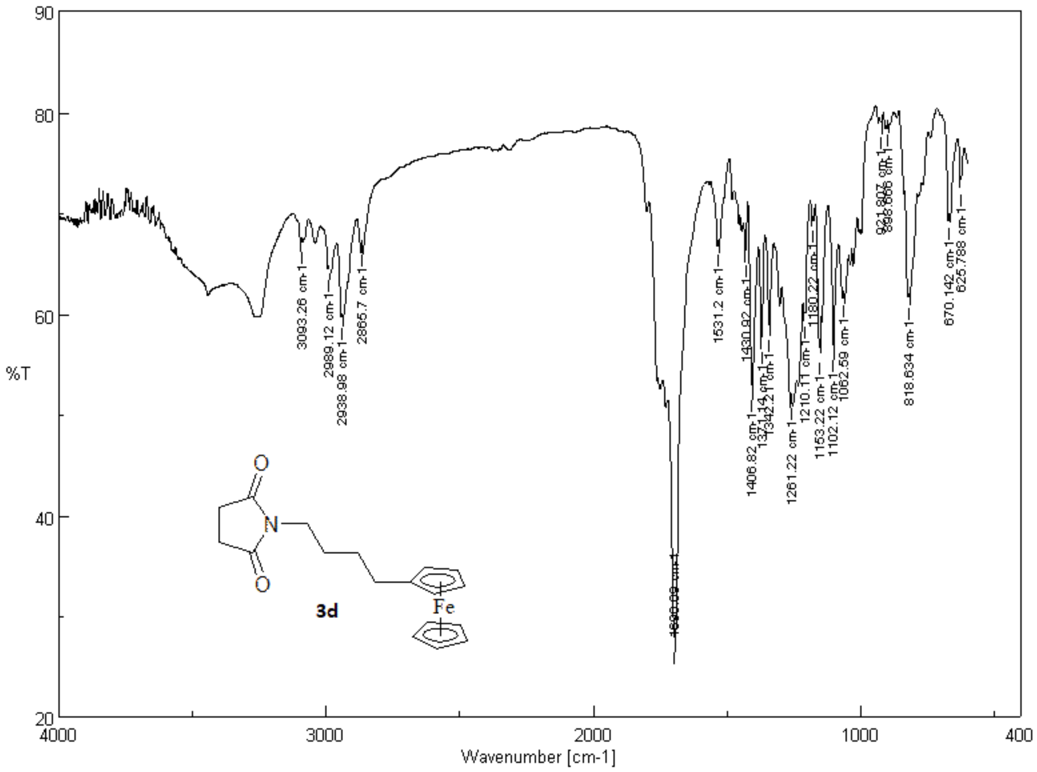

Figure S5: ^{1}H (in $CDCl_3$) ,^{13}C (in $CDCl_3$) NMR and IR data for compound **3e**

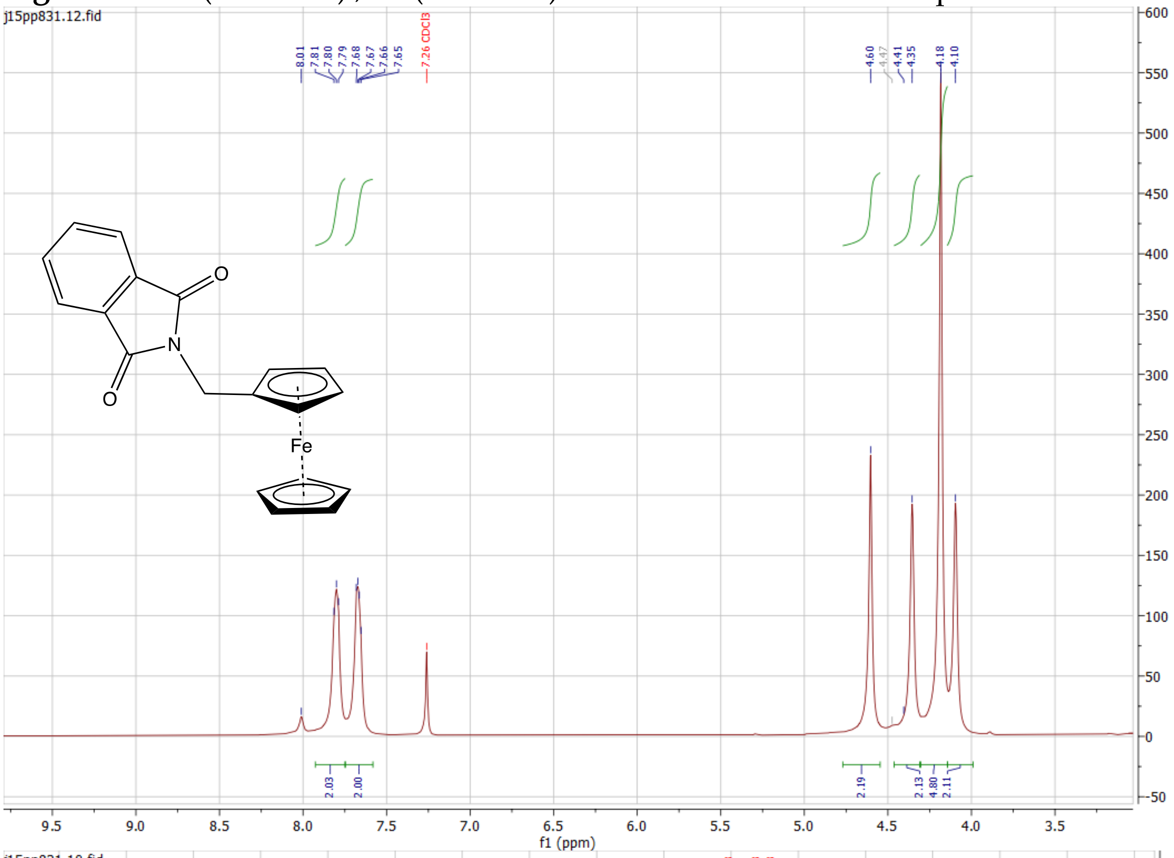

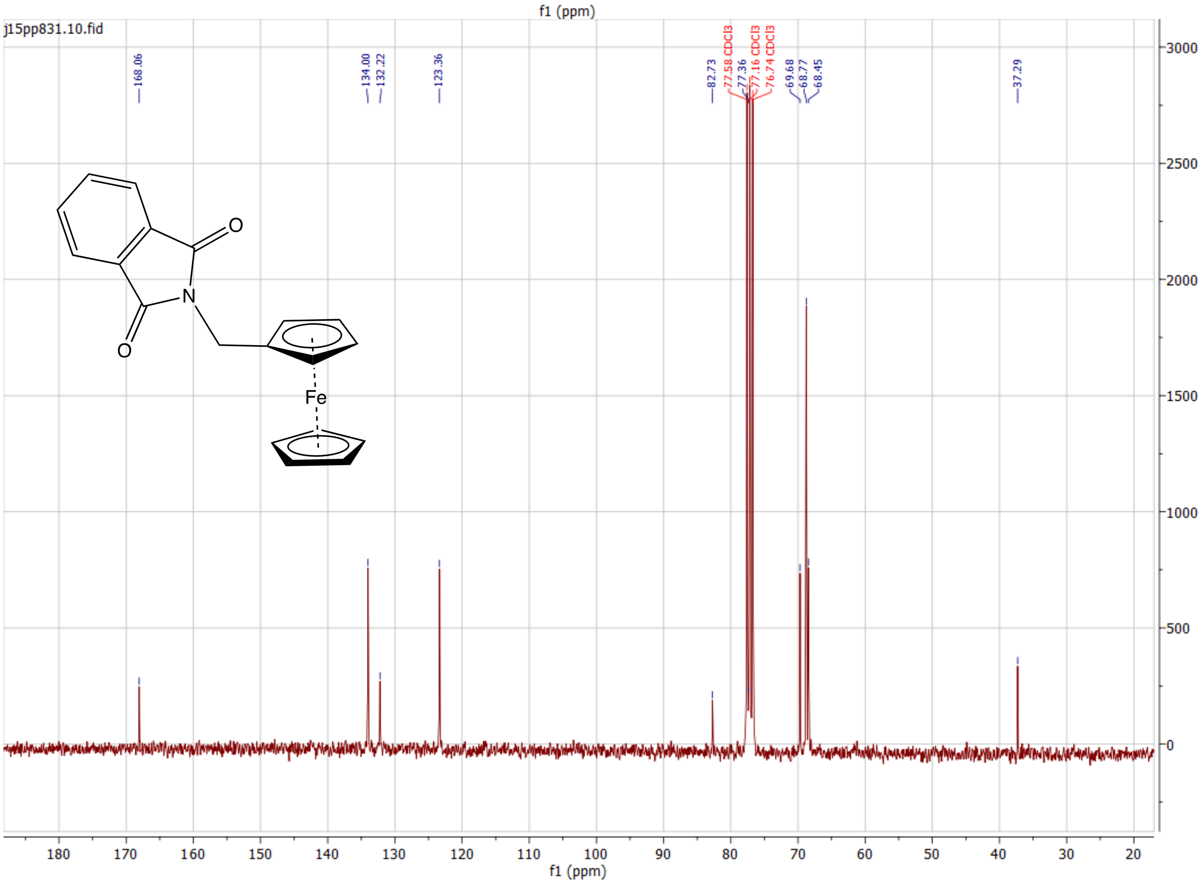

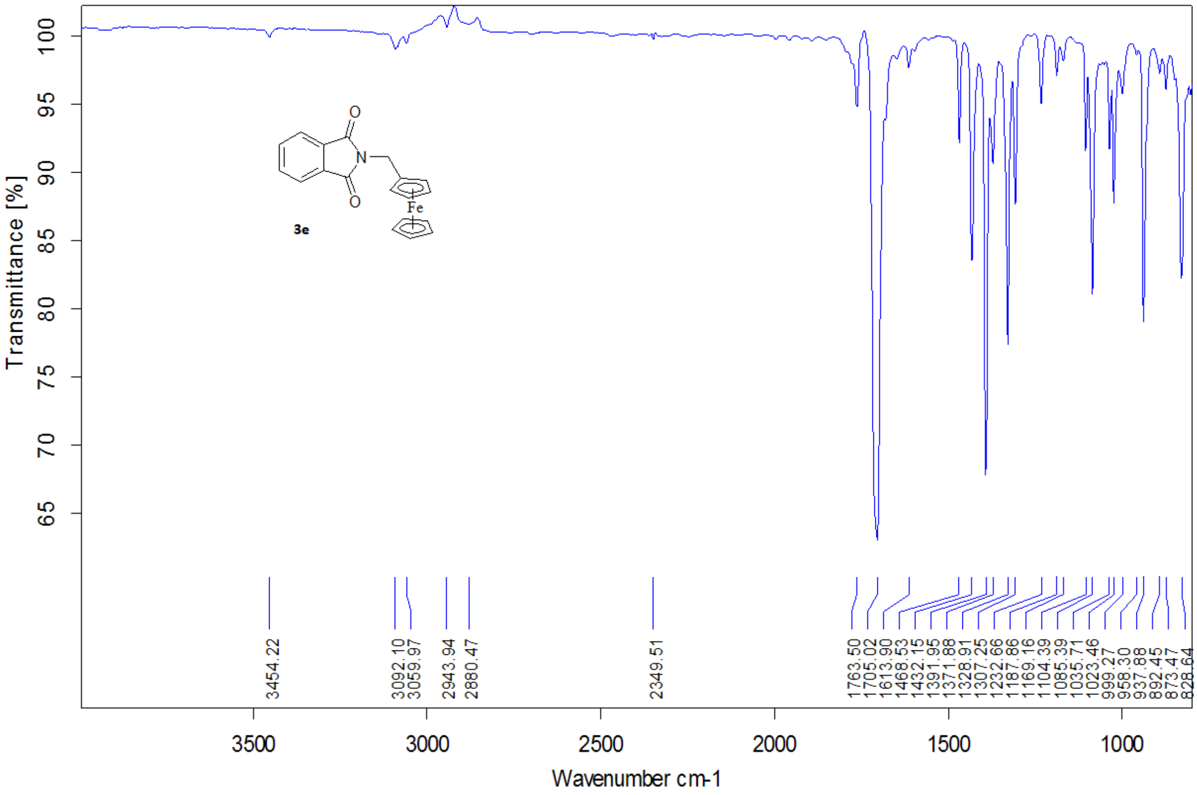

Transmittance [%]
Wavenumber cm-1
3e
Fe
3454.22
3092.10
3059.97
2943.94
2880.47
2349.51
1763.50
1705.02
1613.90
1468.53
1432.15
1391.95
1371.88
1328.91
1307.25
1232.66
1187.86
1169.16
1104.39
1085.39
1035.71
1023.46
999.27
958.30
937.88
892.45
873.47
828.64

Figure S6: ^{1}H (in $CDCl_3$), ^{13}C (in $CDCl_3$) NMR HR-MS and IR for compound **3f**

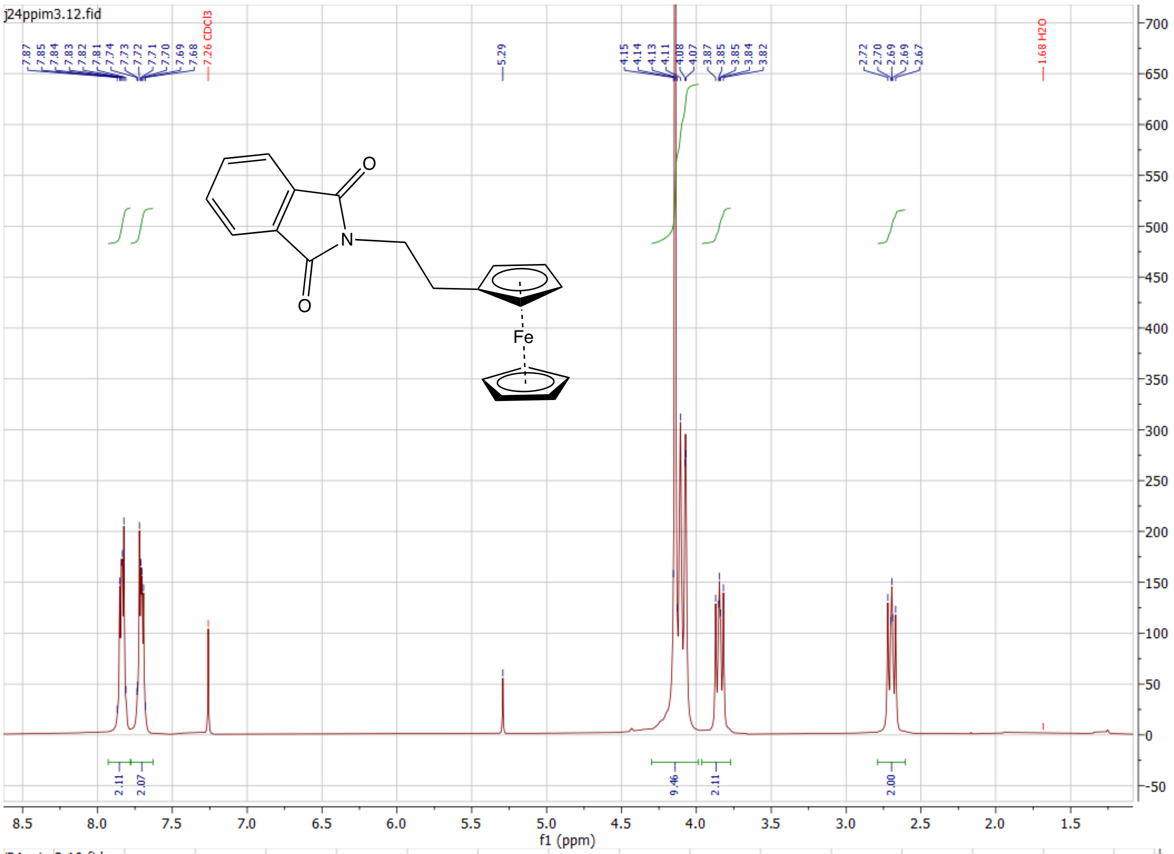

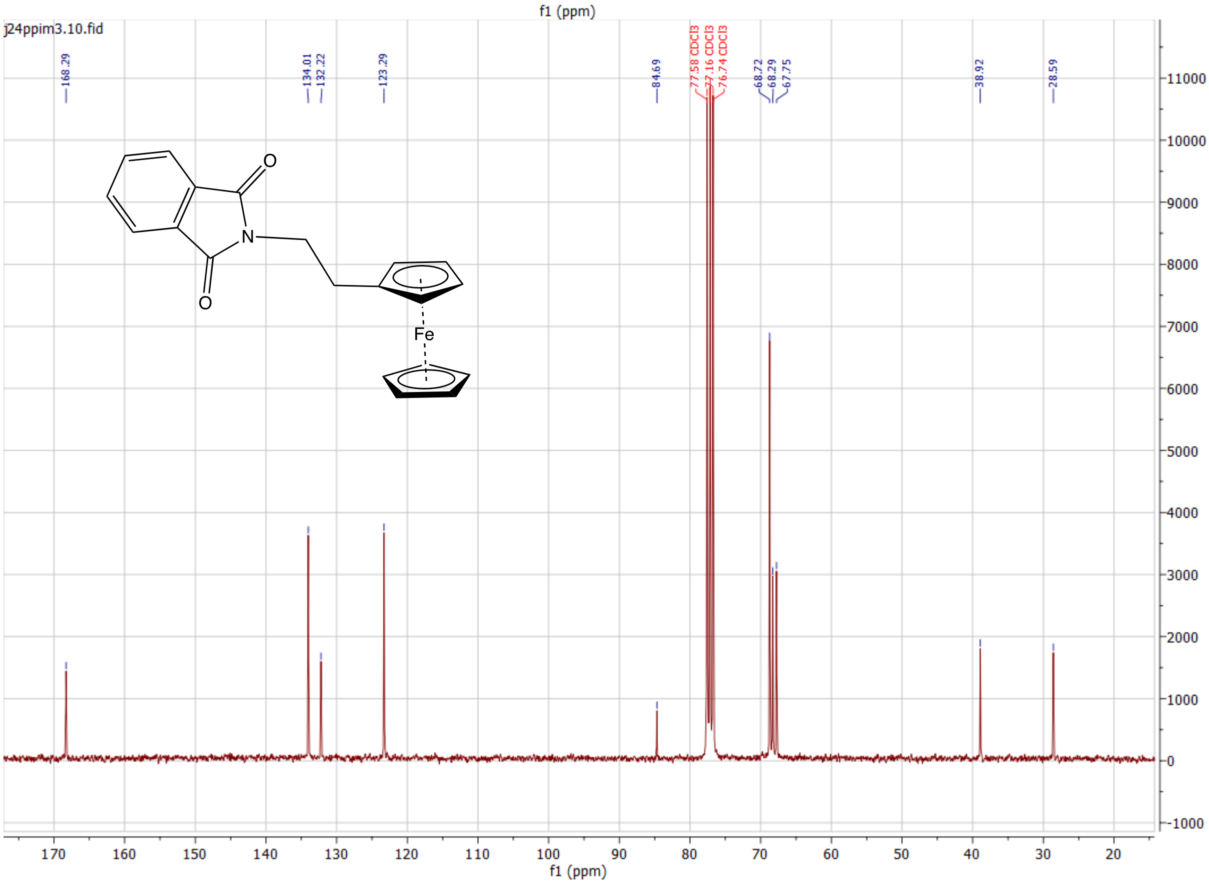

Single Mass Analysis
Tolerance = 5.0 PPM / DBE: min = -1.5, max = 100.0
Element prediction: Off
Number of isotope peaks used for i-FIT = 9

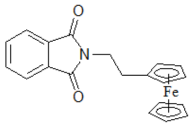

Monoisotopic Mass, Odd Electron Ions
365 formula(e) evaluated with 2 results within limits (all results (up to 1000) for each mass)
Elements Used:
C: 0-100 H: 1-120 N: 0-10 O: 0-10 Fe: 1-1

06-Sep-2013 5::9::2 MeOH LCT Premier XE KE483
ENSCP_P654 28 (0.714) Cm (28:39) 1: TOF MS ES+ 2.02e+004

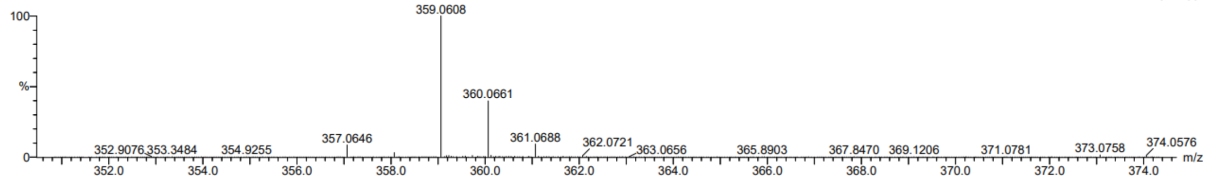

Minimum:				-1.5			
Maximum:		5.0	5.0	100.0			
Mass	Calc. Mass	mDa	PPM	DBE	i-FIT	i-FIT (Norm)	Formula
359.0608	359.0609	-0.1	-0.3	13.0	920.5	0.0	C20 H17 N O2 Fe
	359.0600	0.8	2.2	1.0	930.3	9.7	C4 H17 N9 O7 Fe

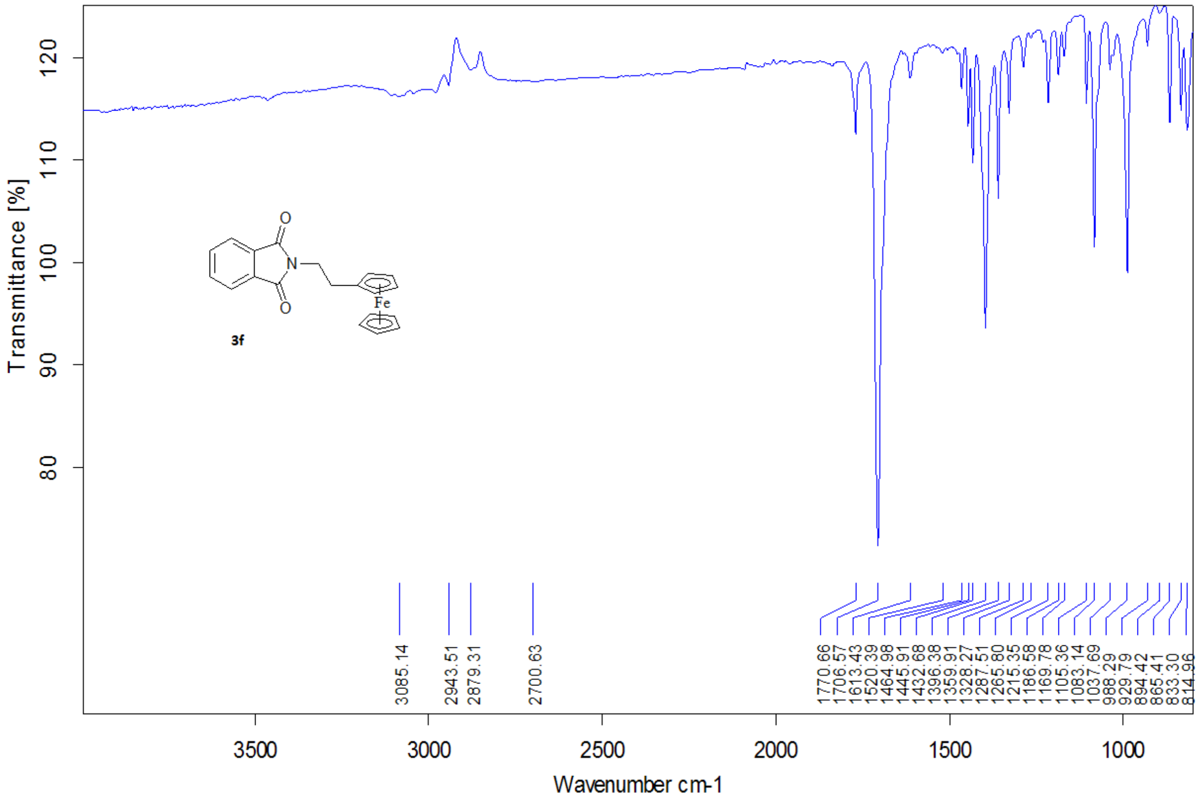

Figure S7: ^{1}H (in CDCl3), ^{13}C (in CDCl3) NMR, HR-MS and IR data for compound **3g**

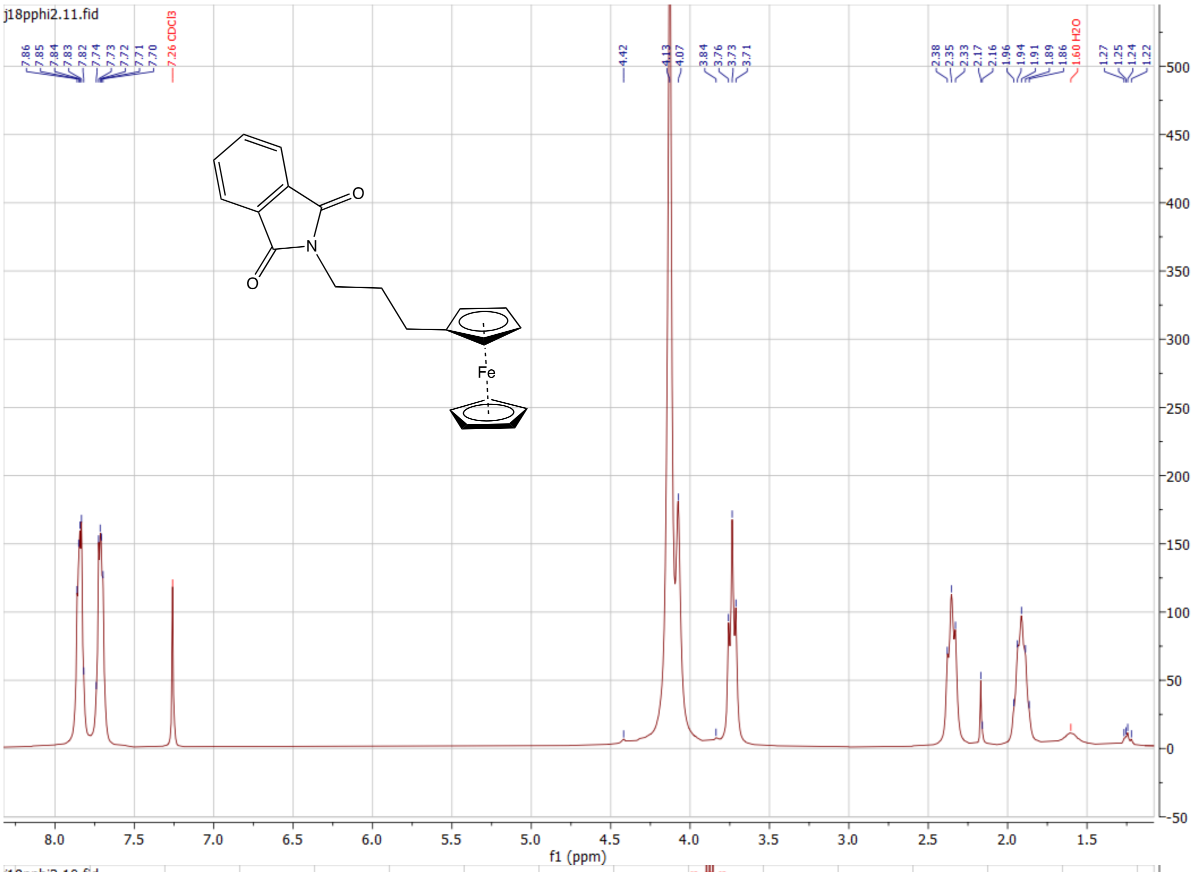

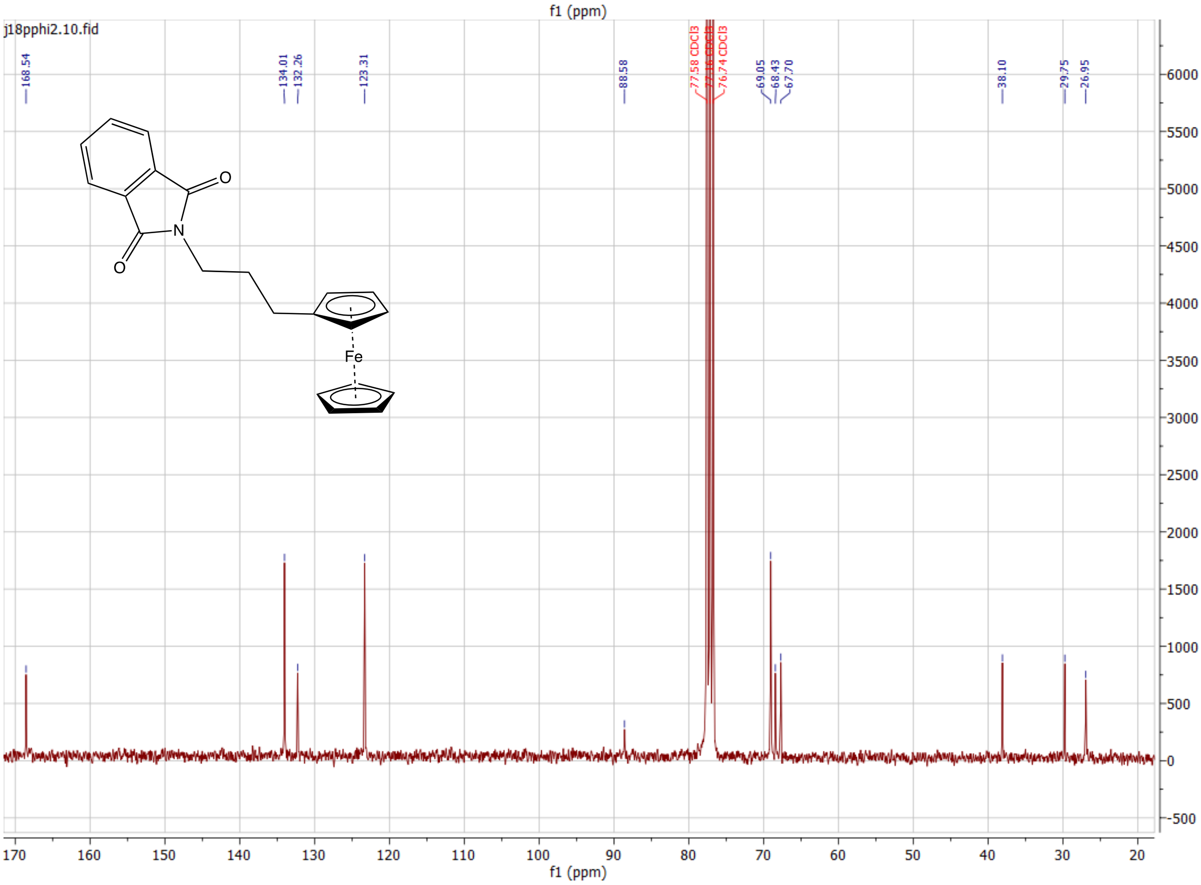

Single Mass Analysis
Tolerance = 5.0 PPM / DBE: min = -1.5, max = 100.0
Element prediction: Off
Number of isotope peaks used for i-FIT = 9

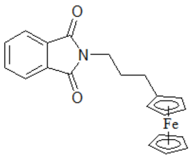

Monoisotopic Mass, Odd Electron Ions
156 formula(e) evaluated with 2 results within limits (all results (up to 1000) for each mass)
Elements Used:
C: 1-150 H: 1-150 N: 0-3 O: 0-10 Fe: 1-1

19-Jun-2013 3::2::0
ENSCP_P659 57 (1.384) Cm (55:60)

ACN

LCT Premier XE KE483
1: TOF MS ES+
1.96e+003

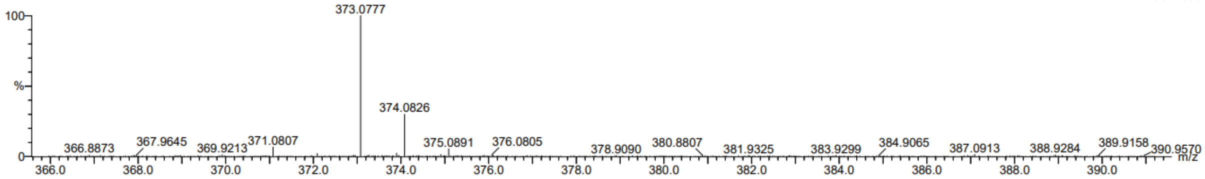

Minimum:				-1.5			
Maximum:		5.0	5.0	100.0			
Mass	Calc. Mass	mDa	PPM	DBE	i-FIT	i-FIT (Norm)	Formula
373.0777	373.0765	1.2	3.2	13.0	457.1	0.0	C21 H19 N O2 Fe
	373.0784	-0.7	-1.9	0.0	463.7	6.6	C9 H23 N3 O9 Fe

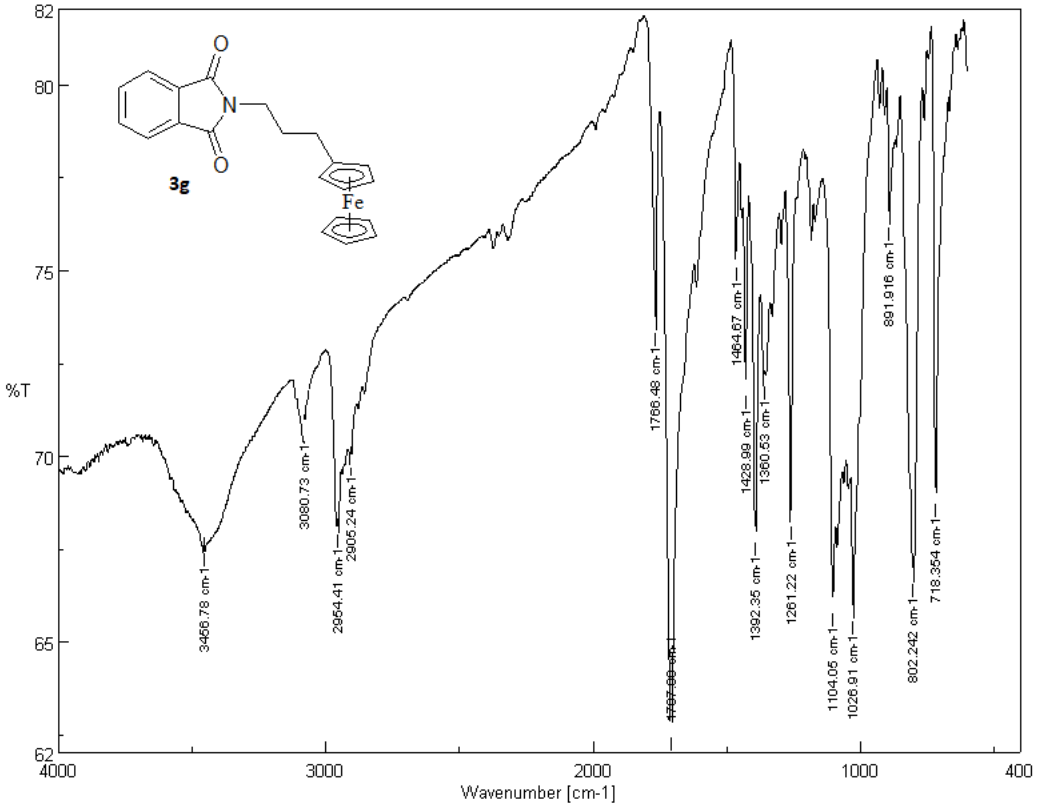

Figure S8: ^{1}H (in acetone-d_6), ^{13}C (in $CDCl_3$) NMR, HR-MS and IR data for compound **3h**

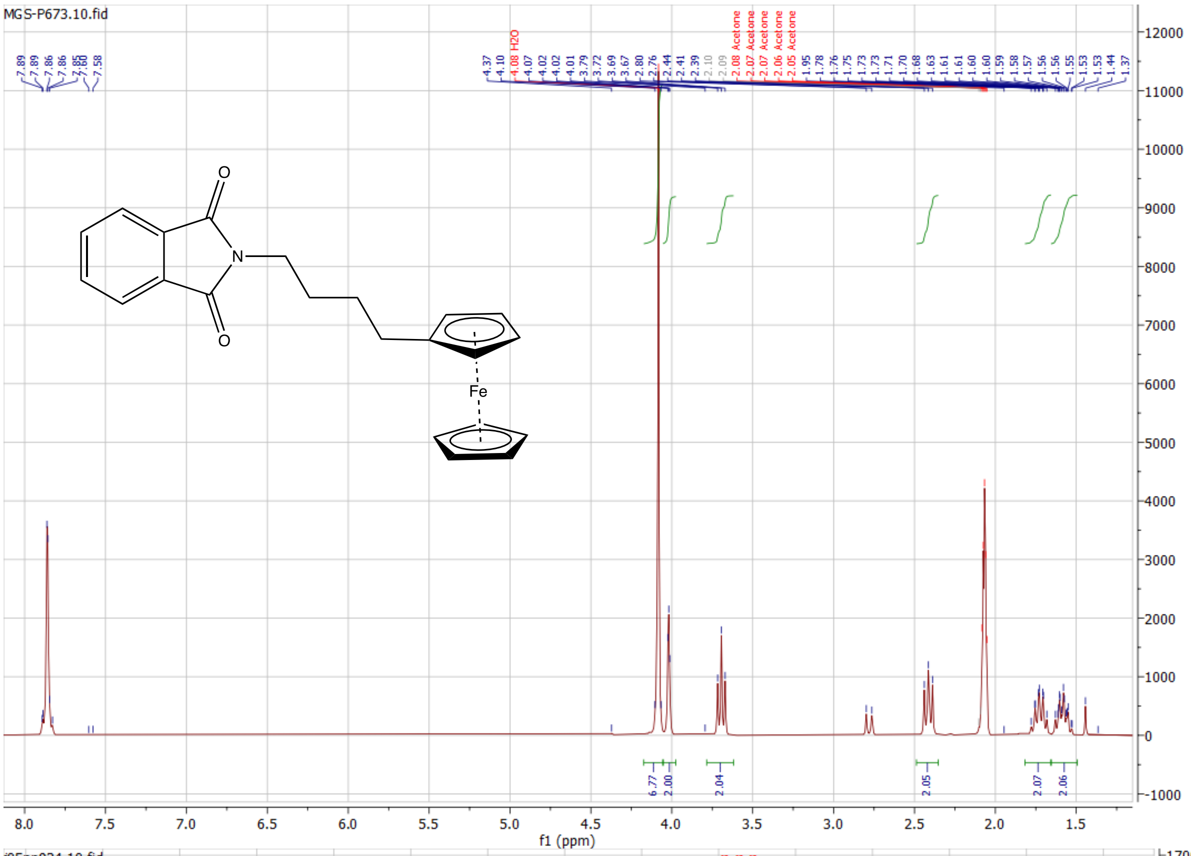

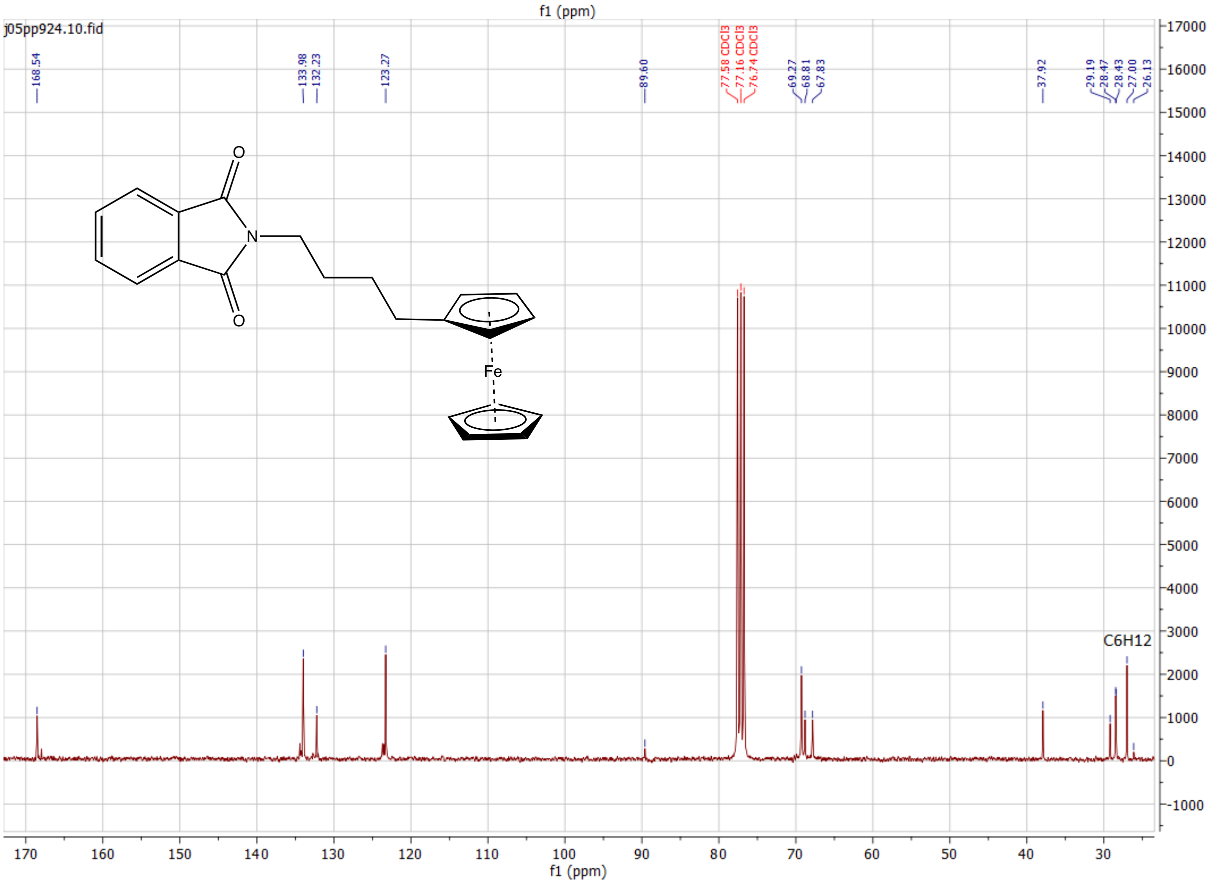

Single Mass Analysis
Tolerance = 5.0 PPM / DBE: min = -1.5, max = 100.0
Element prediction: Off
Number of isotope peaks used for i-FIT = 9

Monoisotopic Mass, Odd Electron Ions
164 formula(e) evaluated with 1 results within limits (all results (up to 1000) for each mass)
Elements Used:
C: 1-150 H: 1-150 N: 0-3 O: 0-10 Fe: 1-1

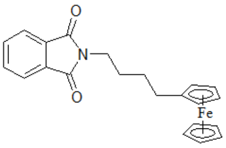

19-Jun-2013 2::4::4
ENSCP_P673 49 (1.204) Cm (47:54)
ACN
LCT Premier XE KE483
1: TOF MS ES+
1.84e+004

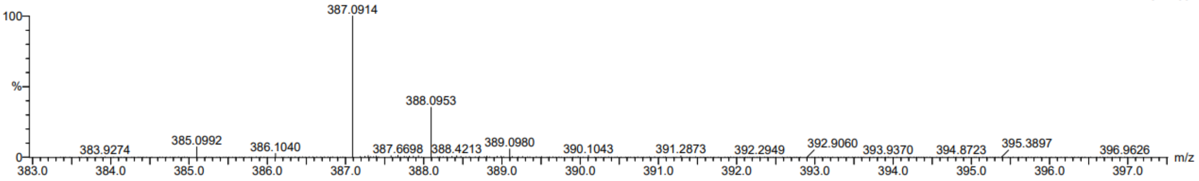

Minimum:				-1.5			
Maximum:		5.0	5.0	100.0			
Mass	Calc. Mass	mDa	PPM	DBE	i-FIT	i-FIT (Norm)	Formula
387.0914	387.0922	-0.8	-2.1	13.0	800.1	0.0	C22 H21 N O2 Fe

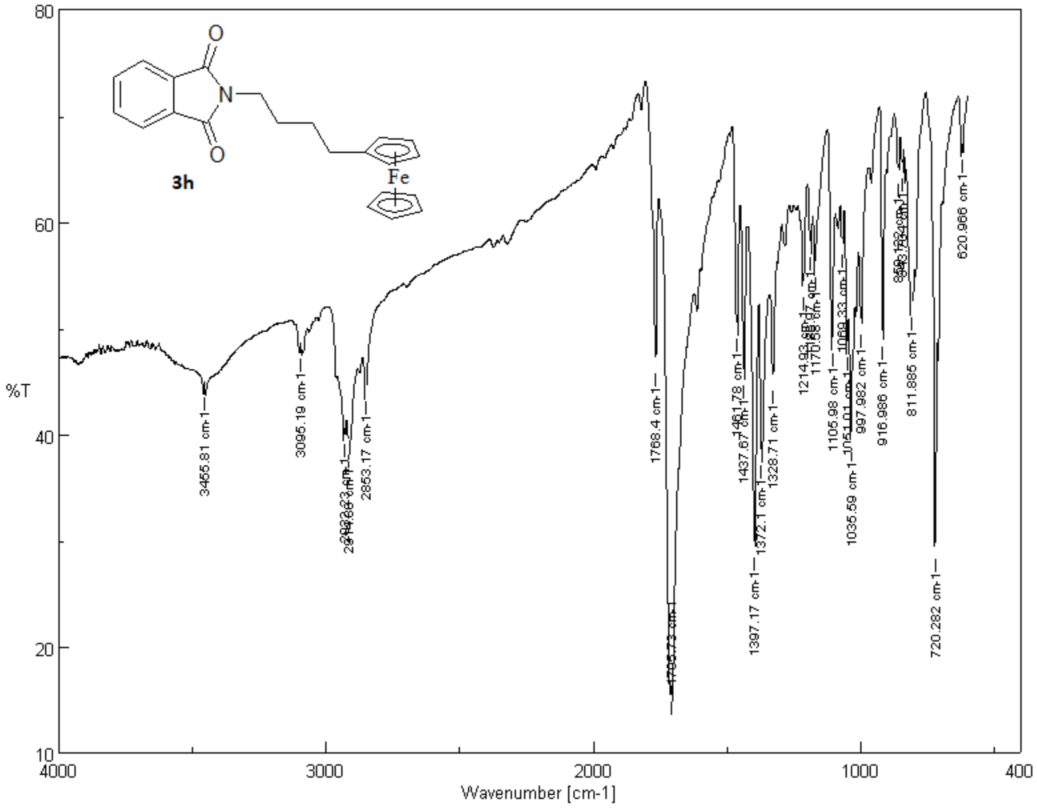

Figure S9: ^{1}H (in $CDCl_3$), ^{13}C (in DMSO-d6) NMR, HR-MS and IR data for compound **3i**

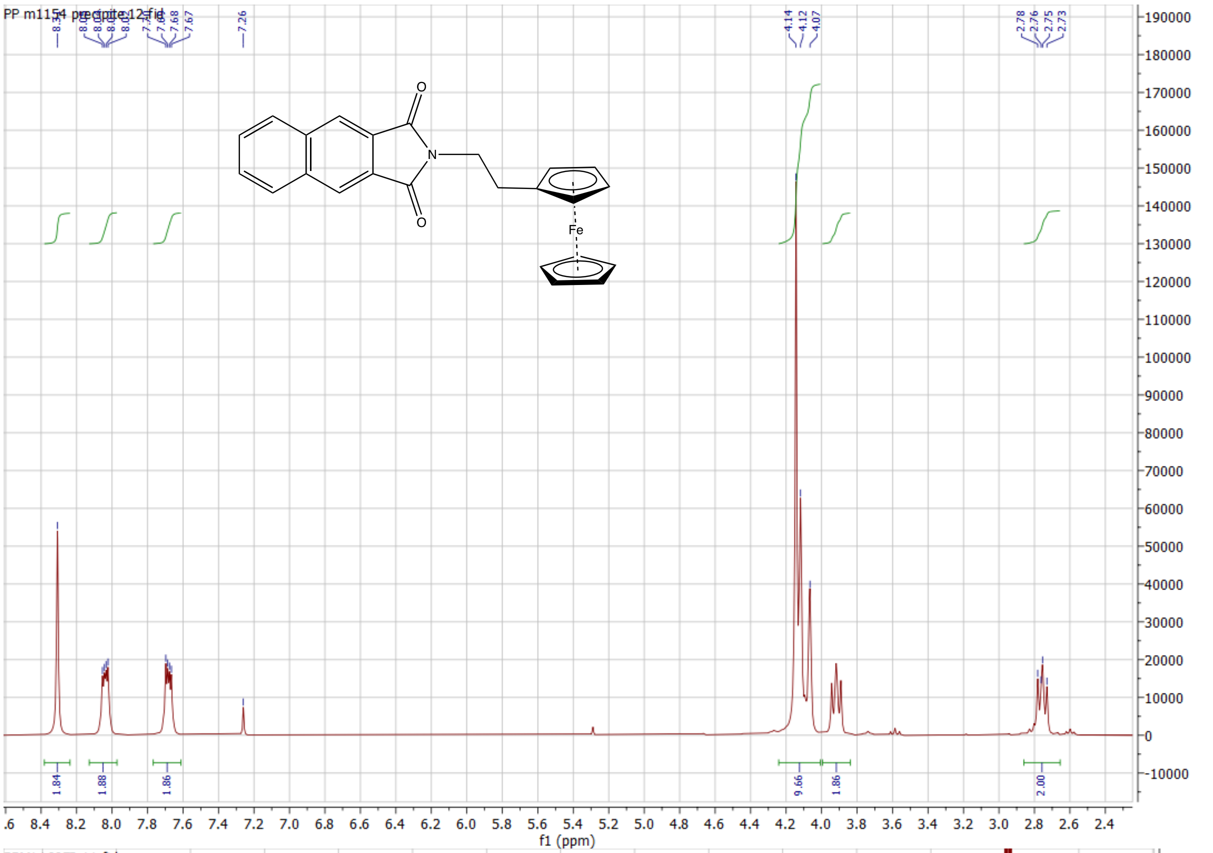

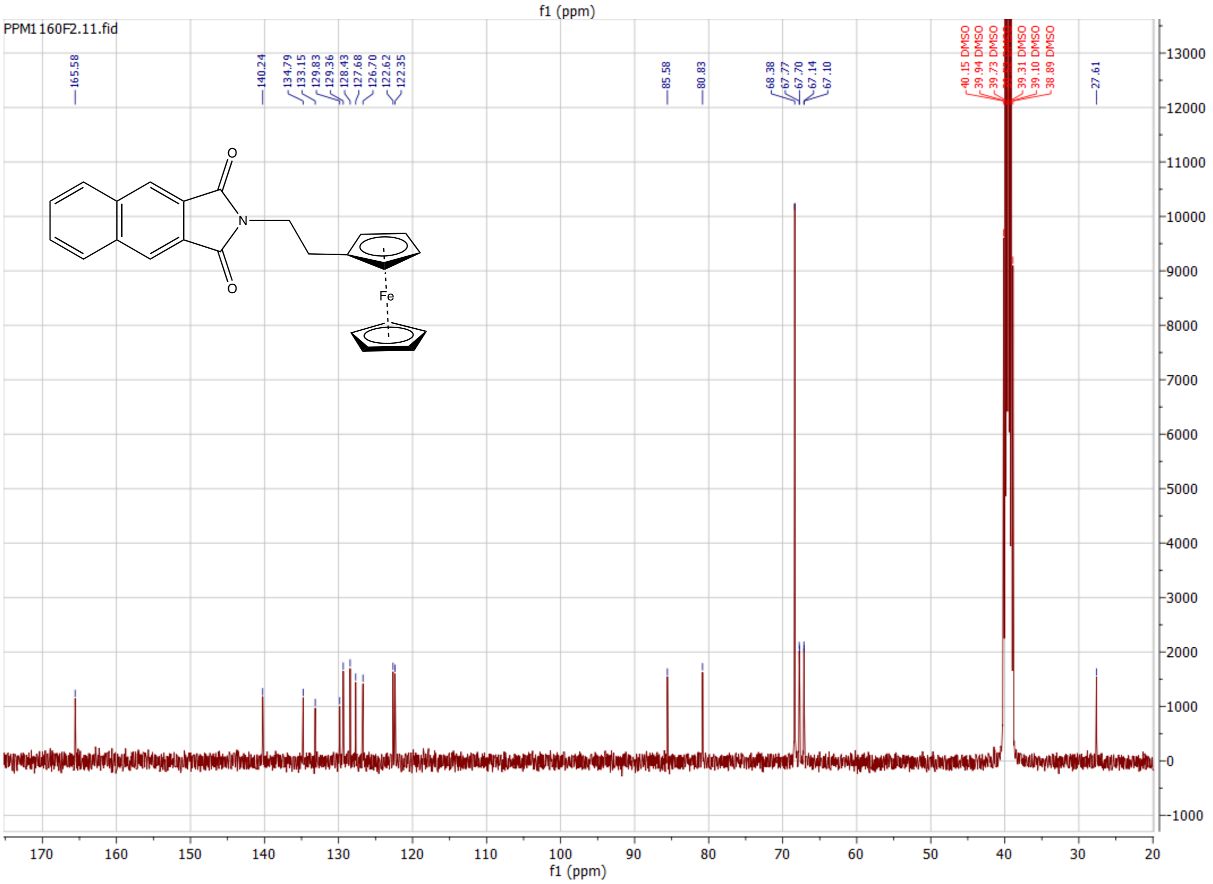

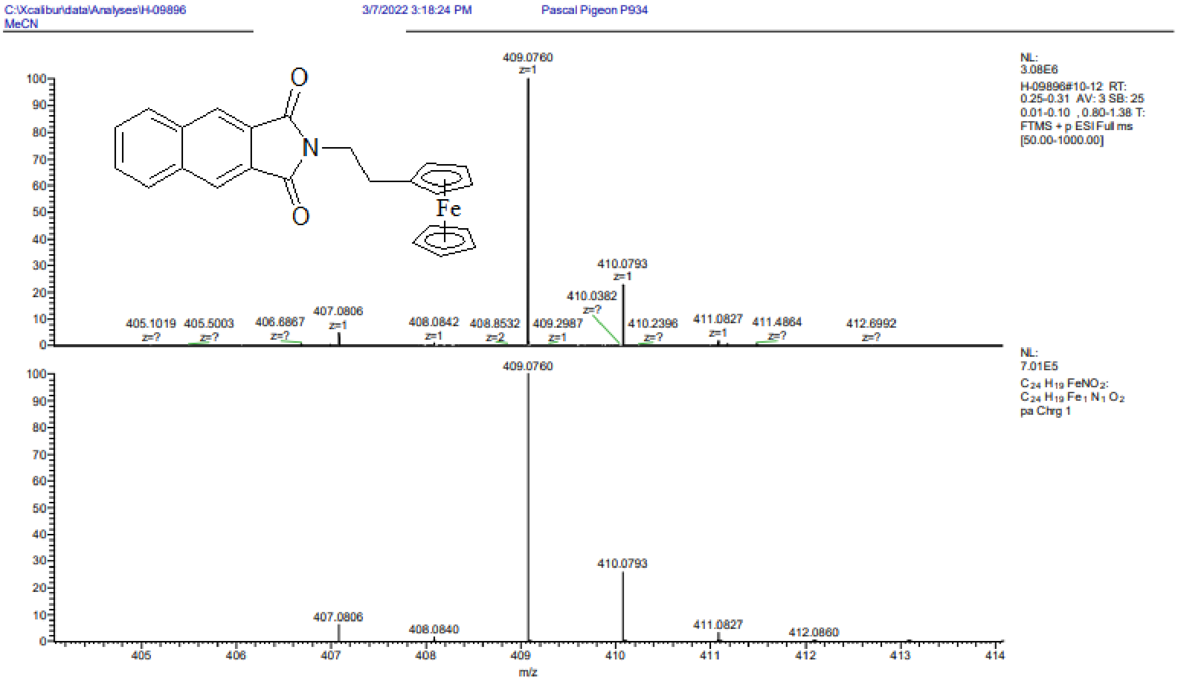

Experimental/theoretical isotopic pattern MS spectrum

Error = 0.1 ppm; Relative Intensity (%) 100

HRMS (ESI) m/z: [M]+ Calcd for C24H19FeNO2 409.0760 . Found 409.076; (Error: 0.1 ppm).

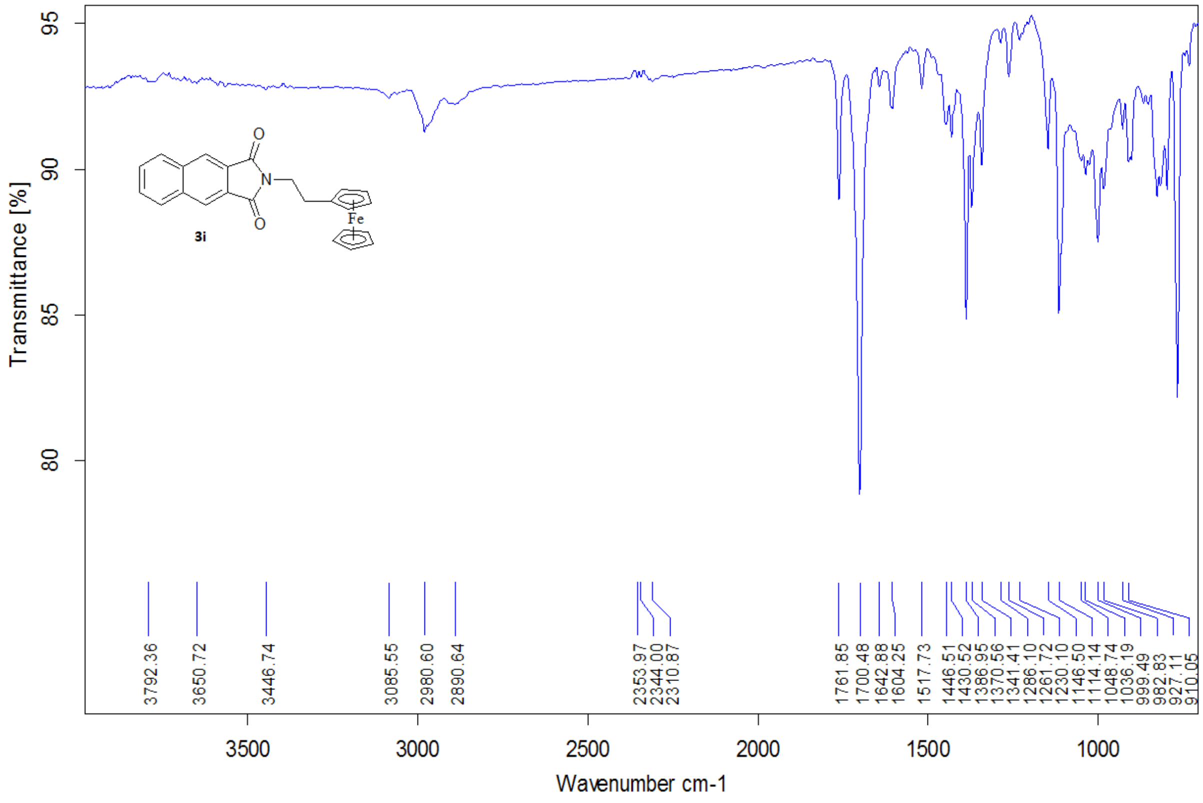

Figure S10: ^{1}H (in $CDCl_3$), ^{13}C (in $CDCl_3$) NMR, HR-MS and IR data for compound **3j**

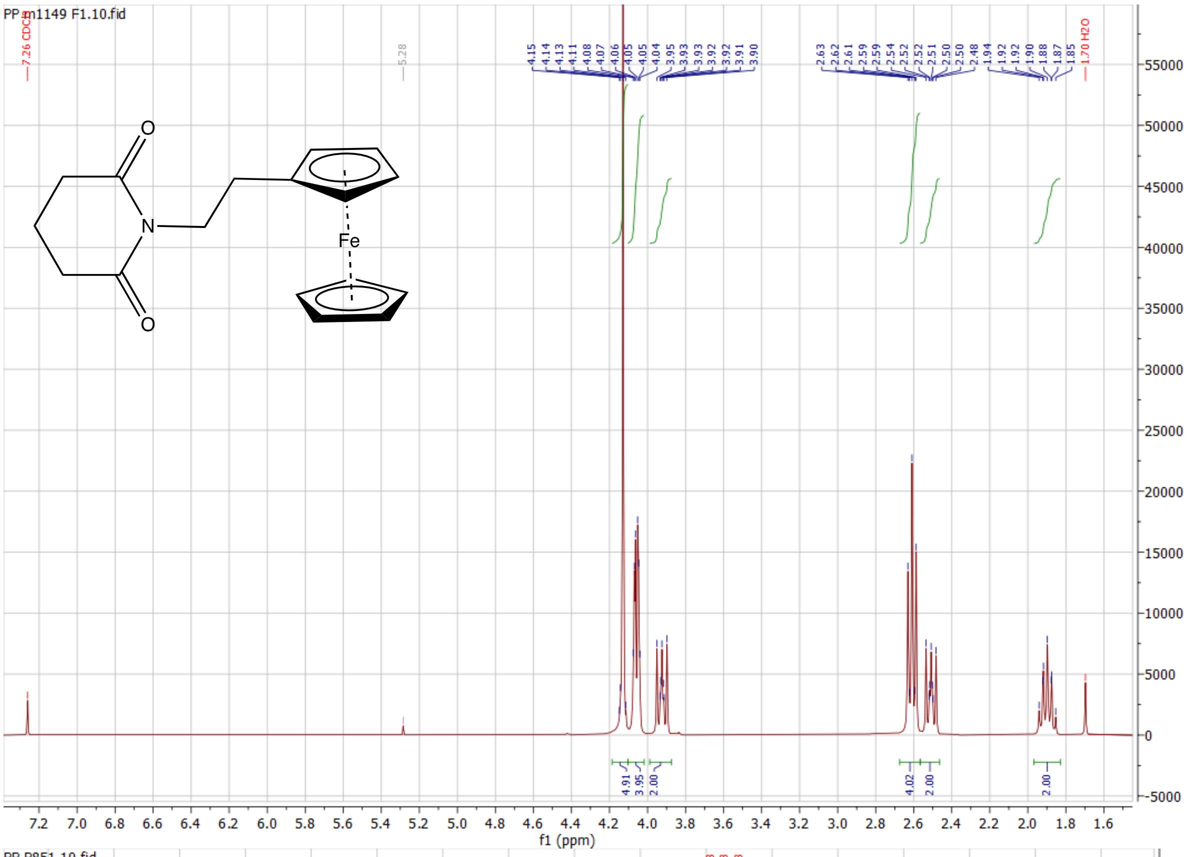

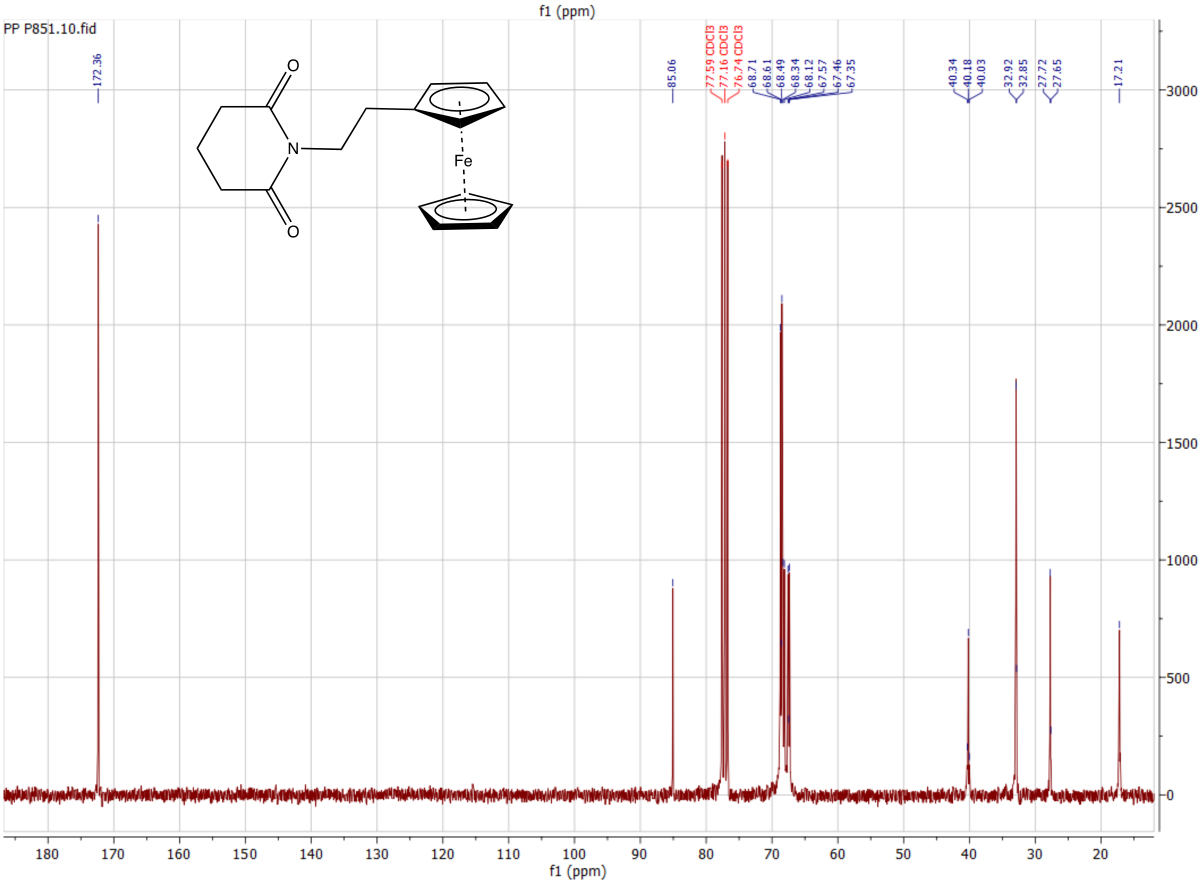

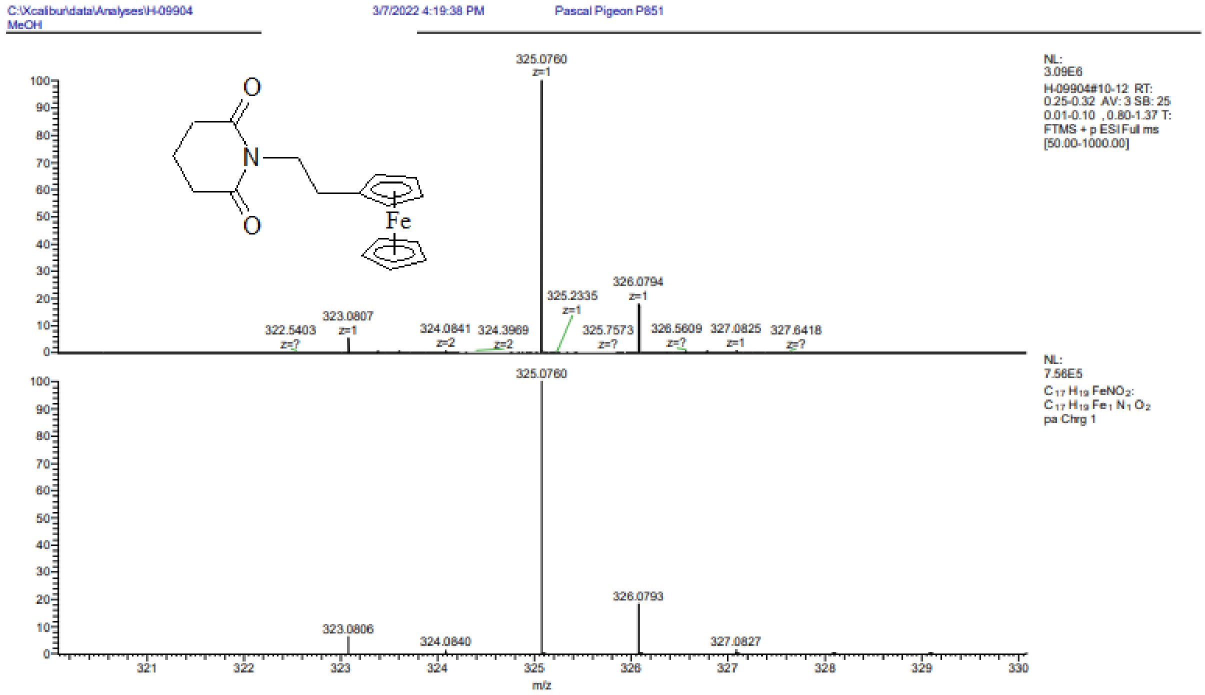

Experimental/theoretical isotopic pattern MS spectrum

Error = 0.1 ppm; Relative Intensity (%) 100

HRMS (ESI) m/z: [M]+ Calcd for C17H19FeNO2 325.0760 . Found 325.076; (Error: 0.1 ppm).

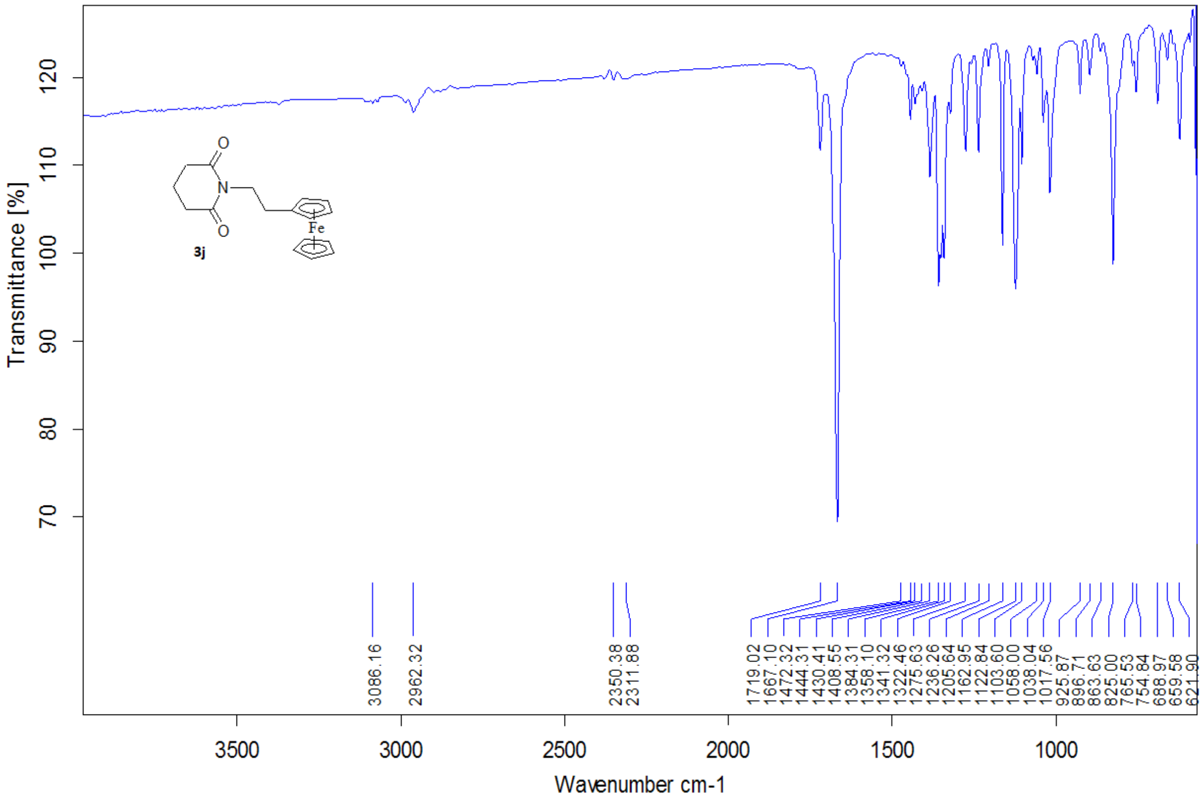

Figure S11: ^{1}H (in CDCl3), ^{13}C (in CDCl3) NMR, HR-MS and IR data for compound **3k**

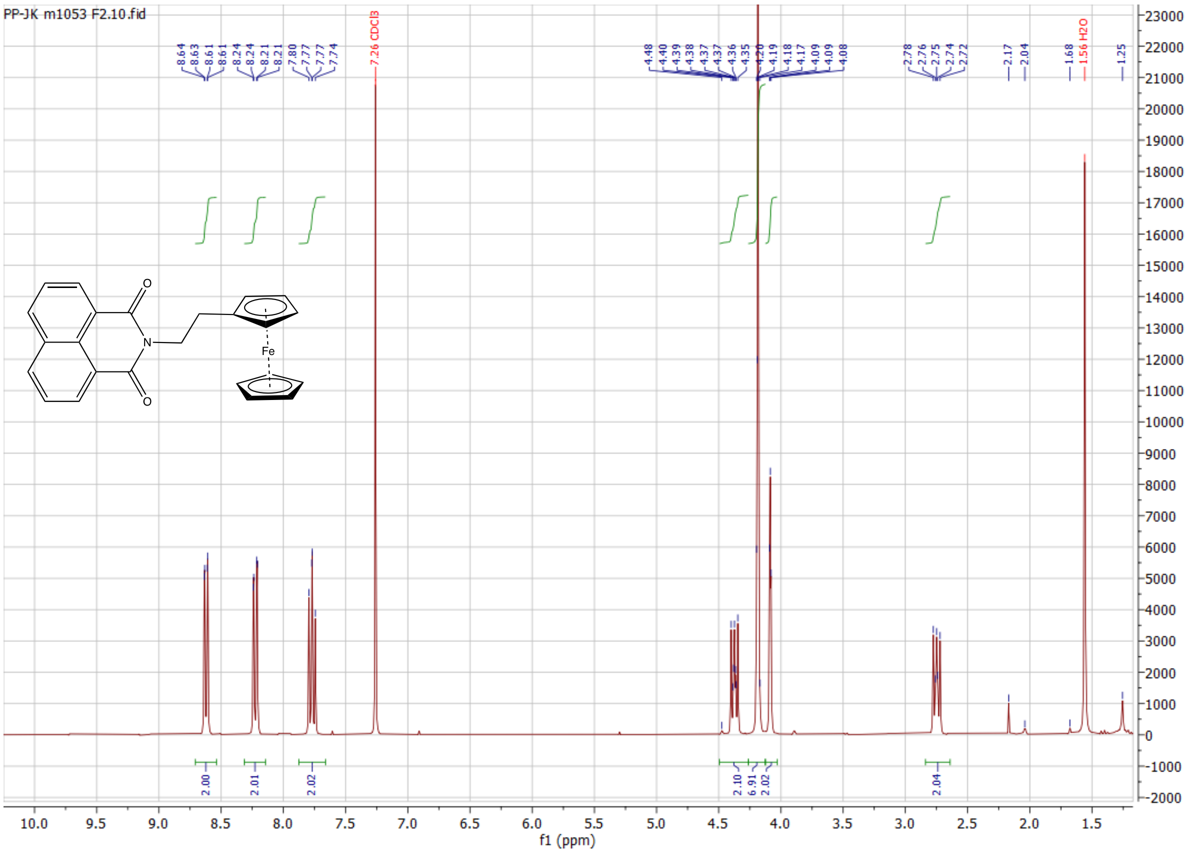

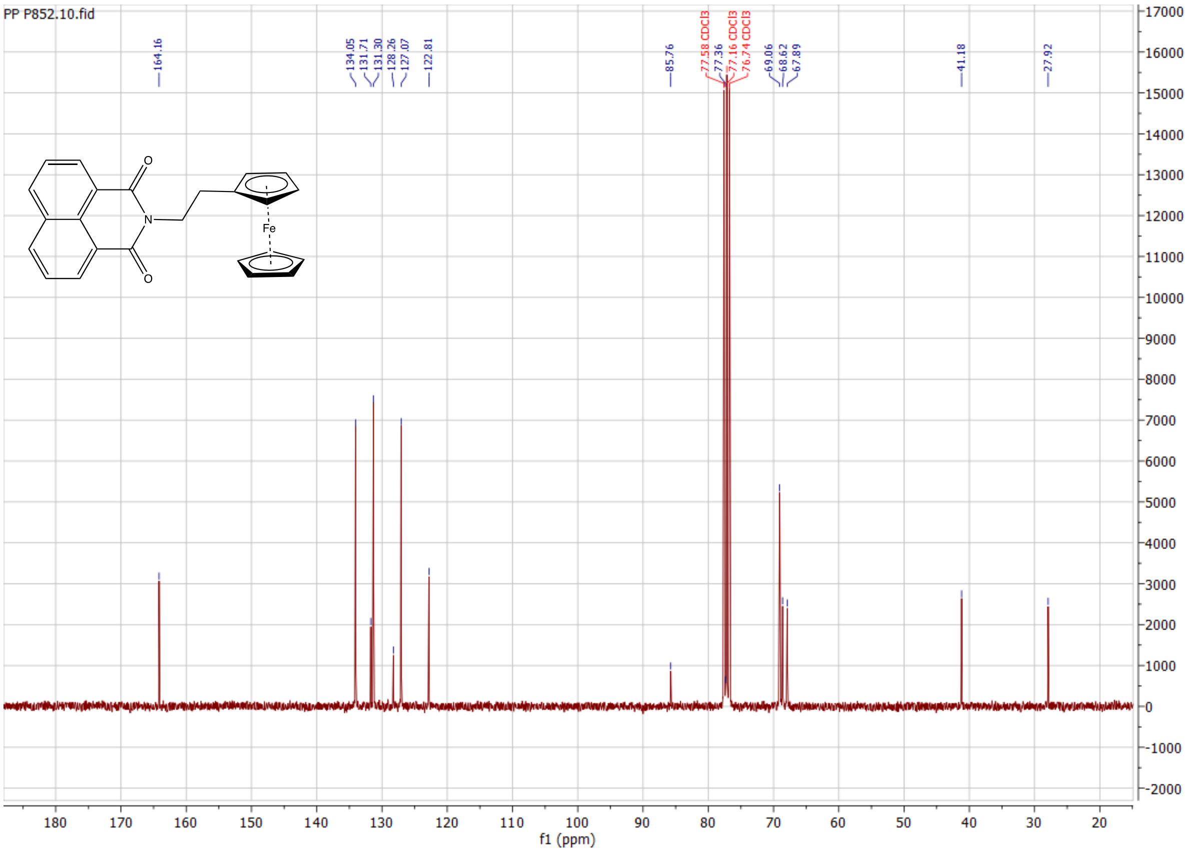

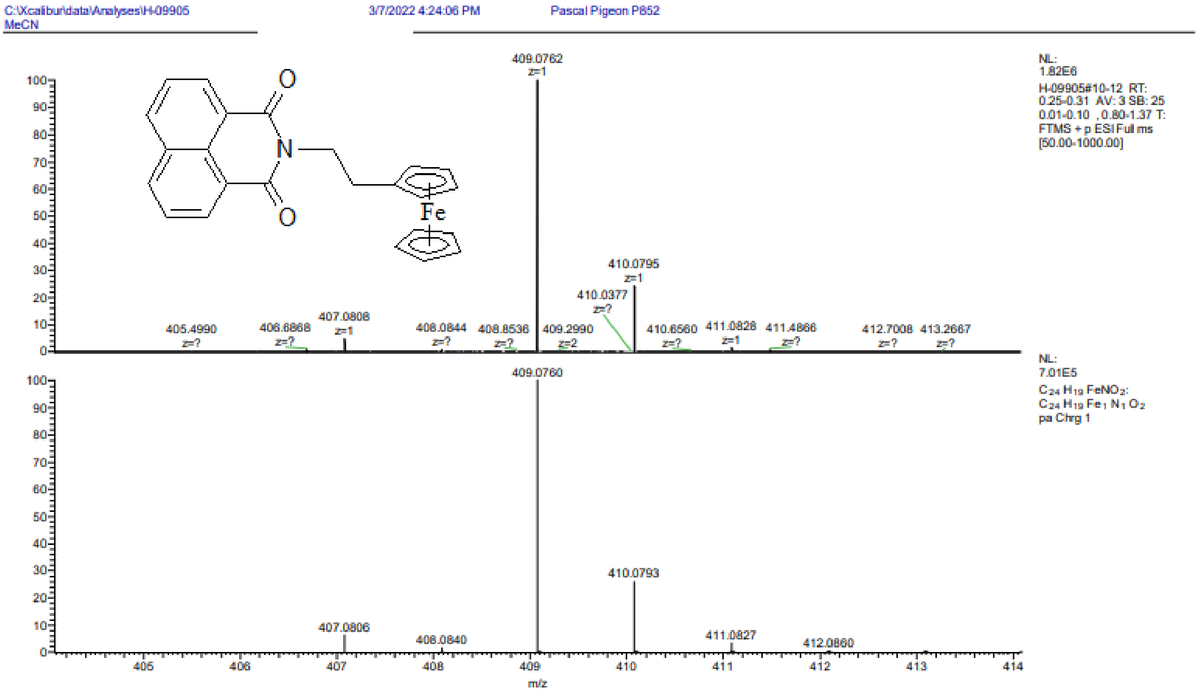

Experimental/theoretical isotopic pattern MS spectrum

Error = 0.6 ppm; Relative Intensity (%) 100

HRMS (ESI) m/z: [M]+ Calcd for C24H19FeNO2 409.0760 . Found 409.0762; (Error: 0.6 ppm).

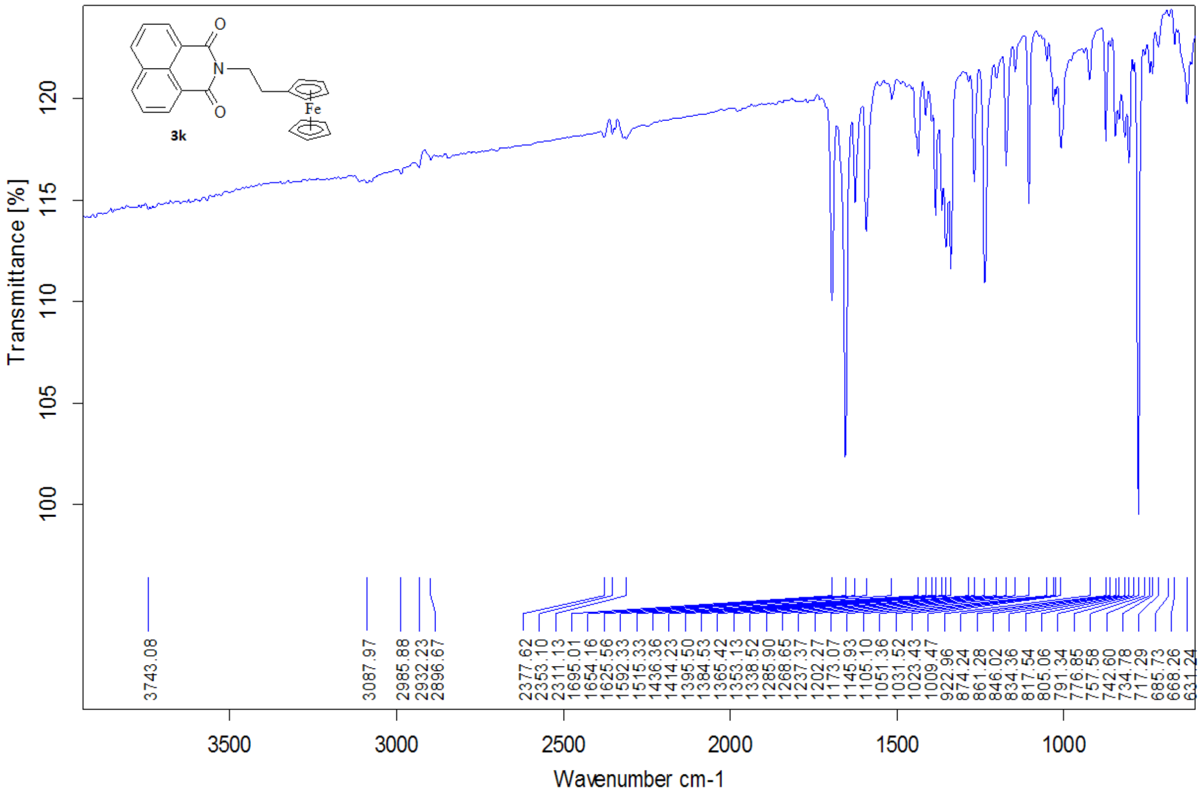

Figure S12: ^{1}H (in in $CDCl_3$),^{13}C (in in $CDCl_3$) NMR, HR-MS and IR data for compound **4b**

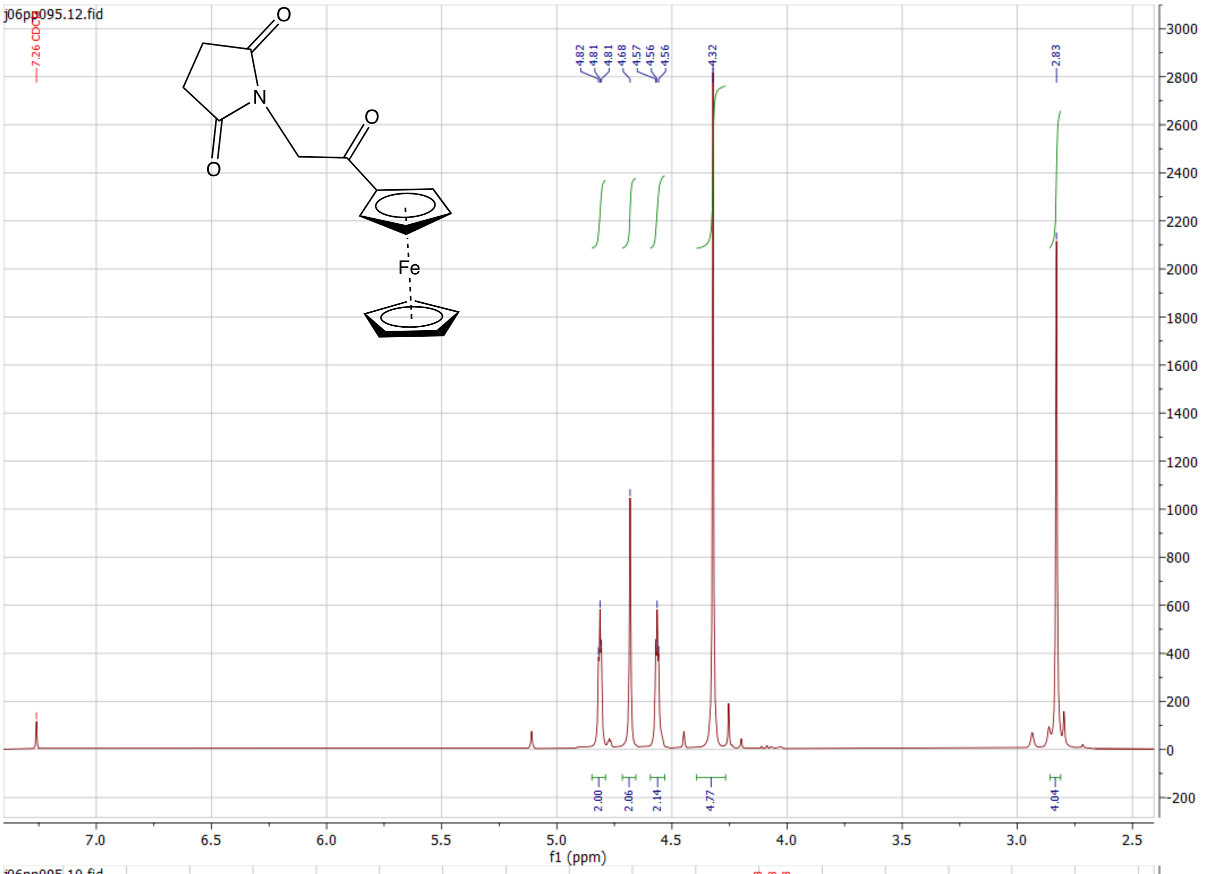

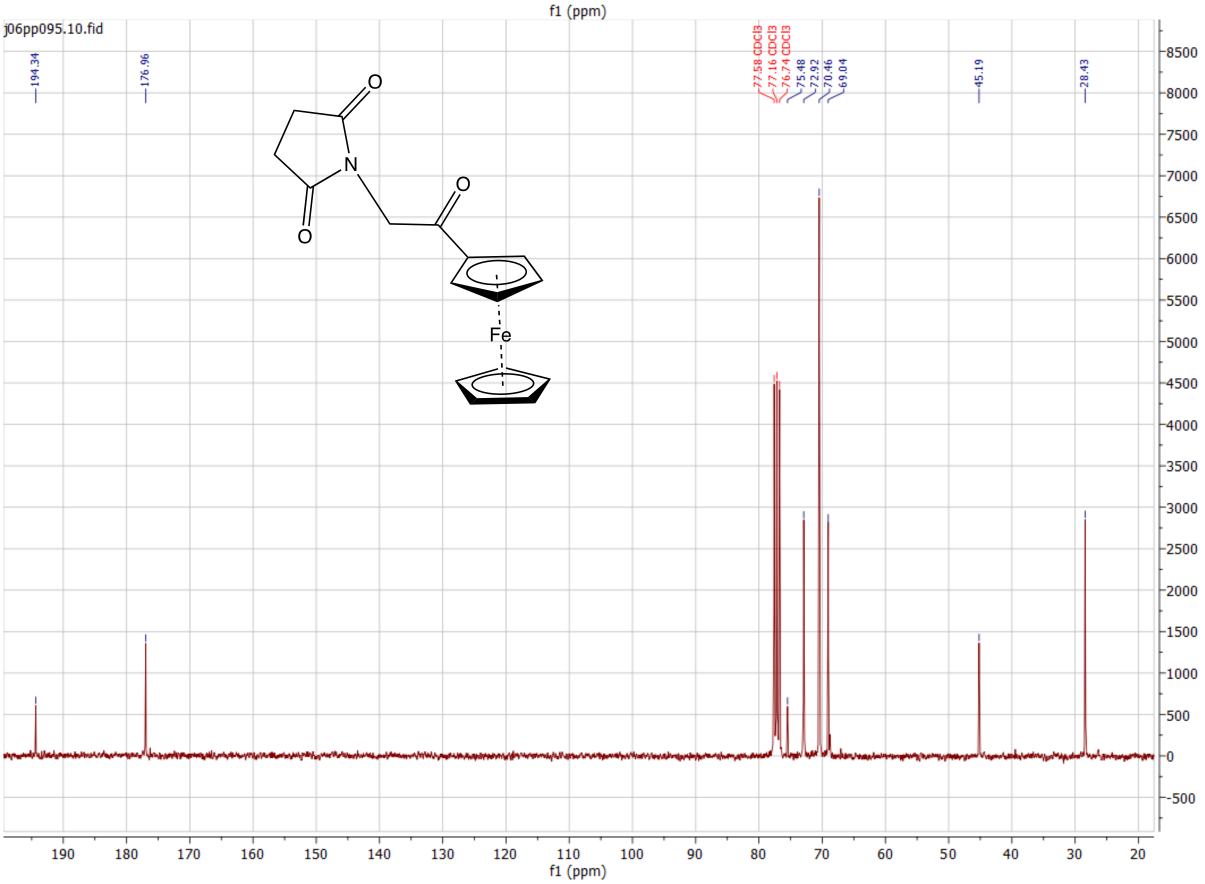

Single Mass Analysis
Tolerance = 5.0 PPM / DBE: min = -1.5, max = 100.0
Element prediction: Off
Number of isotope peaks used for i-FIT = 9

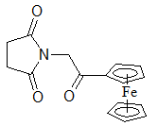

Monoisotopic Mass, Even Electron Ions
310 formula(e) evaluated with 2 results within limits (all results (up to 1000) for each mass)
Elements Used:
C: 0-100 H: 1-120 N: 0-10 O: 0-10 Fe: 1-1

06-Sep-2013 5::1::7
ENSCP_P737 26 (0.681) Cm (26:32)
MeOH
LCT Premier XE KE483
1: TOF MS ES+
1.44e+004

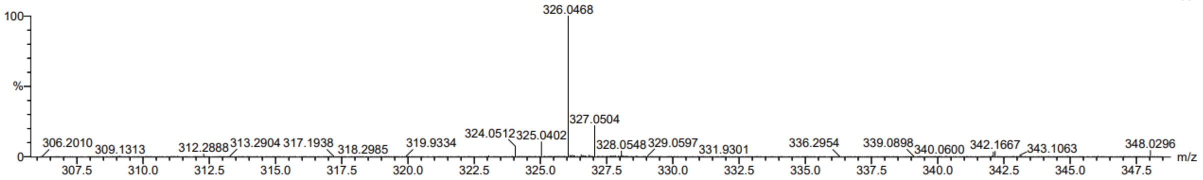

Minimum:				-1.5			
Maximum:		5.0	5.0	100.0			
Mass	Calc. Mass	mDa	PPM	DBE	i-FIT	i-FIT (Norm)	Formula
326.0468	326.0480	-1.2	-3.7	9.5	763.4	0.0	C16 H16 N O3 Fe
	326.0453	1.5	4.6	10.5	770.9	7.5	C12 H12 N7 O Fe

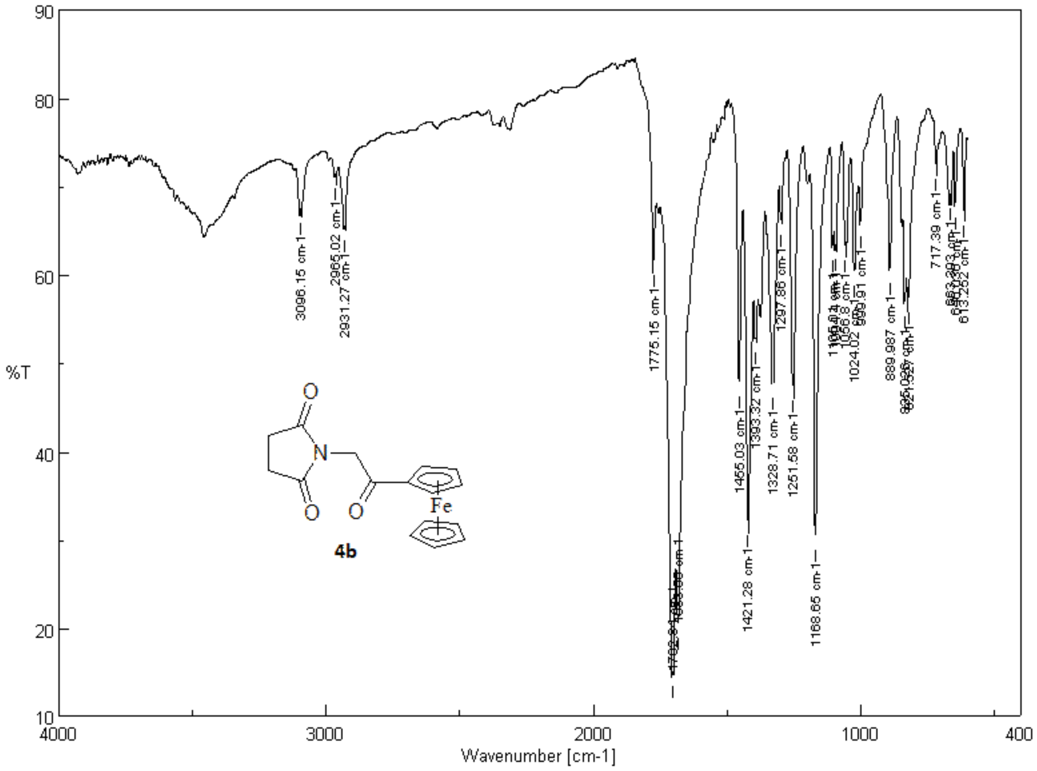

Figure S13: ^1H (in in $CDCl_3$), ^{13}C (in in $CDCl_3$) NMR, HR-MS and IR data for compound **4c**

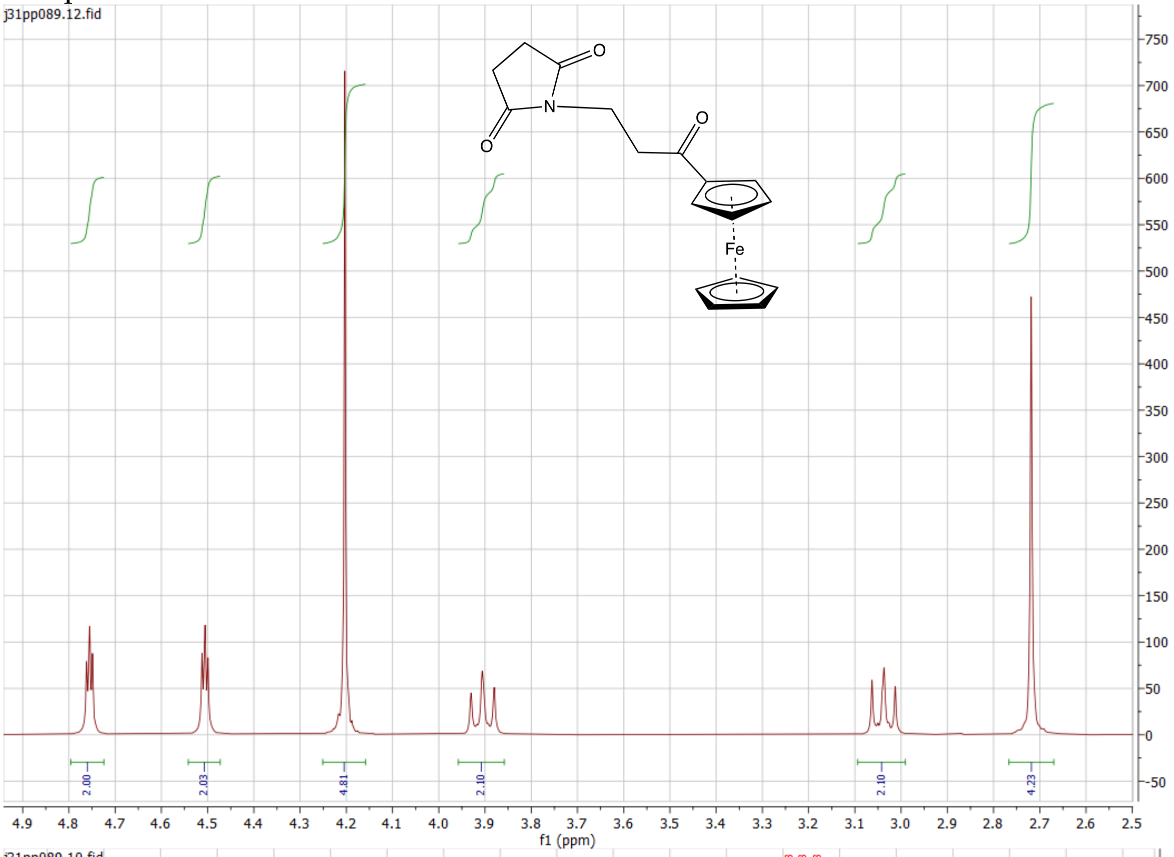

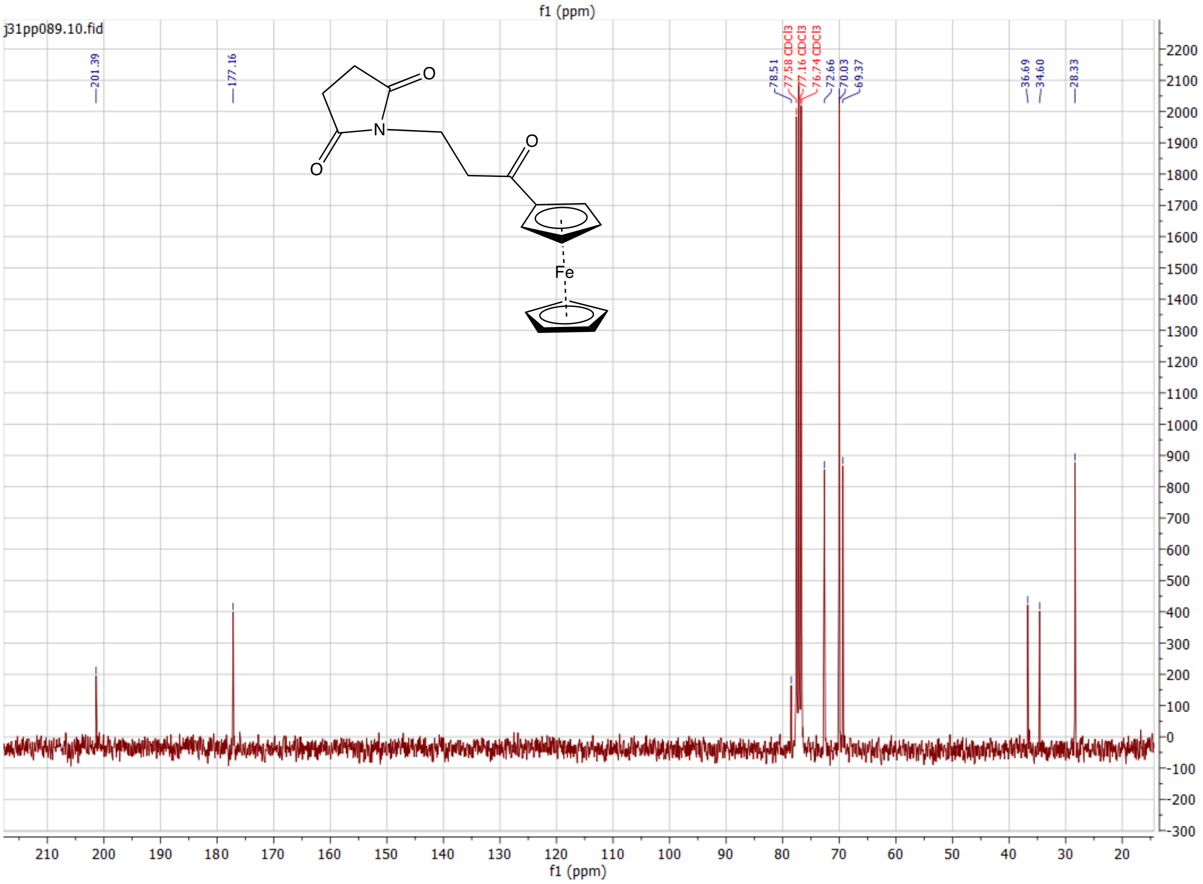

Single Mass Analysis
Tolerance = 5.0 PPM / DBE: min = -1.5, max = 100.0
Element prediction: Off
Number of isotope peaks used for i-FIT = 9

Monoisotopic Mass, Even Electron Ions
332 formula(e) evaluated with 2 results within limits (all results (up to 1000) for each mass)
Elements Used:
C: 0-100 H: 1-120 N: 0-10 O: 0-10 Fe: 1-1

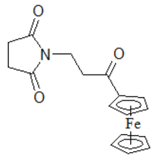

06-Sep-2013 5::8::9
ENSCP_P724 24 (0.625) Cm (24:31)
MeOH
LCT Premier XE KE483
1: TOF MS ES+
3.09e+004

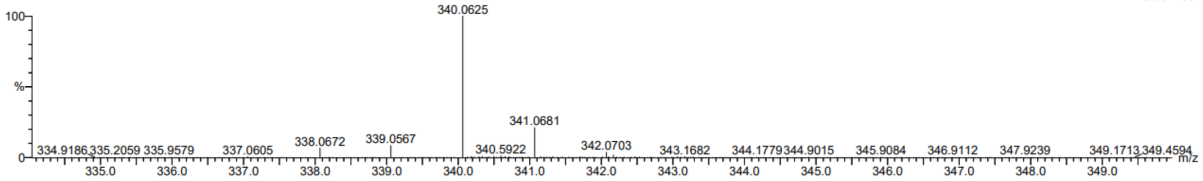

Minimum:				-1.5			
Maximum:		5.0	5.0	100.0			
Mass	Calc. Mass	mDa	PPM	DBE	i-FIT	i-FIT (Norm)	Formula
340.0625	340.0636	-1.1	-3.2	9.5	975.5	0.0	C17 H18 N O3 Fe
	340.0609	1.6	4.7	10.5	982.2	6.7	C13 H14 N7 O Fe

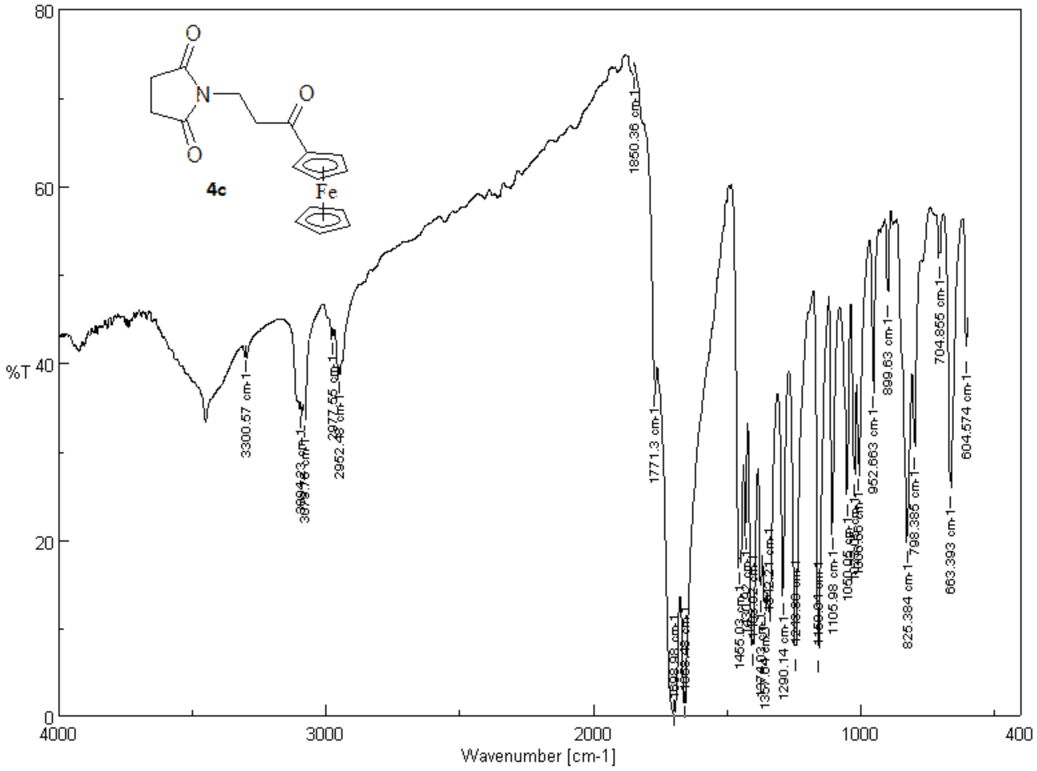

Figure S14: ^{1}H (in $CDCl_3$), ^{13}C (in $CDCl_3$) NMR, HR-MS and IR data for compound **4d**

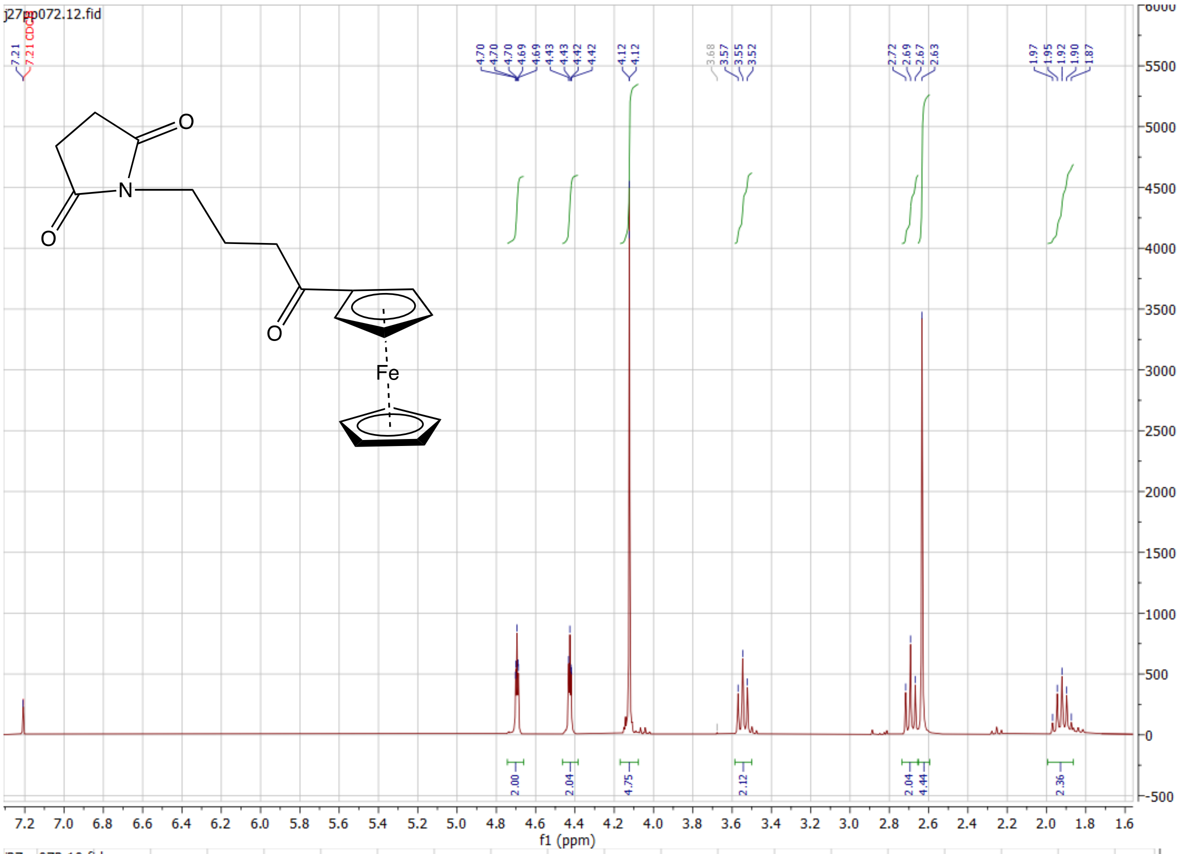

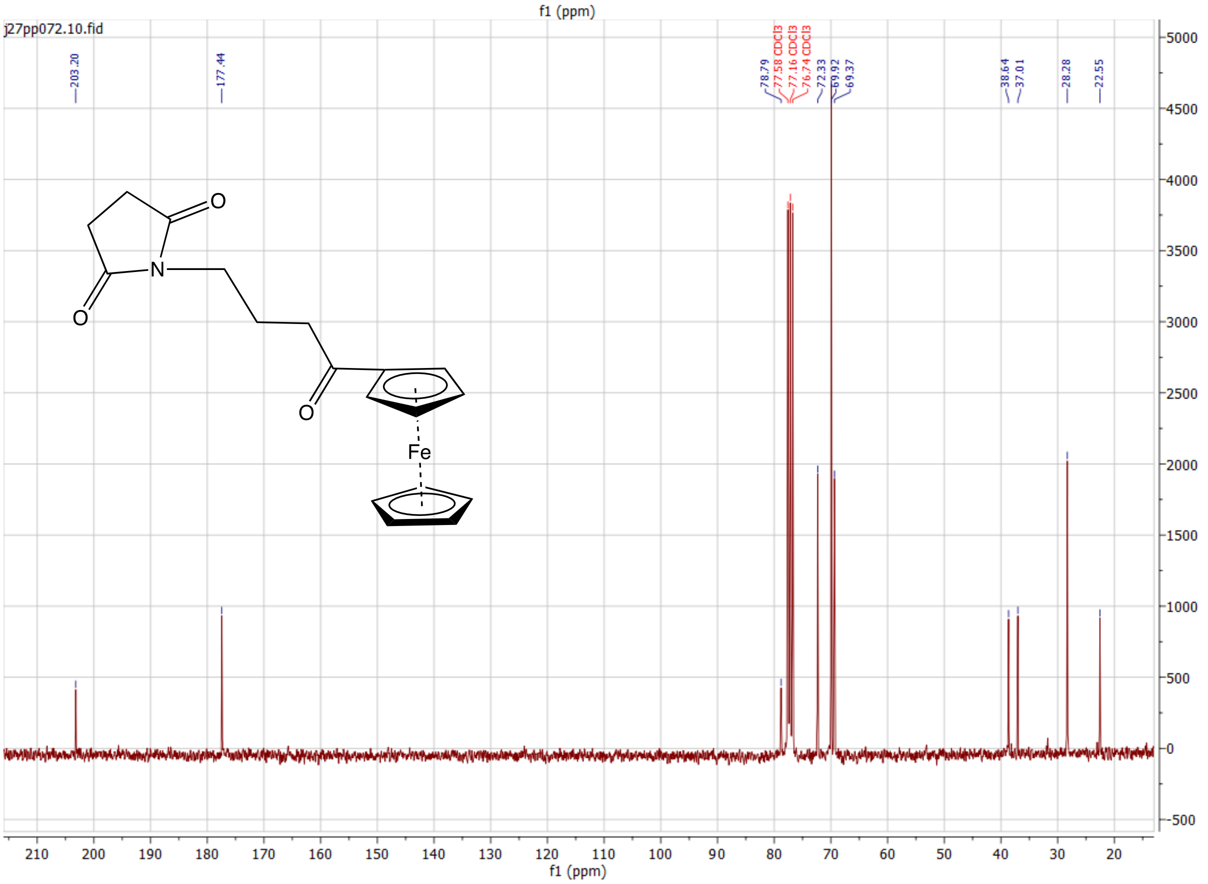

Single Mass Analysis
Tolerance = 5.0 PPM / DBE: min = -1.5, max = 100.0
Element prediction: Off
Number of isotope peaks used for i-FIT = 9

Monoisotopic Mass, Even Electron Ions
356 formula(e) evaluated with 1 results within limits (all results (up to 1000) for each mass)
Elements Used:
C: 0-100 H: 1-120 N: 0-10 O: 0-10 Fe: 1-1

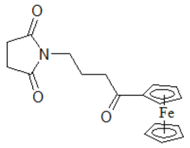

06-Sep-2013 5::3::2
ENSCP_P726 21 (0.573) Cm (20:28)

MeOH

LCT Premier XE KE483
1: TOF MS ES+
4.84e+004

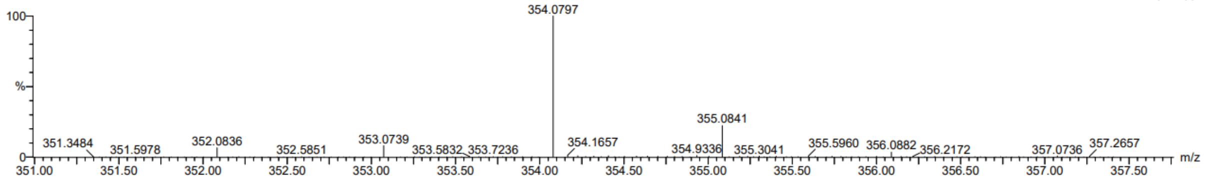

	Mass	Calc. Mass	mDa	PPM	DBE	i-FIT	i-FIT (Norm)	Formula
Minimum:					-1.5			
Maximum:			5.0	5.0	100.0			
	354.0797	354.0793	0.4	1.1	9.5	604.1	0.0	C18 H20 N O3 Fe

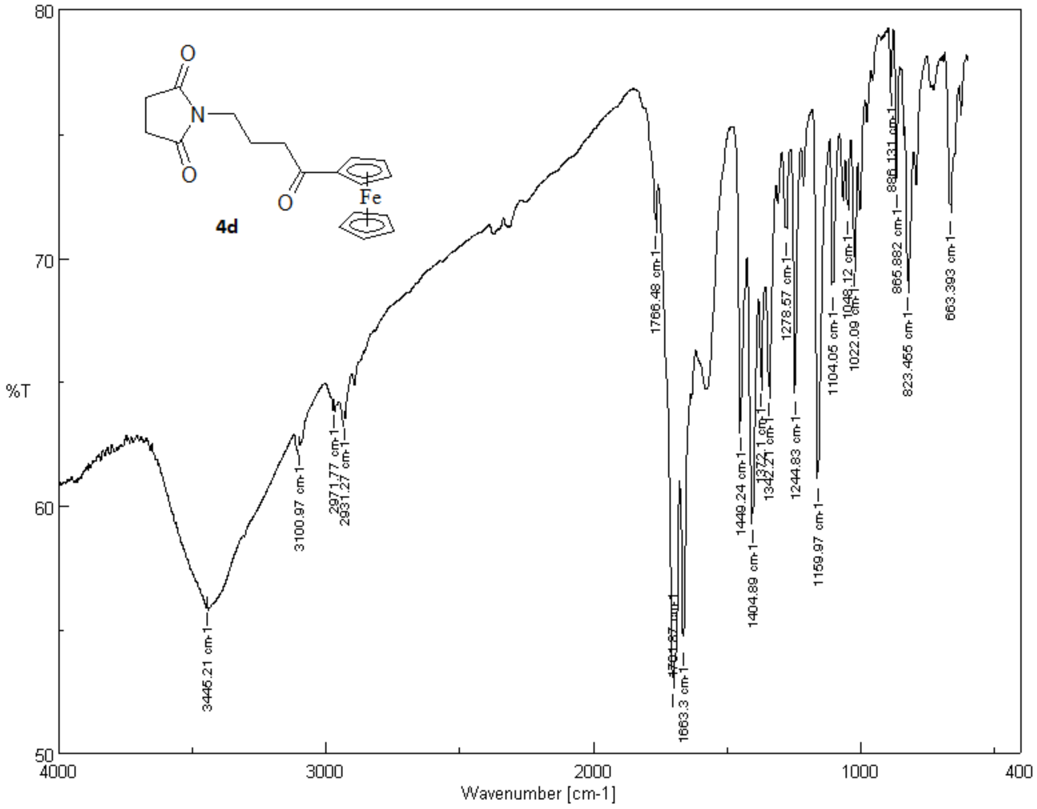

Figure S15: ^{1}H (in $CDCl_3$),^{13}C (in $CDCl_3$) NMR, HR-MS and IR data for compound **4f**

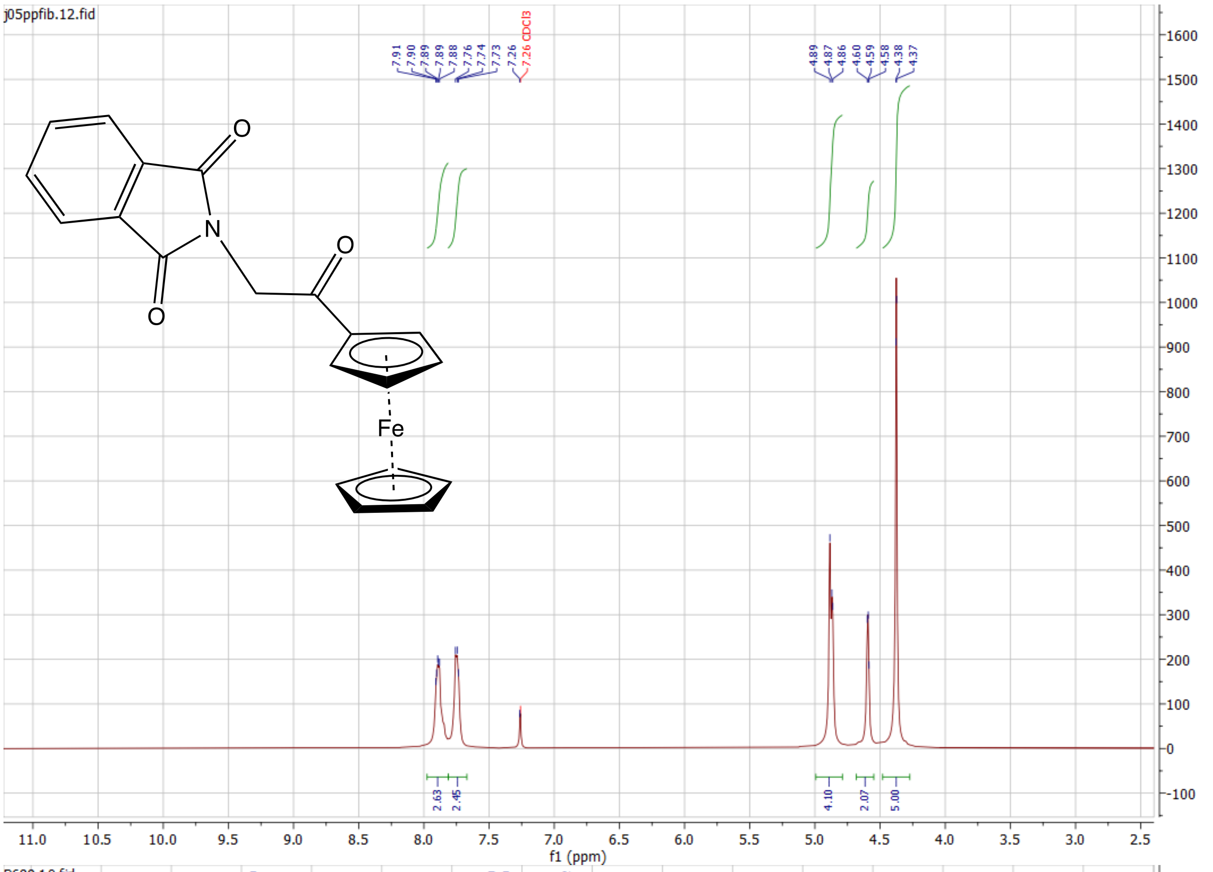

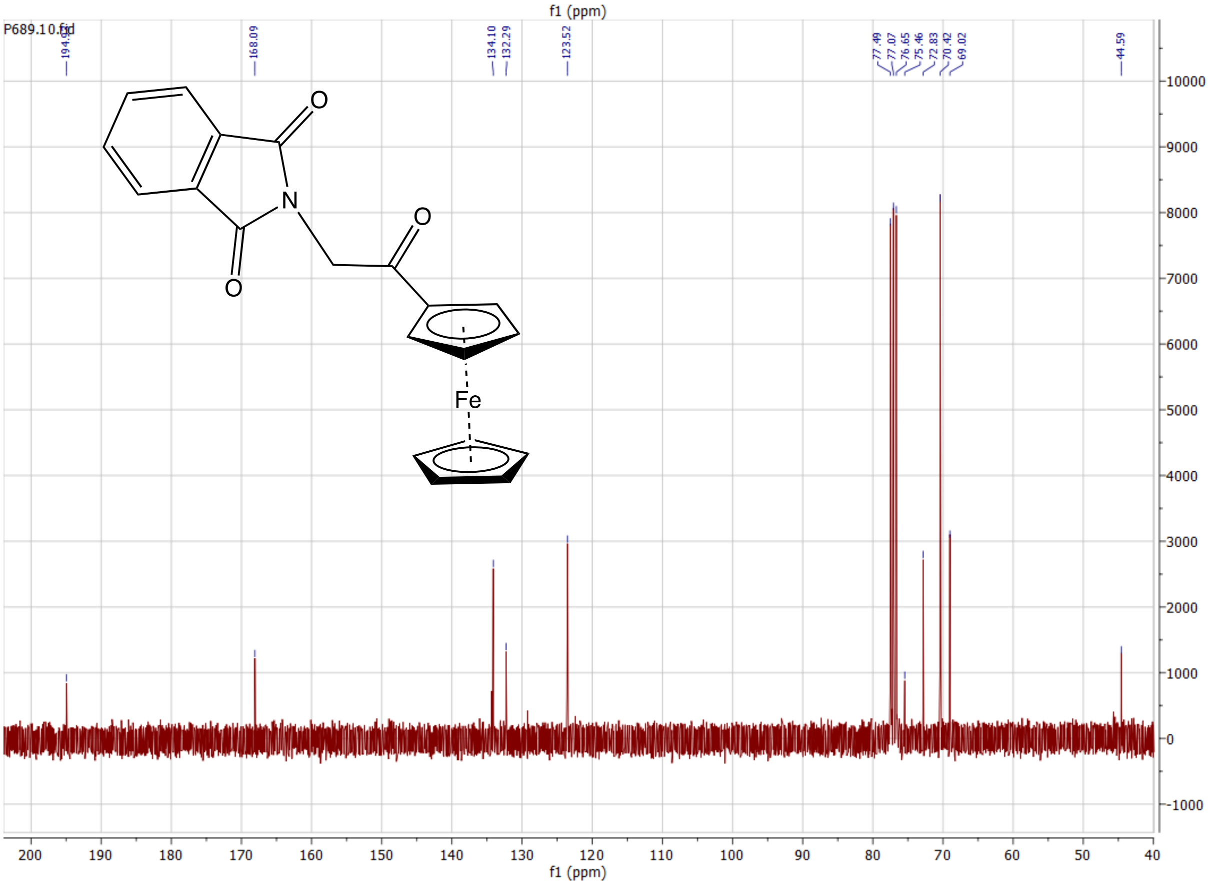

Single Mass Analysis
Tolerance = 5.0 PPM / DBE: min = -1.5, max = 100.0
Element prediction: Off
Number of isotope peaks used for i-FIT = 9

Monoisotopic Mass, Even Electron Ions
382 formula(e) evaluated with 2 results within limits (all results (up to 1000) for each mass)
Elements Used:
C: 0-100 H: 1-120 N: 0-10 O: 0-10 Fe: 1-1

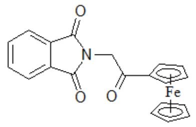

06-Sep-2013 5::8::1
ENSCP_P689 25 (0.661) Cm (25:32)
MeOH
LCT Premier XE KE483
1: TOF MS ES+
2.57e+004

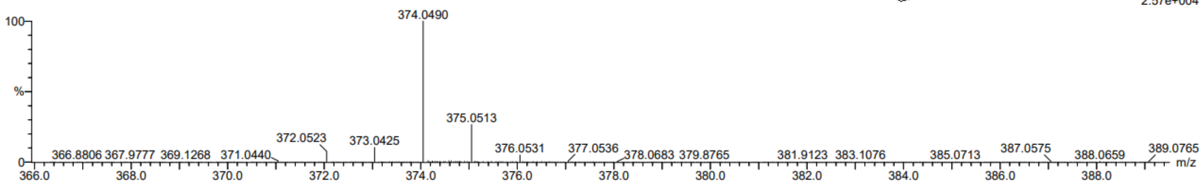

Minimum:				-1.5			
Maximum:		5.0	5.0	100.0			
Mass	Calc. Mass	mDa	PPM	DBE	i-FIT	i-FIT (Norm)	Formula
374.0490	374.0480	1.0	2.7	13.5	836.7	0.0	C20 H16 N O3 Fe
	374.0498	-0.8	-2.1	0.5	849.4	12.7	C8 H20 N3 O10 Fe

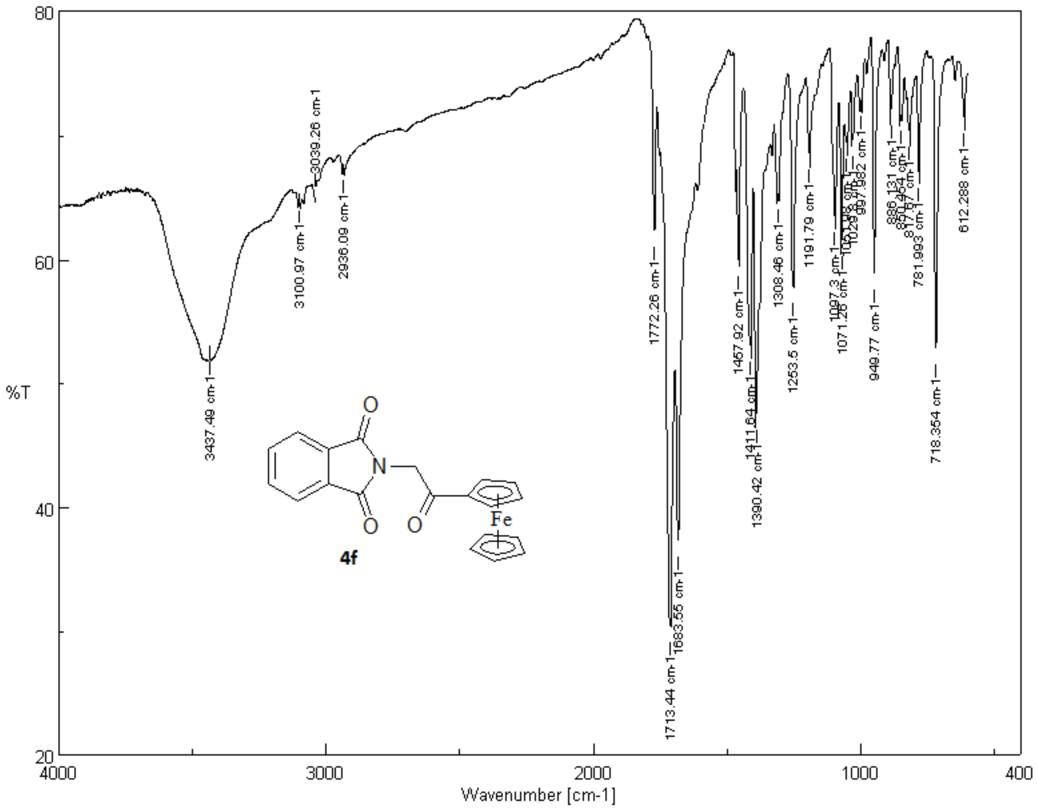

Figure S16: ^{1}H (in $CDCl_3$), ^{13}C (in $CDCl_3$) NMR, HR-MS and IR data for compound **4g**

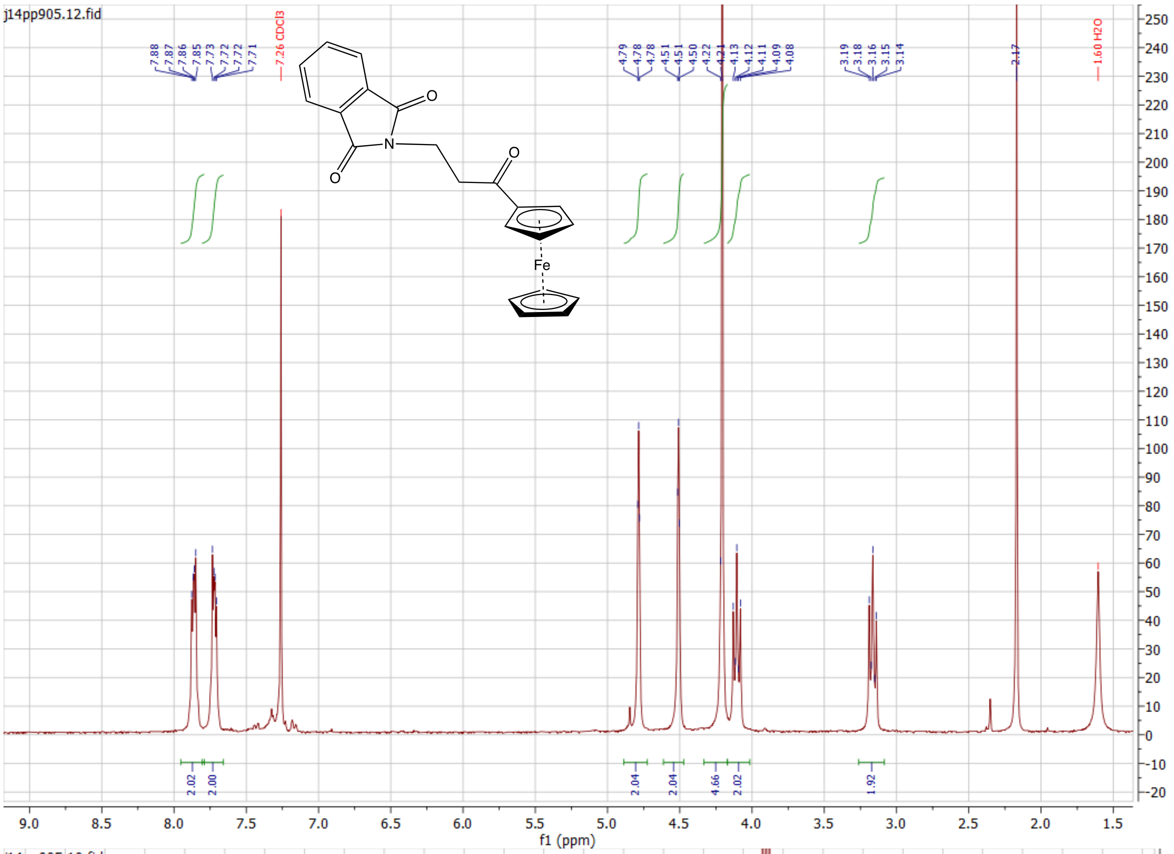

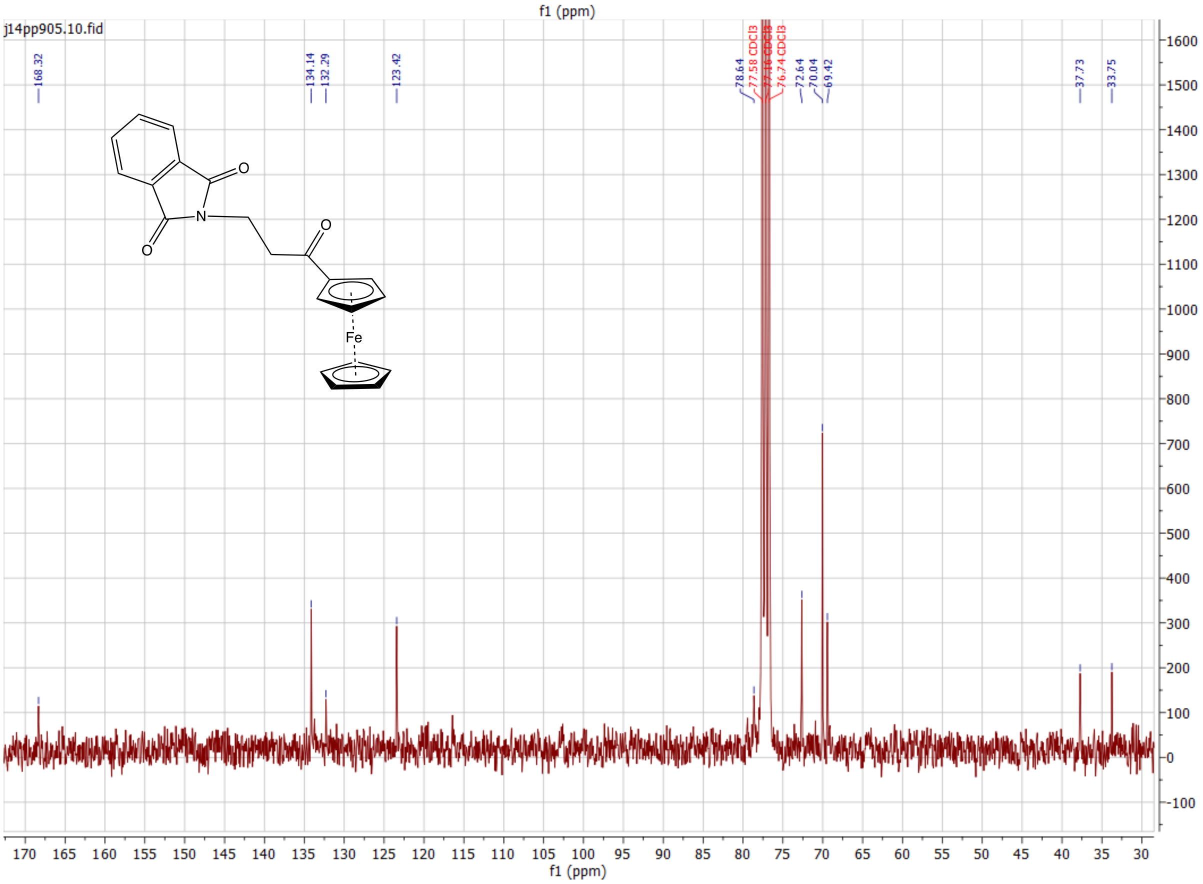

Single Mass Analysis
Tolerance = 5.0 PPM / DBE: min = -1.5, max = 100.0
Element prediction: Off
Number of isotope peaks used for i-FIT = 9

Monoisotopic Mass, Even Electron Ions
401 formula(e) evaluated with 3 results within limits (all results (up to 1000) for each mass)
Elements Used:
C: 0-100 H: 1-120 N: 0-10 O: 0-10 Fe: 1-1

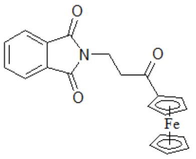

06-Sep-2013 5::2::1
ENSCP_P662 26 (0.680) Cm (26:32)
MeOH
LCT Premier XE KE483
1: TOF MS ES+
2.21e+004

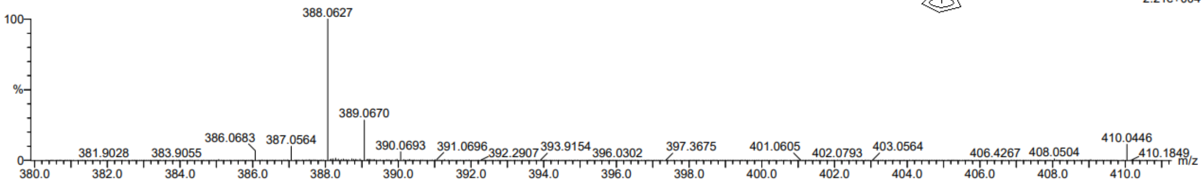

Minimum:				-1.5			
Maximum:		5.0	5.0	100.0			
Mass	Calc. Mass	mDa	PPM	DBE	i-FIT	i-FIT (Norm)	Formula
388.0627	388.0636	-0.9	-2.3	13.5	795.1	0.0	C21 H18 N O3 Fe
	388.0609	1.8	4.6	14.5	803.3	8.2	C17 H14 N7 O Fe
	388.0628	-0.1	-0.3	1.5	811.9	16.8	C5 H18 N9 O8 Fe

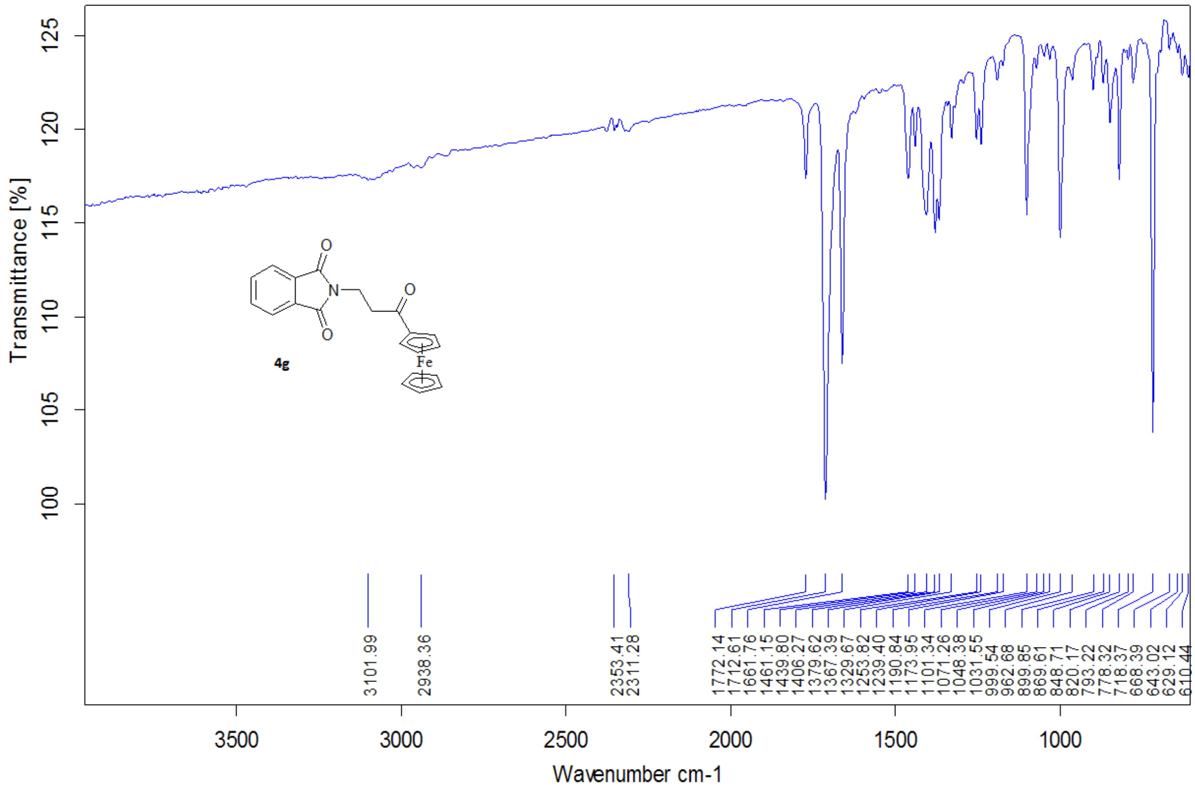

Figure S17: ^{1}H (in $CDCl_3$), ^{13}C (in $CDCl_3$) NMR, HR-MS and IR data for compound **4h**

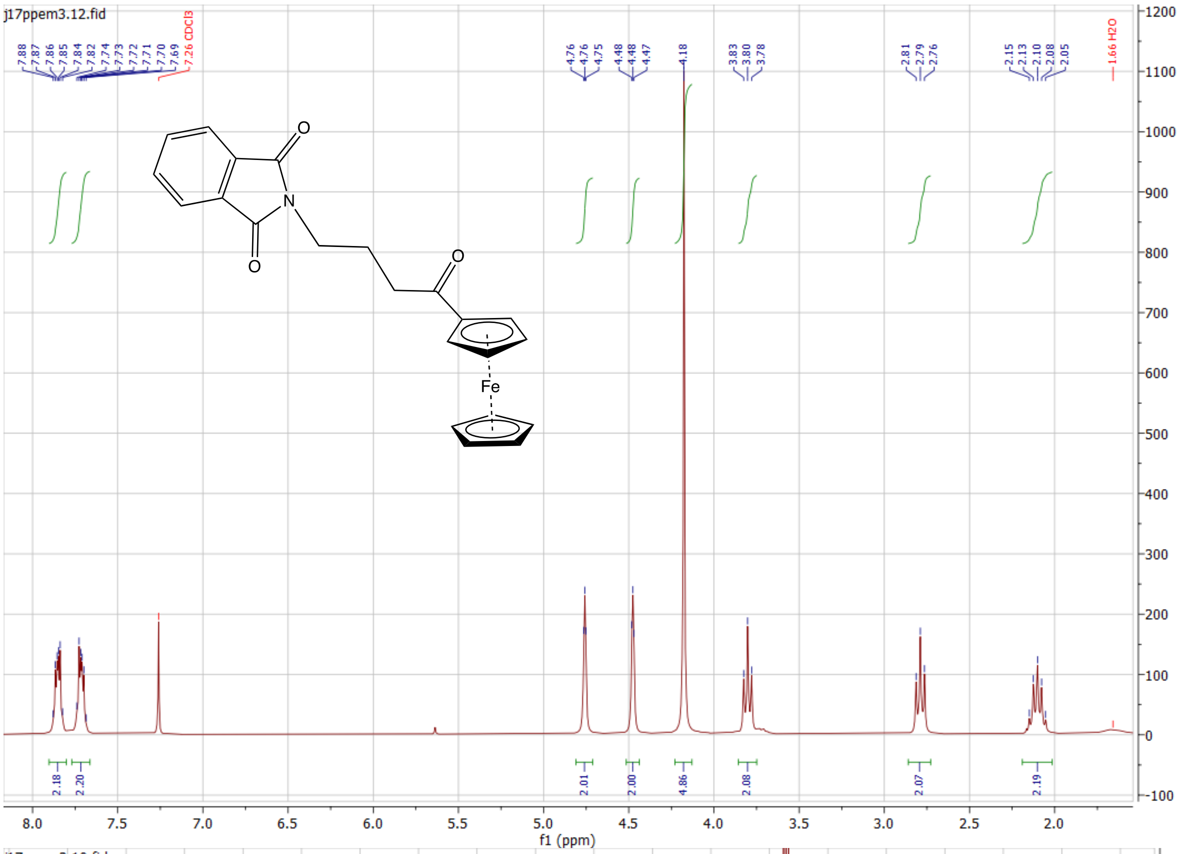

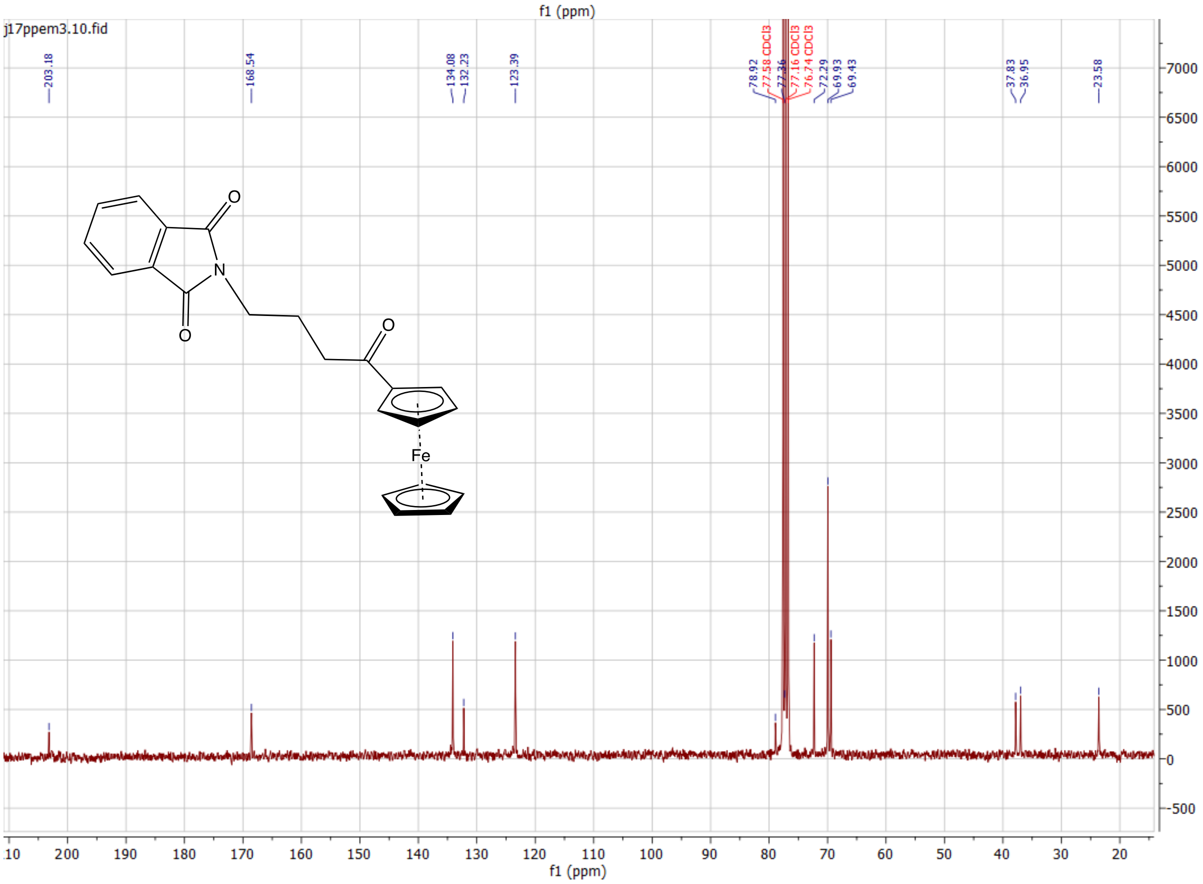

Single Mass Analysis
Tolerance = 5.0 PPM / DBE: min = -1.5, max = 100.0
Element prediction: Off
Number of isotope peaks used for i-FIT = 9

Monoisotopic Mass, Even Electron Ions
423 formula(e) evaluated with 2 results within limits (all results (up to 1000) for each mass)
Elements Used:
C: 0-100 H: 1-120 N: 0-10 O: 0-10 Fe: 1-1

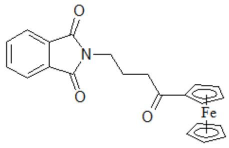

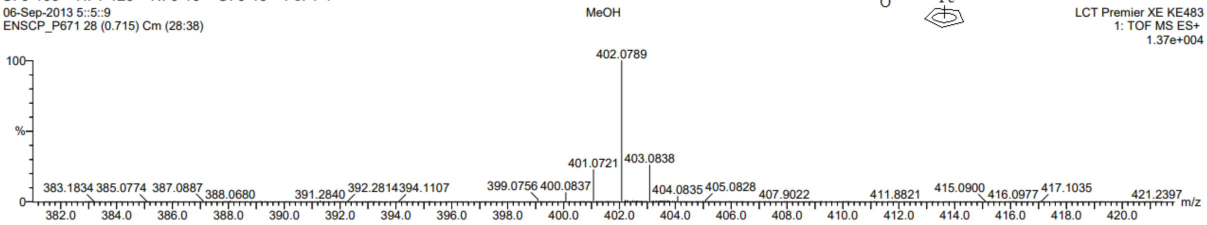

Minimum:				-1.5			
Maximum:		5.0	5.0	100.0			
Mass	Calc. Mass	mDa	PPM	DBE	i-FIT	i-FIT (Norm)	Formula
402.0789	402.0793	-0.4	-1.0	13.5	810.4	0.0	C22 H20 N O3 Fe
	402.0784	0.5	1.2	1.5	824.7	14.3	C6 H20 N9 O8 Fe

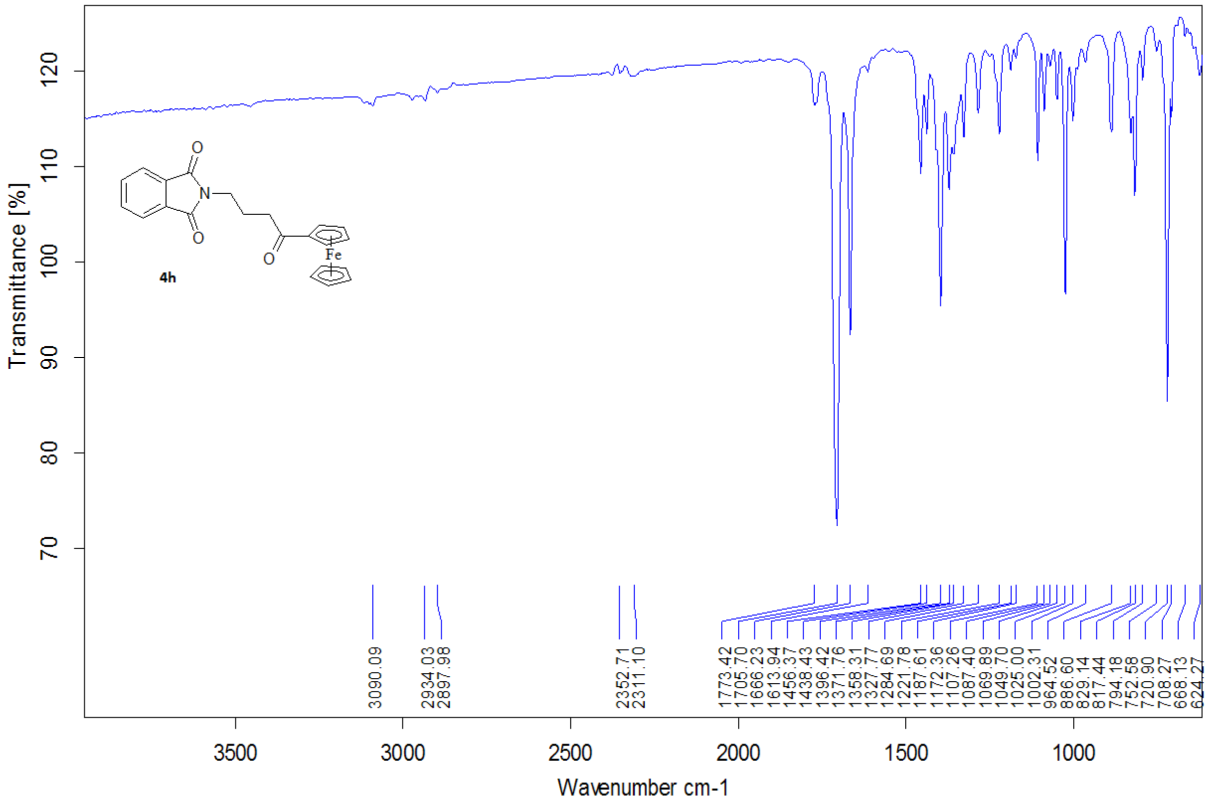

Figure S18: ^{1}H (in DMSO-d$_6$), ^{13}C (in DMSO-d$_6$), COSY (in DMSO-d$_6$) NMR, NMR, HR-MS and IR data for compound **6a**

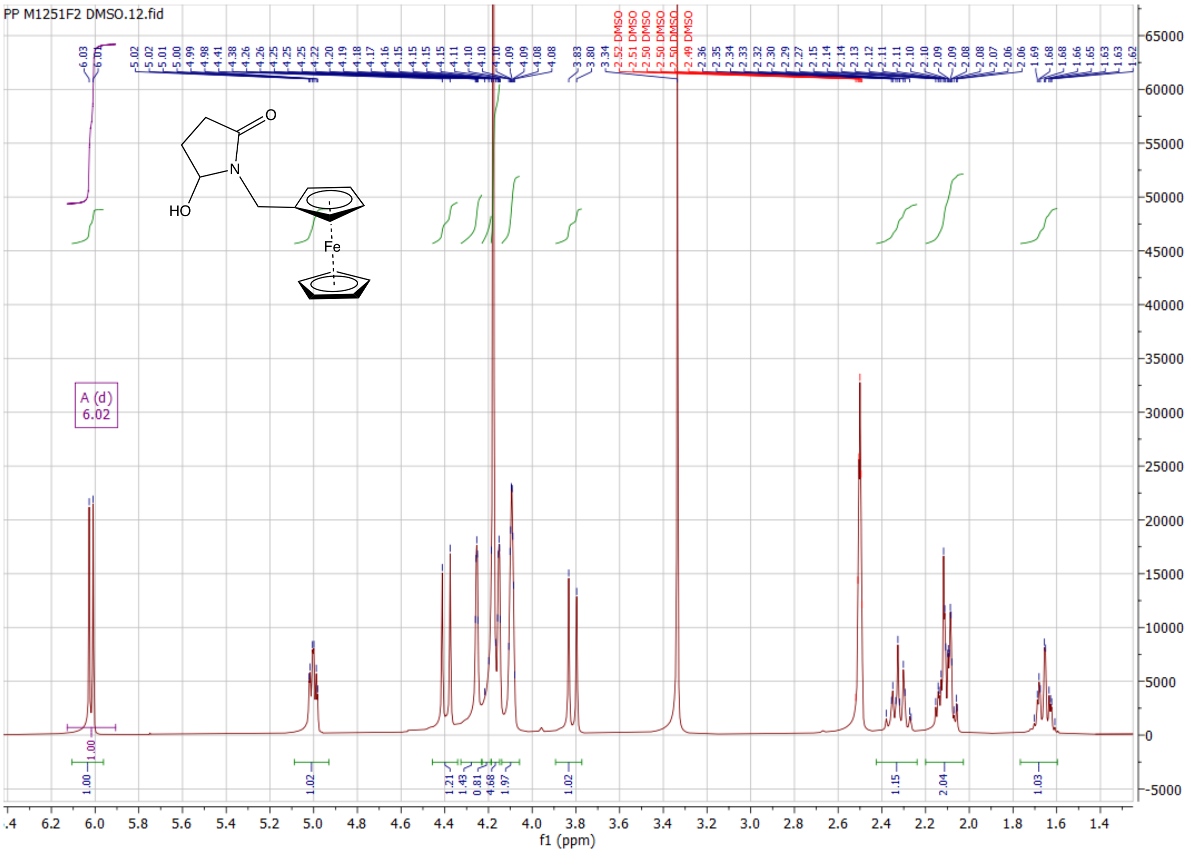

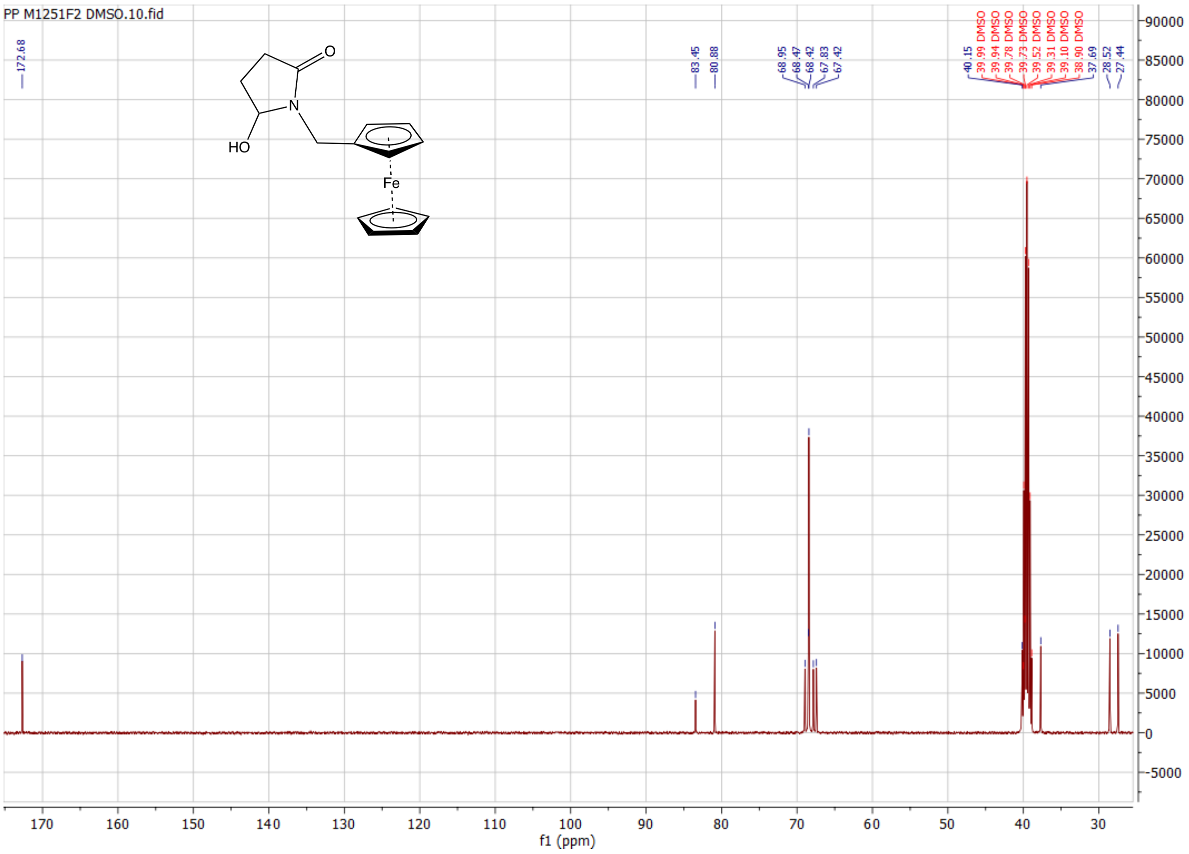

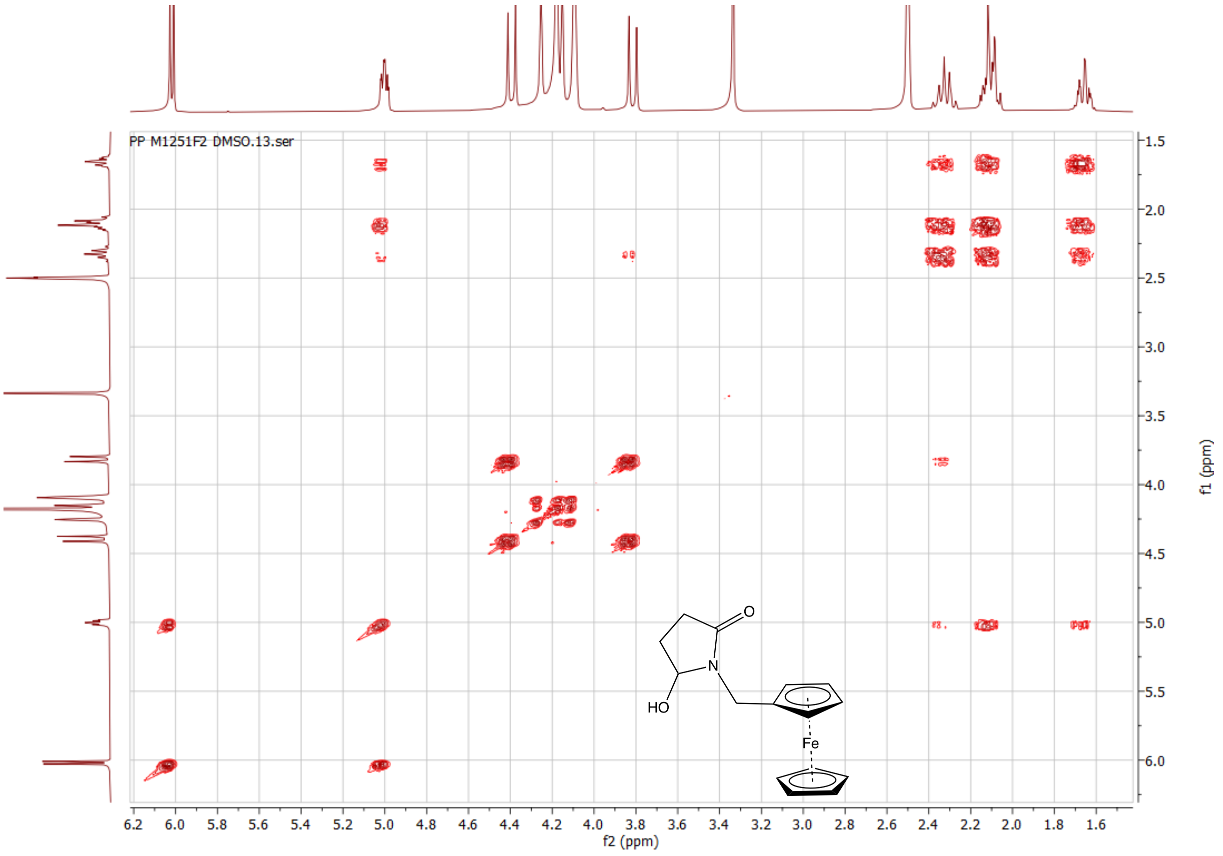

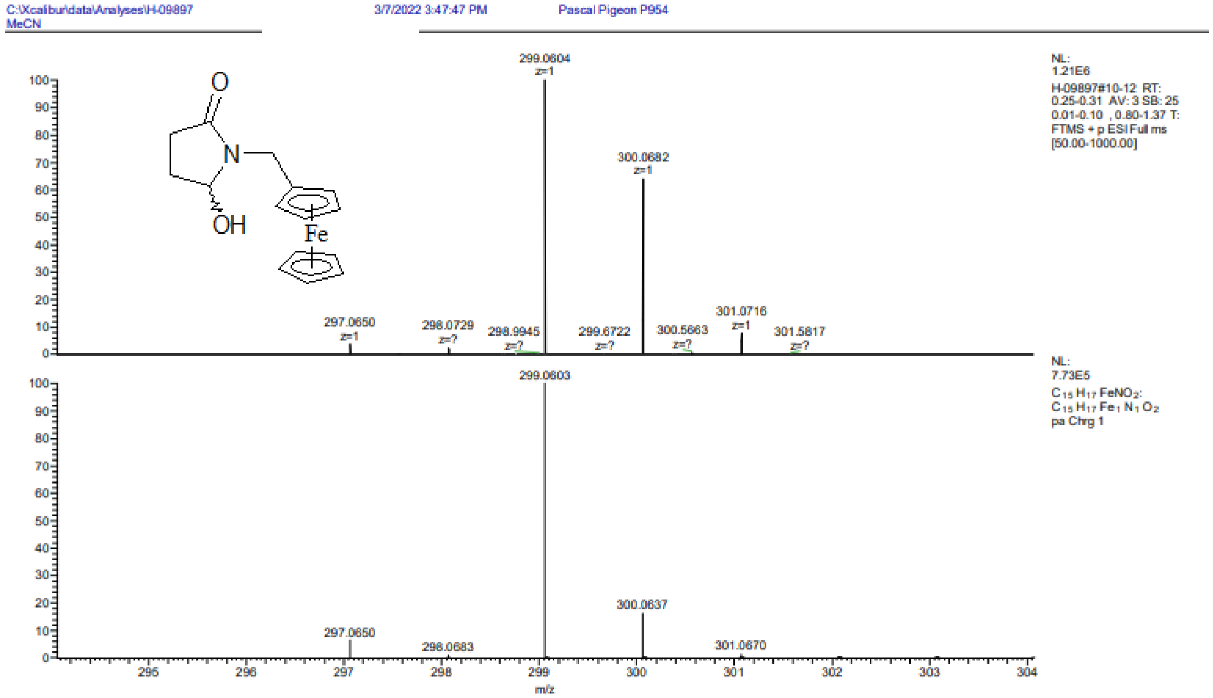

Experimental/theoretical isotopic pattern MS spectrum

Error = 0.3 ppm; Relative Intensity (%) 100

HRMS (ESI) m/z: [M]+ Calcd for C15H17FeNO2 299.0603 . Found 299.0604; (Error: 0.3 ppm).

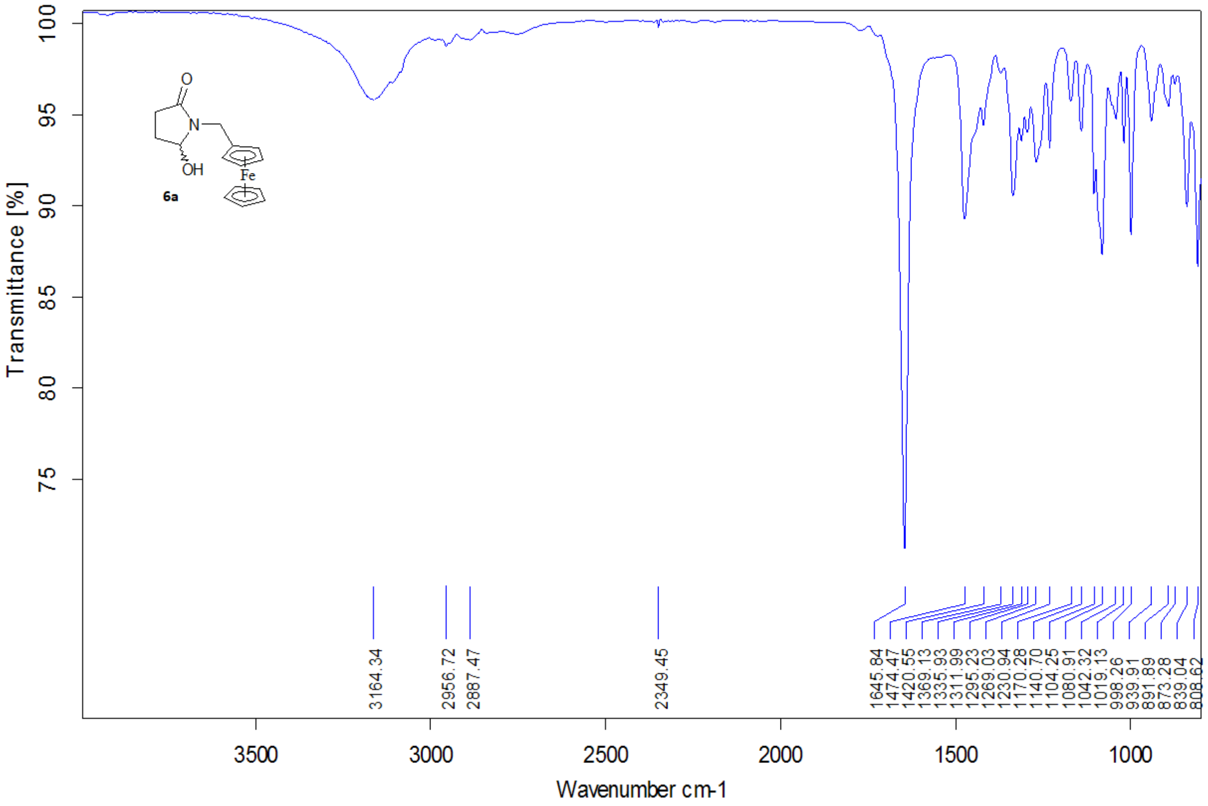

Transmittance [%]
100
95
90
85
80
75
O
N
OH
Fe
6a
3164.34
2956.72
2887.47
2349.45
1645.84
1474.47
1420.55
1369.13
1335.93
1311.99
1295.23
1269.03
1230.94
1170.28
1140.70
1104.25
1080.91
1042.32
1019.13
998.26
939.91
891.89
873.28
839.04
808.62
3500
3000
2500
2000
1500
1000
Wavenumber cm-1

Figure S19: ^{1}H (in DMSO-d6), ^{13}C (in DMSO-d6), COSY (in DMSO-d6), HMQC (in DMSO-d6) NMR and IR data for compound **7a**

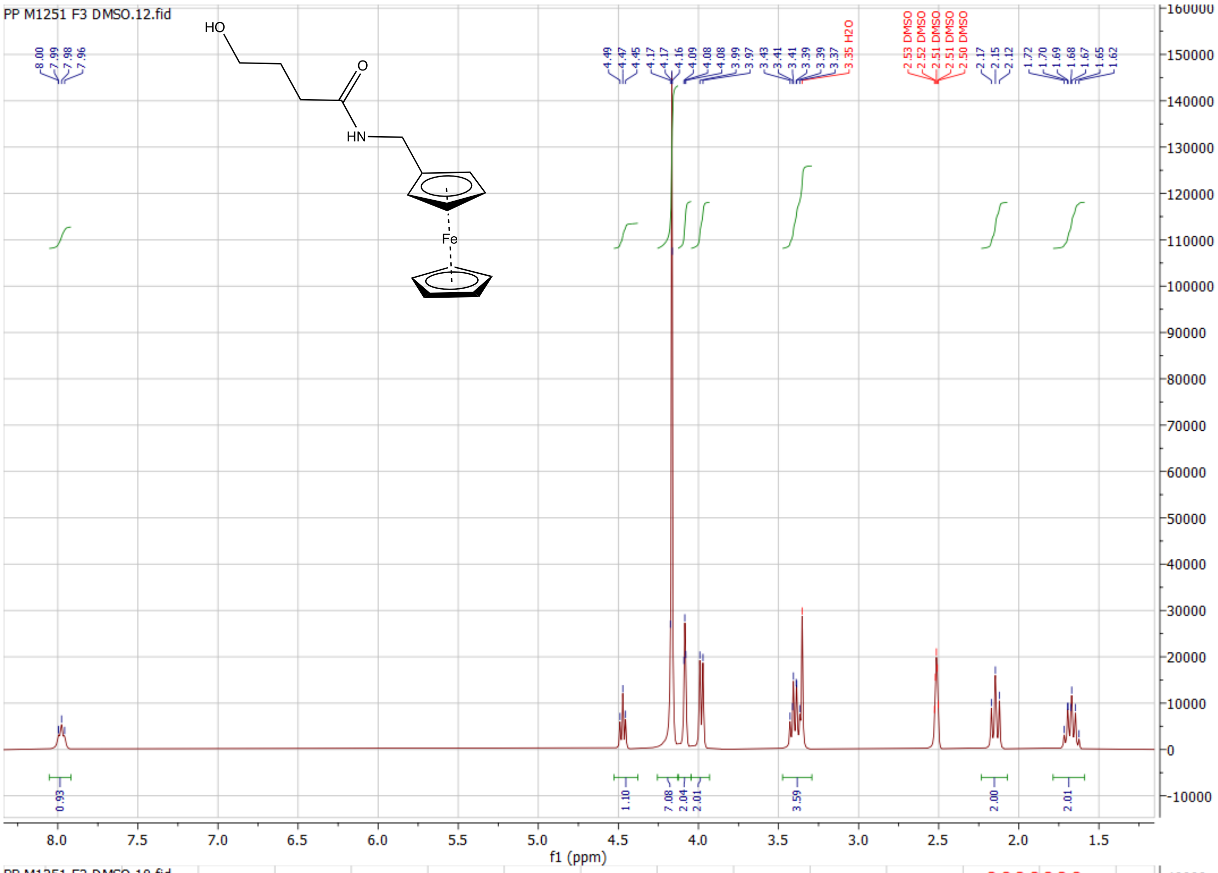

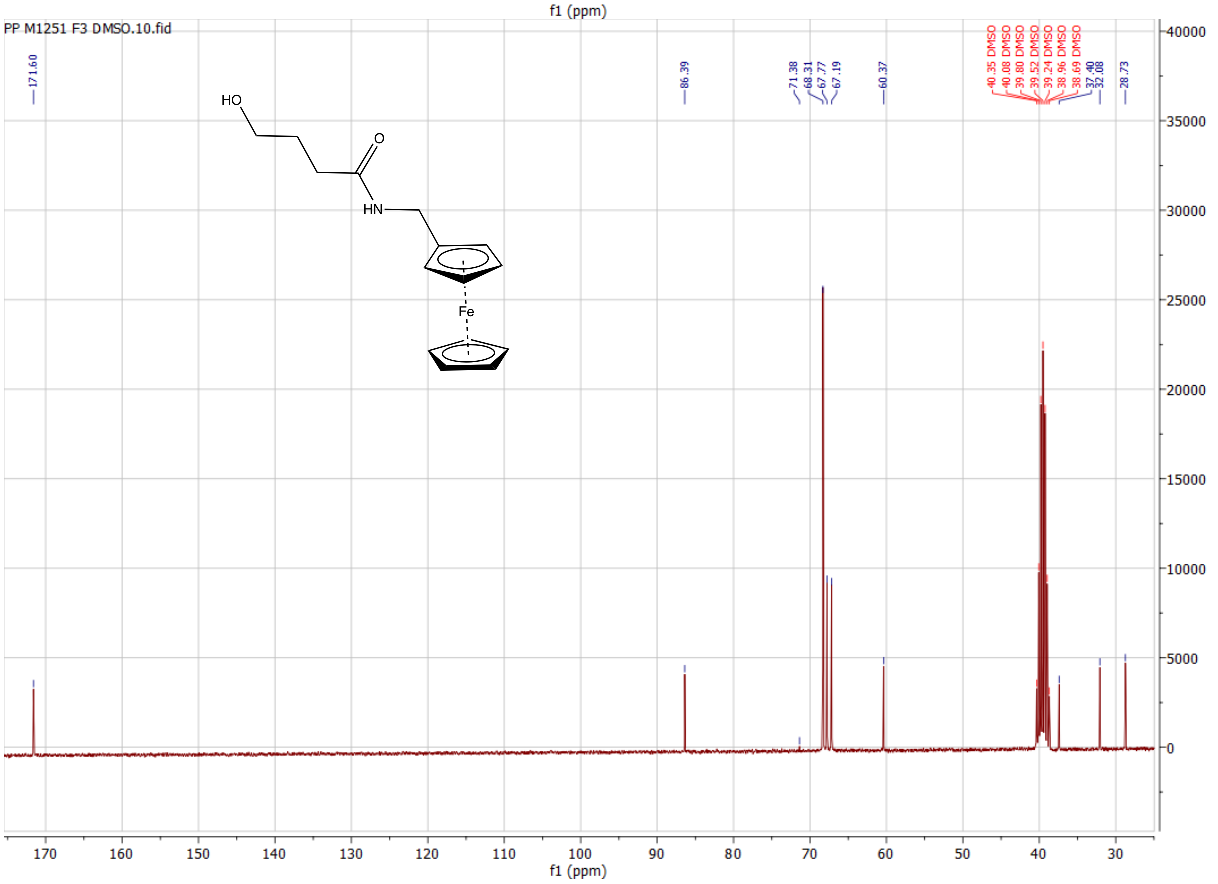

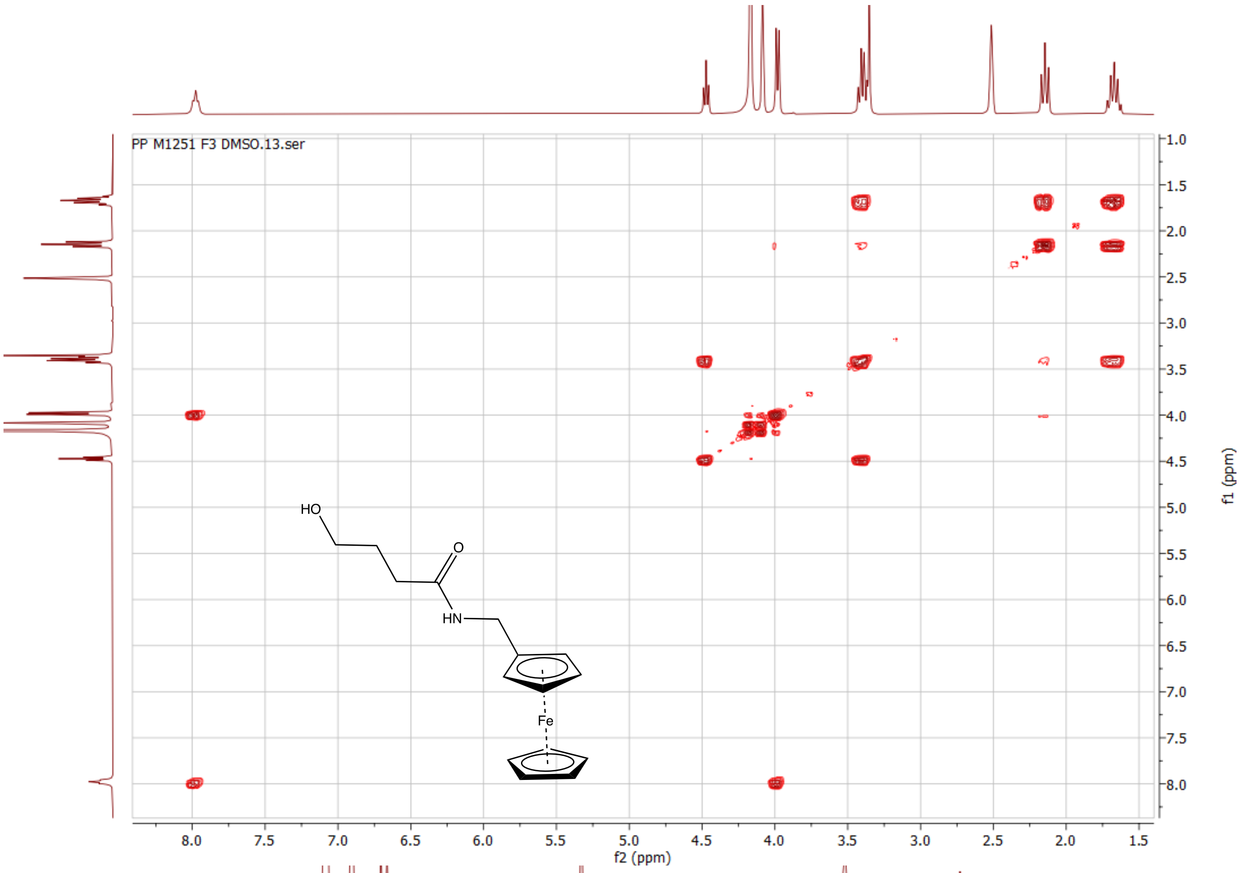
PP M1251 F3 DMSO.13.ser
HO
O
HN
Fe
f2 (ppm)
f1 (ppm)

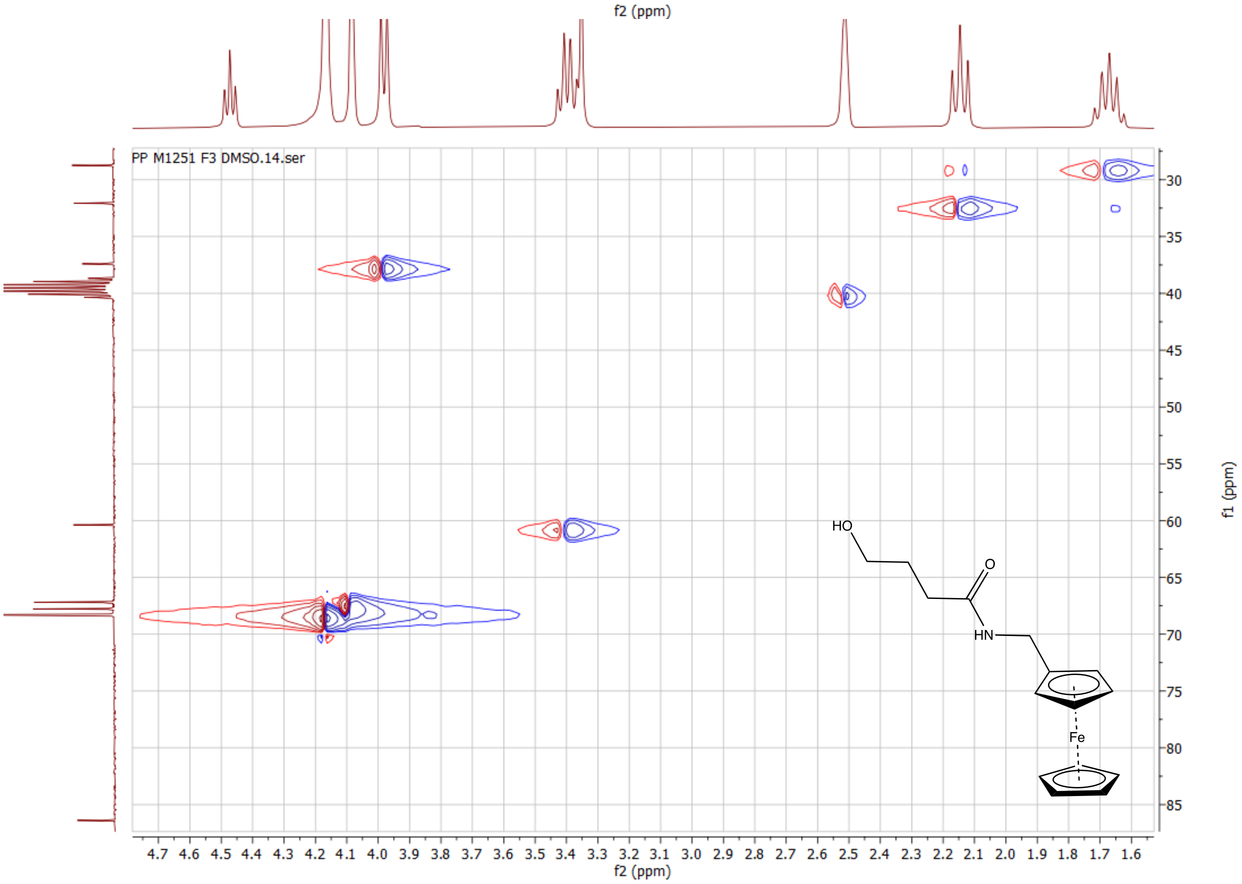
PP M1251 F3 DMSO.14.ser
HO
O
HN
Fe
f2 (ppm)
f1 (ppm)

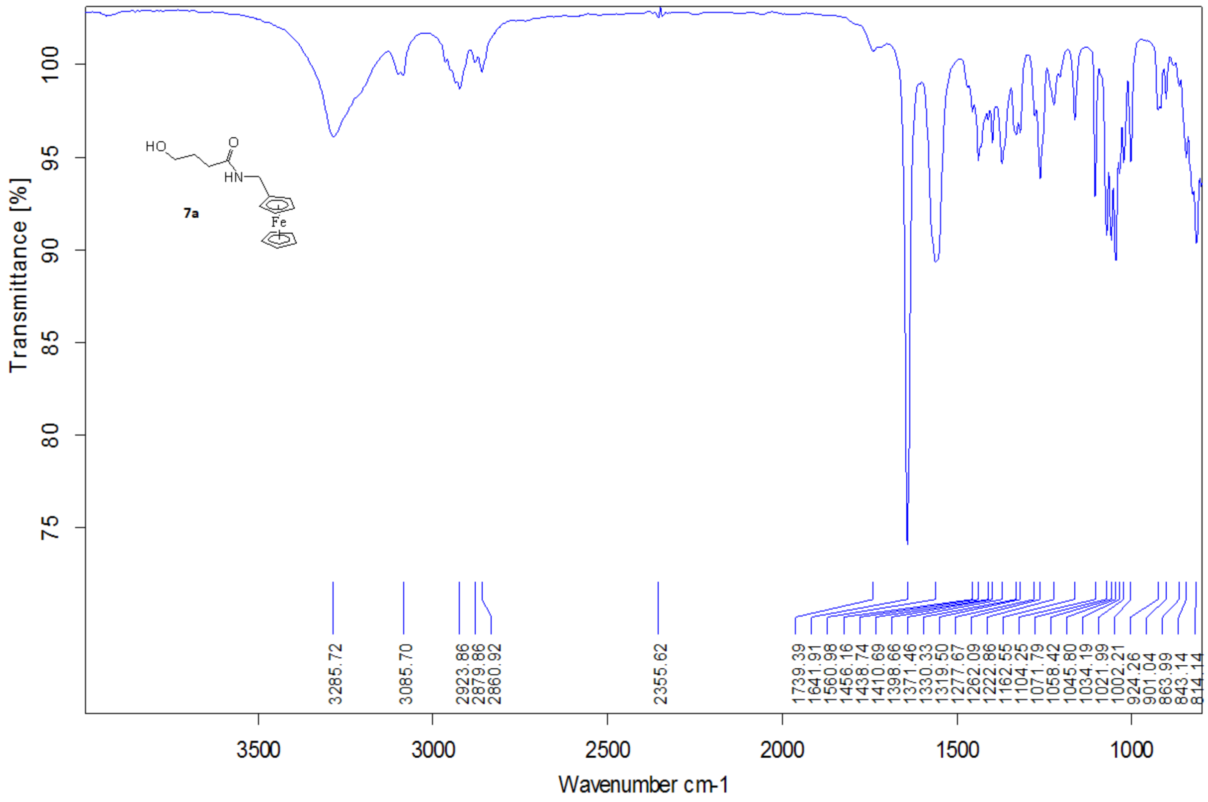

HO
O
HN
Fe
7a
Transmittance [%]
Wavenumber cm-1
3285.72
3085.70
2923.86
2879.86
2860.92
2355.62
1739.39
1641.91
1560.98
1456.16
1438.74
1410.69
1398.66
1371.46
1330.33
1319.50
1277.67
1262.09
1222.86
1162.55
1104.25
1071.79
1058.42
1045.80
1034.19
1021.99
1002.21
924.26
901.04
863.99
843.14
814.14

Figure S20: ^{1}H (in DMSO-d6) NMR for compound **6b**

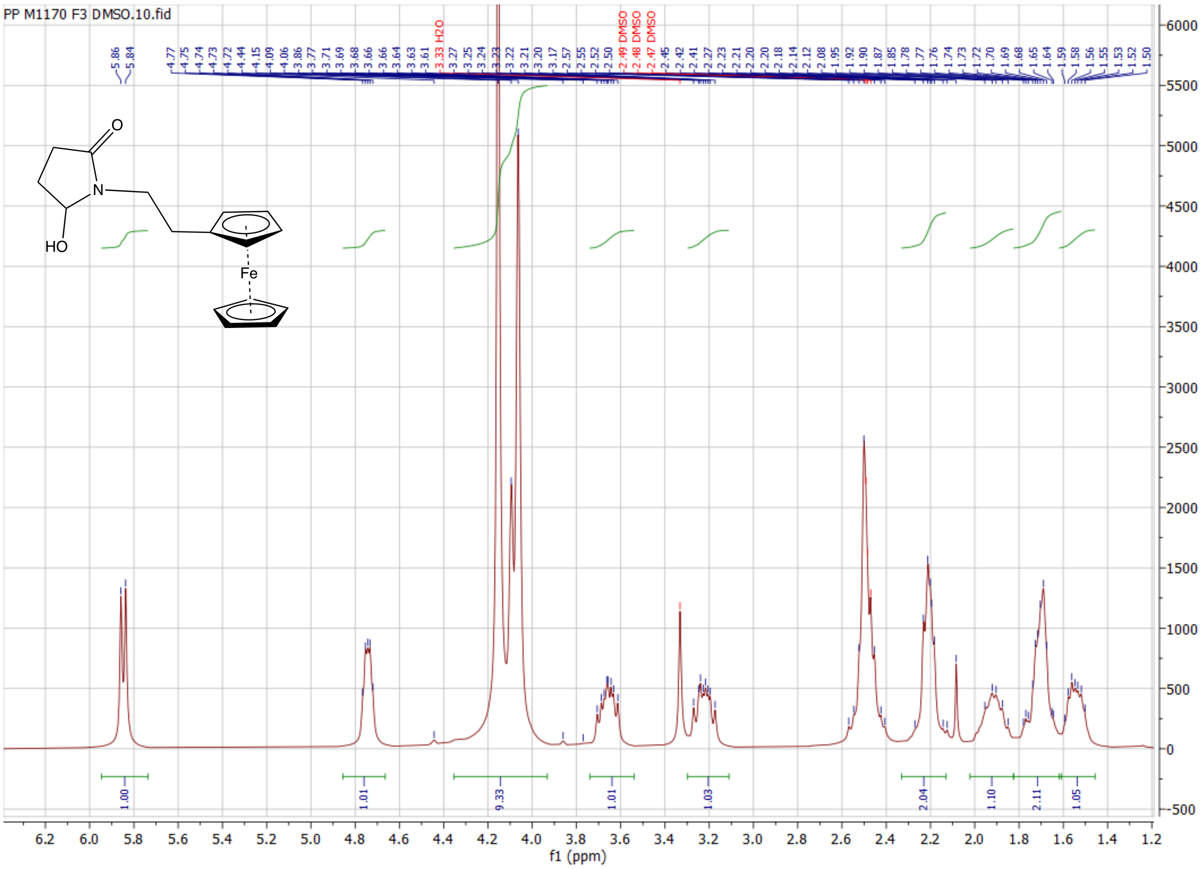

Figure S21: ^{1}H (in CDCl3), ^{13}C (in CDCl3), COSY (in CDCl3) NMR, HR-MS and IR data for compound **6c**

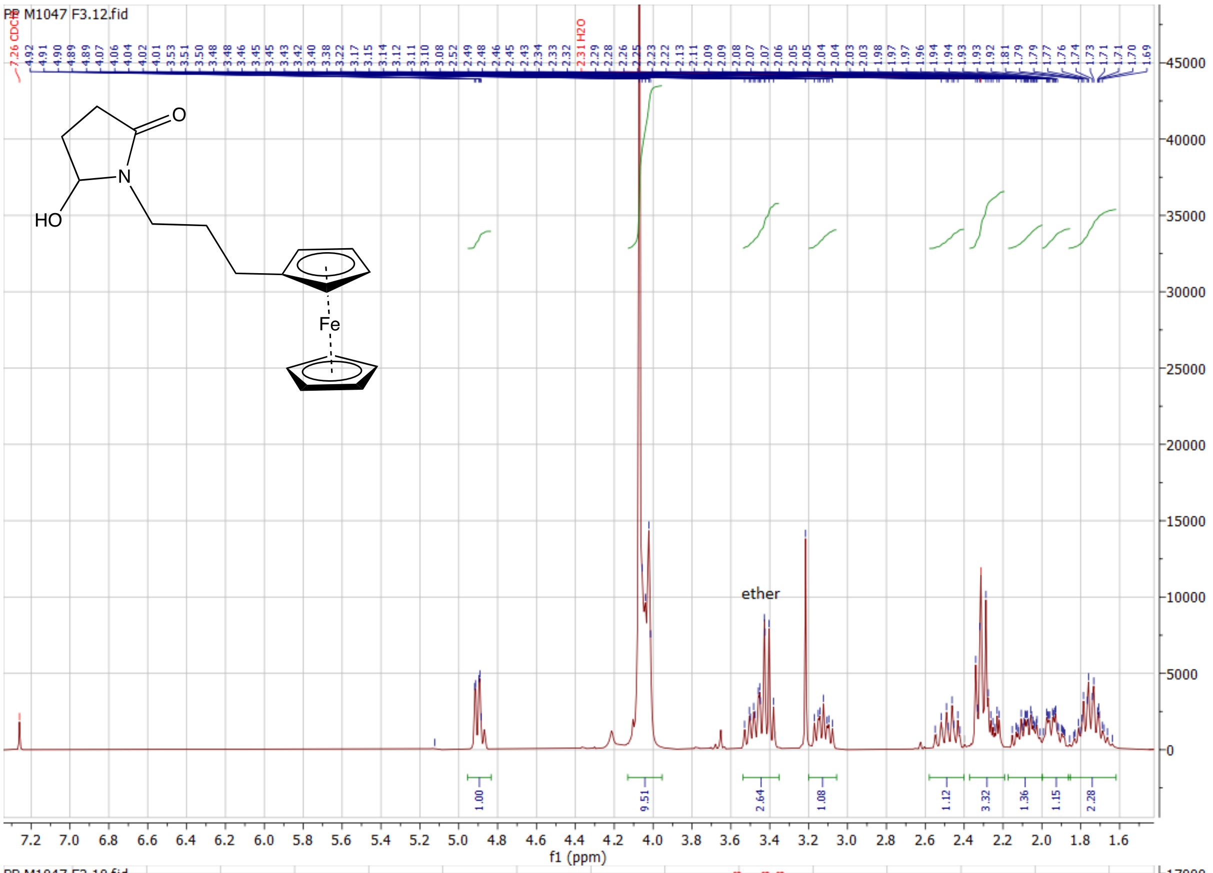

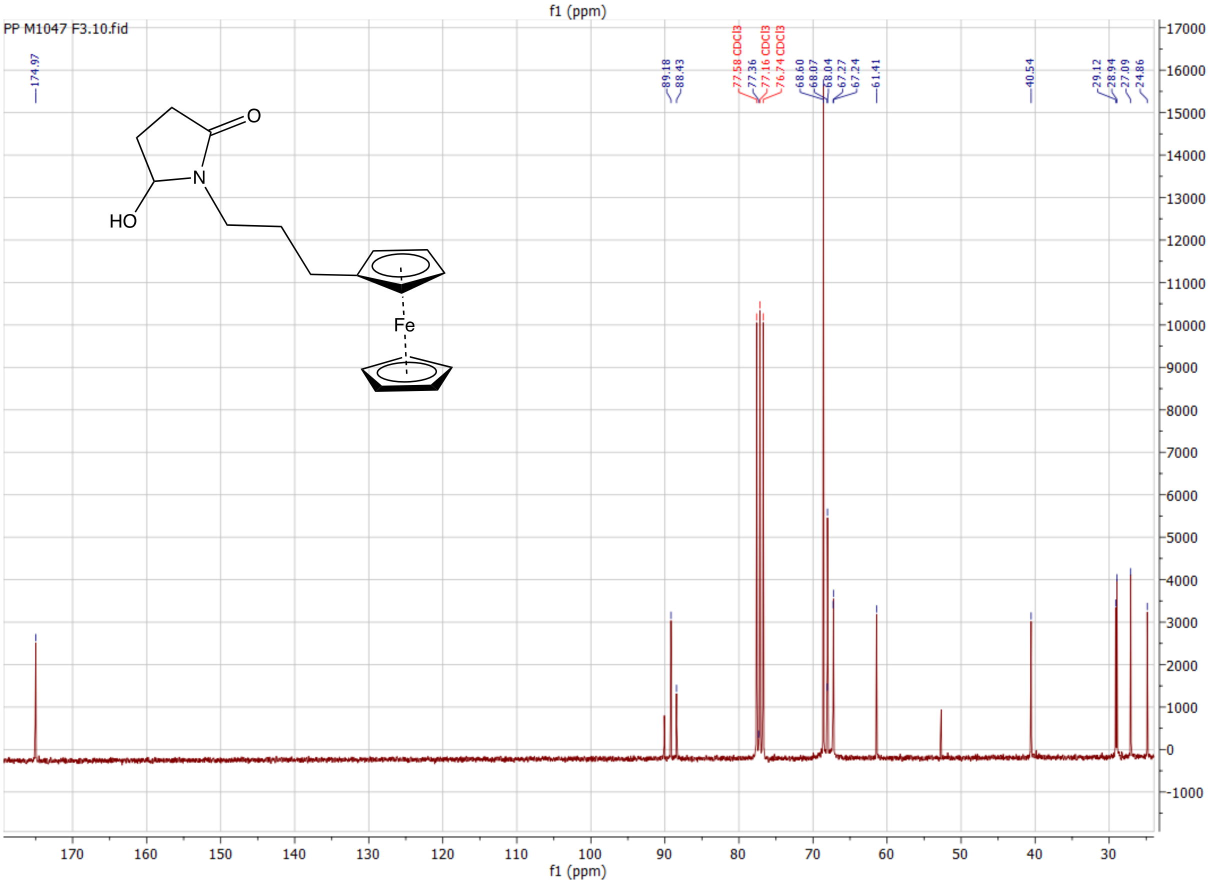

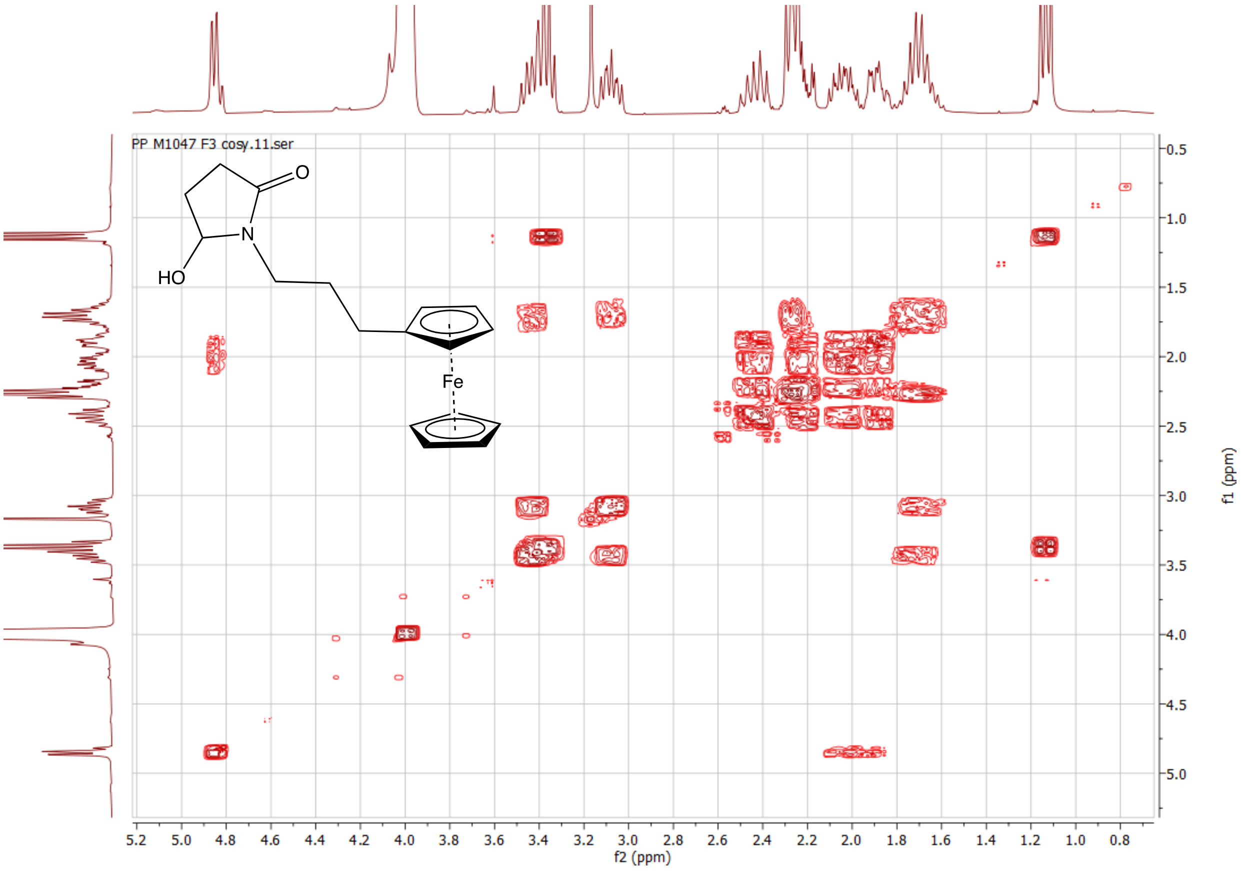

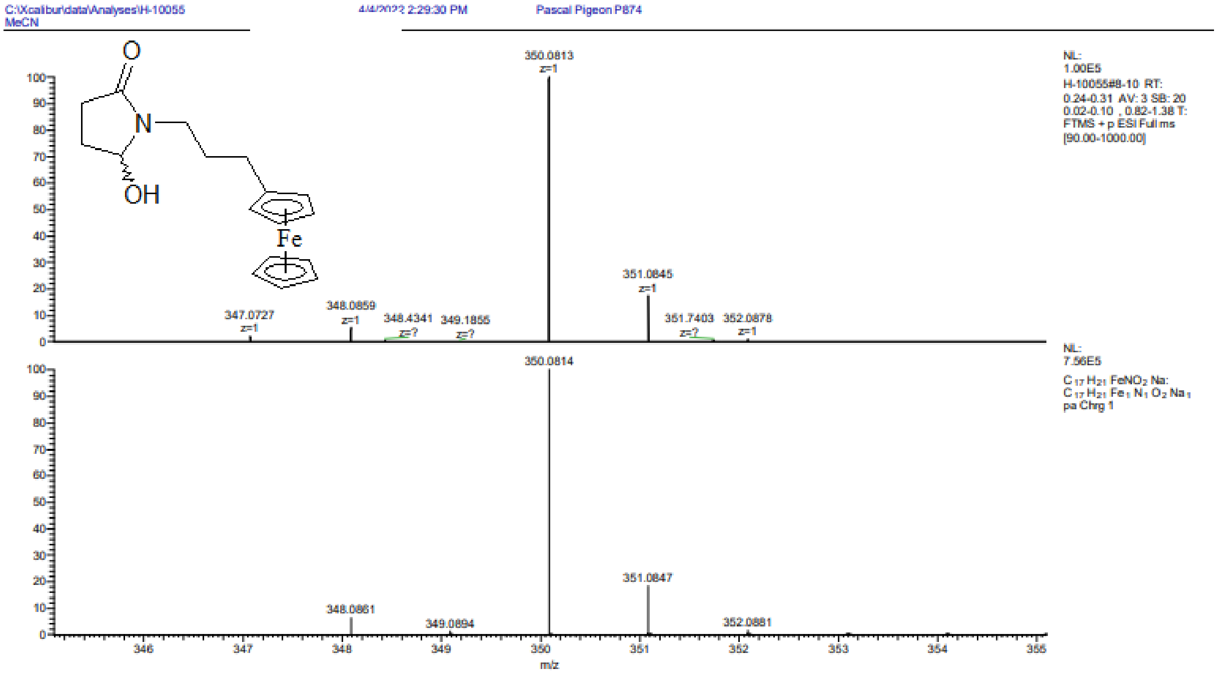

Experimental/theoretical isotopic pattern MS spectrum

Error = -0.2 ppm; Relative Intensity (%) 100

HRMS (ESI) m/z: [M+Na]+ Calcd for C17H21FeNO2Na 350.0814. Found 350.0813; (Error: -0.2 ppm).

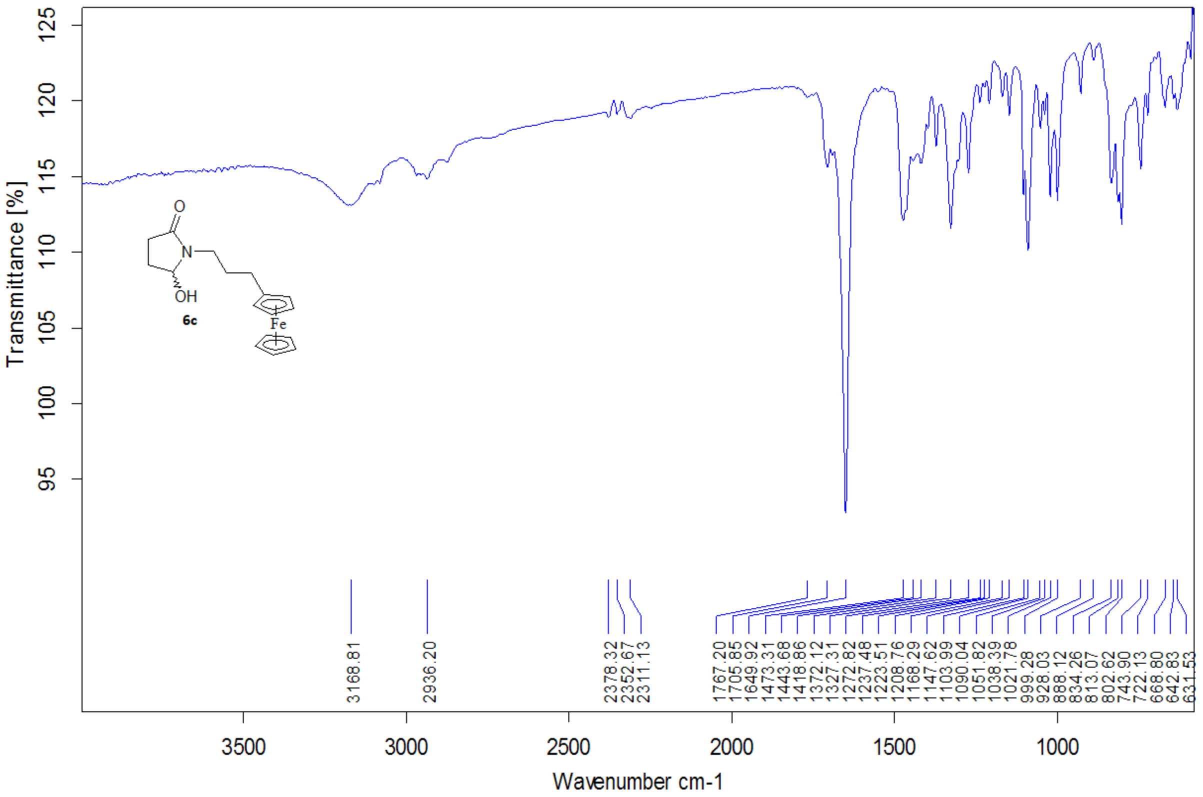

Transmittance [%]
125
120
115
110
105
100
95
O
N
OH
6c
Fe
3168.81
2936.20
2378.32
2352.67
2311.13
1767.20
1705.85
1649.92
1473.31
1443.68
1418.86
1372.12
1327.31
1272.82
1237.48
1223.51
1208.76
1168.29
1147.62
1103.99
1090.04
1051.82
1038.39
1021.78
999.28
928.03
888.12
834.26
813.07
802.62
743.90
722.13
668.80
642.83
631.53
3500
3000
2500
2000
1500
1000
Wavenumber cm-1

Figure S22: ^{1}H (in DMSO-d6), ^{13}C (in DMSO-d6) NMR and IR data for compound **6e**

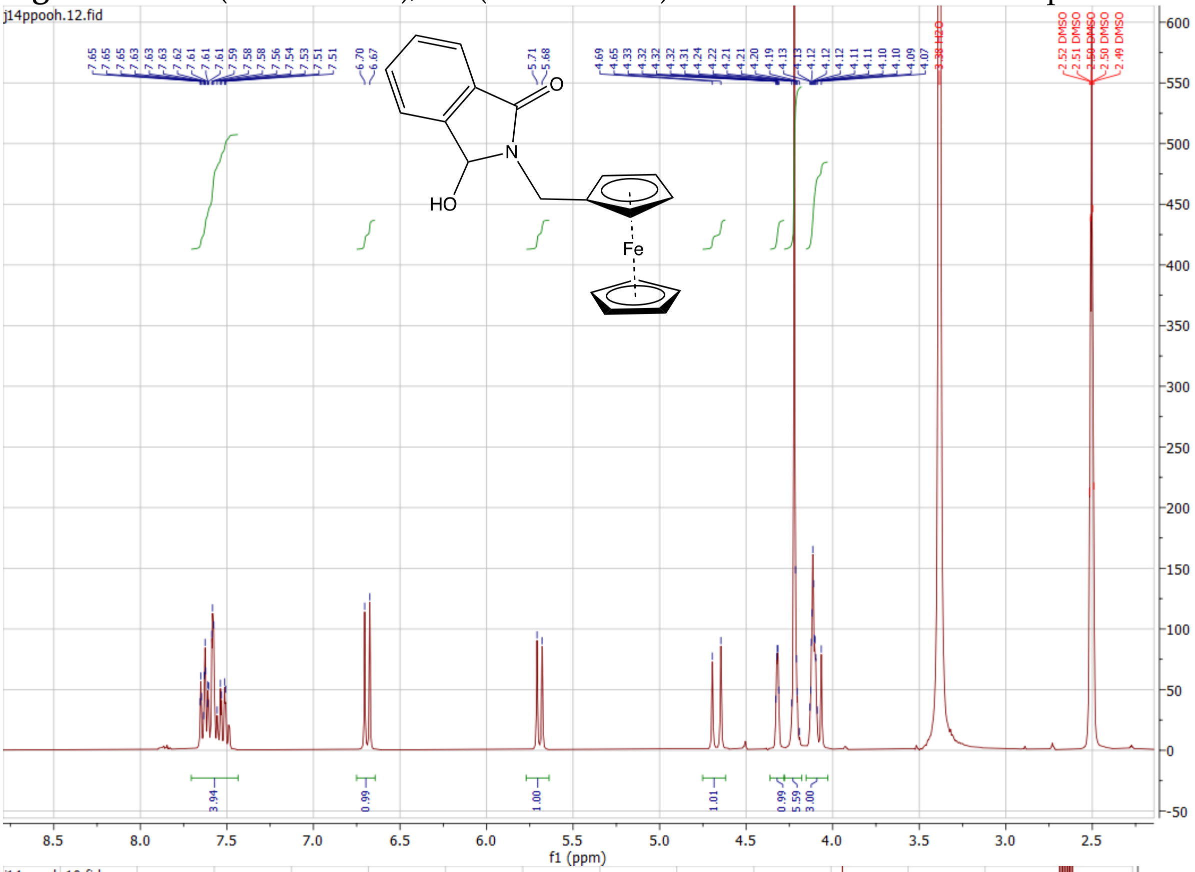

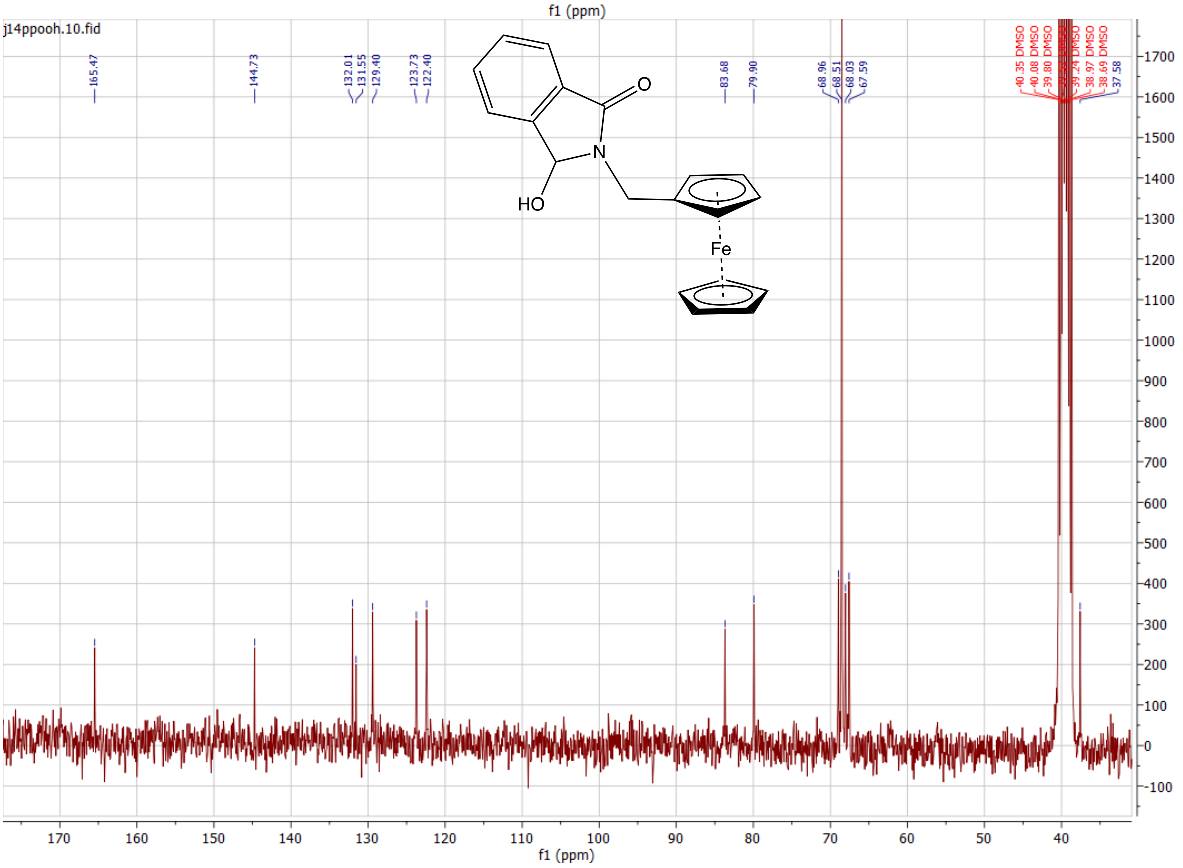

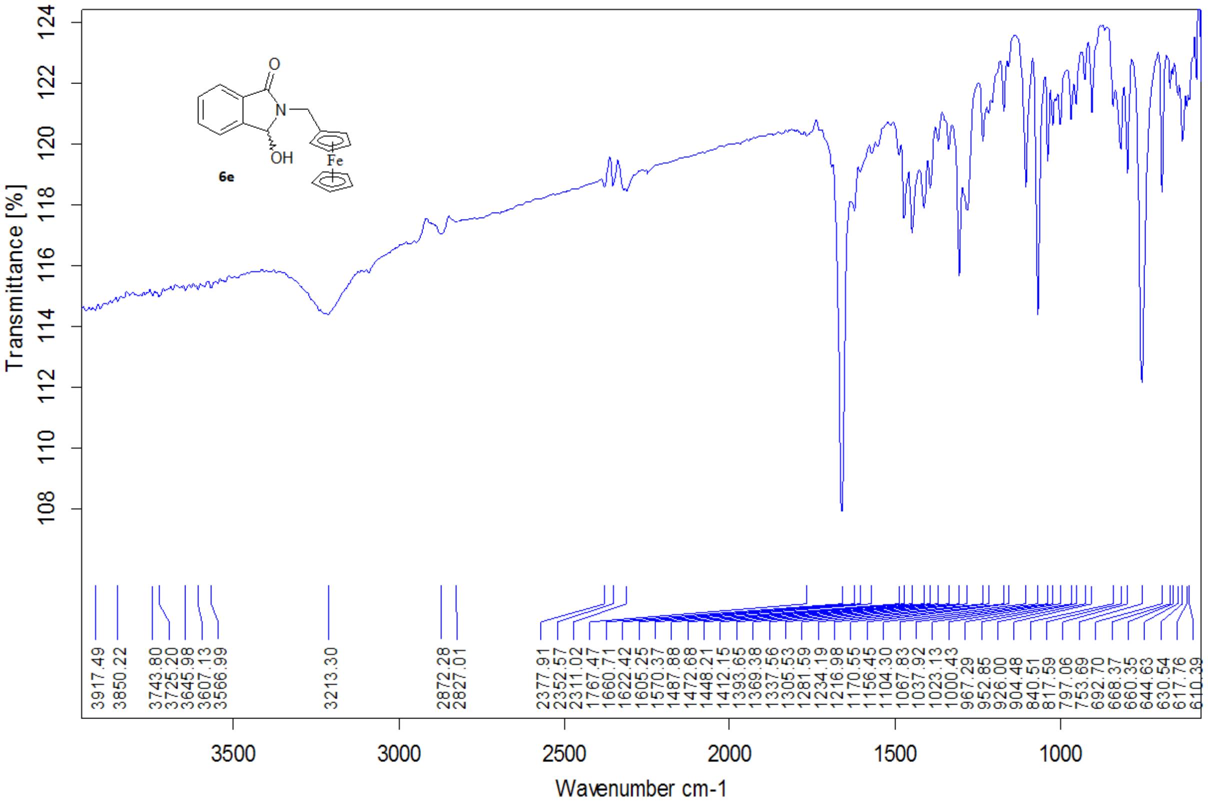

Transmittance [%]
Wavenumber cm-1
6e
OH
N
O
Fe
3917.49
3850.22
3743.80
3725.20
3645.98
3607.13
3566.99
3213.30
2872.28
2827.01
2377.91
2352.57
2311.02
1767.47
1660.71
1622.42
1605.25
1570.37
1487.88
1472.68
1448.21
1412.15
1393.65
1369.38
1337.56
1305.53
1281.59
1234.19
1216.98
1170.55
1156.45
1104.30
1067.83
1037.92
1023.13
1000.43
967.29
952.85
926.00
904.48
840.51
817.59
797.06
753.69
692.70
668.37
660.35
644.63
630.54
617.76
610.39

Figure S23: ^{1}H (in DMSO-d_6), ^{13}C (in DMSO-d_6) NMR, HR-MS and IR data for compound **6f**

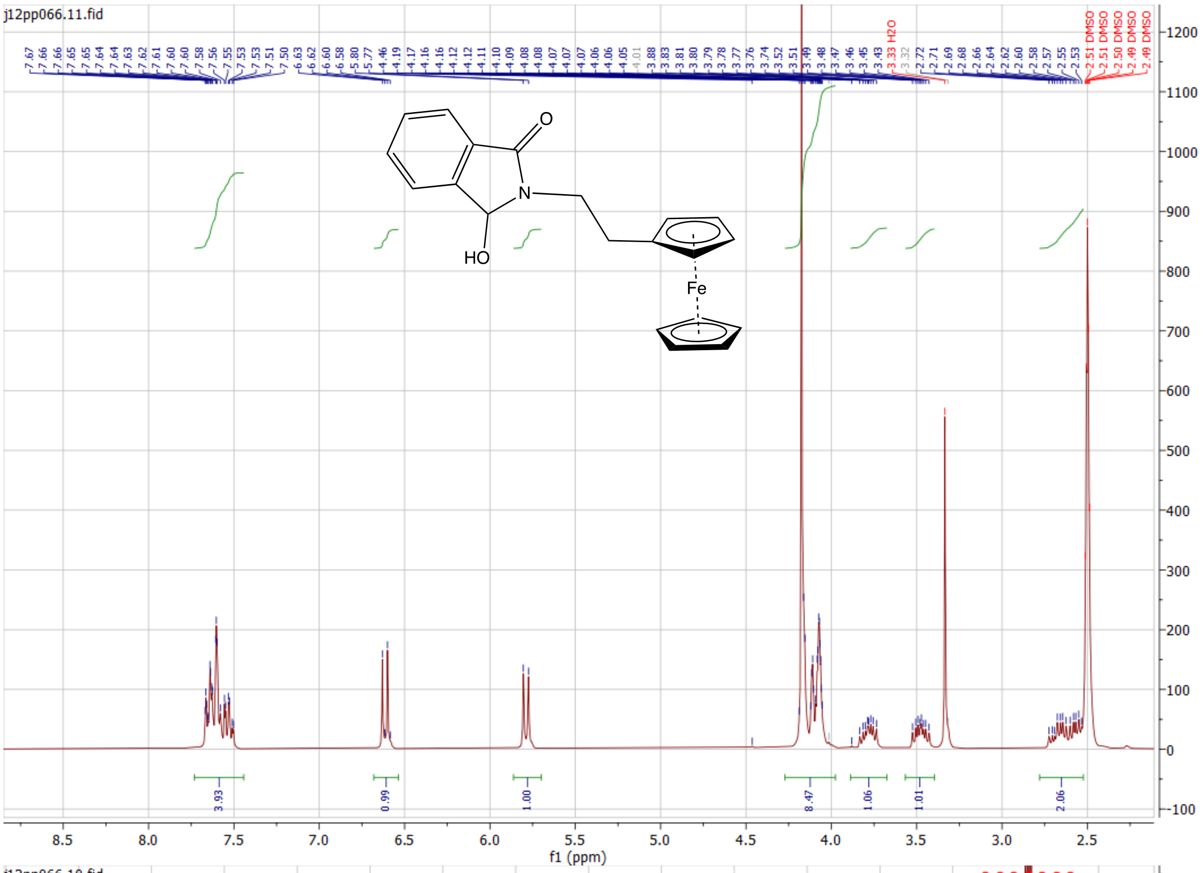

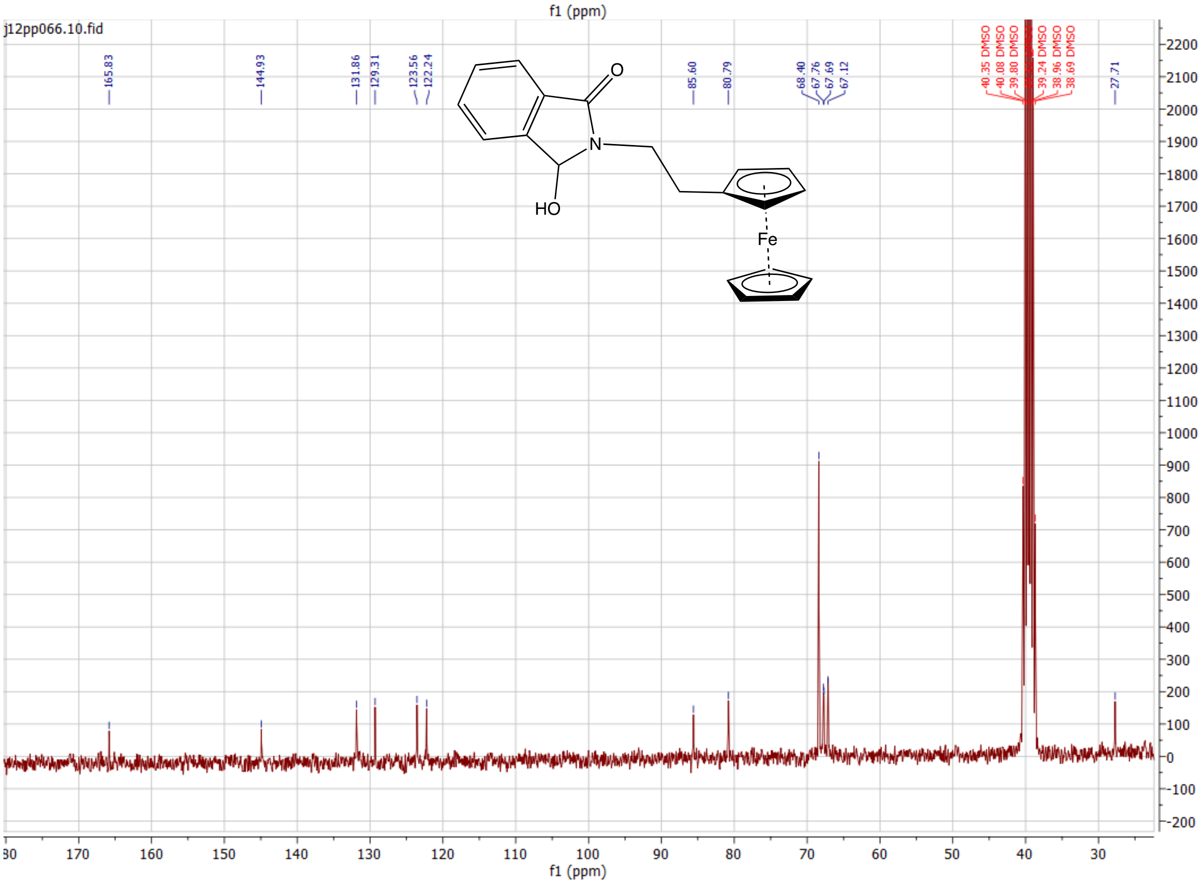

Single Mass Analysis
Tolerance = 5.0 PPM / DBE: min = -1.5, max = 100.0
Element prediction: Off
Number of isotope peaks used for i-FIT = 9

Monoisotopic Mass, Odd Electron Ions
152 formula(e) evaluated with 1 results within limits (all results (up to 1000) for each mass)
Elements Used:
C: 1-150 H: 1-150 N: 0-3 O: 0-10 Fe: 1-1

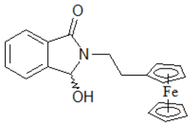

19-Jun-2013 2::5::3
ENSCP_P677 37 (0.932) Cm (31:44)

ACN

LCT Premier XE KE483
1: TOF MS ES+
6.17e+004

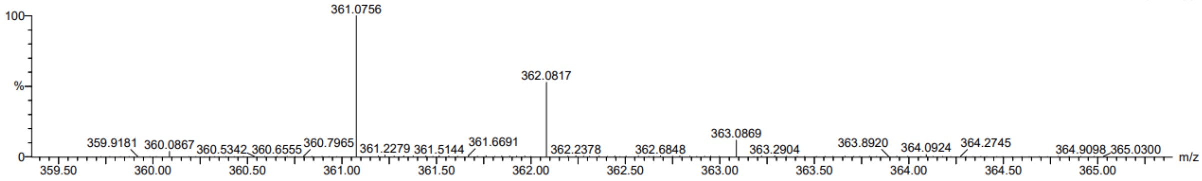

Mass	Calc. Mass	mDa	PPM	DBE	i-FIT	i-FIT (Norm)	Formula
Minimum:				-1.5			
Maximum:		5.0	5.0	100.0			
361.0756	361.0765	-0.9	-2.5	12.0	751.7	0.0	C20 H19 N O2 Fe

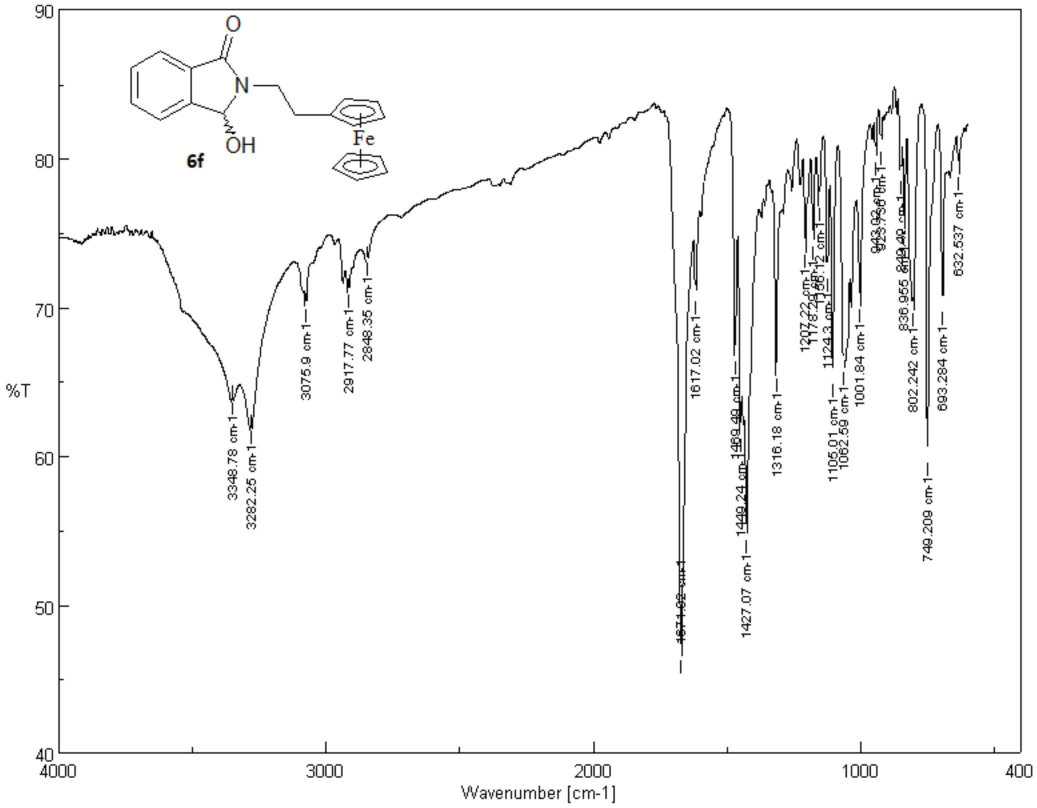

Figure S24: ^{1}H (in DMSO-d$_6$), ^{13}C (in DMSO-d$_6$) NMR, HR-MS and IR data for compound **6g**

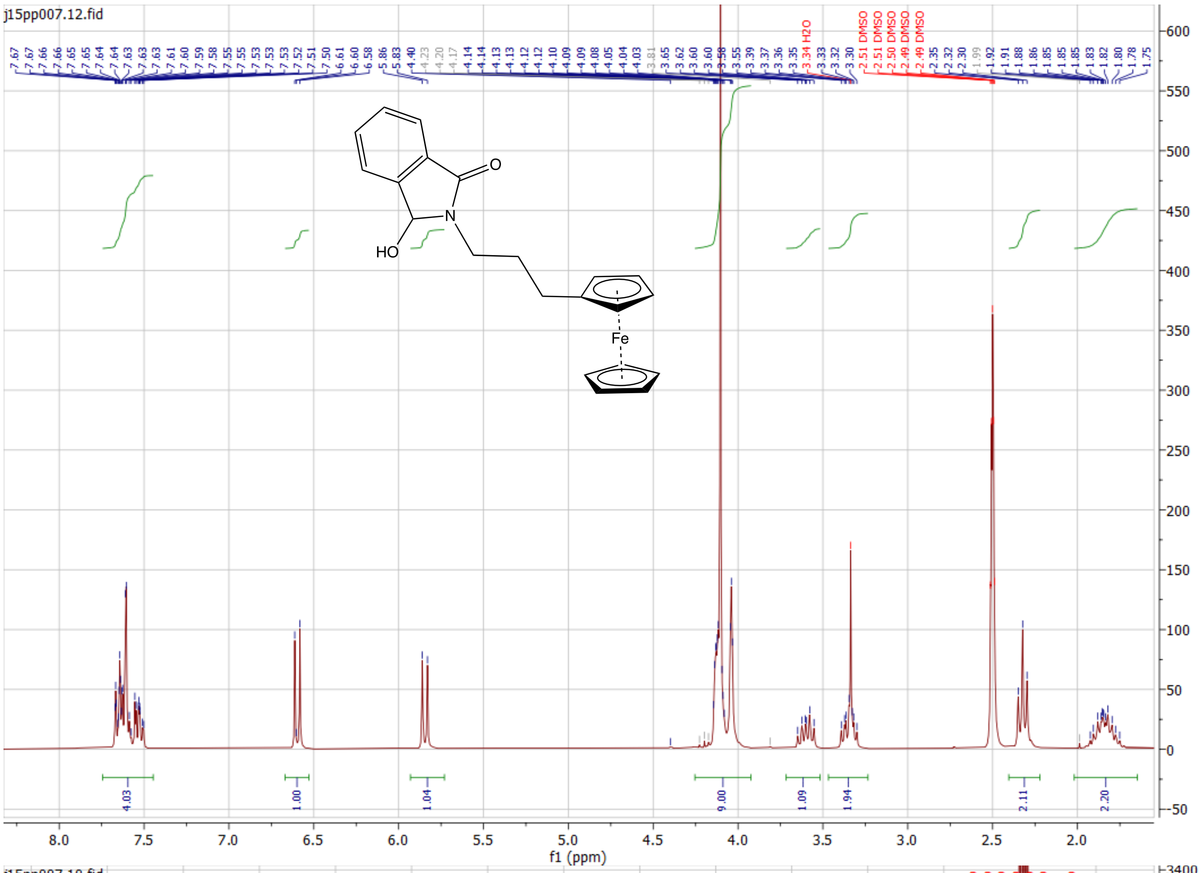

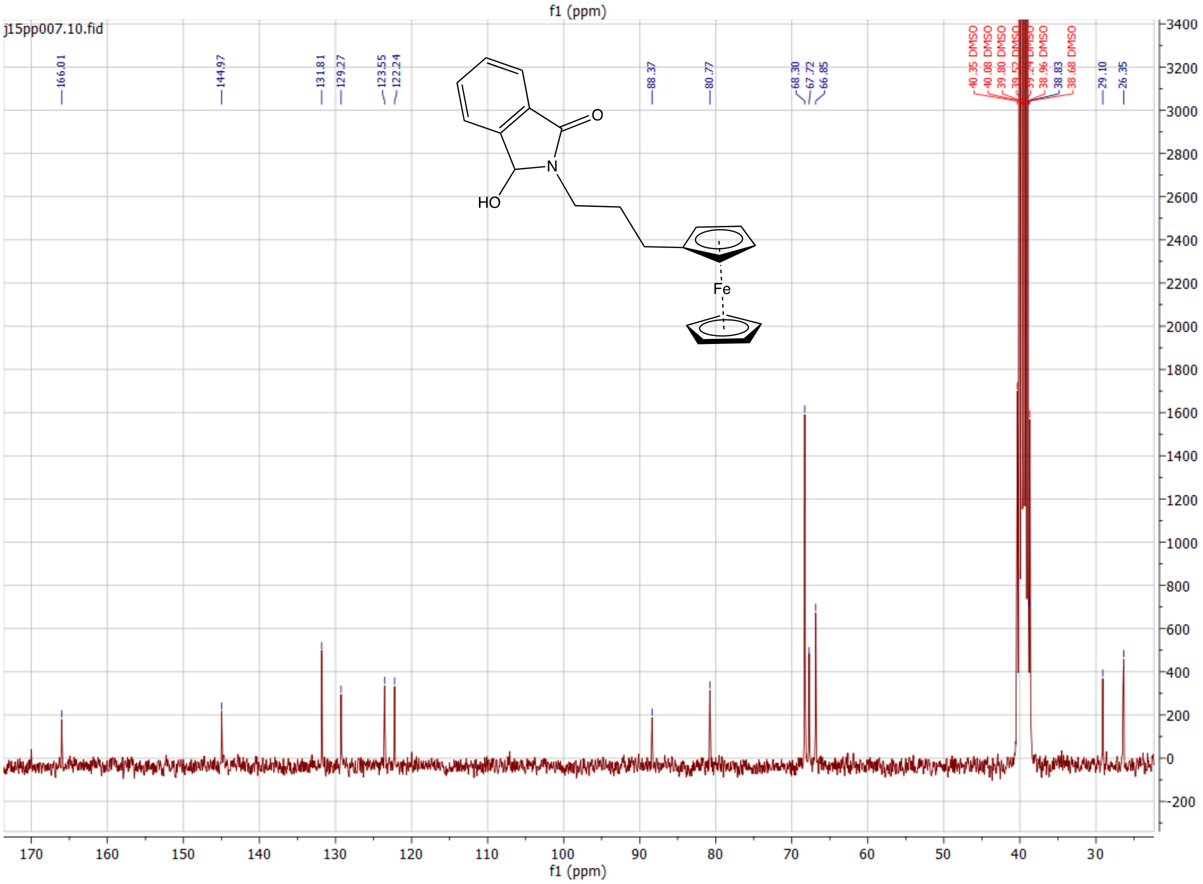

Single Mass Analysis
Tolerance = 5.0 PPM / DBE: min = -1.5, max = 100.0
Element prediction: Off
Number of isotope peaks used for i-FIT = 9

Monoisotopic Mass, Odd Electron Ions
160 formula(e) evaluated with 2 results within limits (all results (up to 1000) for each mass)
Elements Used:
C: 1-150 H: 1-150 N: 0-3 O: 0-10 Fe: 1-1

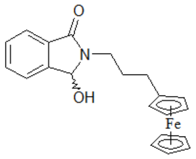

19-Jun-2013 3::0::1
ENSCP_P678 49 (1.206) Cm (48:58)

ACN

LCT Premier XE KE483
1: TOF MS ES+
1.26e+004

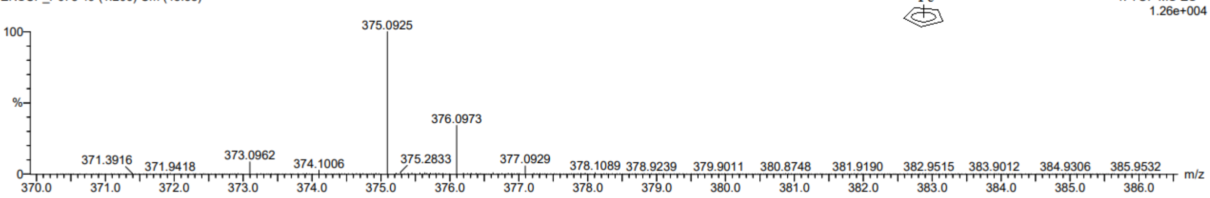

Minimum:				-1.5			
Maximum:		5.0	5.0	100.0			
Mass	Calc. Mass	mDa	PPM	DBE	i-FIT	i-FIT (Norm)	Formula
375.0925	375.0922	0.3	0.8	12.0	788.0	0.0	C21 H21 N O2 Fe
	375.0940	-1.5	-4.0	-1.0	795.2	7.2	C9 H25 N3 O9 Fe

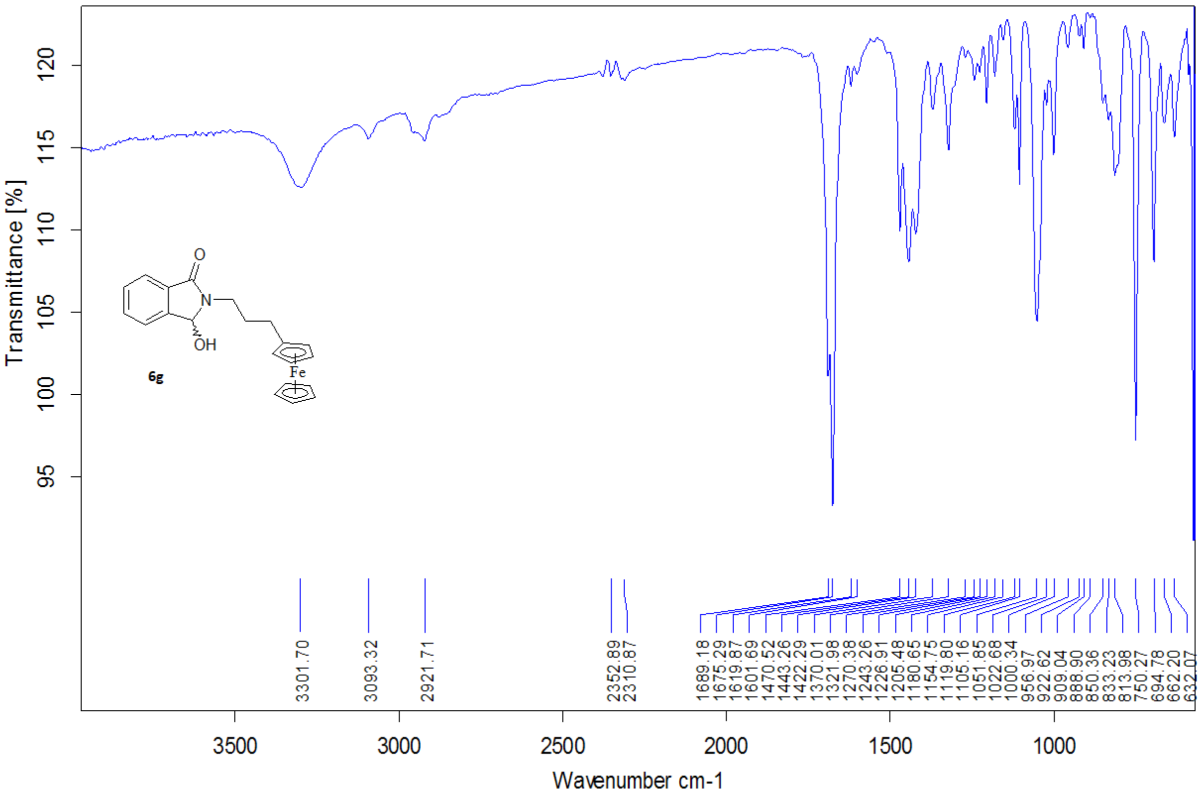

Figure S25: ^{1}H (in DMSO-d6) and ^{13}C (in DMSO-d6) NMR and HR-MS data for compound **6h**

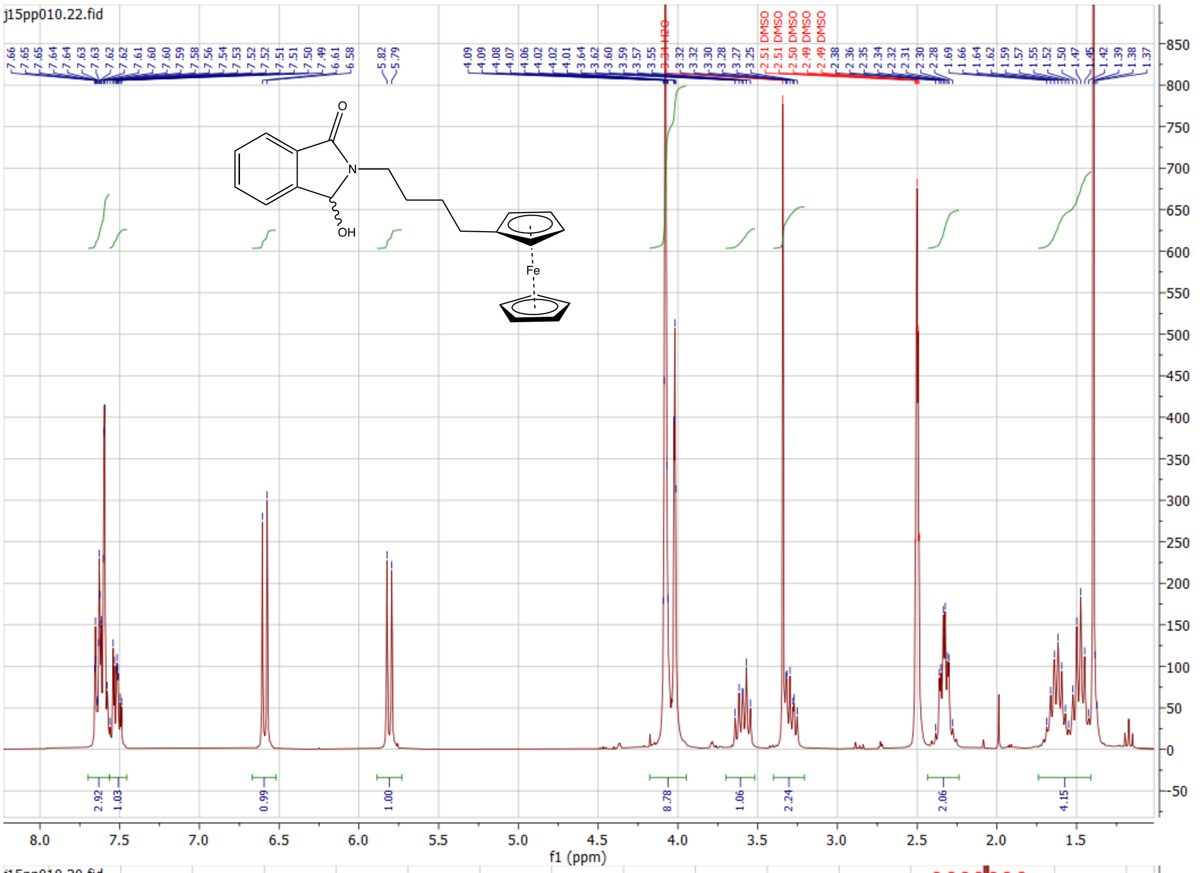

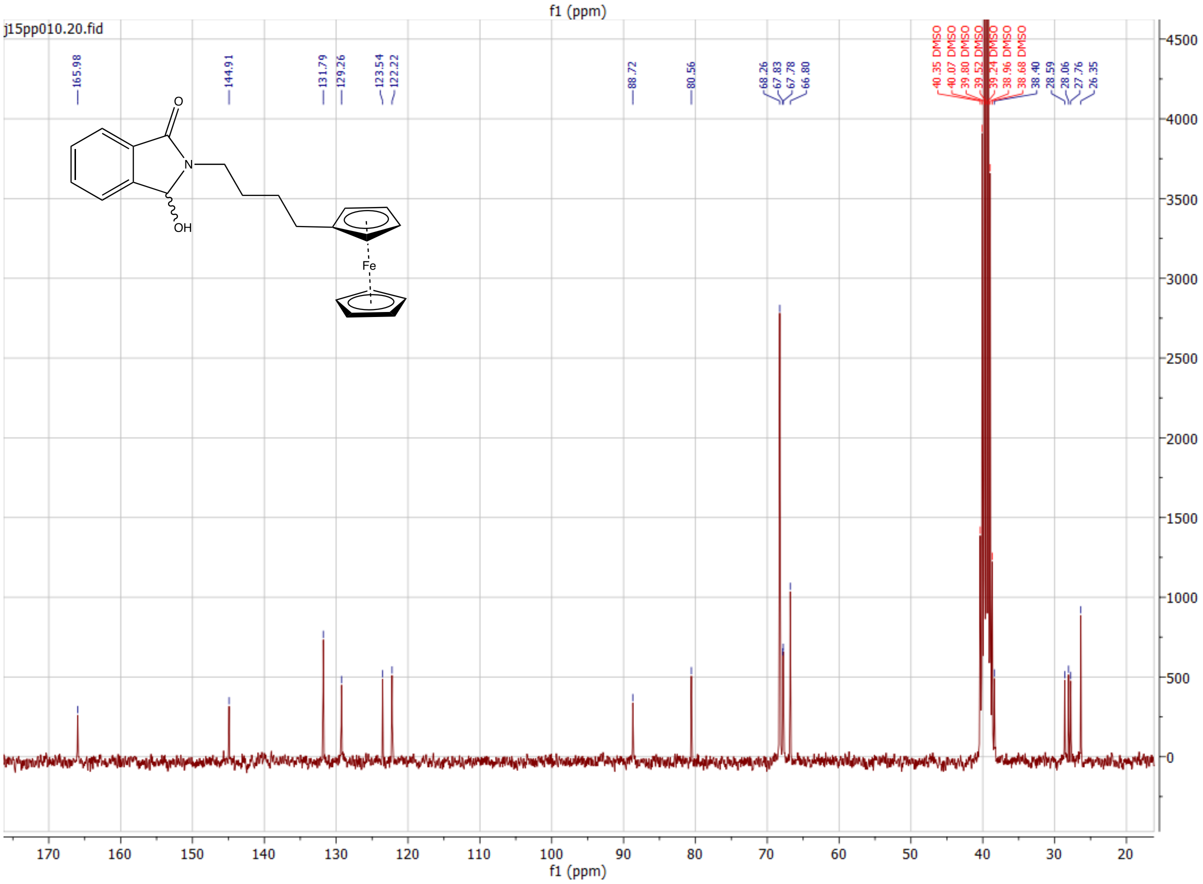

Single Mass Analysis
Tolerance = 5.0 PPM / DBE: min = -1.5, max = 100.0
Element prediction: Off
Number of isotope peaks used for i-FIT = 9

Monoisotopic Mass, Odd Electron Ions
412 formula(e) evaluated with 3 results within limits (all results (up to 1000) for each mass)
Elements Used:
C: 0-100 H: 1-120 N: 0-10 O: 0-10 Fe: 1-1

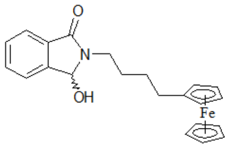

06-Sep-2013 5::7::4
ENSCP_P679 29 (0.753) Cm (29:39)

MeOH

LCT Premier XE KE483
1: TOF MS ES+
2.55e+004

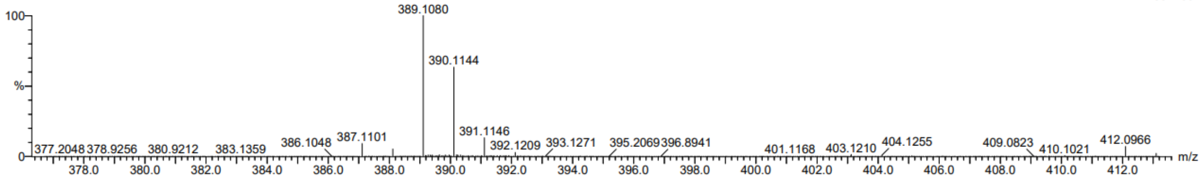

Minimum:				-1.5			
Maximum:		5.0	5.0	100.0			
Mass	Calc. Mass	mDa	PPM	DBE	i-FIT	i-FIT (Norm)	Formula
389.1080	389.1078	0.2	0.5	12.0	1020.1	0.0	C22 H23 N O2 Fe
	389.1097	-1.7	-4.4	-1.0	1023.3	3.2	C10 H27 N3 O9 Fe
	389.1070	1.0	2.6	0.0	1026.1	6.1	C6 H23 N9 O7 Fe

Figure S26: ^{1}H (in DMSO-d_6), ^{13}C (in DMSO-d_6) NMR, HR-MS and IR data for compound **6i**

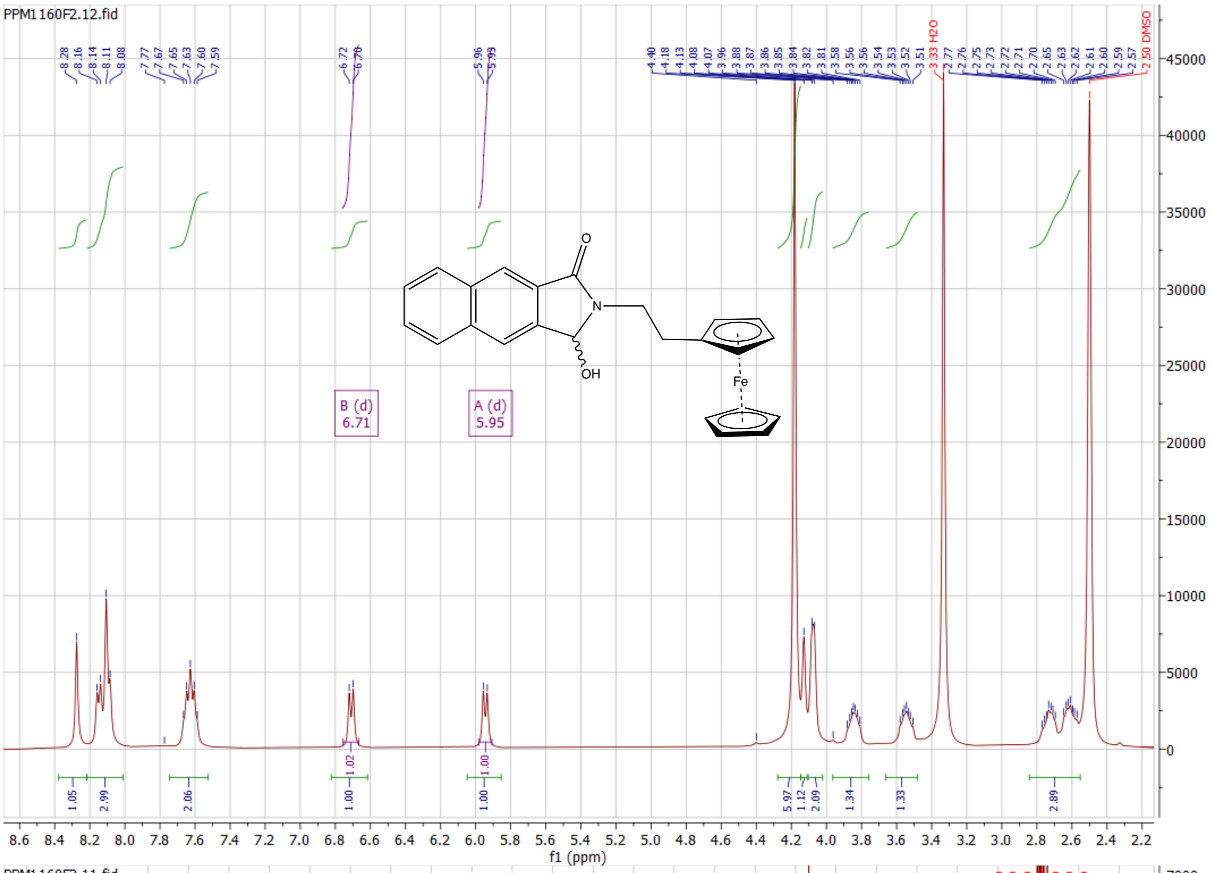

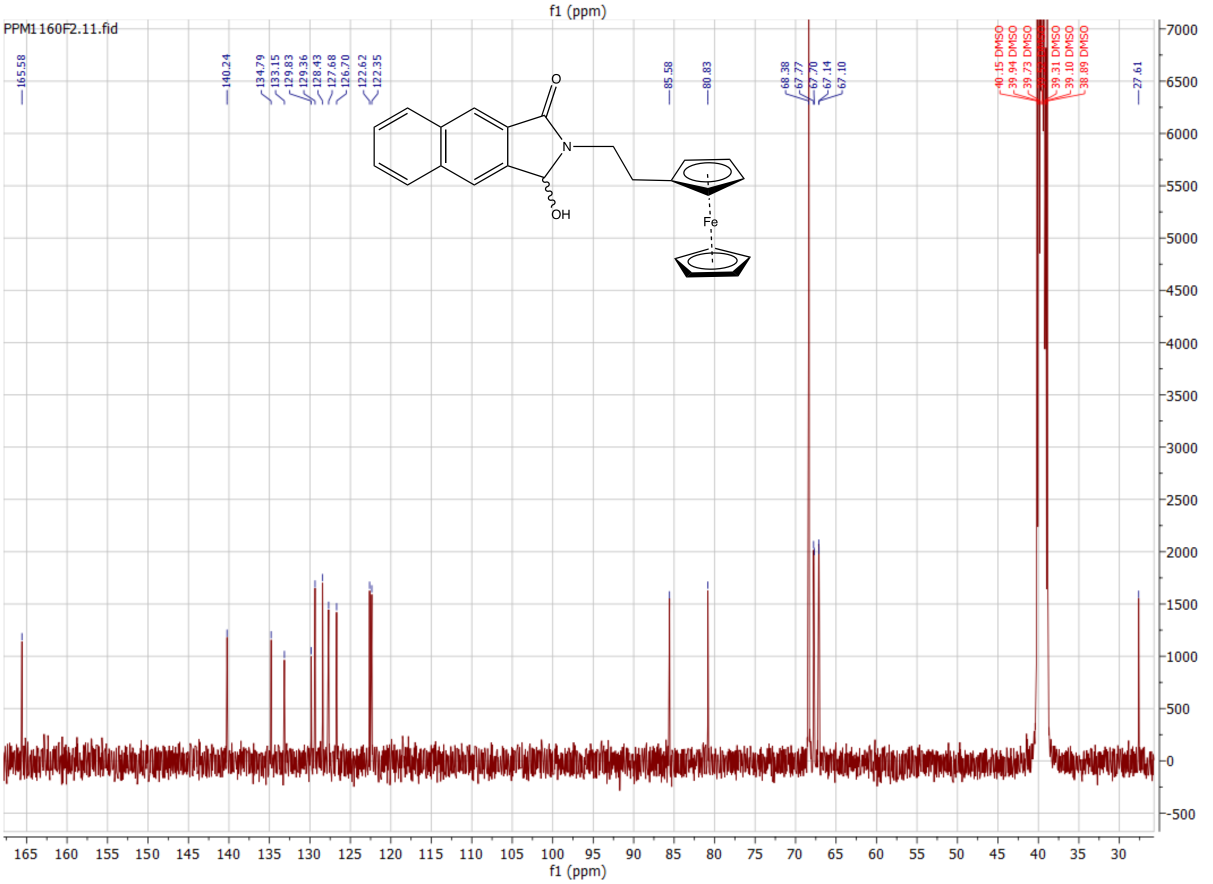

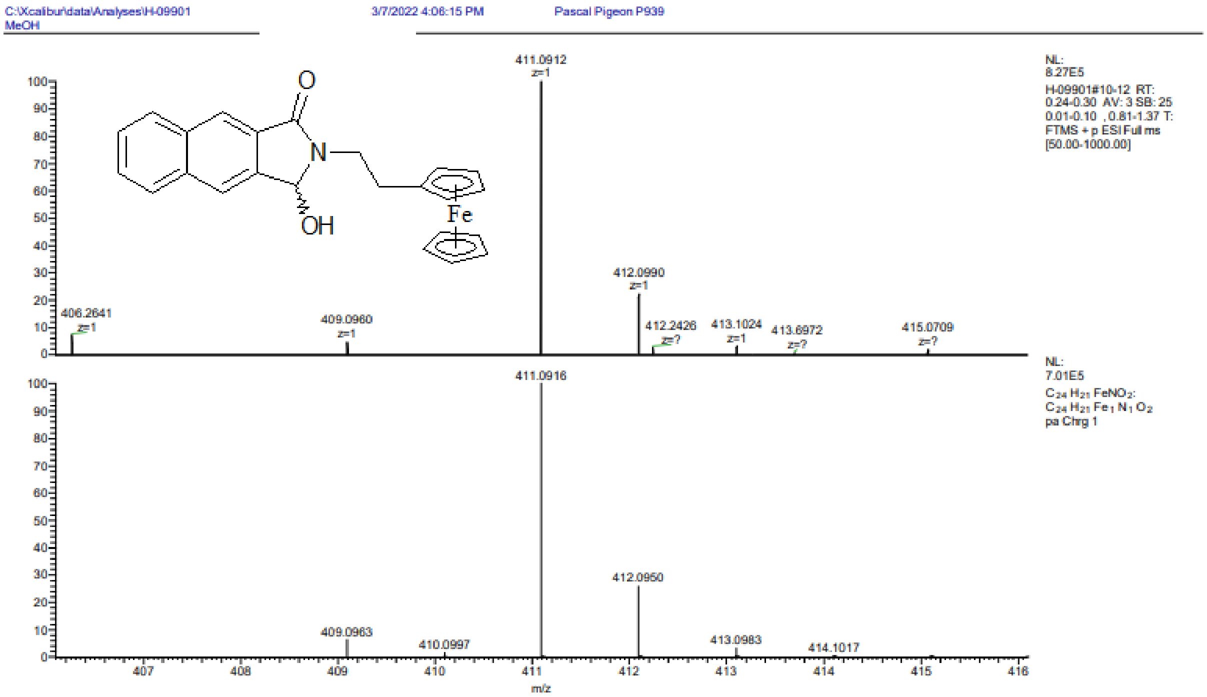

Experimental/theoretical isotopic pattern MS spectrum

Error = -1.0 ppm; Relative Intensity (%) 100

HRMS (ESI) m/z: [M]+ Calcd for C24H21FeNO2 411.0916 . Found 411.0912; (Error: -1.0 ppm).

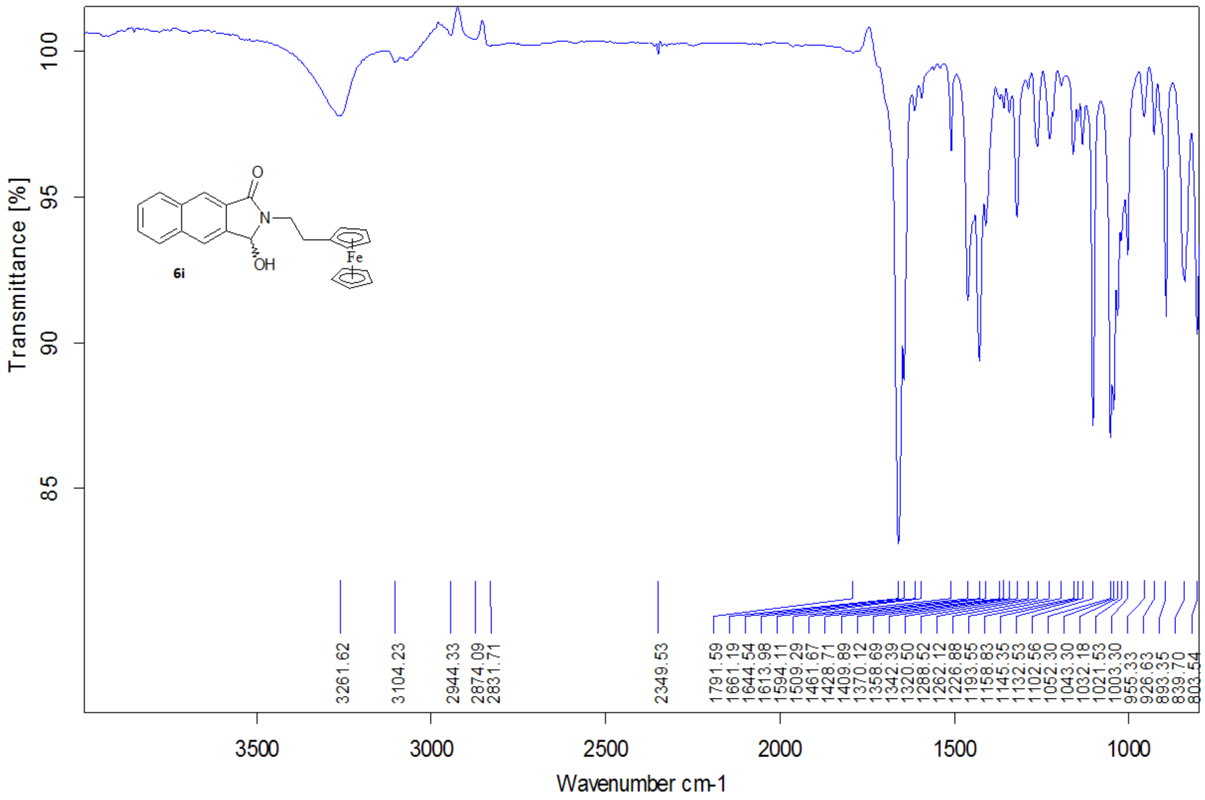

Figure S27: ^{1}H (in $CDCl_3$), COSY (in $CDCl_3$) NMR data for compound **6j**

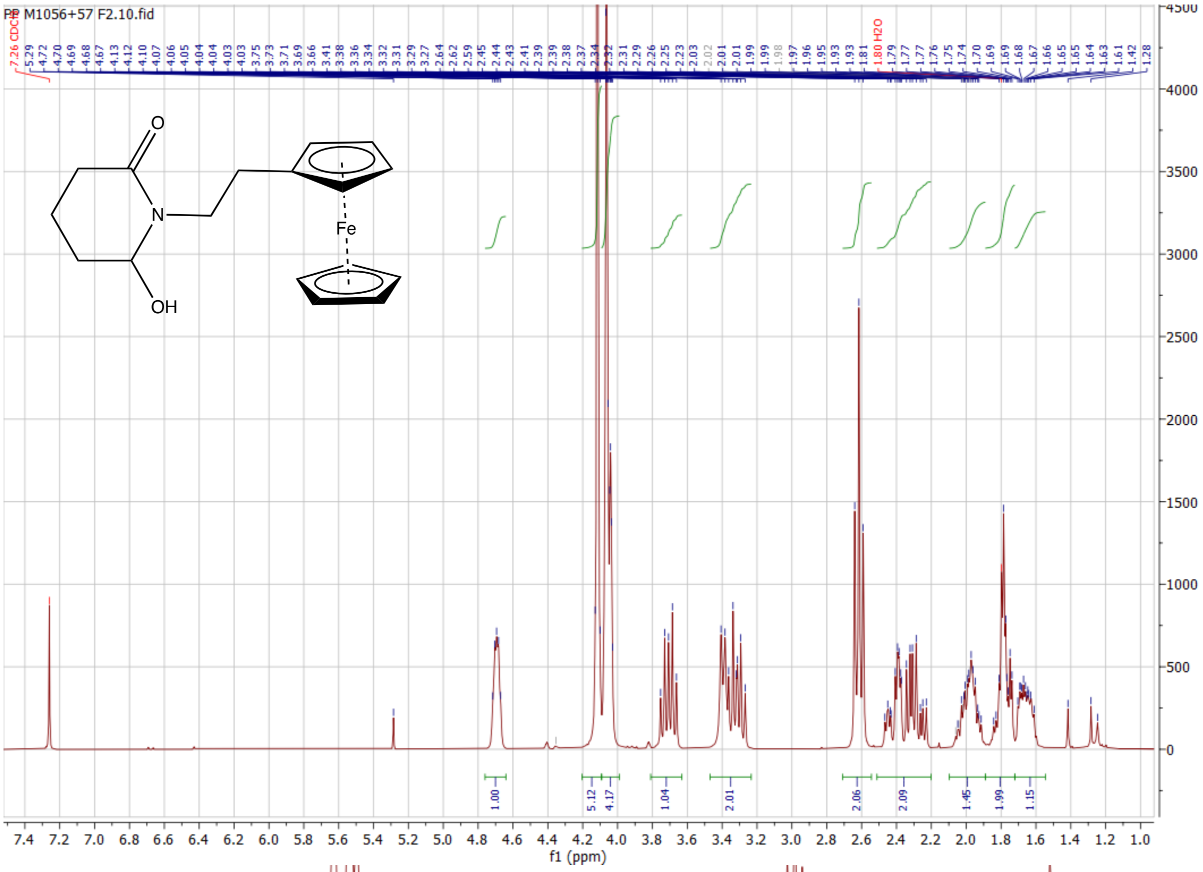

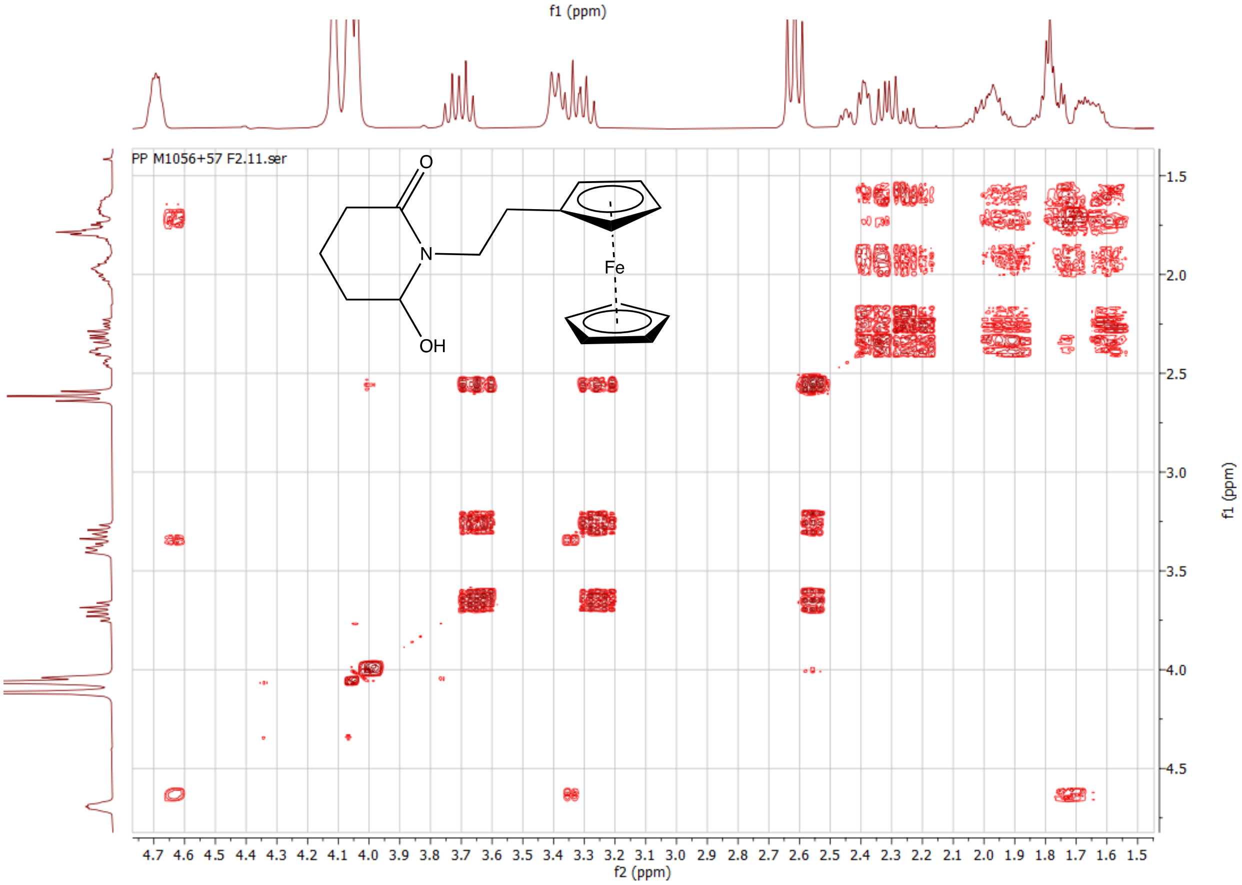

Figure S28: ^{1}H (in $CDCl_3$), ^{13}C (in $CDCl_3$) NMR and IR data for compound **7j**

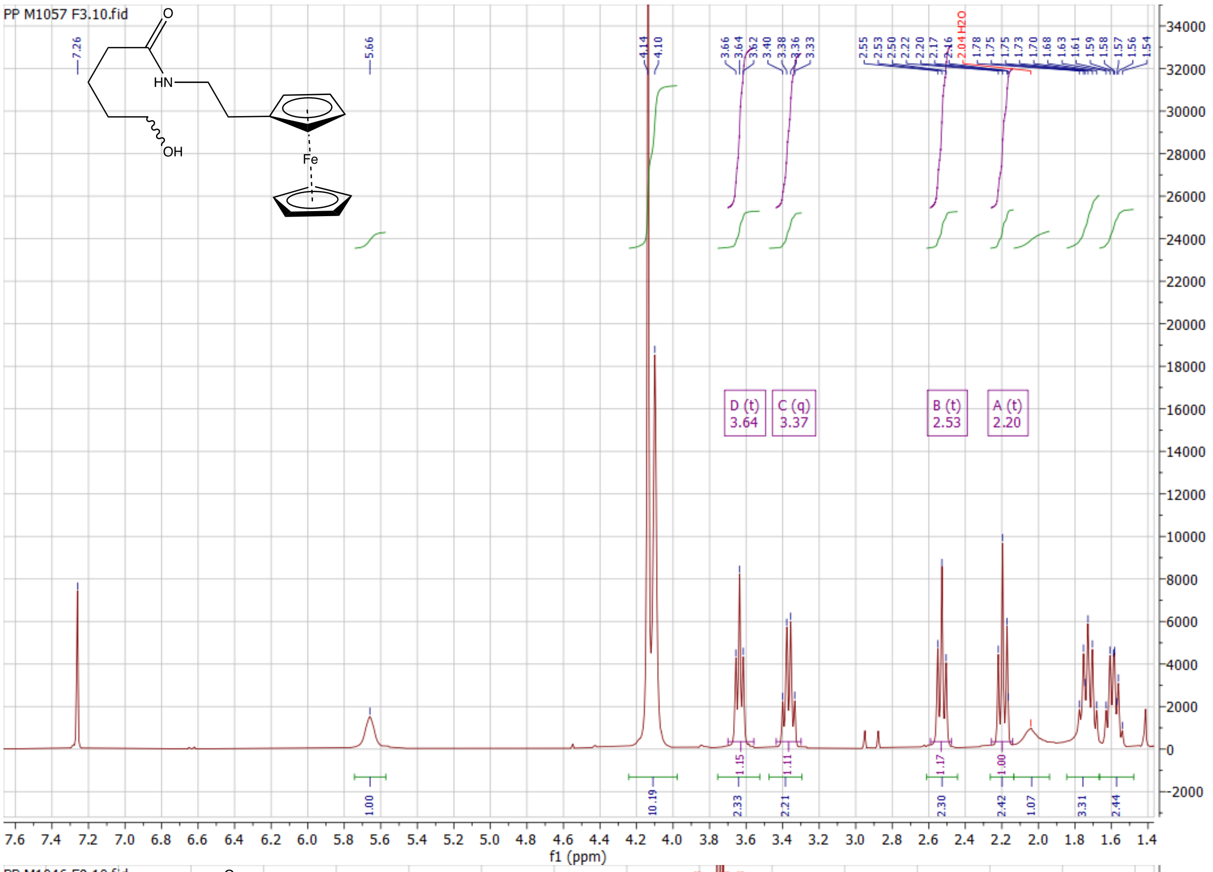

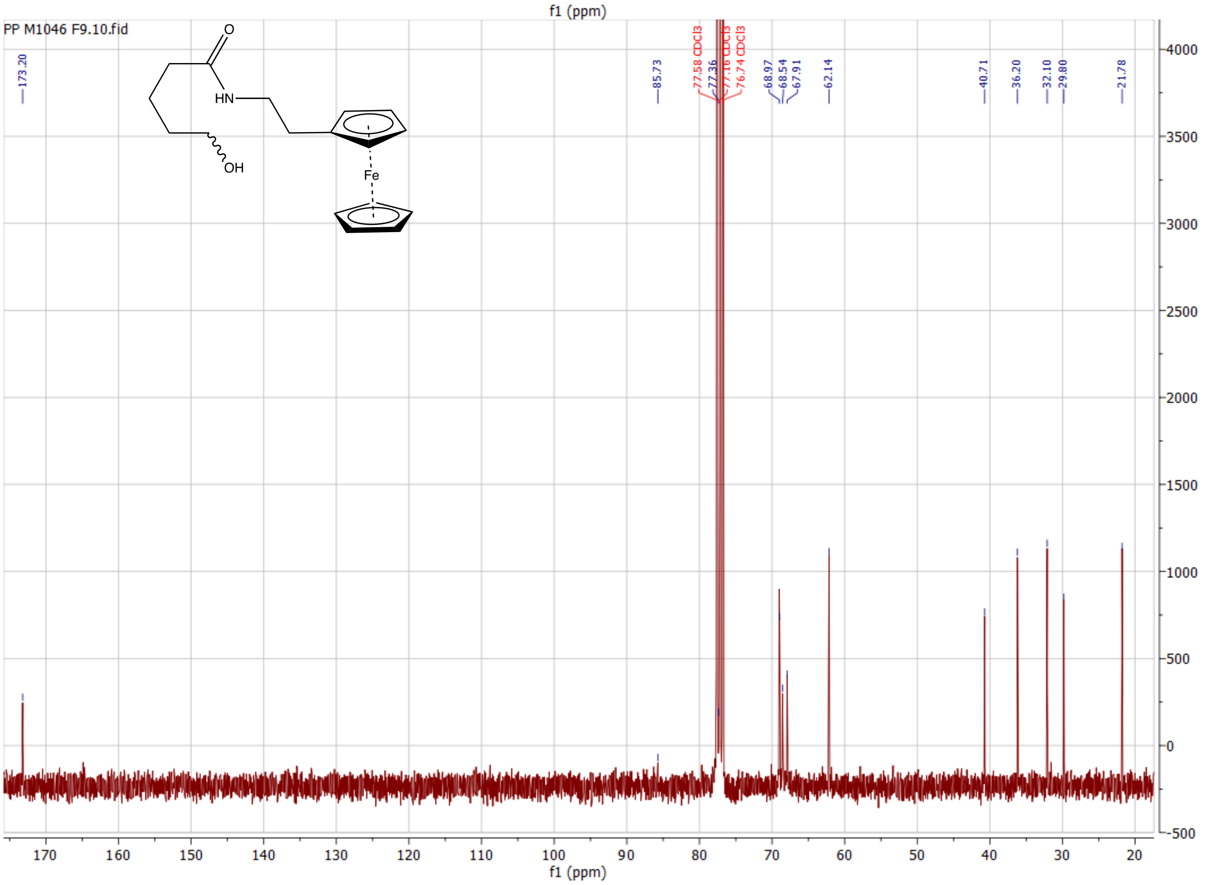

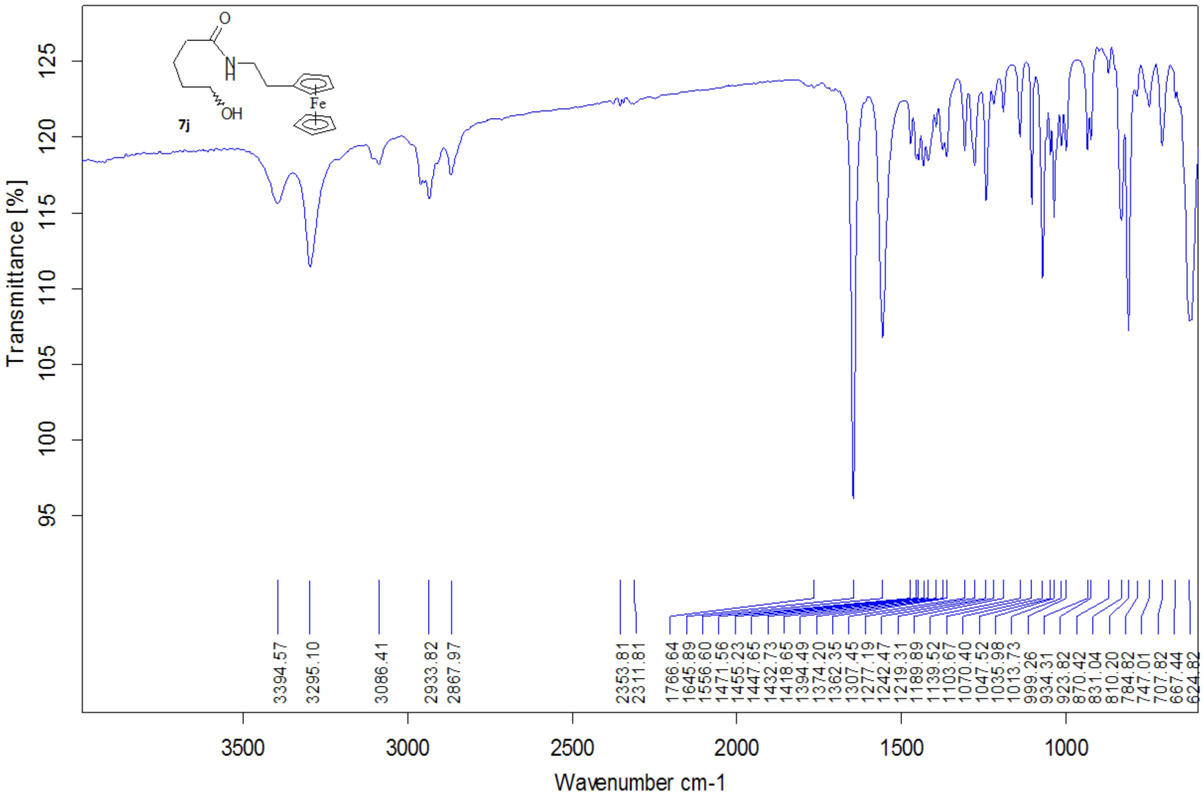

O
N
H
Fe
OH
7j
Transmittance [%]
125
120
115
110
105
100
95
3394.57
3295.10
3086.41
2933.82
2867.97
2353.81
2311.81
1766.64
1645.89
1556.60
1471.56
1455.23
1447.65
1432.73
1418.65
1394.49
1374.20
1362.35
1307.45
1277.19
1242.47
1219.31
1189.89
1139.52
1103.67
1070.40
1047.52
1035.98
1013.73
999.26
934.31
923.82
870.42
831.04
810.20
784.82
747.01
707.82
667.44
624.82
3500
3000
2500
2000
1500
1000
Wavenumber cm-1

Figure S29: ^{1}H (in $CDCl_3$), ^{13}C (in $CDCl_3$), COSY (in $CDCl_3$), HMQC (in $CDCl_3$) NMR and IR data for compound **8j**

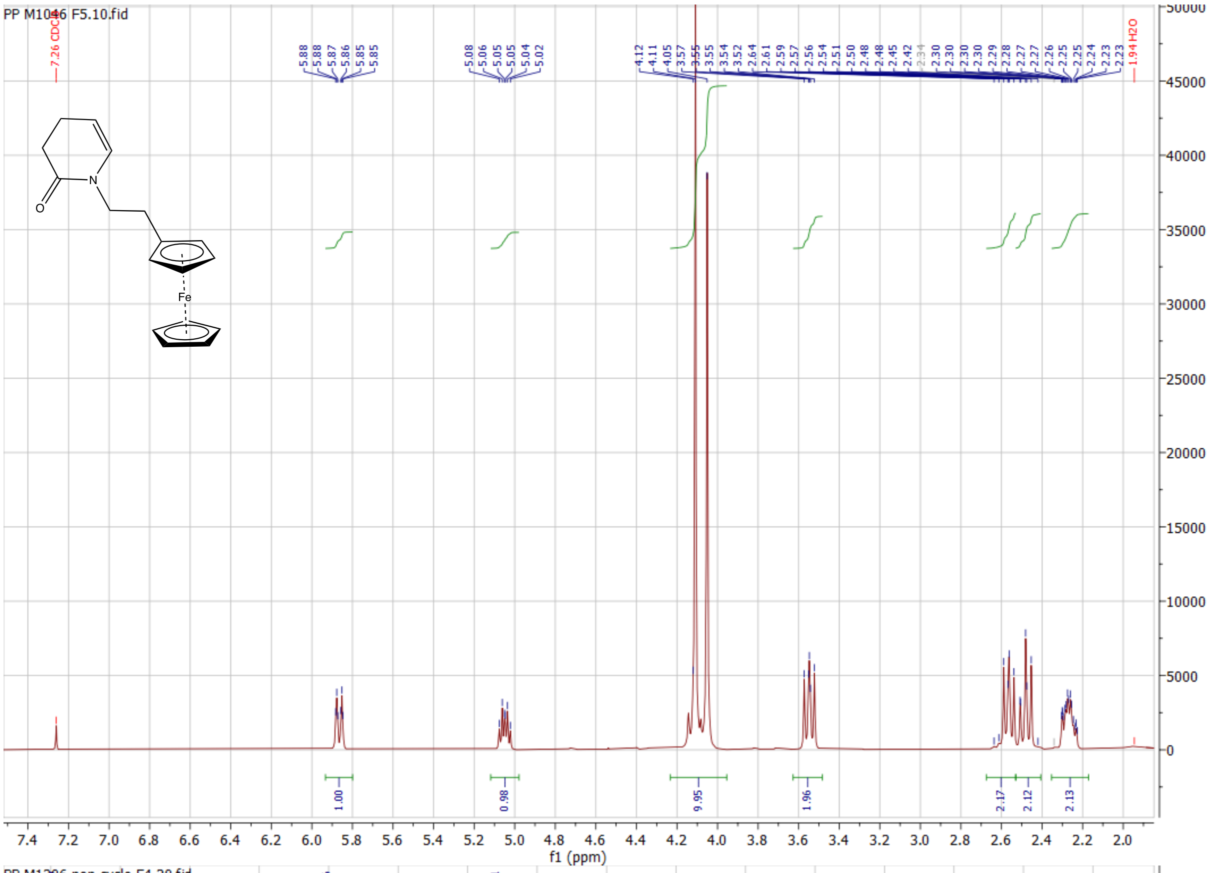

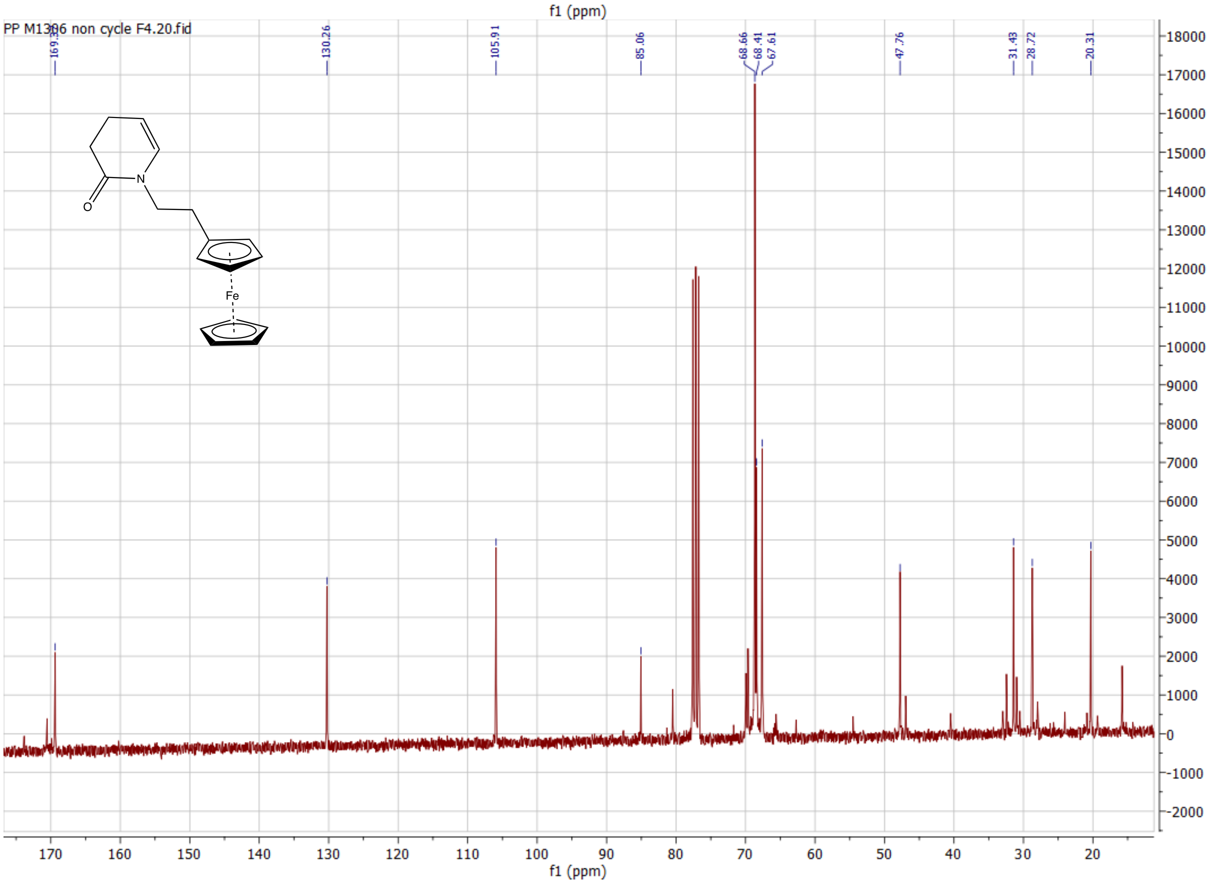

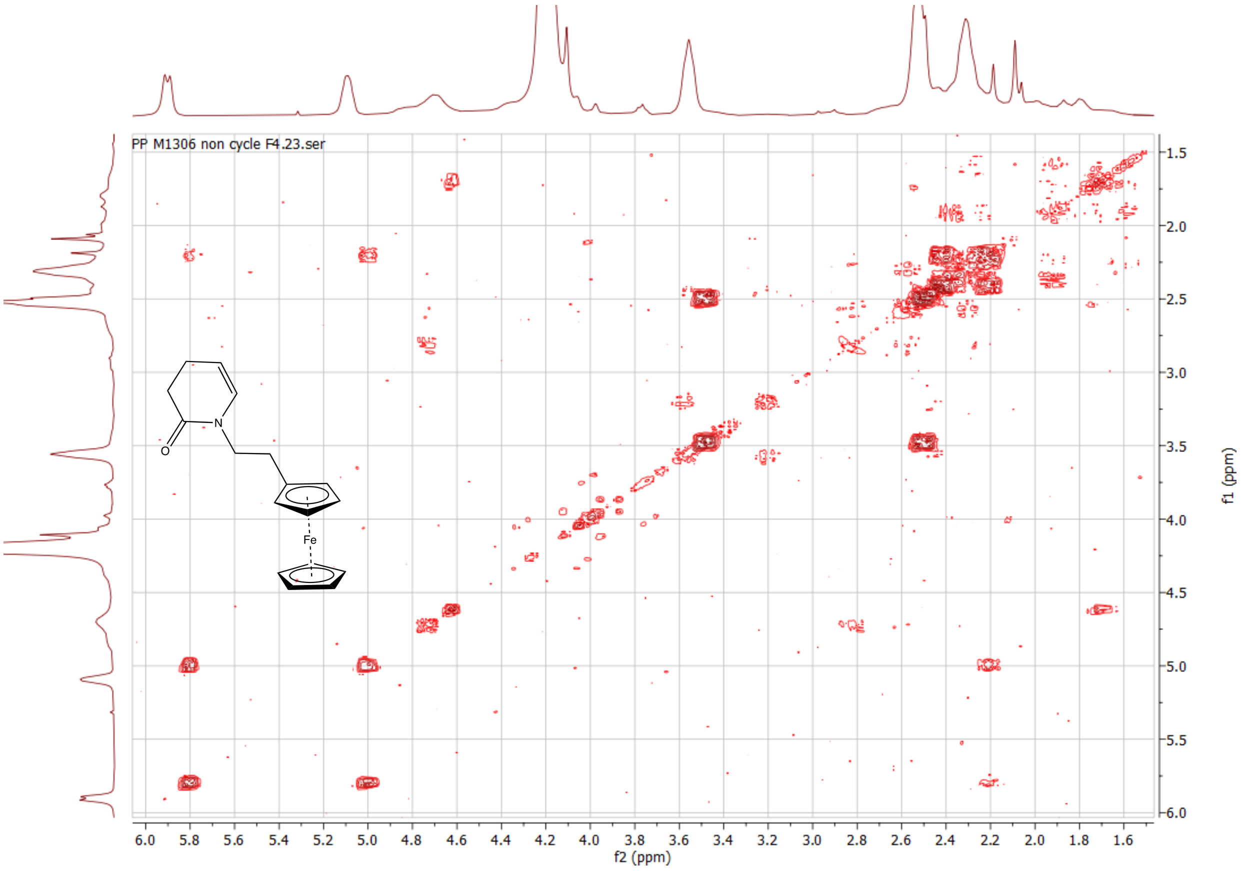
PP M1306 non cycle F4.23.ser
O
N
Fe
f2 (ppm)
f1 (ppm)

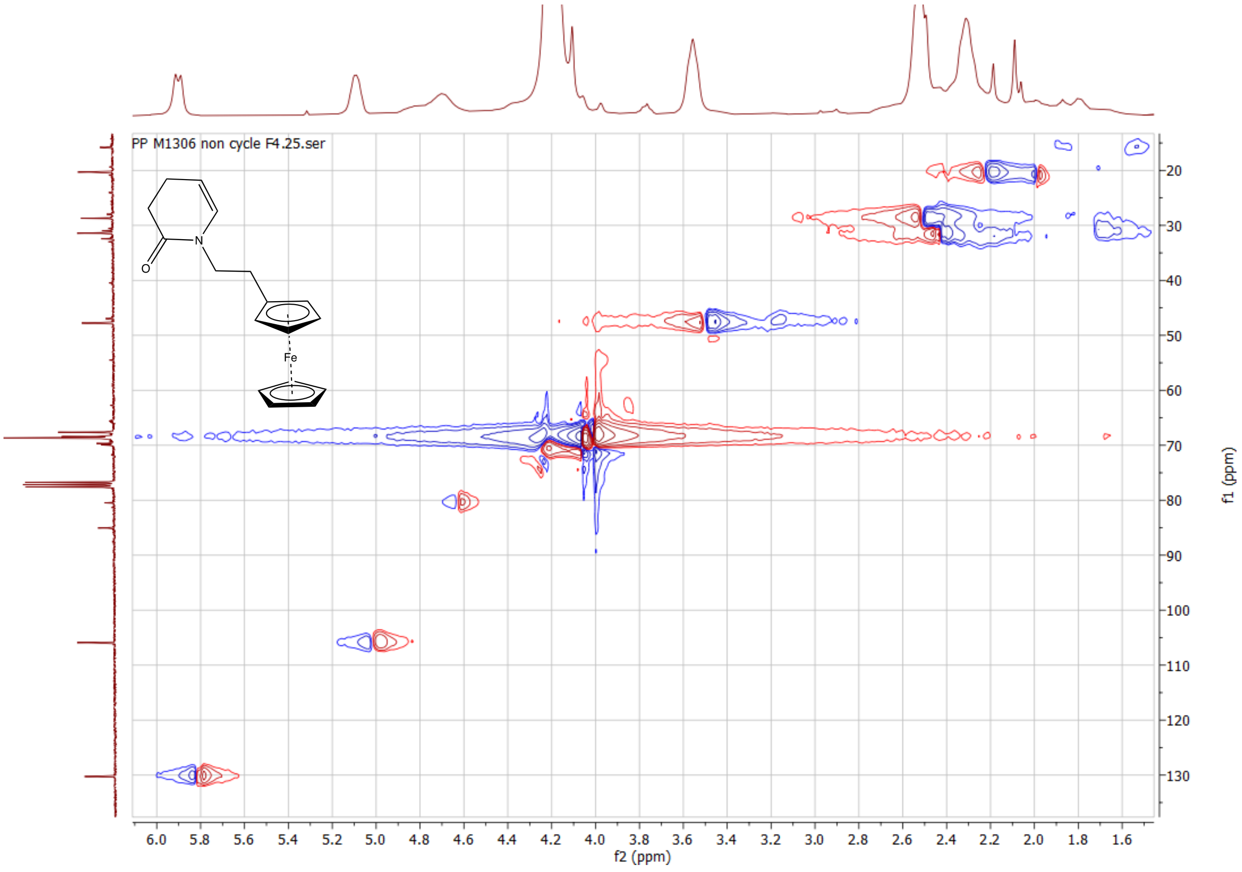
PP M1306 non cycle F4.25.ser
O
N
Fe
f2 (ppm)
f1 (ppm)

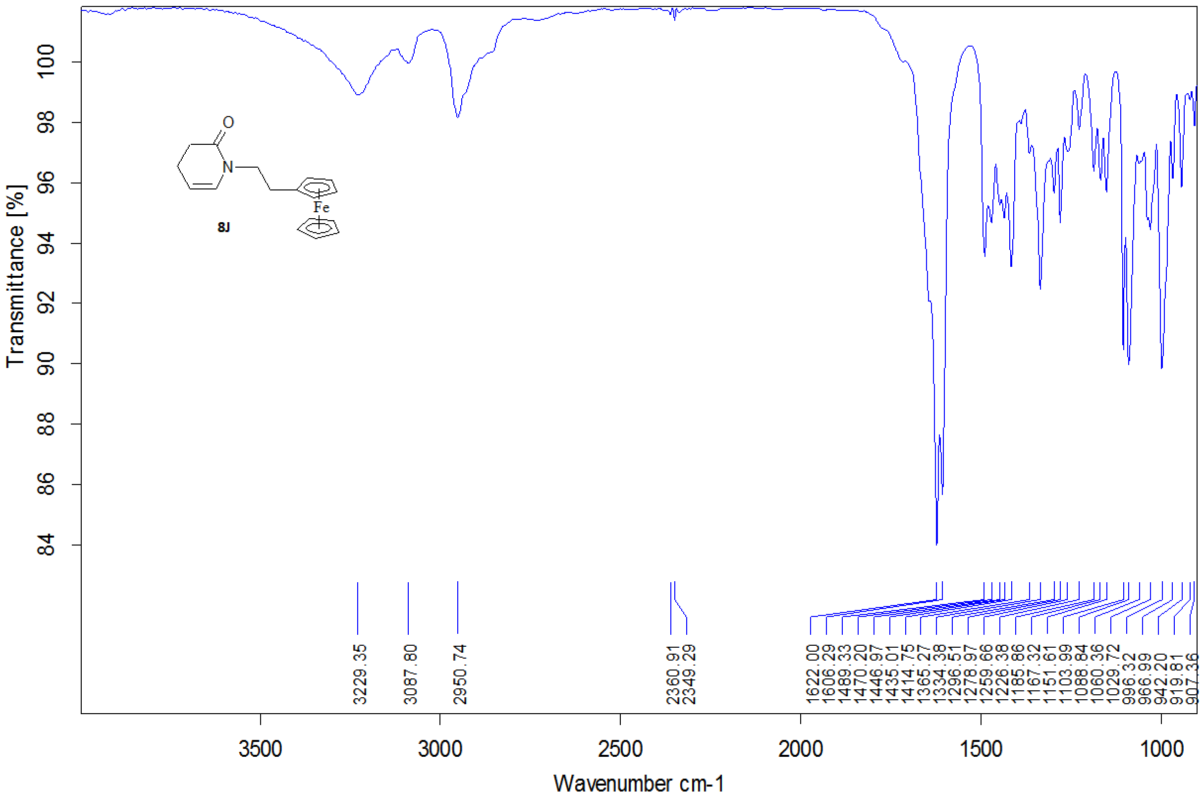

Transmittance [%]
Wavenumber cm-1
8J
Fe
O
N
3229.35
3087.80
2950.74
2360.91
2349.29
1622.00
1606.29
1489.33
1470.20
1446.97
1435.01
1414.75
1365.27
1334.38
1296.51
1278.97
1259.66
1226.38
1185.86
1167.32
1151.61
1103.99
1088.84
1060.36
1029.72
996.32
966.99
942.20
919.81
907.36

Figure S30: ^{1}H (in $CDCl_3$), ^{13}C (in $CDCl_3$) NMR and IR data for compound **9j**

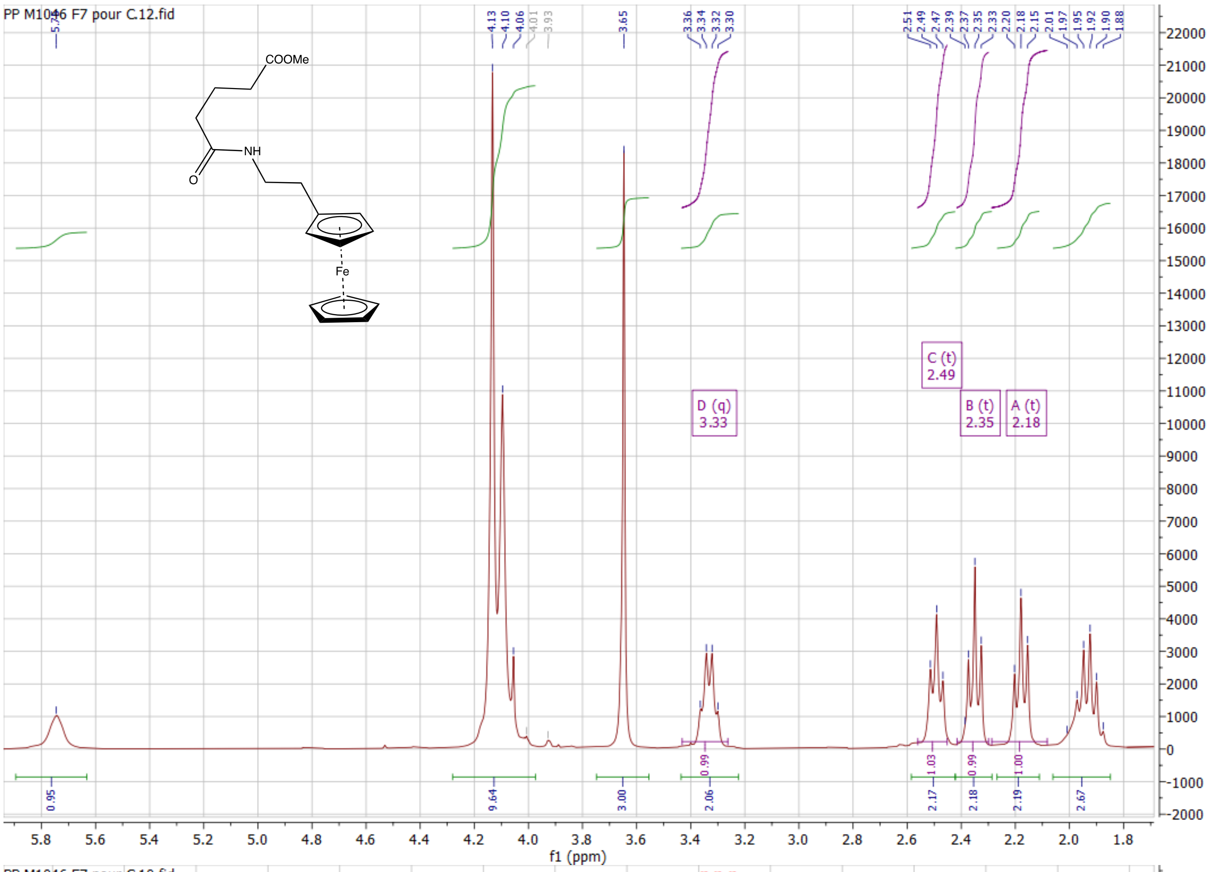

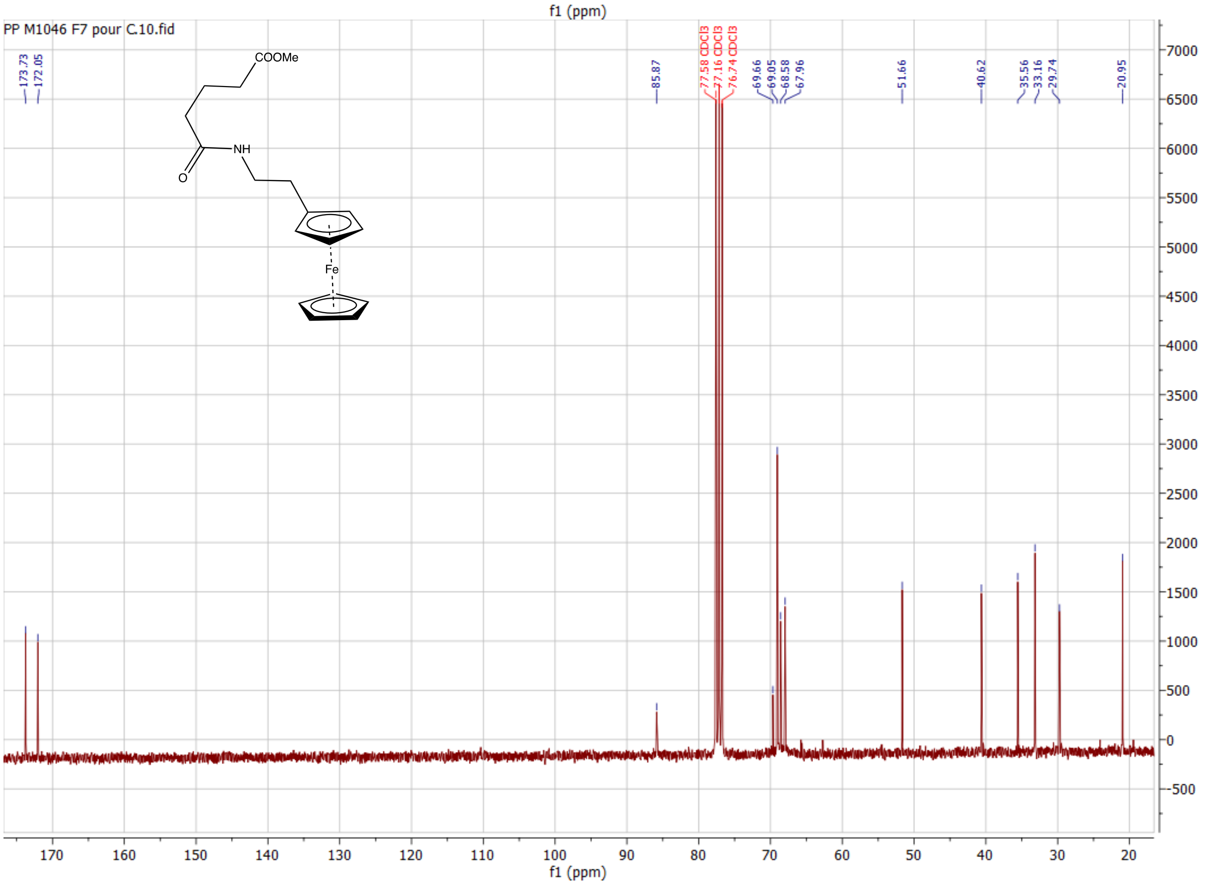

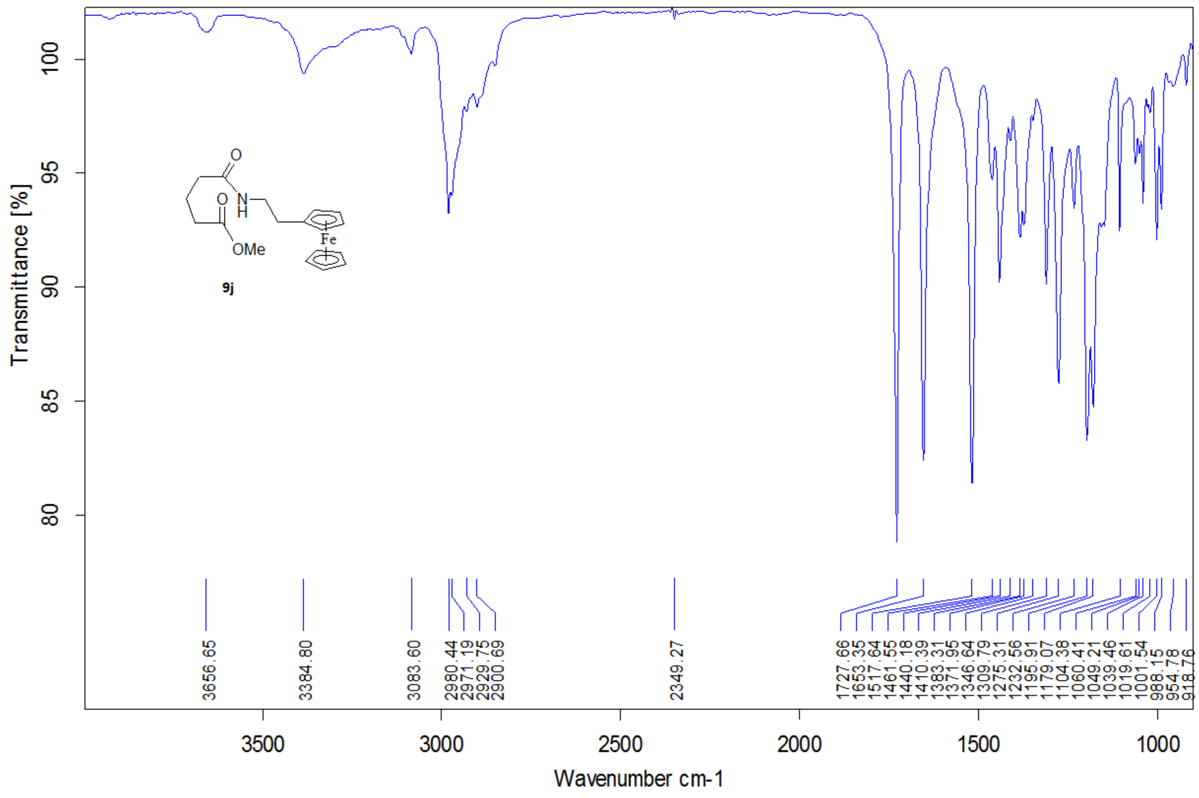
Transmittance [%]
Wavenumber cm-1
OMe
Fe
9j
3656.65
3384.80
3083.60
2980.44
2971.19
2929.75
2900.69
2349.27
1727.66
1653.35
1517.64
1461.55
1440.18
1410.39
1383.31
1371.95
1346.64
1309.79
1275.31
1232.56
1195.91
1179.07
1104.38
1060.41
1049.21
1039.46
1019.61
1001.54
988.15
954.78
918.76

Figure S31: ^{1}H (in $CDCl_3$),^{13}C (in $CDCl_3$), COSY (in $CDCl_3$),HMQC (in $CDCl_3$) NMR, HR-MS and IR data for compound **10b**

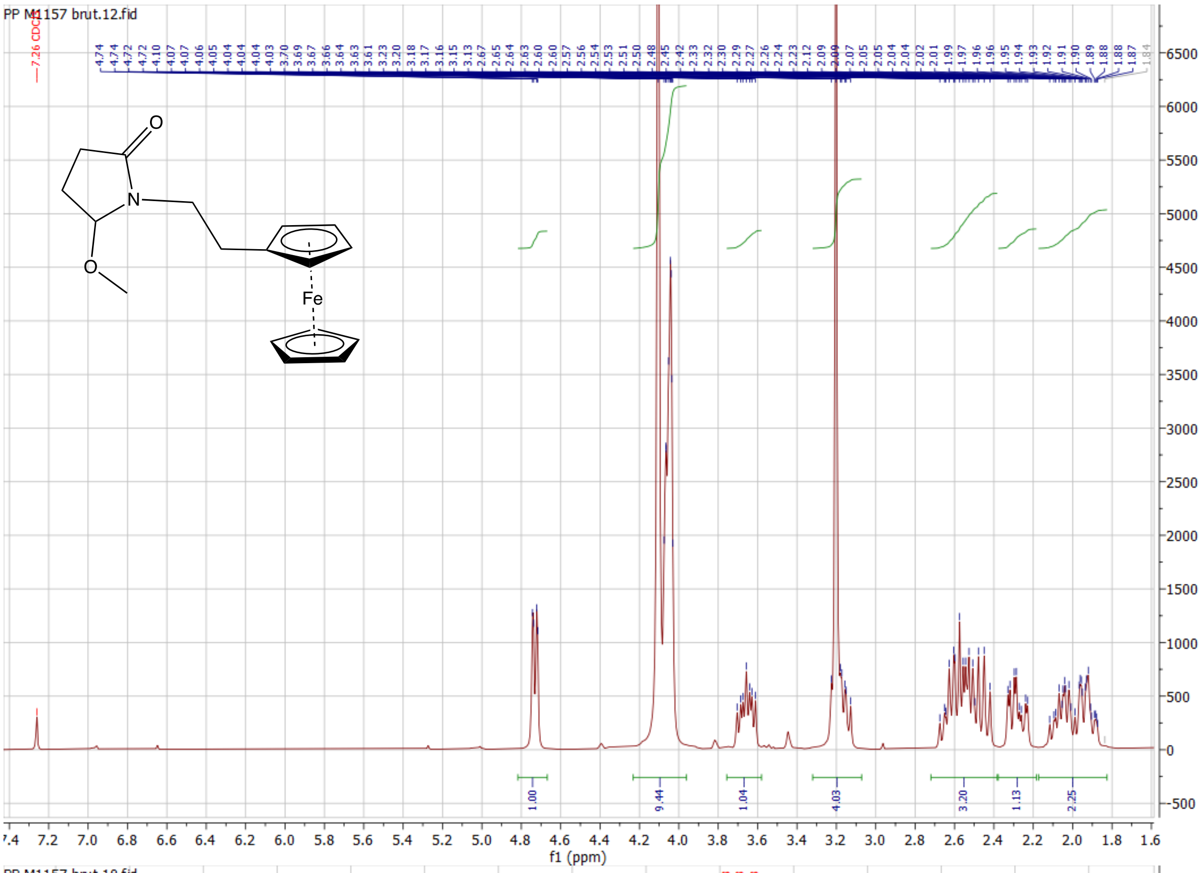

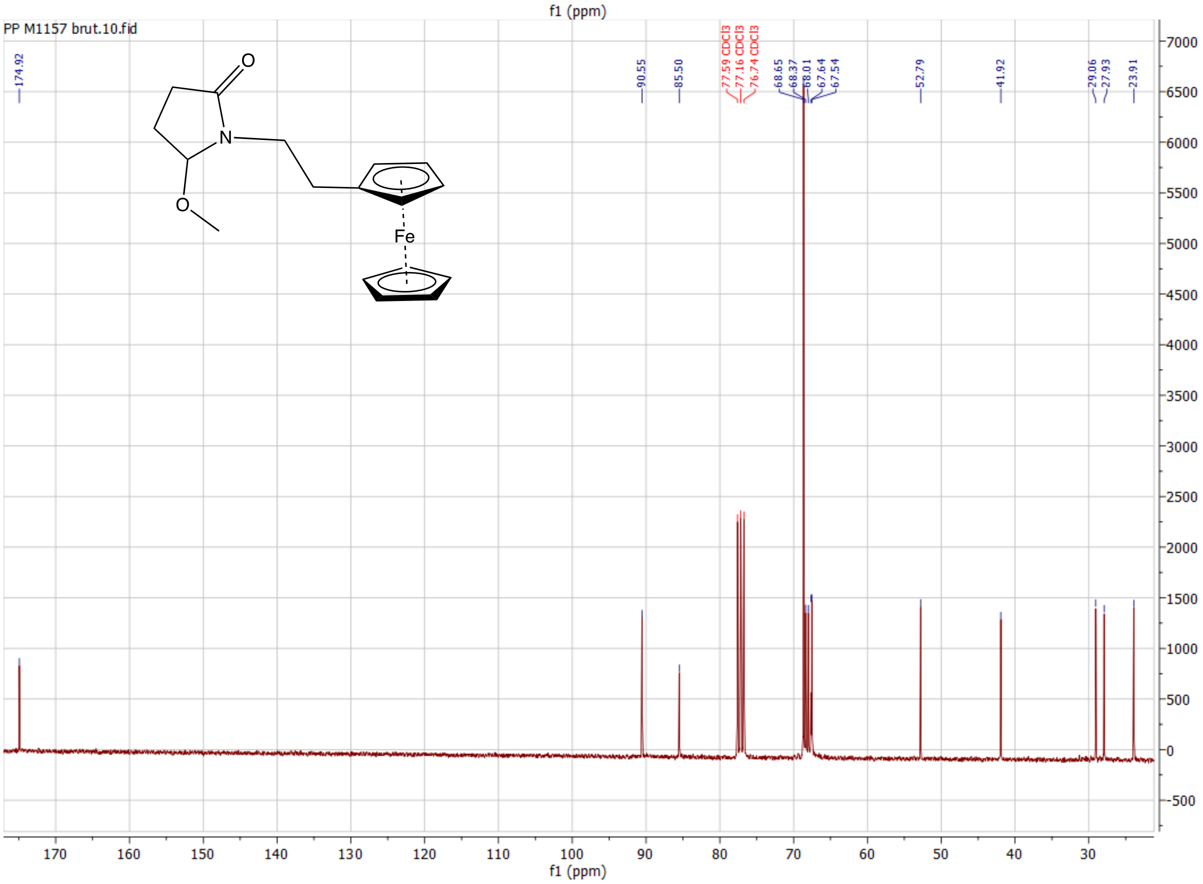

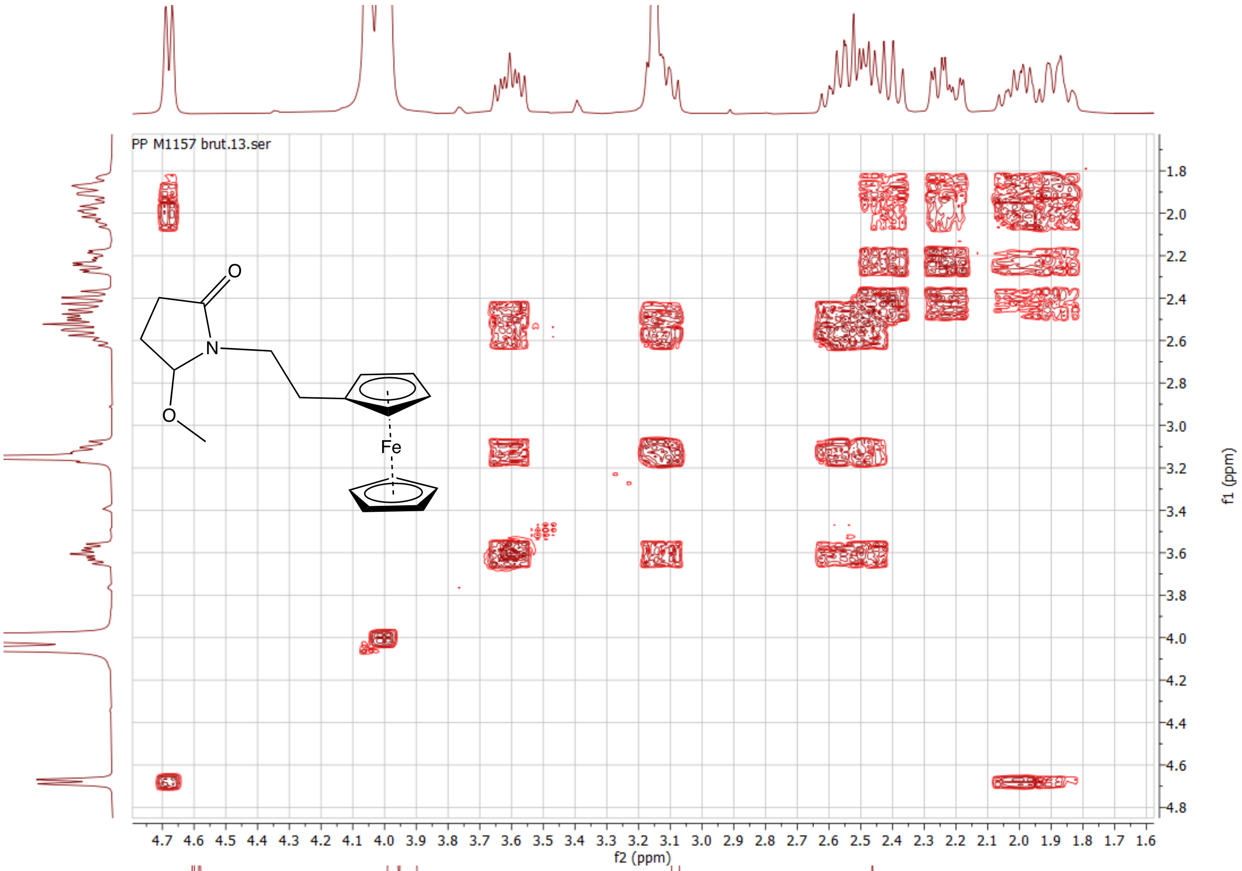
PP M1157 brut.13.ser
f2 (ppm)
f1 (ppm)
O
N
O
Fe

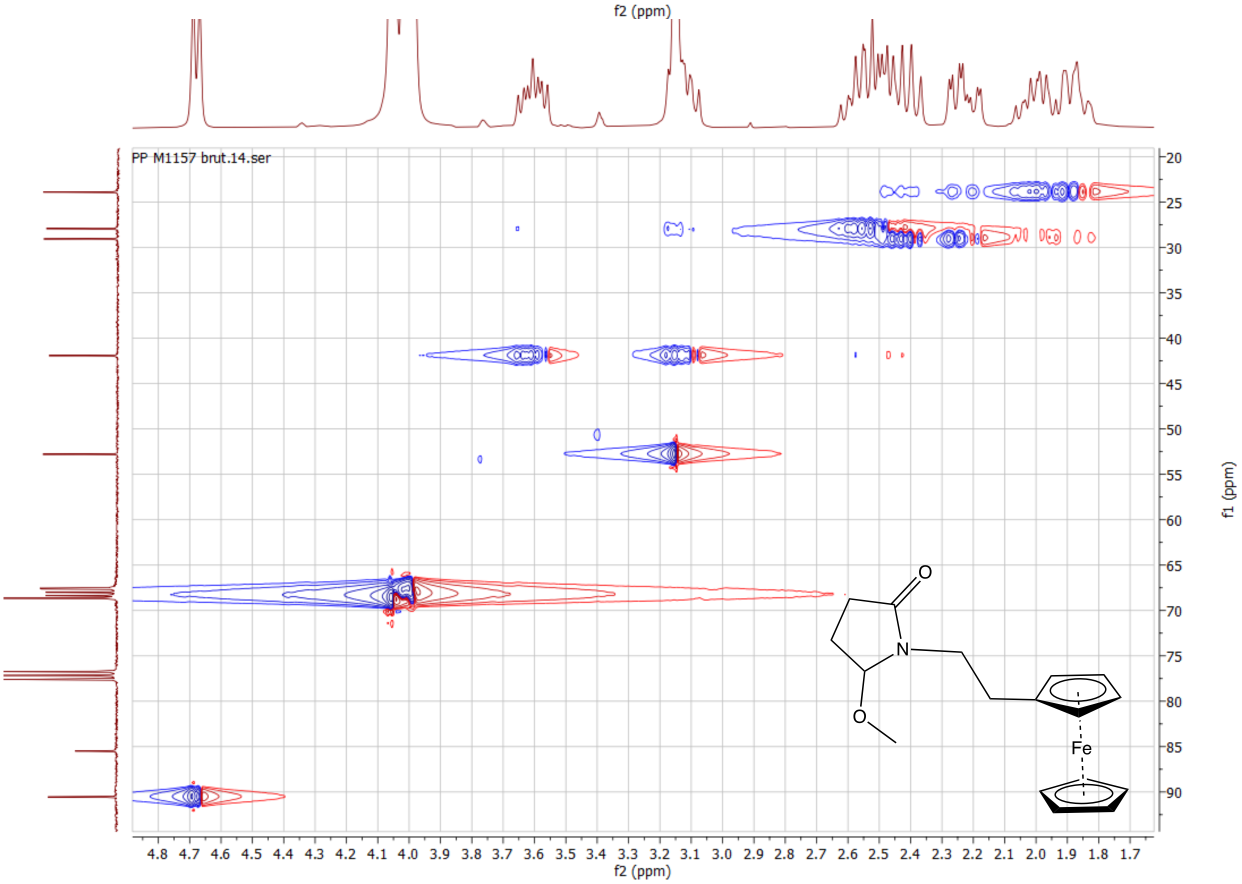
PP M1157 brut.14.ser
f2 (ppm)
f1 (ppm)
O
N
O
Fe

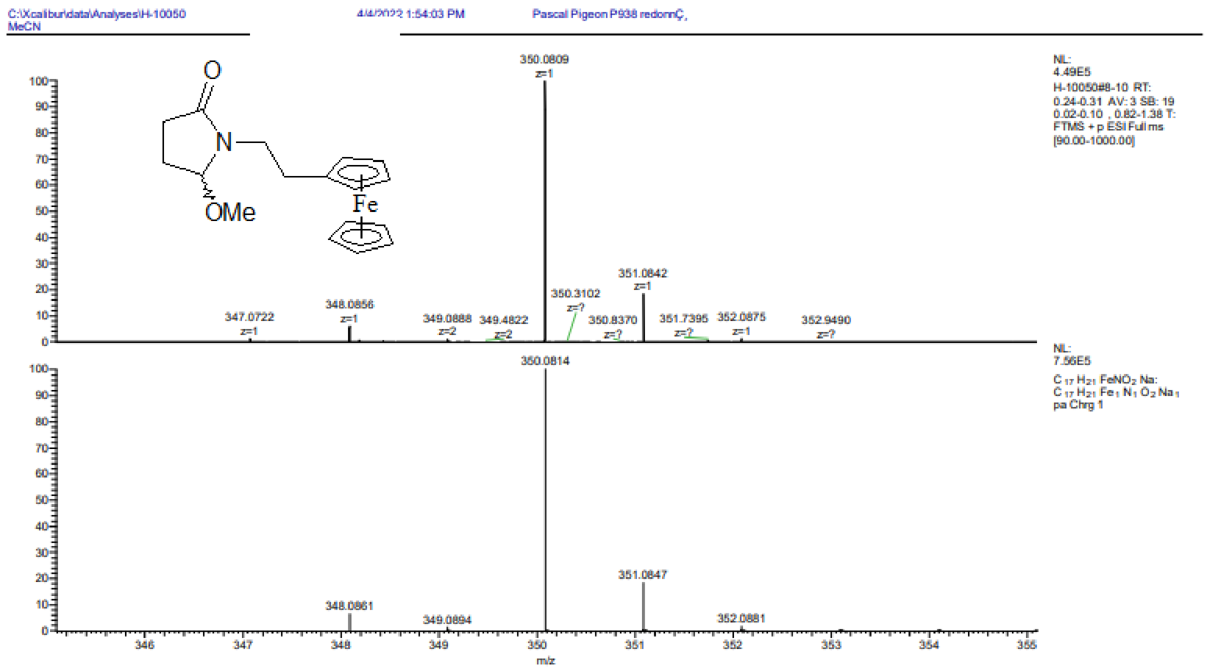

Experimental/theoretical isotopic pattern MS spectrum

Error = -1.4 ppm; Relative Intensity (%) 100

HRMS (ESI) m/z: [M+Na]+ Calcd for C17H21FeNO2Na 350.0814. Found 350.0809; (Error: -1.4 ppm).

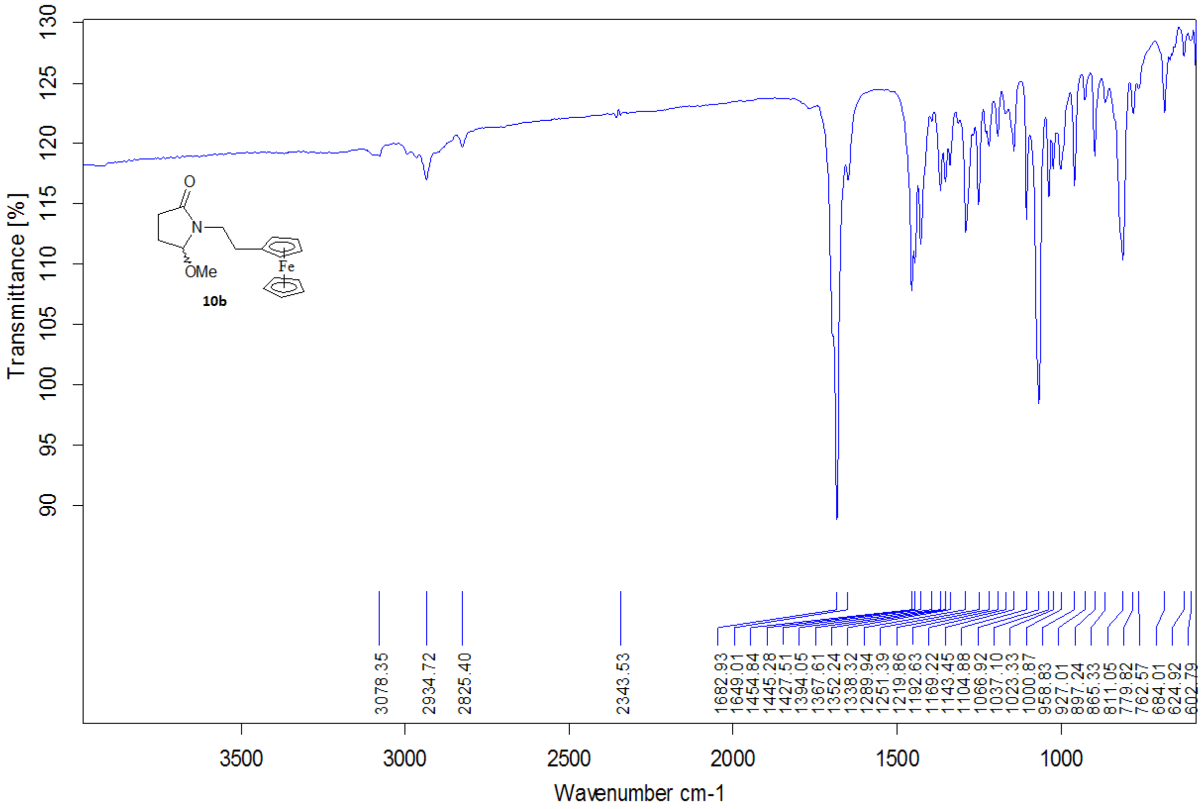

Figure S32: ^{1}H (in $CDCl_3$), ^{13}C (in $CDCl_3$) NMR, HR-MS and IR data for compound **10e**

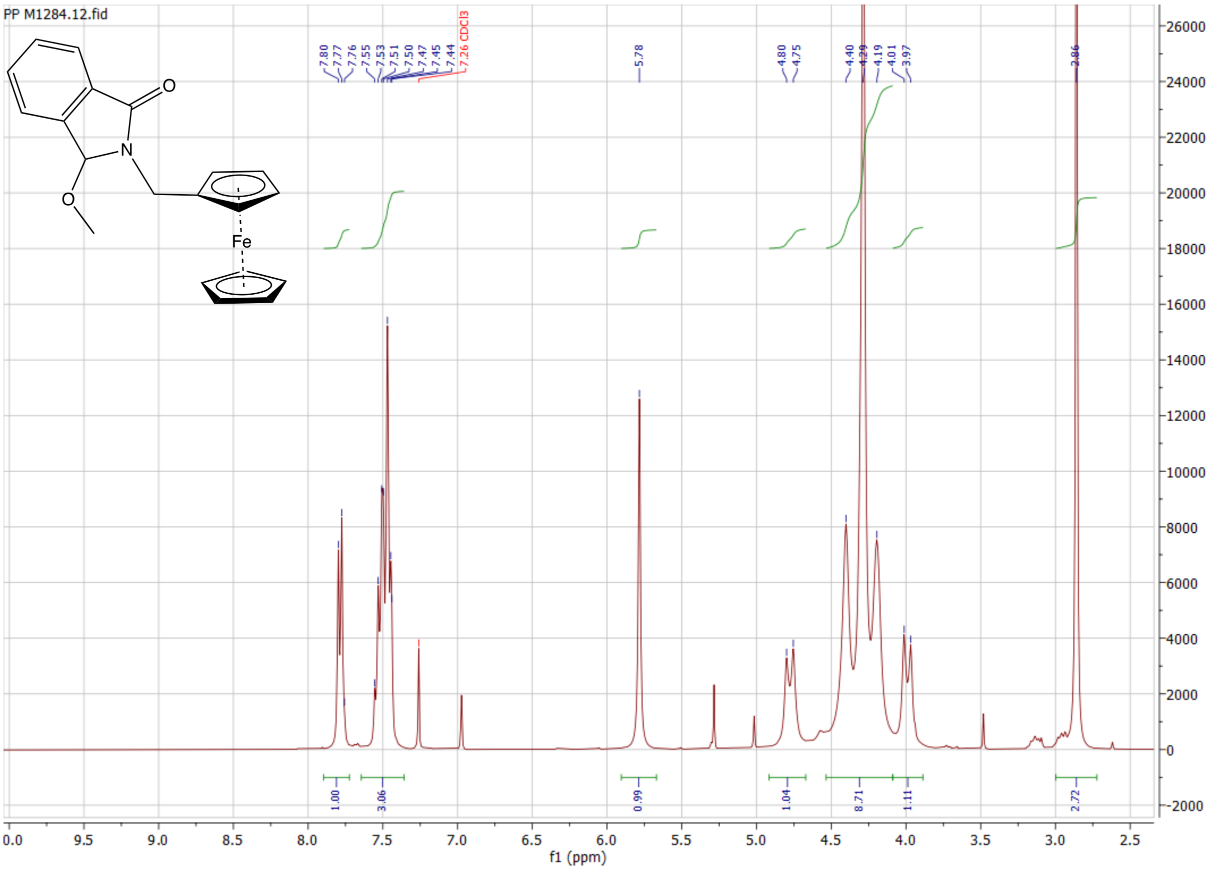

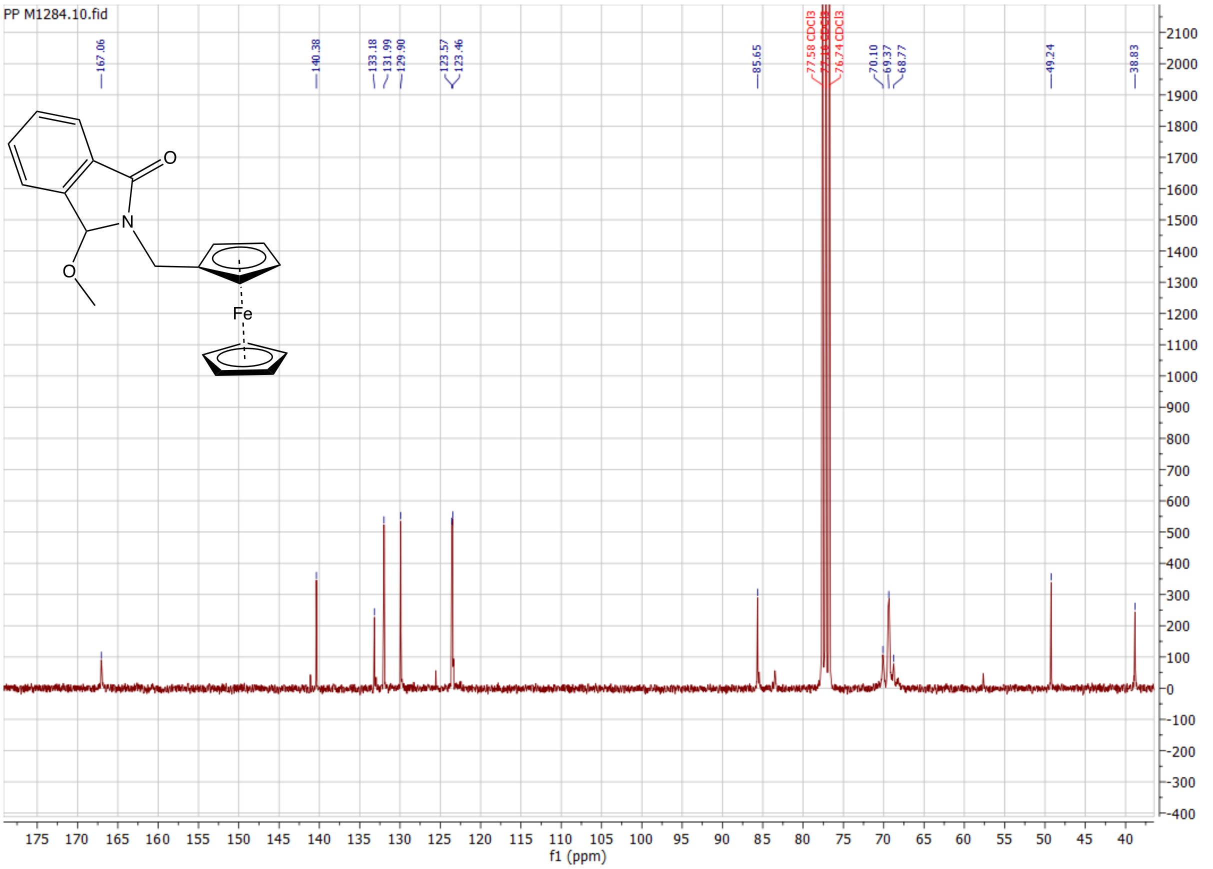

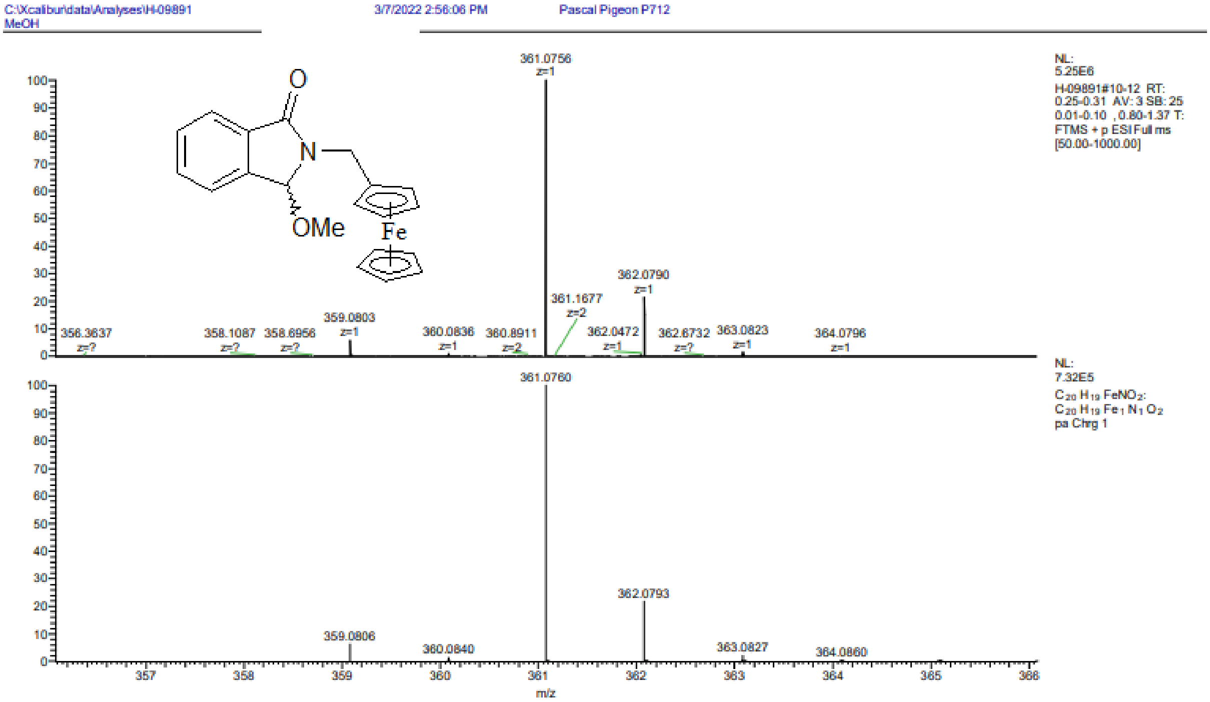

Experimental/theoretical isotopic pattern MS spectrum

Error = -1.0 ppm; Relative Intensity (%) 100

HRMS (ESI) m/z: [M]+ Calcd for C20H19FeNO2 361.0760 . Found 361.0756; (Error: -1.0 ppm).

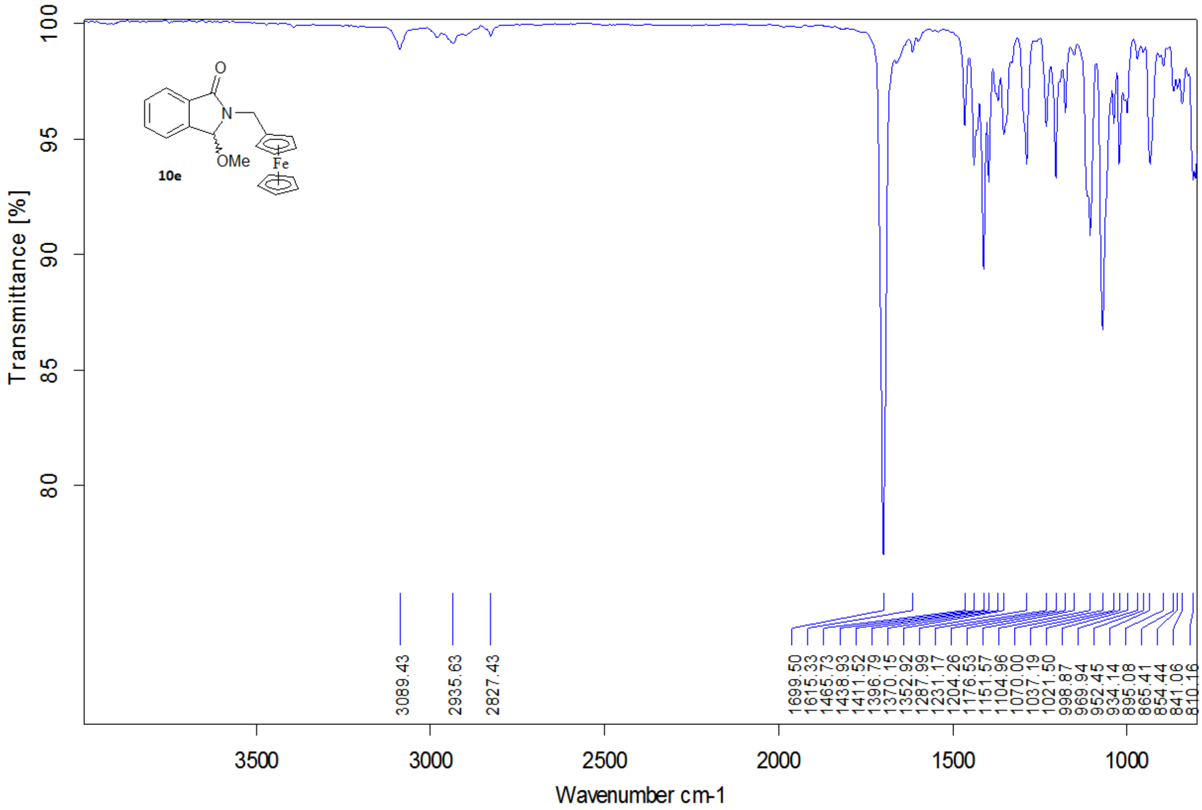

Figure S33: ^{1}H (in $CDCl_3$), ^{13}C (in $CDCl_3$) NMR, HR-MS and IR data for compound **10f**

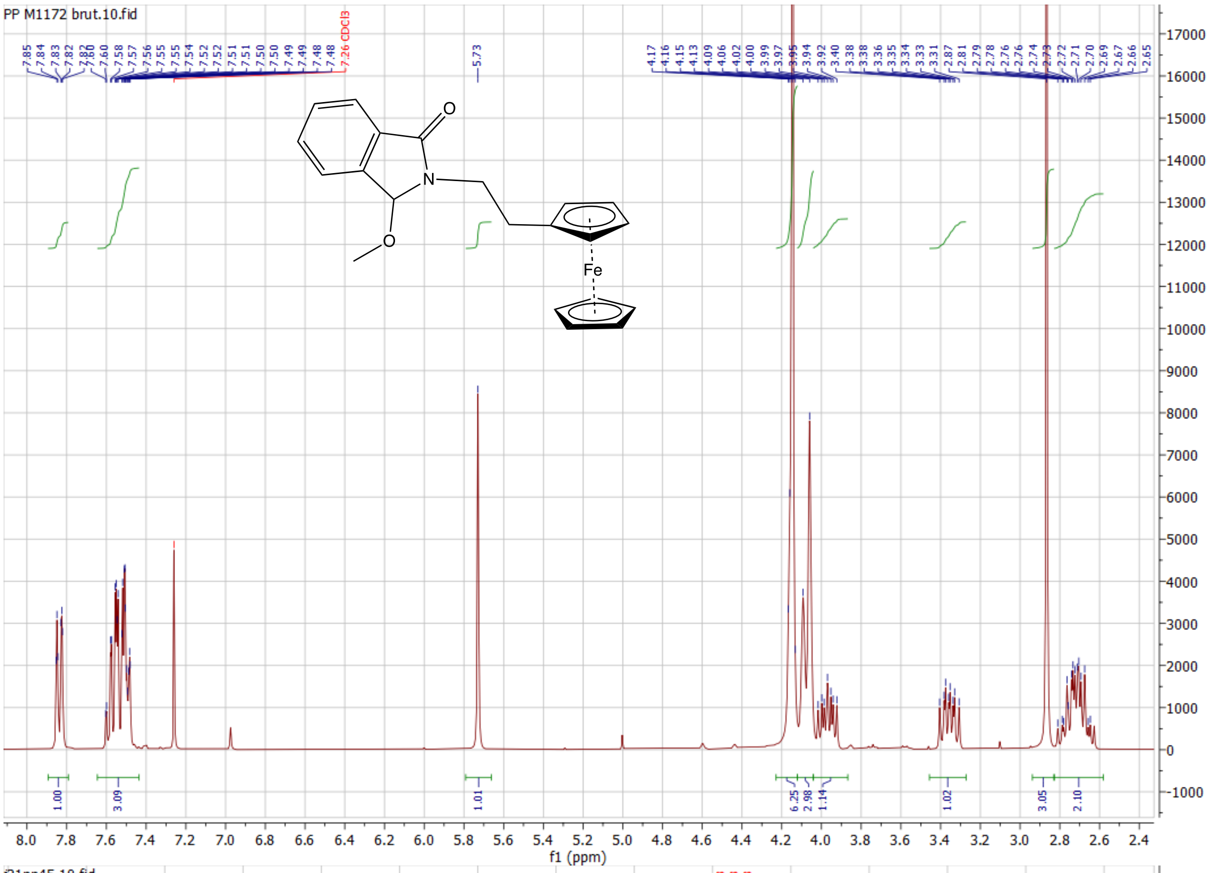

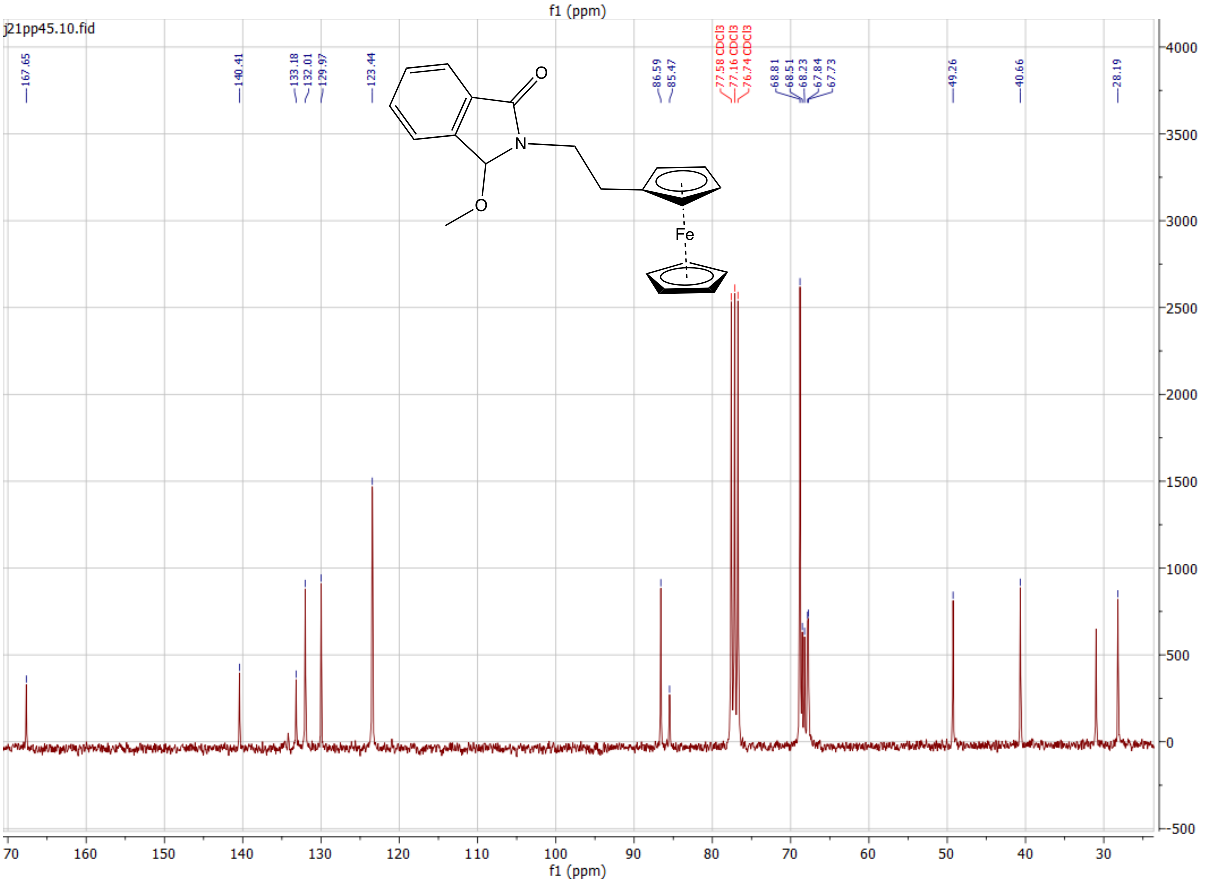

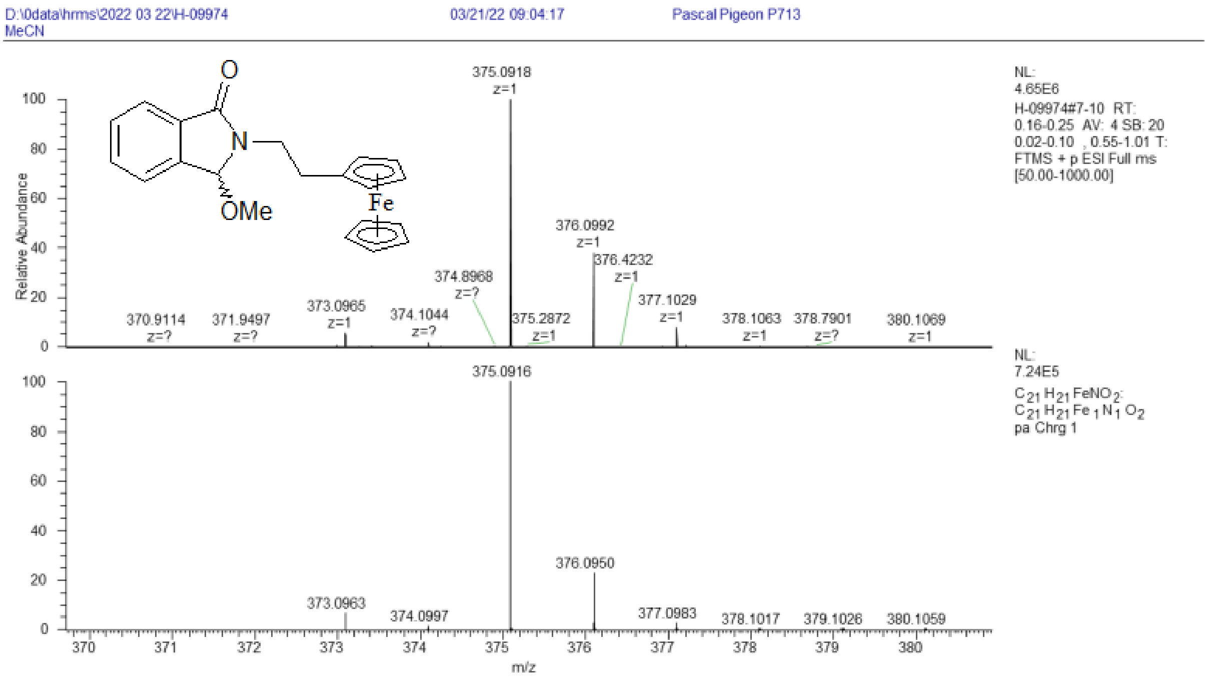

Experimental/theoretical isotopic pattern MS spectrum

Error = 0.2 ppm; Relative Intensity (%) 100

HRMS (ESI) m/z: [M+H]+ Calcd for C21H21FeNO2H 376.0994. Found 376.0995; (Error: 0.2 ppm).

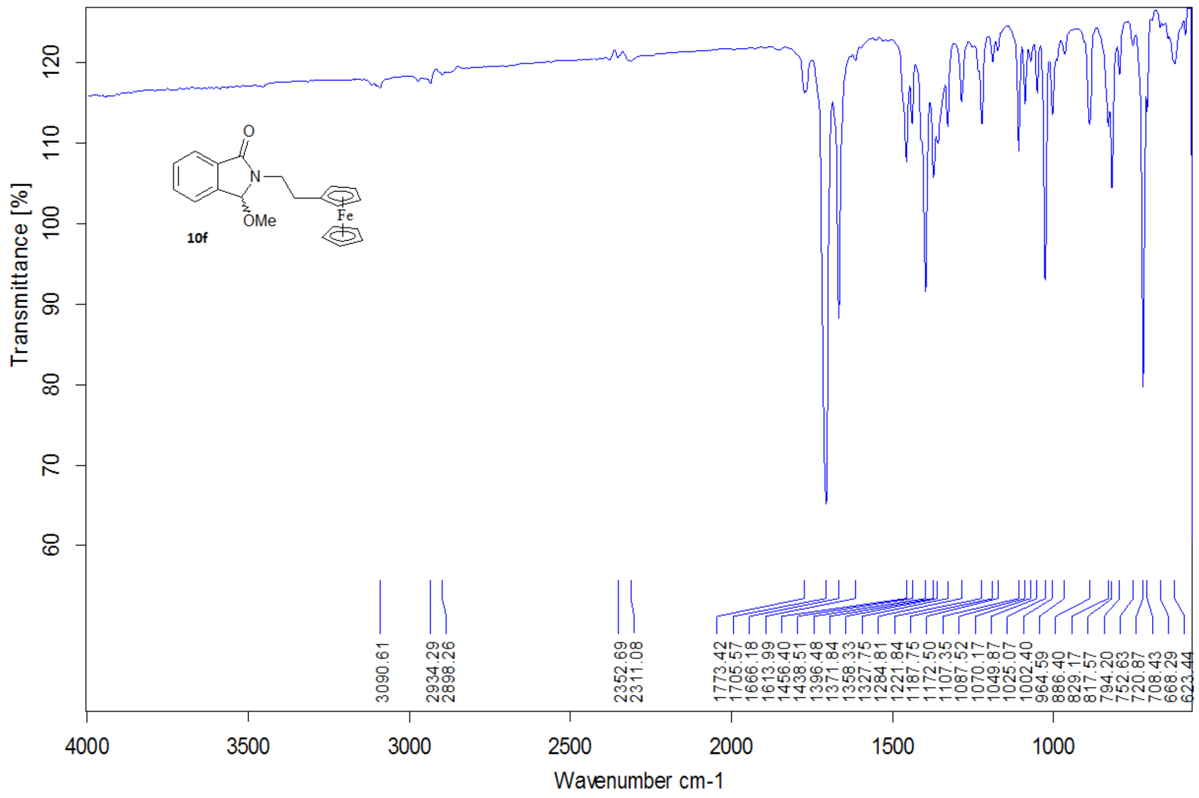

Figure S34: ^{1}H (in $CDCl_3$), ^{13}C (in $CDCl_3$) NMR, HR-MS and IR data for compound **10g**

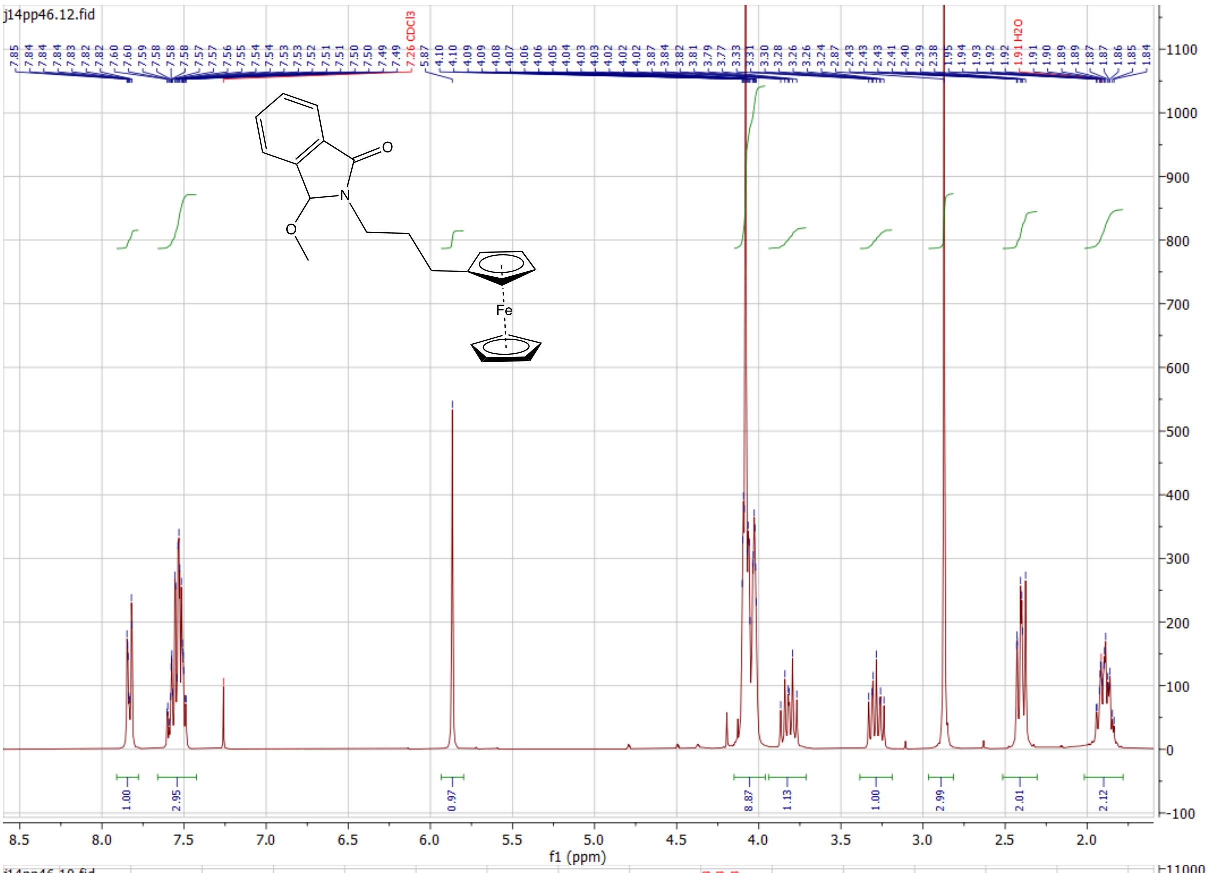

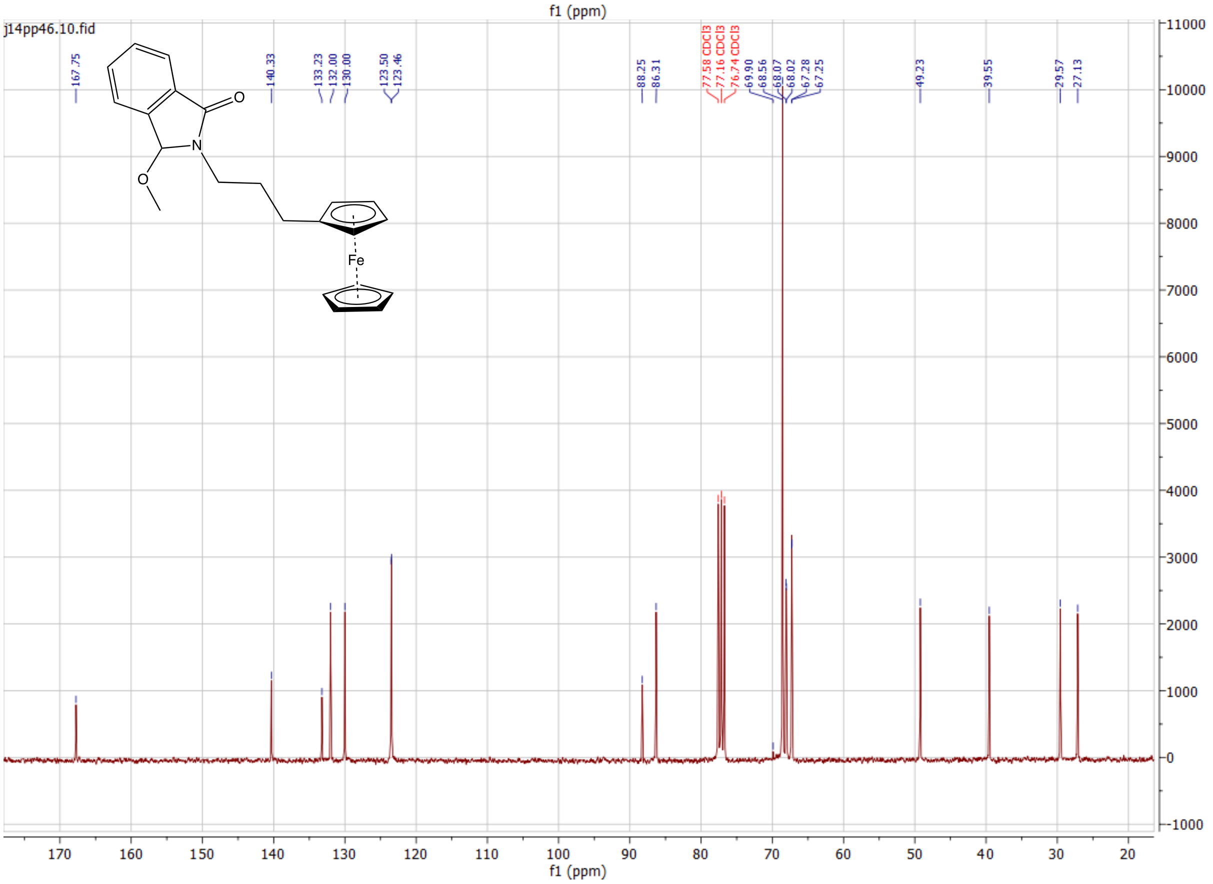

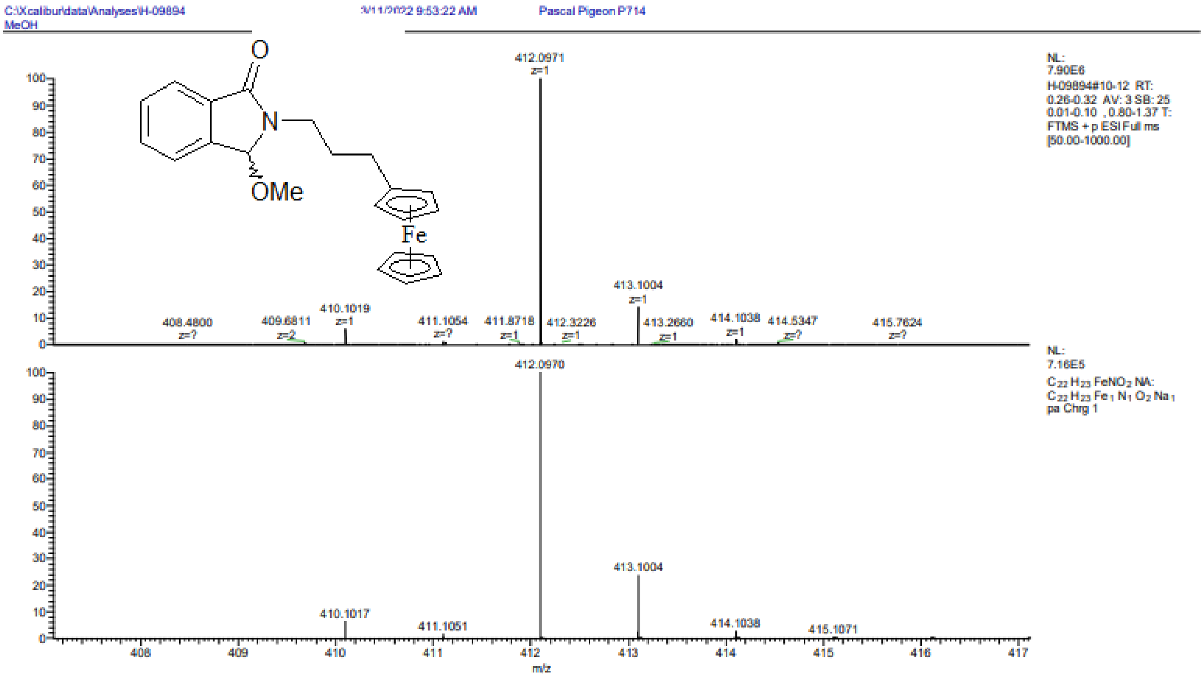

Experimental/theoretical isotopic pattern MS spectrum

Error = 0.2 ppm; Relative Intensity (%) 100

HRMS (ESI) m/z: [M]+ Calcd for C22H23FeNO2Na 412.0970 . Found 412.0971; (Error: 0.2 ppm).

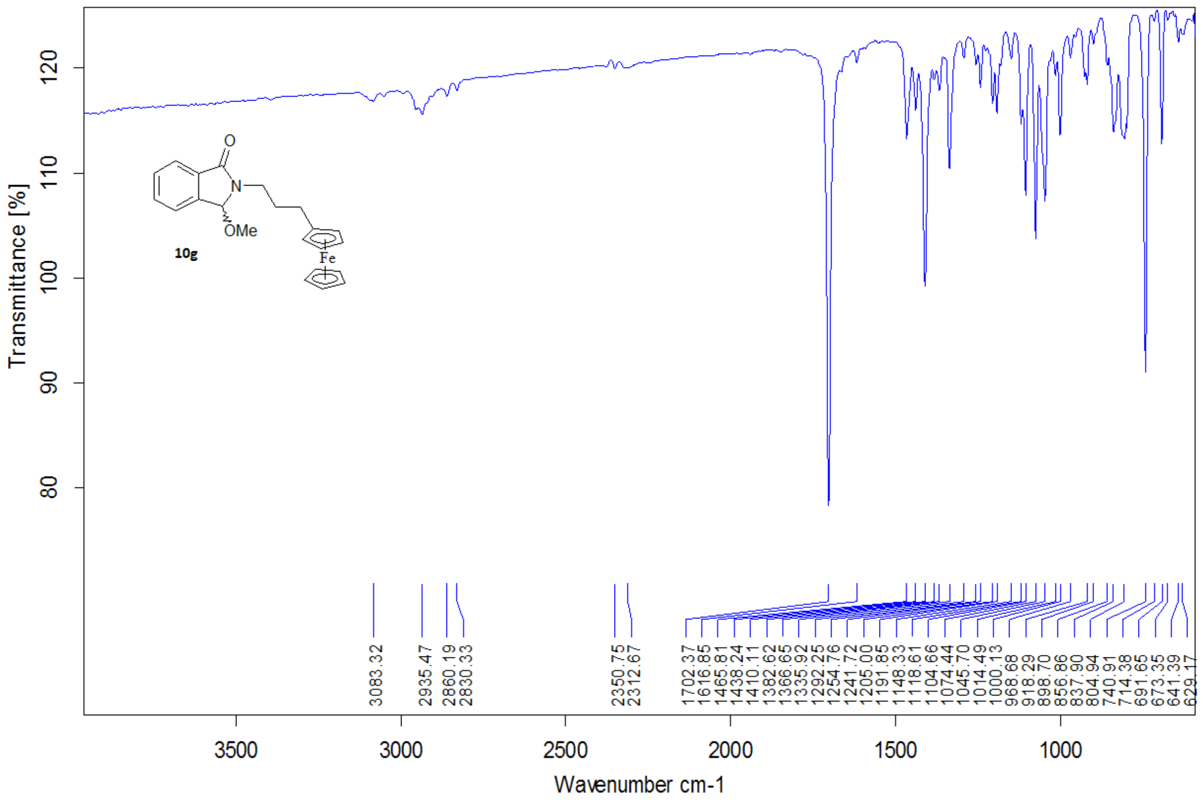

Figure S35: ^{1}H (in $CDCl_3$), ^{13}C (in $CDCl_3$) NMR, HR-MS and IR data for compound **10h**

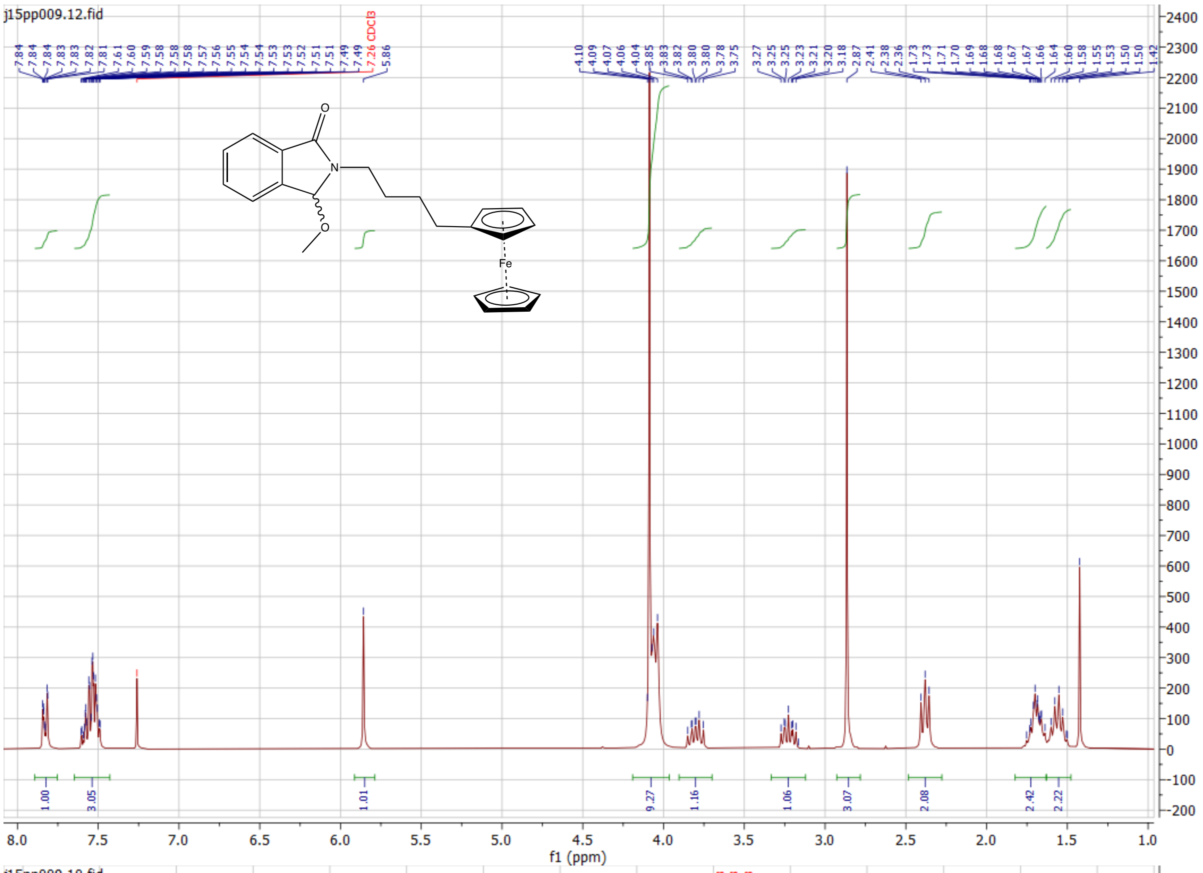

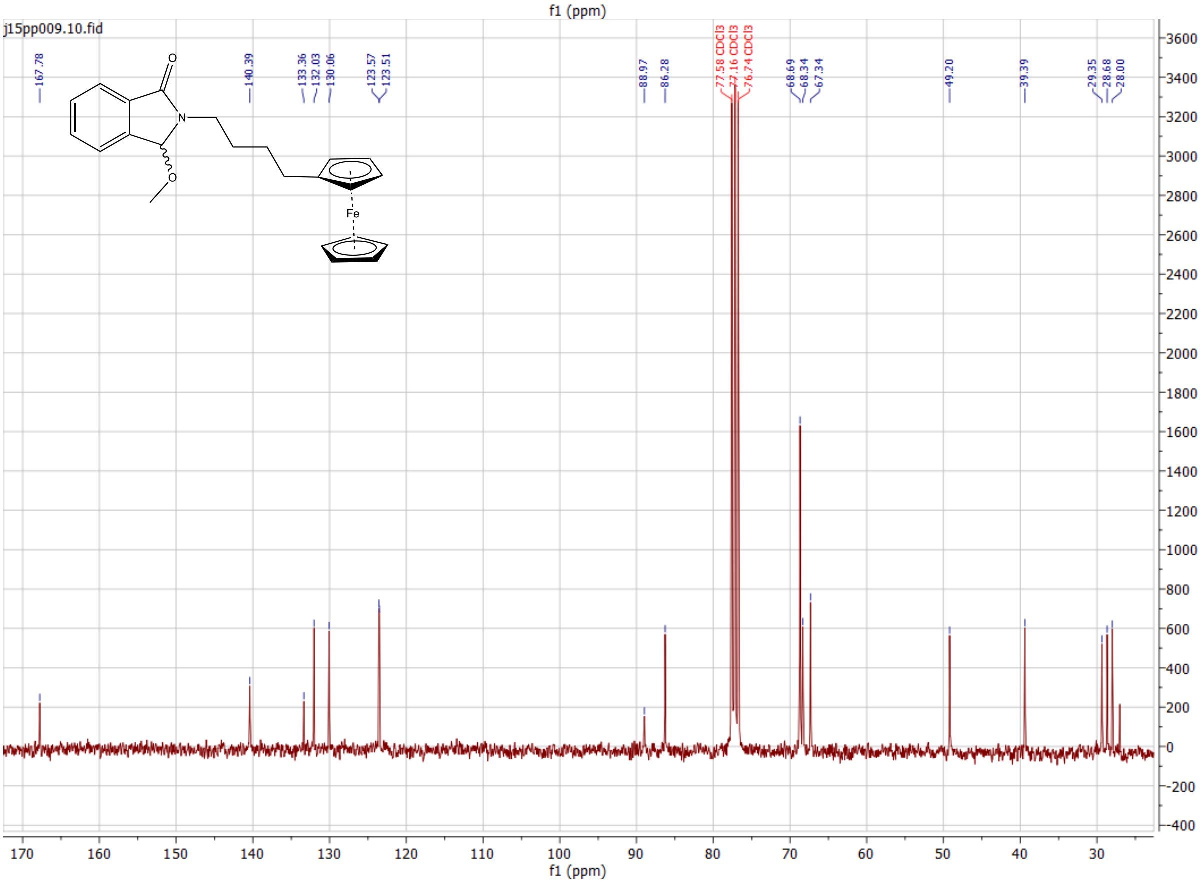

Single Mass Analysis
Tolerance = 5.0 PPM / DBE: min = -1.5, max = 100.0
Element prediction: Off
Number of isotope peaks used for i-FIT = 9

Monoisotopic Mass, Odd Electron Ions
174 formula(e) evaluated with 1 results within limits (all results (up to 1000) for each mass)
Elements Used:
C: 1-150 H: 1-150 N: 0-3 O: 0-10 Fe: 1-1
19-Jun-2013 2::1::6
ENSCP_P715 37 (0.932) Cm (32:47)

ACN

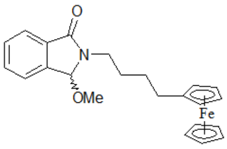

LCT Premier XE KE483
1: TOF MS ES+
1.12e+005

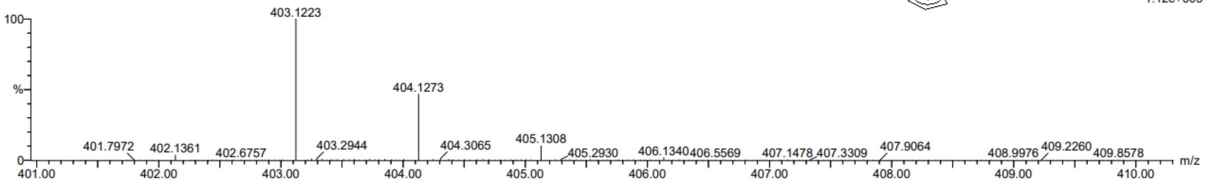

Minimum:				-1.5			
Maximum:		5.0	5.0	100.0			
Mass	Calc. Mass	mDa	PPM	DBE	i-FIT	i-FIT (Norm)	Formula
403.1223	403.1235	-1.2	-3.0	12.0	1238.0	0.0	C23 H25 N O2 Fe

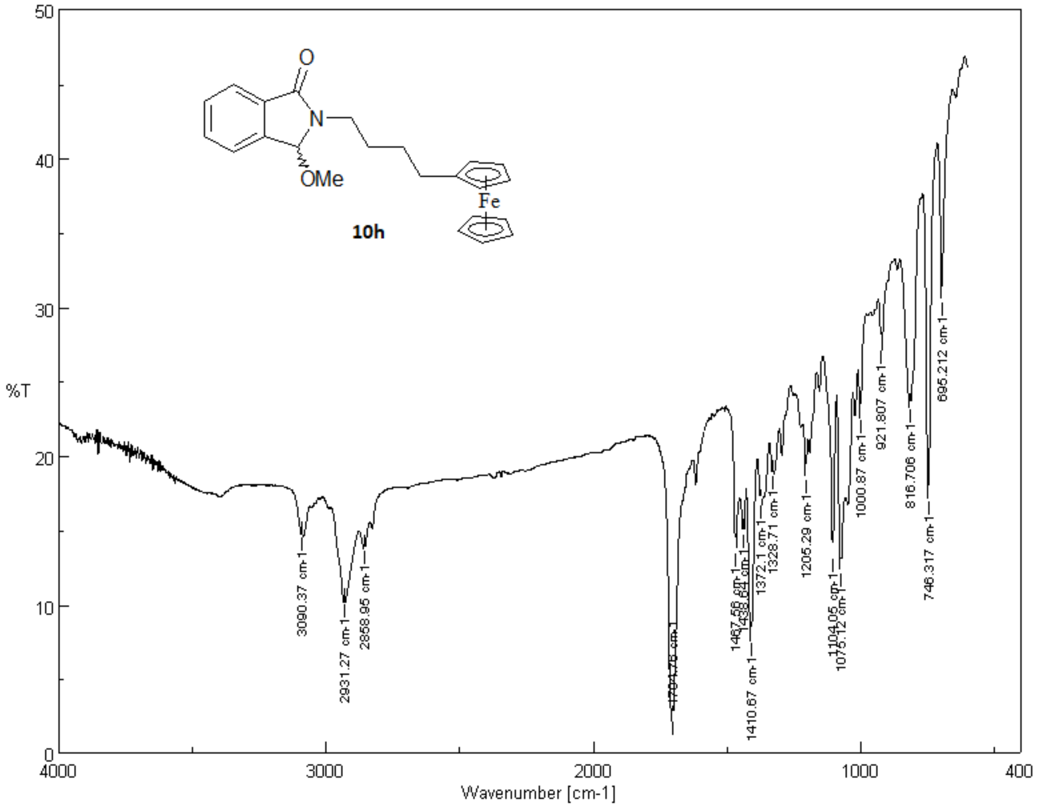

Figure S36: ^{1}H (in DMSO-d6), ^{13}C (in CDCl3), COSY (in DMSO-d6) NMR, HR-MS and IR data for compound **11**

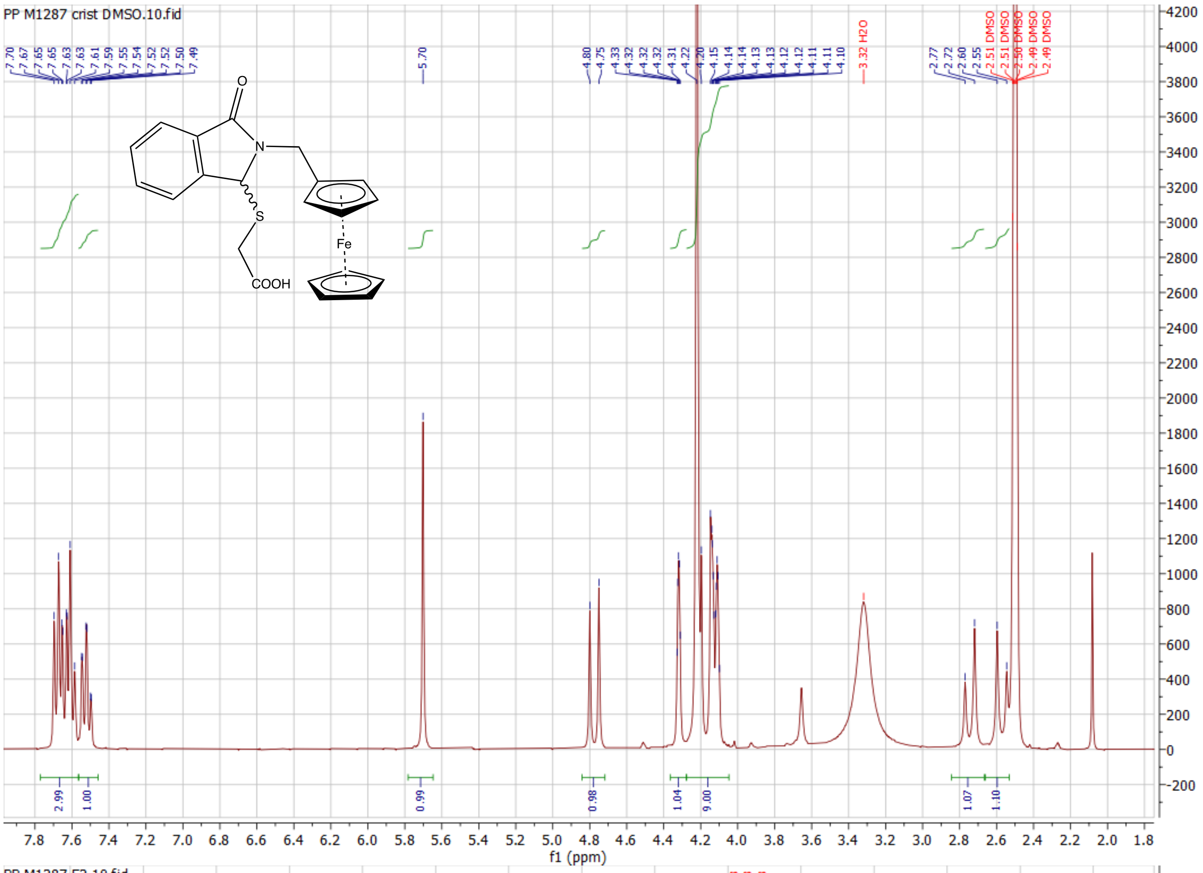

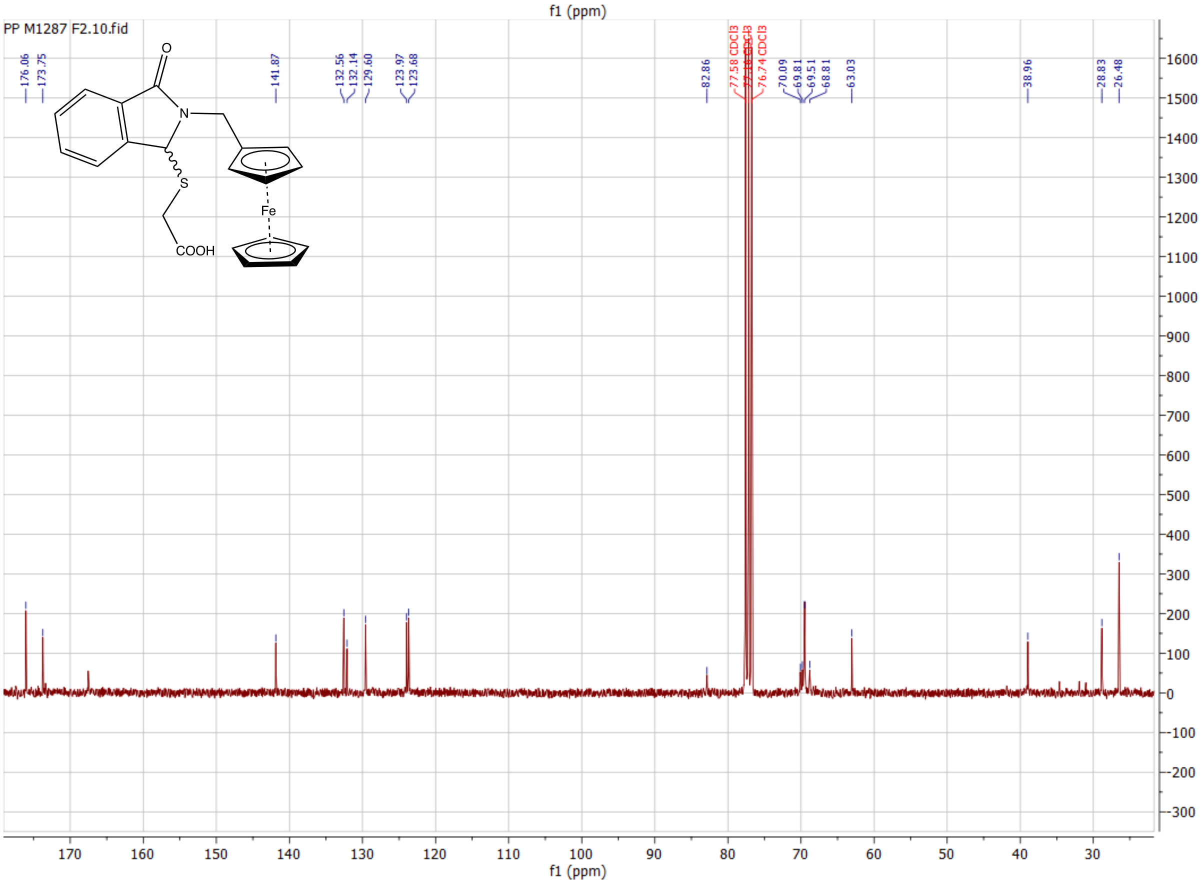

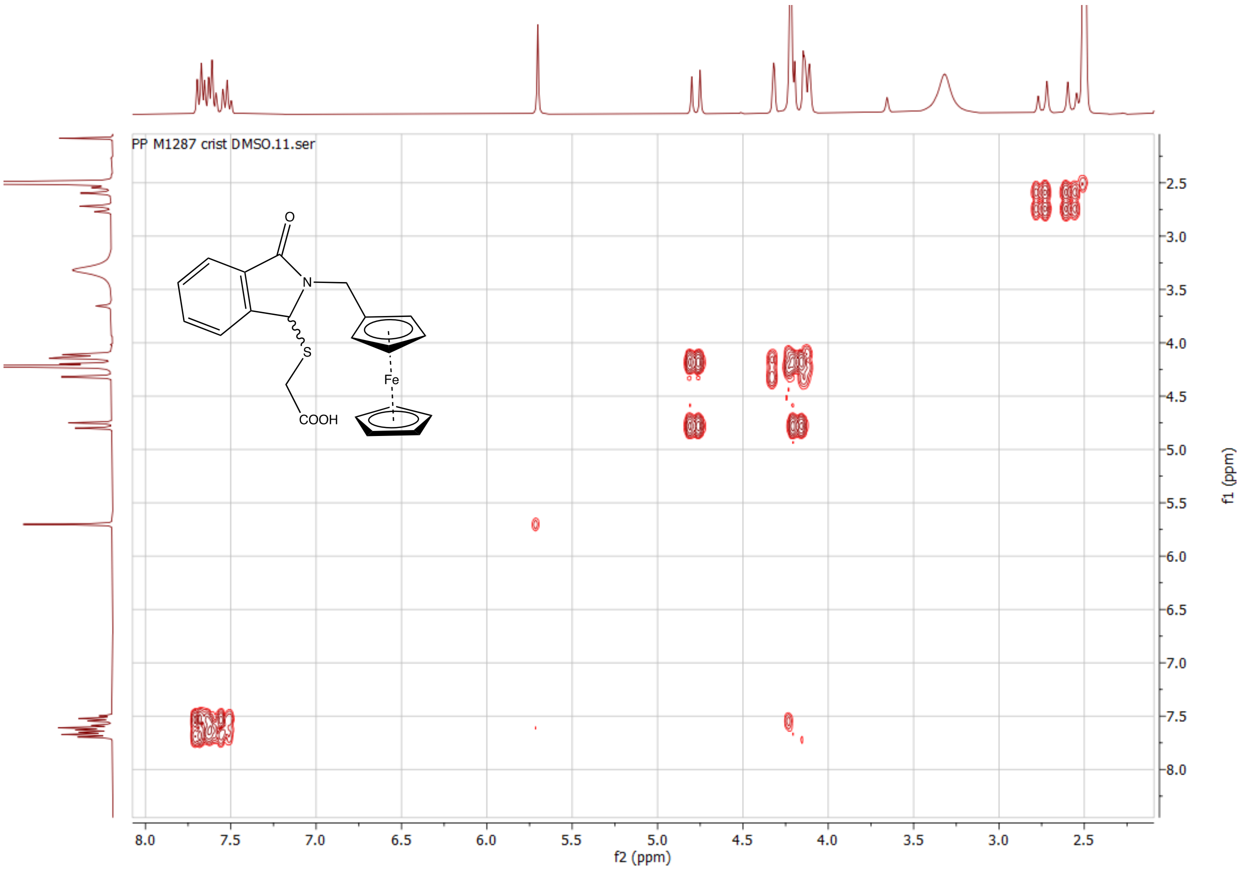

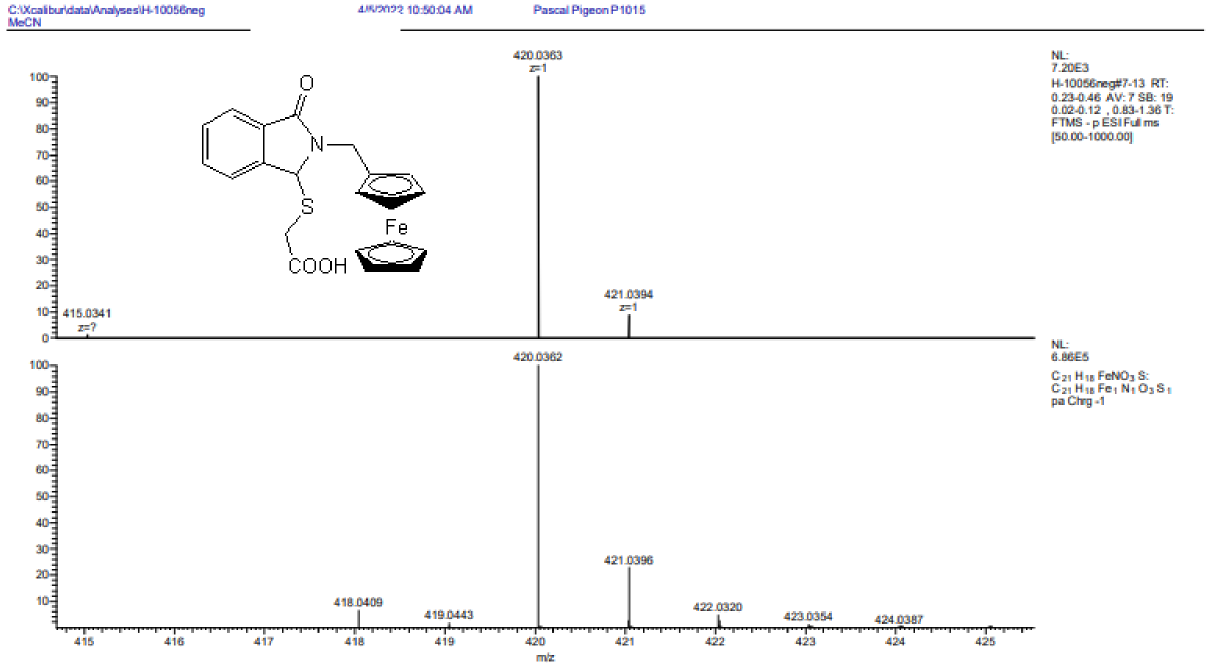

Experimental/theoretical isotopic pattern MS spectrum

Error = 0.9 ppm; Relative Intensity (%) 100

HRMS (ESI) m/z: [M] 1- Calcd for C21H18FeNO3S420.0362 . Found 420.0366; (Error: 0.9 ppm).

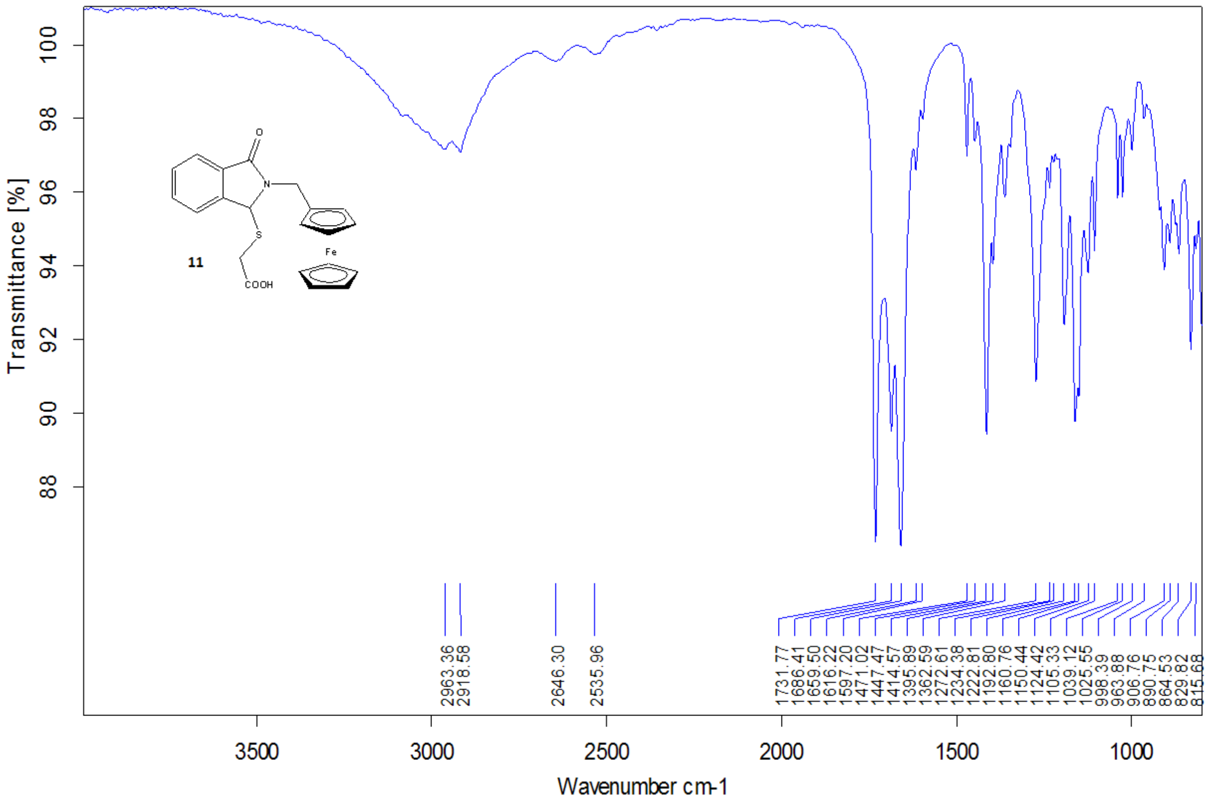
Transmittance [%]
100
98
96
94
92
90
88
O
N
S
COOH
Fe
11
2963.36
2918.58
2646.30
2535.96
1731.77
1686.41
1659.50
1616.22
1597.20
1471.02
1447.47
1414.57
1395.89
1362.59
1272.61
1234.38
1222.81
1192.80
1160.76
1150.44
1124.42
1105.33
1039.12
1025.55
998.39
963.88
906.76
890.75
864.53
829.82
815.68
3500
3000
2500
2000
1500
1000
Wavenumber cm-1

Figure S37: ^{1}H (in DMSO-d6), ^{13}C (in DMSO-d6) NMR, HR-MS and IR data for compound **13**

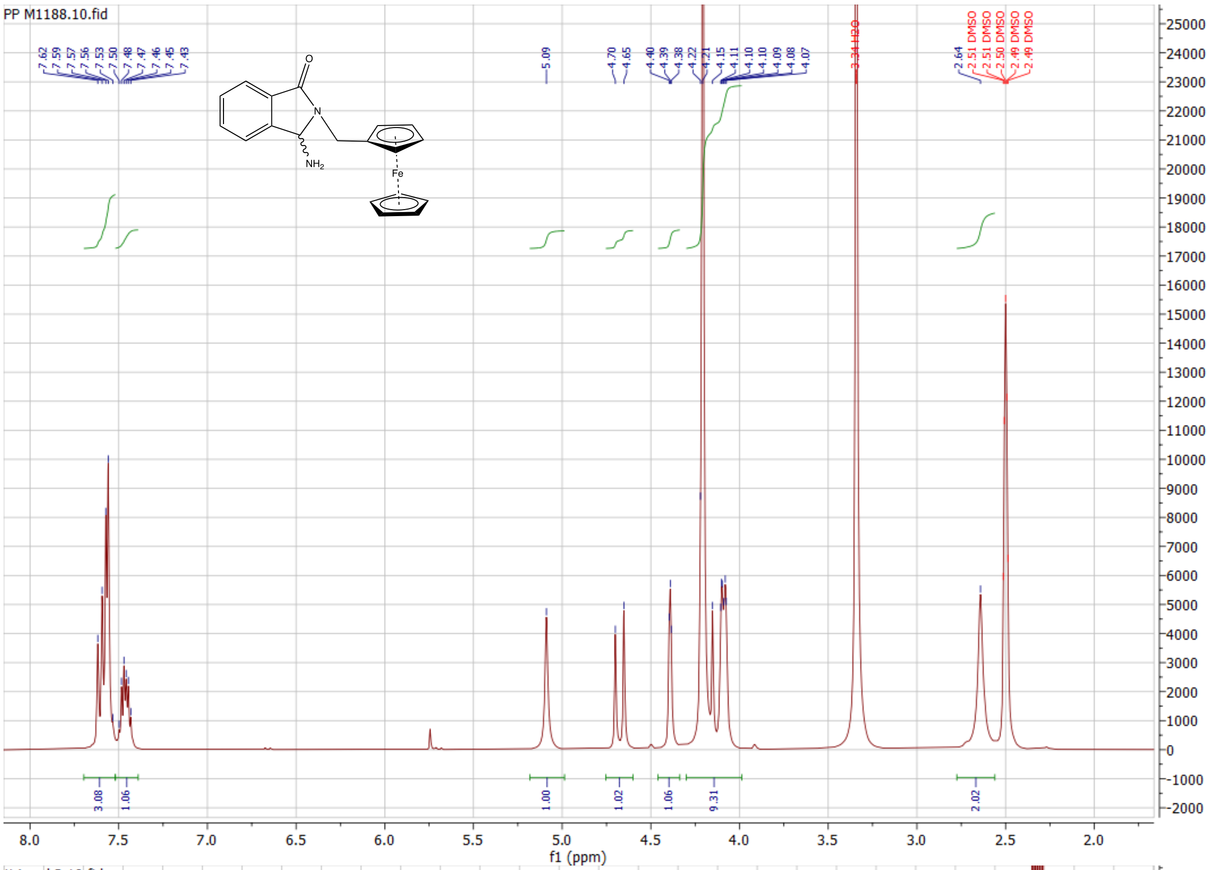

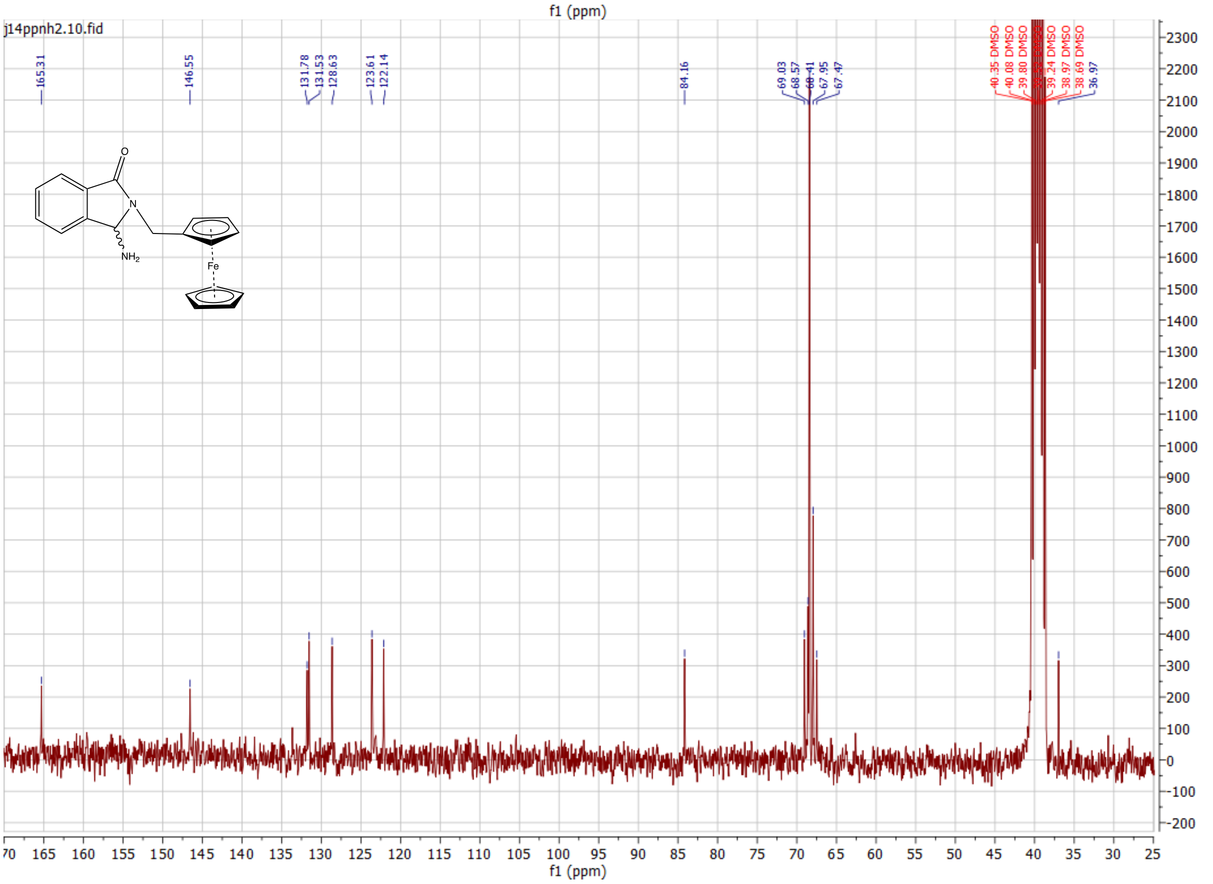

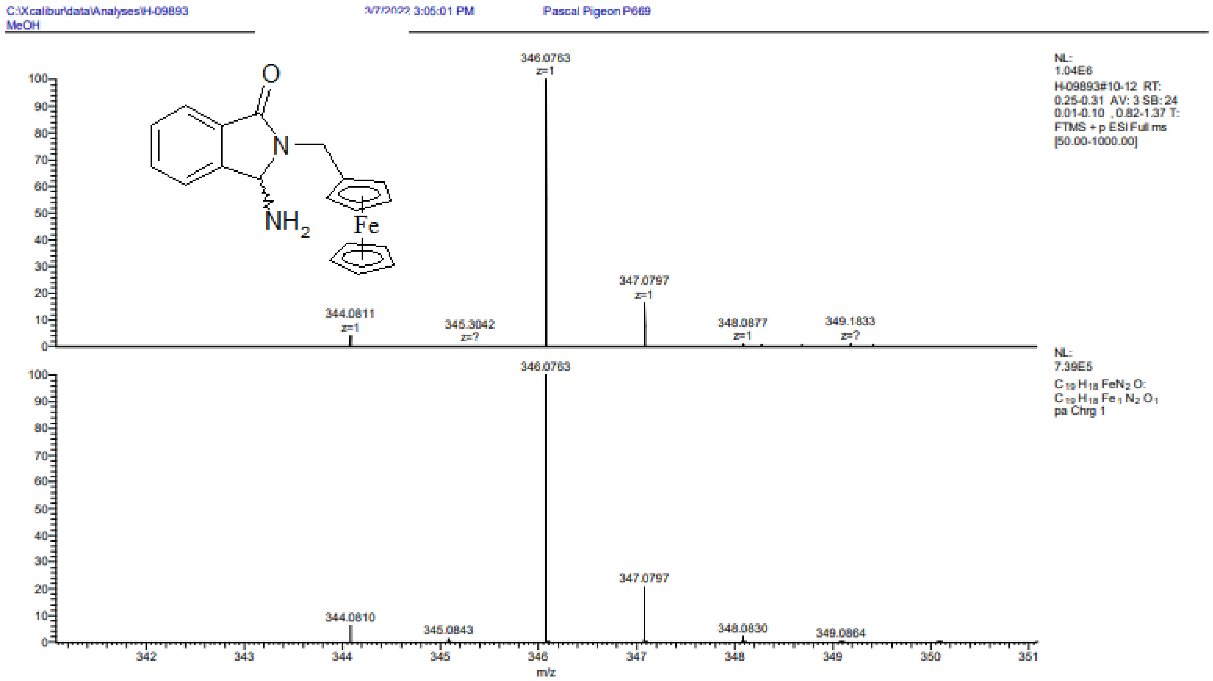

Experimental/theoretical isotopic pattern MS spectrum

Error = 0.0 ppm; Relative Intensity (%) 100

HRMS (ESI) m/z: [M]+ Calcd for C19H18FeN2O 346.0763 . Found 346.0763; (Error: 0.0 ppm).

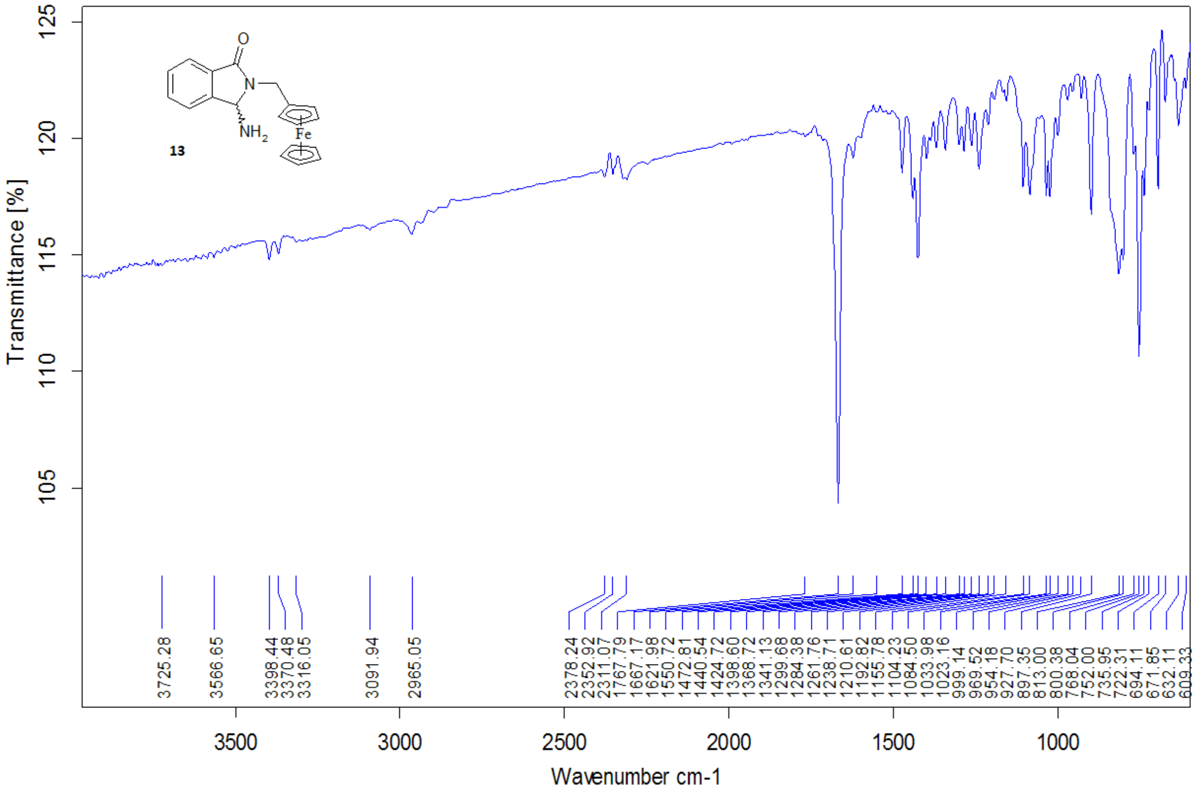

Figure S38: ^{1}H (in CDCl3), ^{13}C (in CDCl3) NMR, HR-MS and IR data for compound **14**

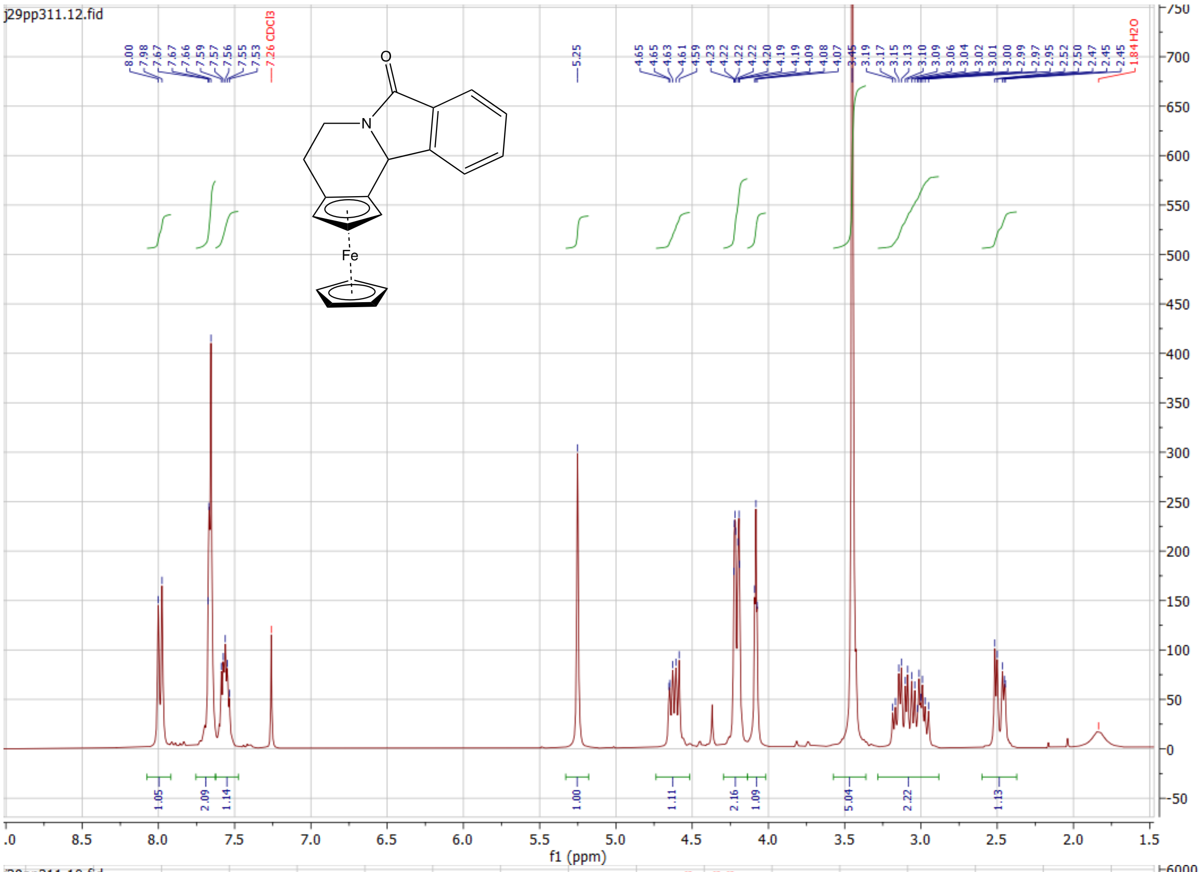

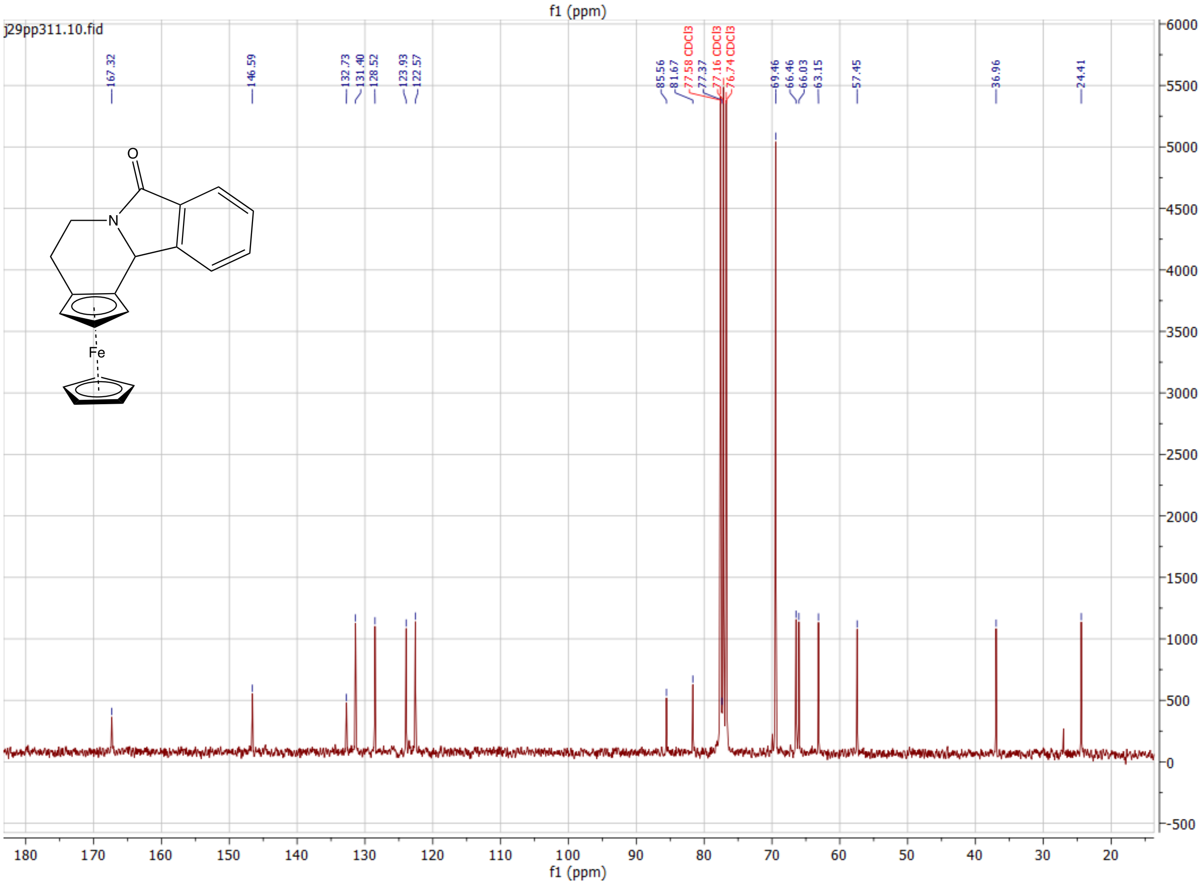

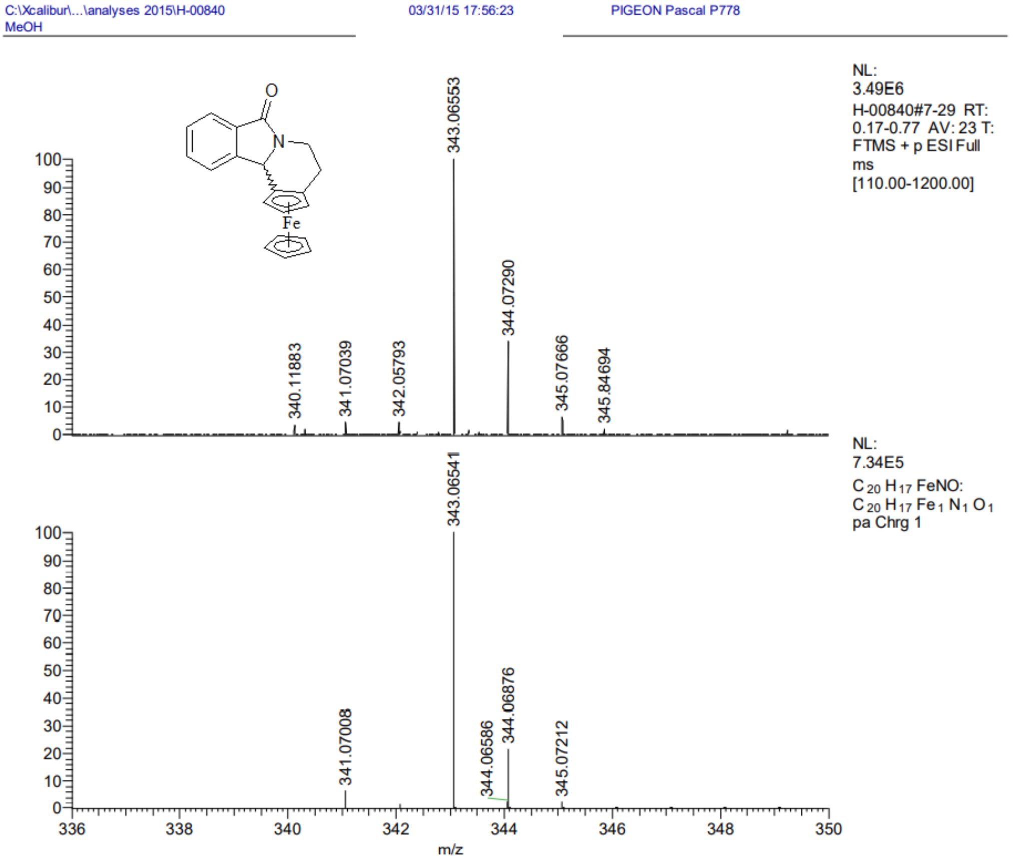

Erreur = 0.4 ppm (erreur calculée pour l'ion expérimental 343.06553 par rapport à la valeur théorique de 343.06541)

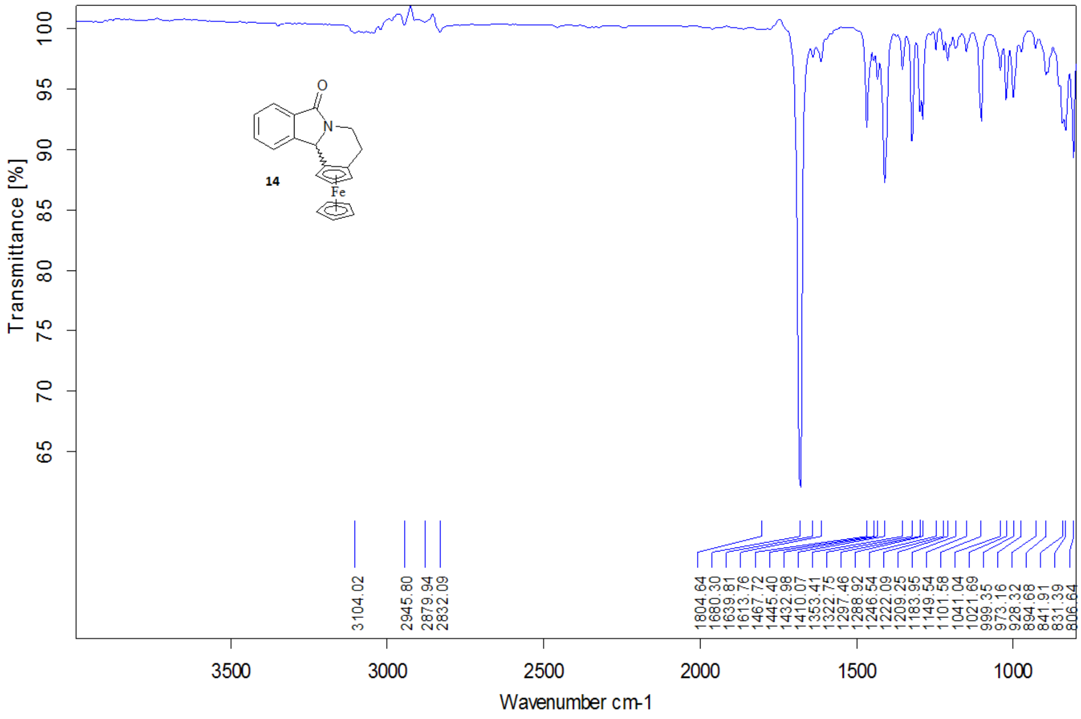

Figure S39: ^{1}H (in $CDCl_3$), ^{13}C (in $CDCl_3$), COSY (in $CDCl_3$), HMQC (in $CDCl_3$) NMR, HR-MS and IR data for compound **15**

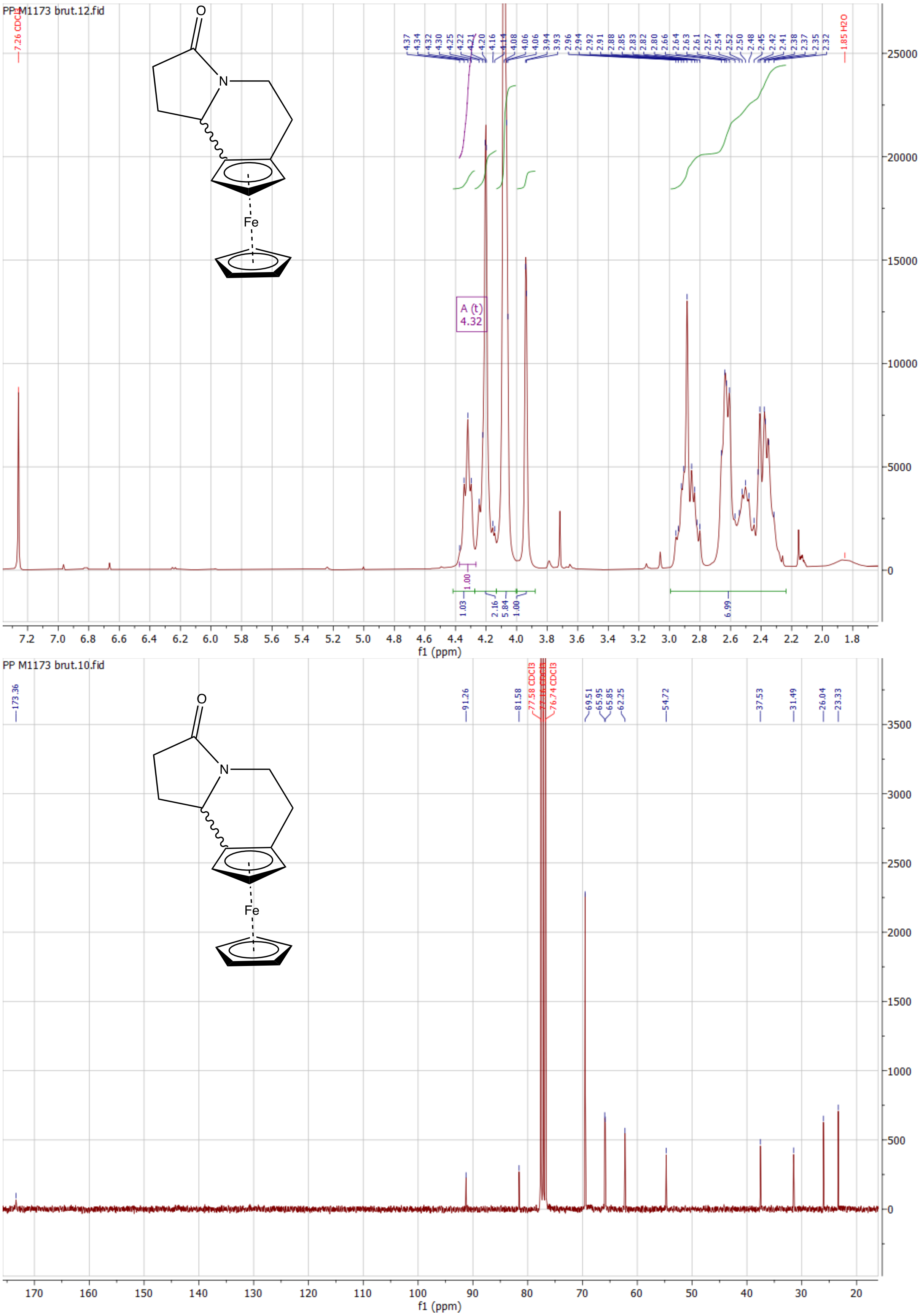

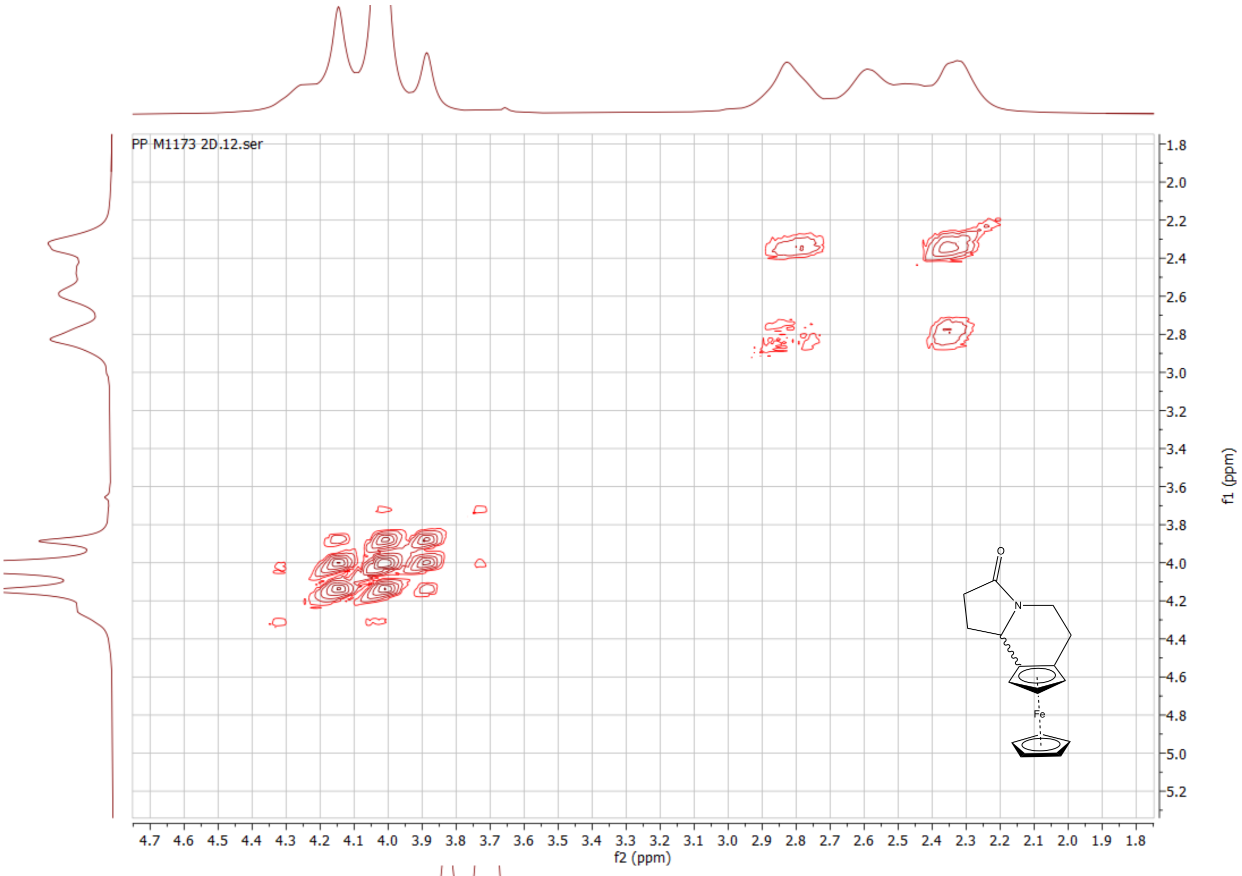
PP M1173 2D.12.ser
f2 (ppm)
f1 (ppm)
O
N
Fe

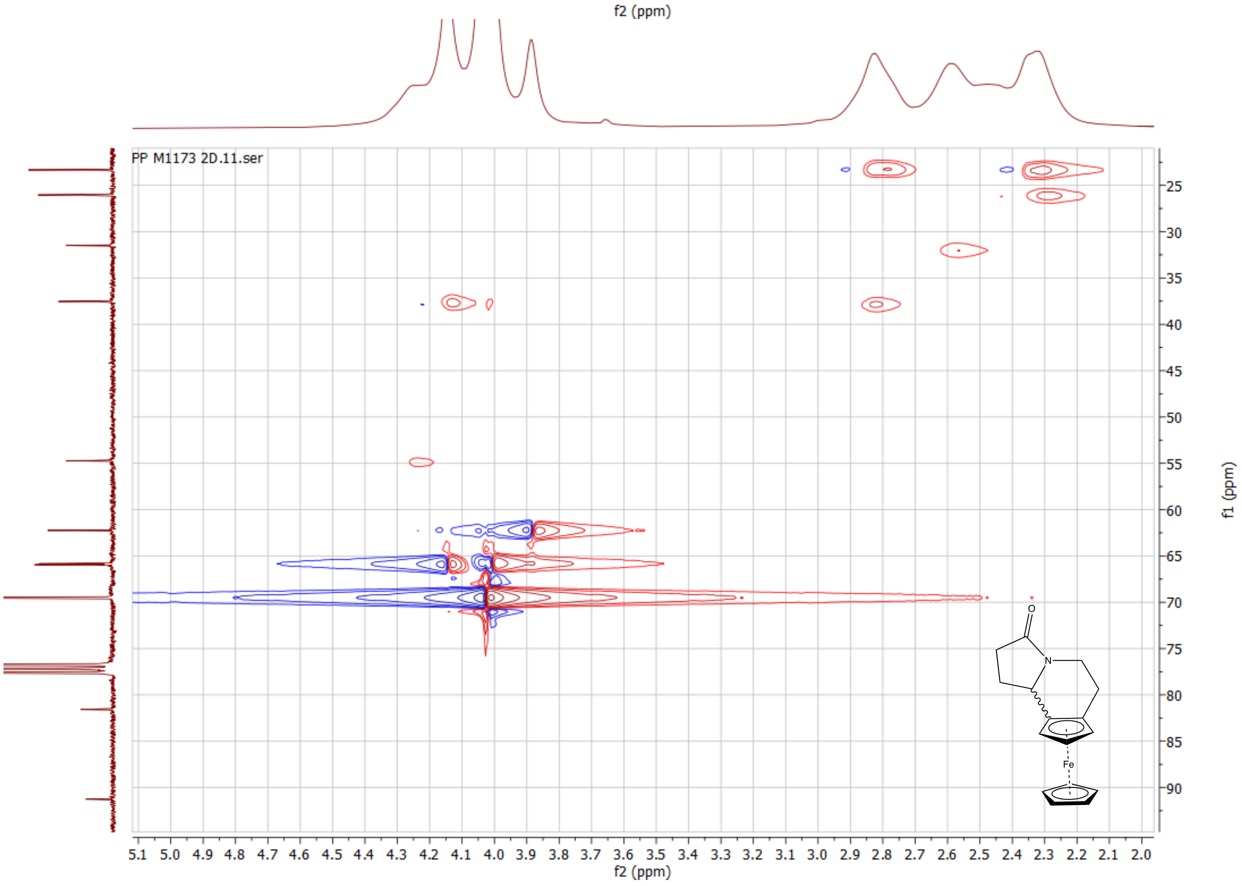
PP M1173 2D.11.ser
f2 (ppm)
f1 (ppm)
O
N
Fe

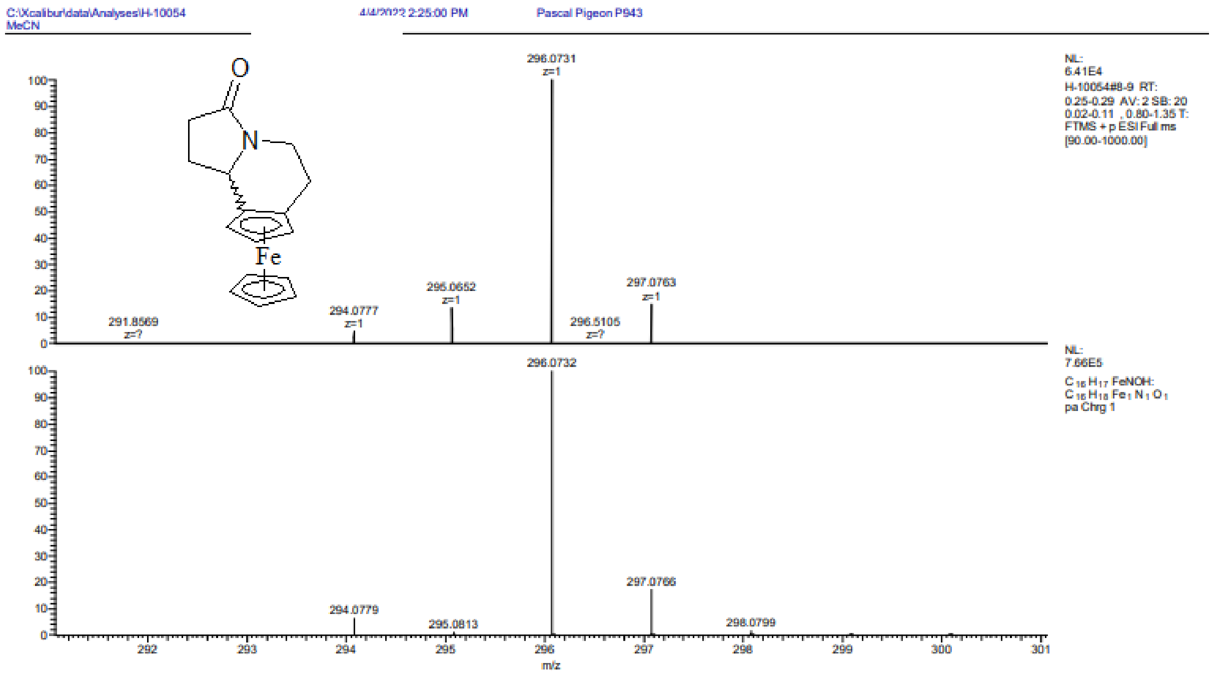

Experimental/theoretical isotopic pattern MS spectrum

Error = -0.4 ppm; Relative Intensity (%) 100

HRMS (ESI) m/z: [M+H]+ Calcd for C16H17FeNOH 296.0732. Found 296.0731; (Error: -0.4 ppm).

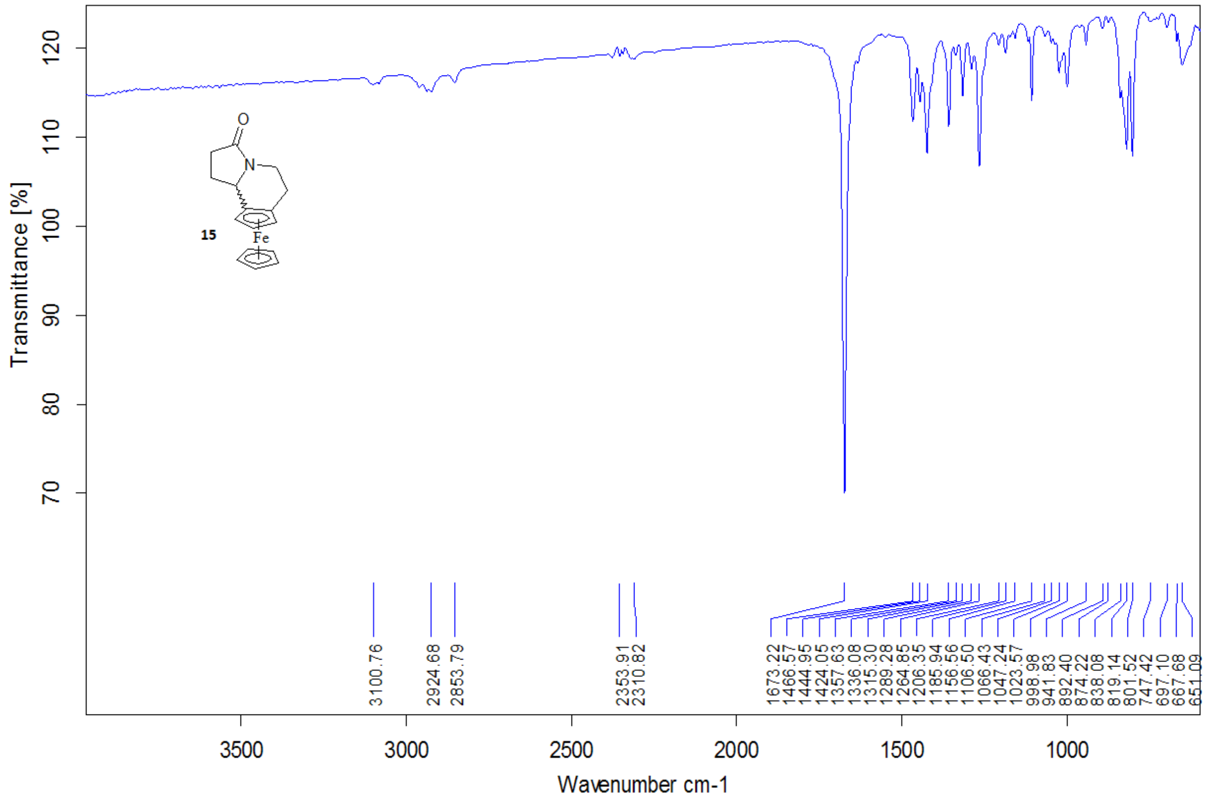

Figure S40: ^{1}H (in $CDCl_3$), ^{13}C (in $CDCl_3$), COSY (in $CDCl_3$), HMQC (in $CDCl_3$) NMR, HR-MS and IR data for compound **16**

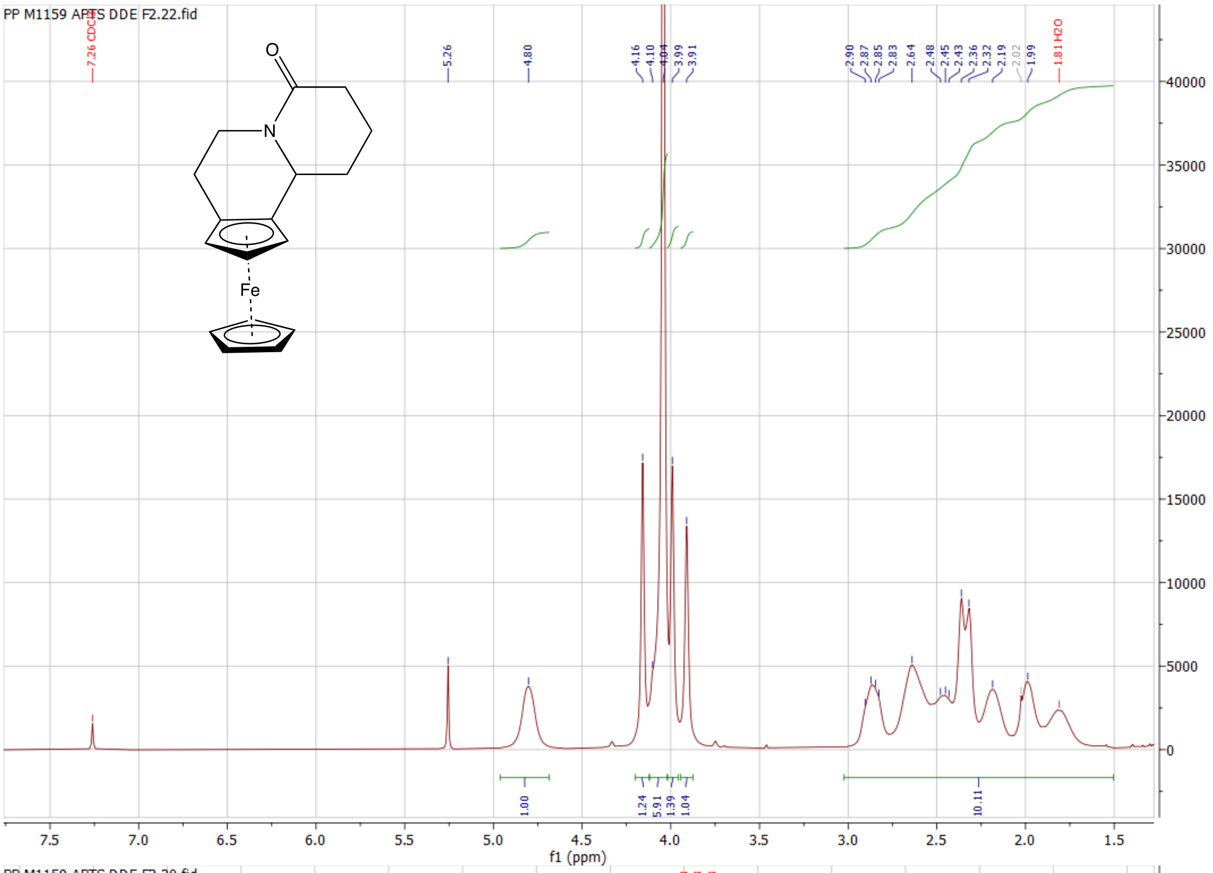

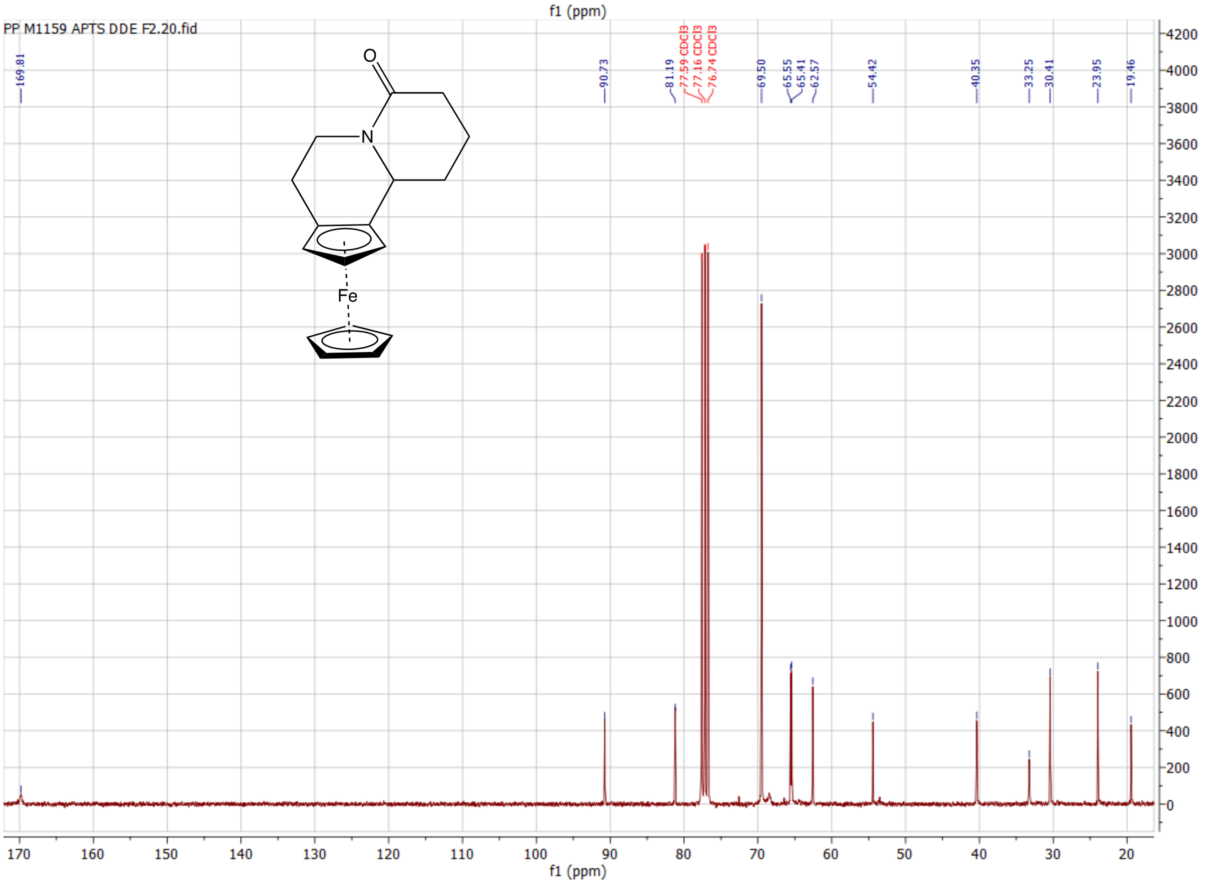

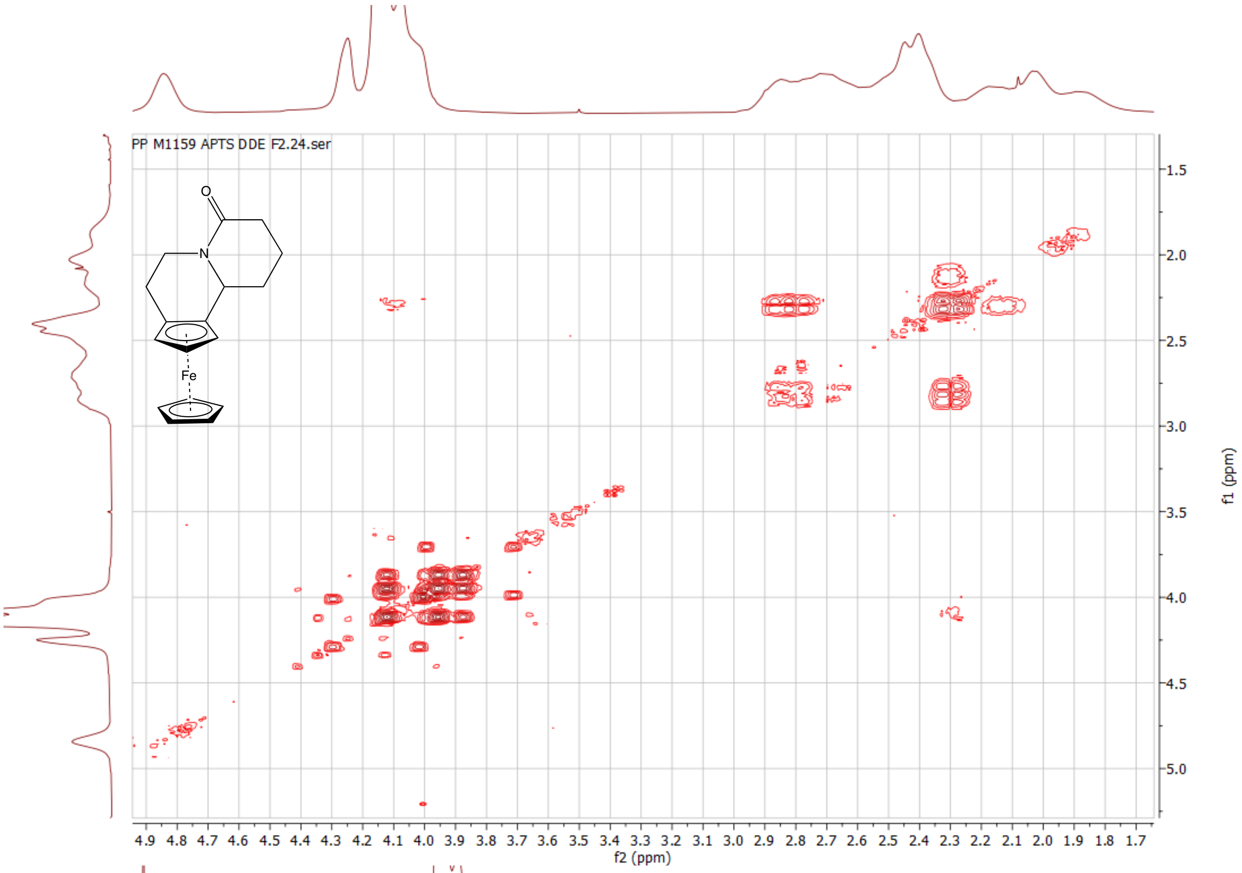
PP M1159 APTS DDE F2.24.ser
O
N
Fe
f2 (ppm)
f1 (ppm)

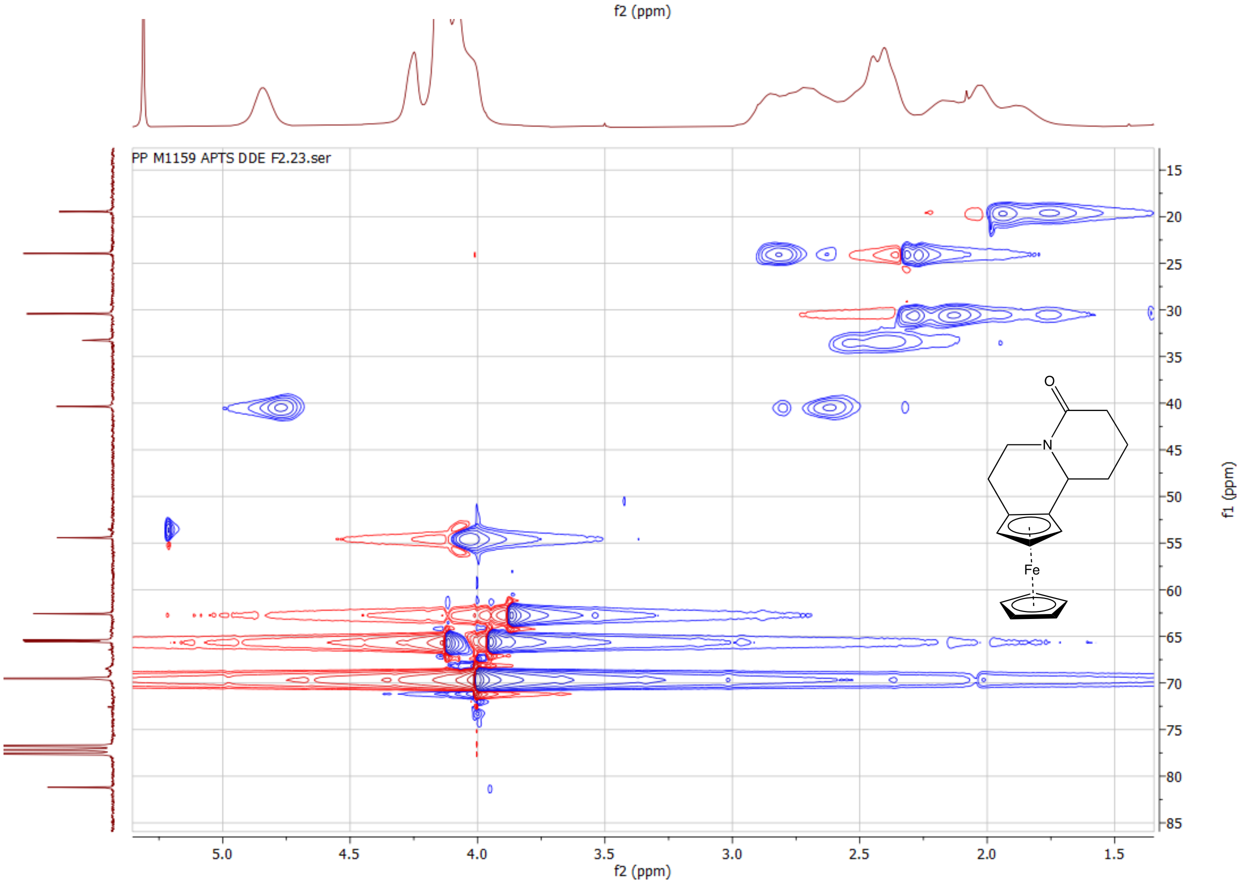
PP M1159 APTS DDE F2.23.ser
O
N
Fe
f2 (ppm)
f1 (ppm)

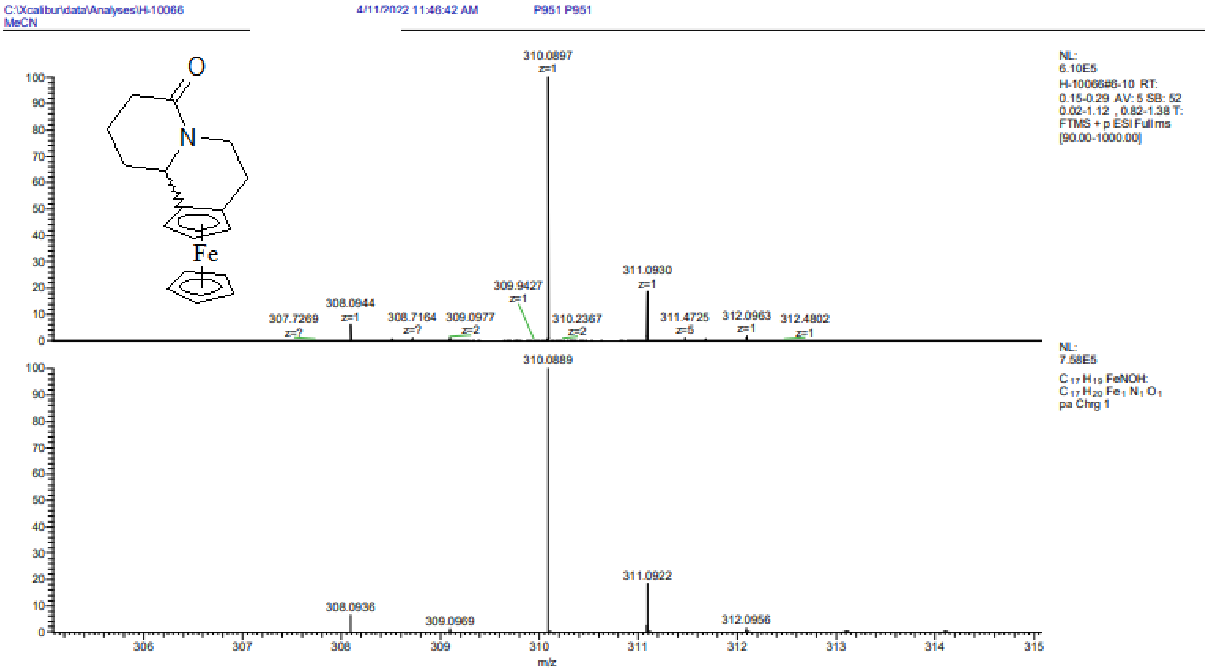

Experimental/theoretical isotopic pattern MS spectrum

Error = 2.7 ppm; Relative Intensity (%) 100

HRMS (ESI) m/z: [M+H]+ Calcd for C17H19FeNOH 310.0889. Found 310.0897; (Error: 2.7 ppm).

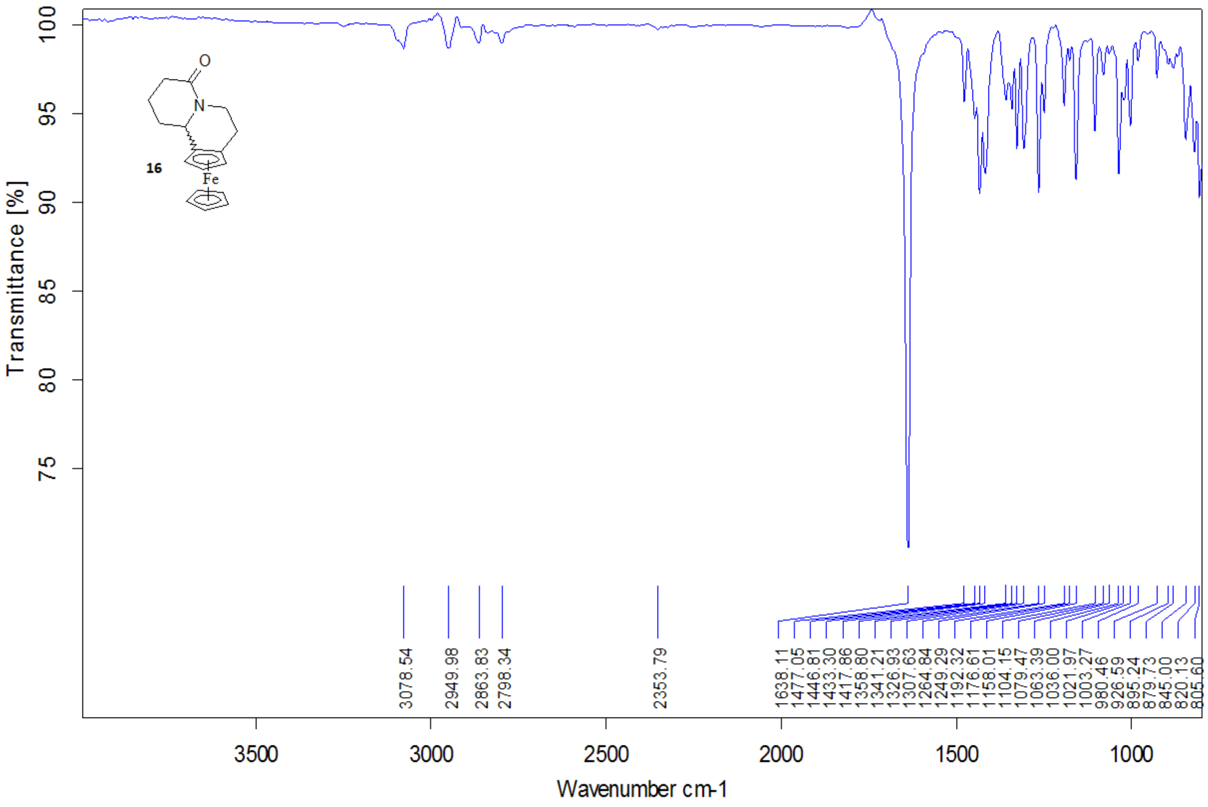

Figure S41: ^{1}H (in DMSO-d6), ^{13}C (in DMSO-d6) and COSY (in DMSO-d6) NMR and IR data for compound **17**

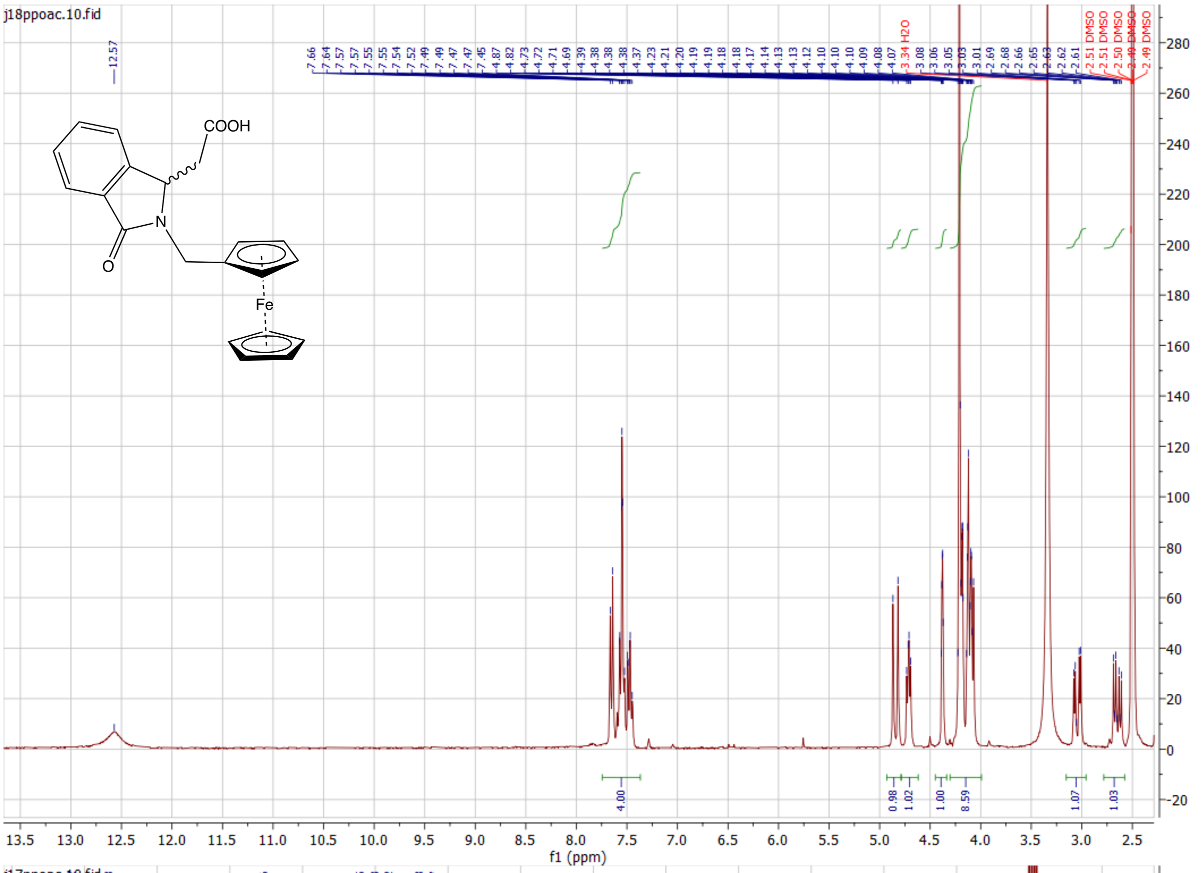

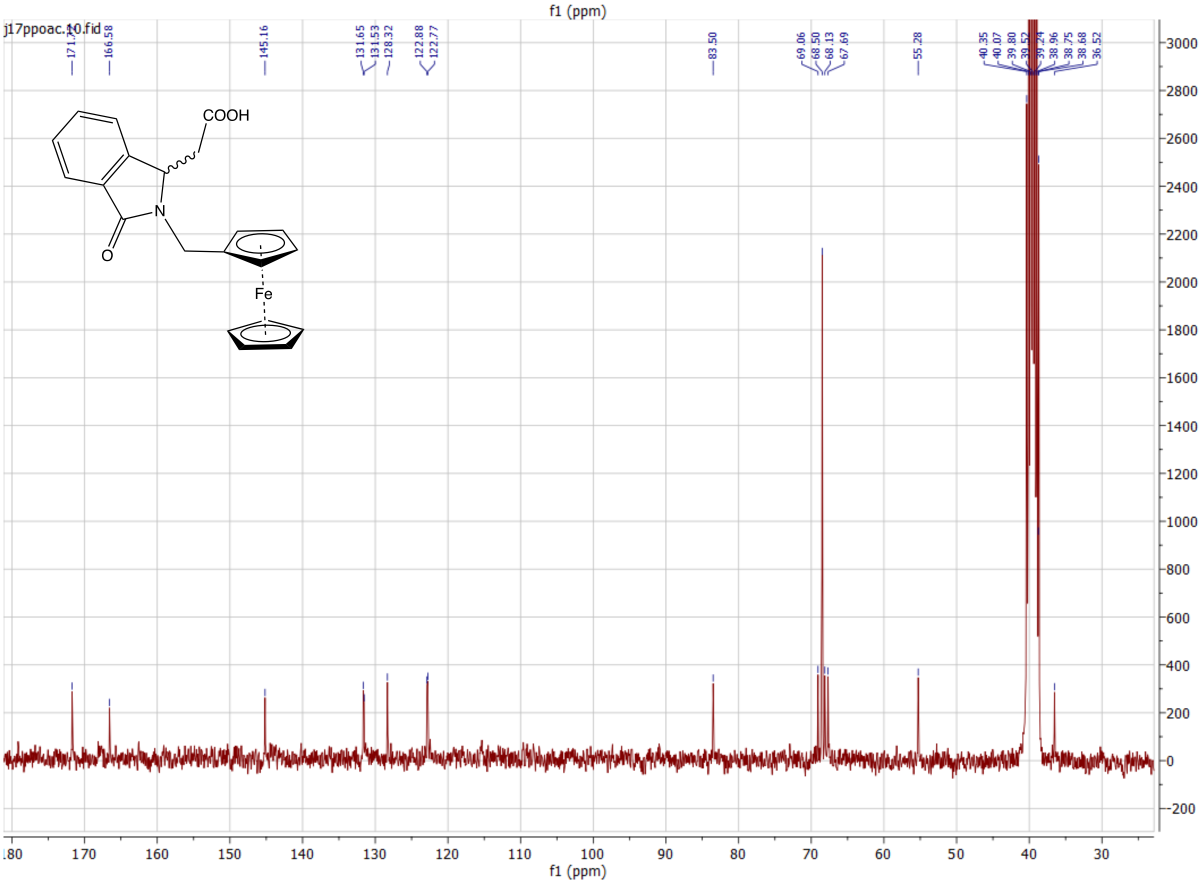

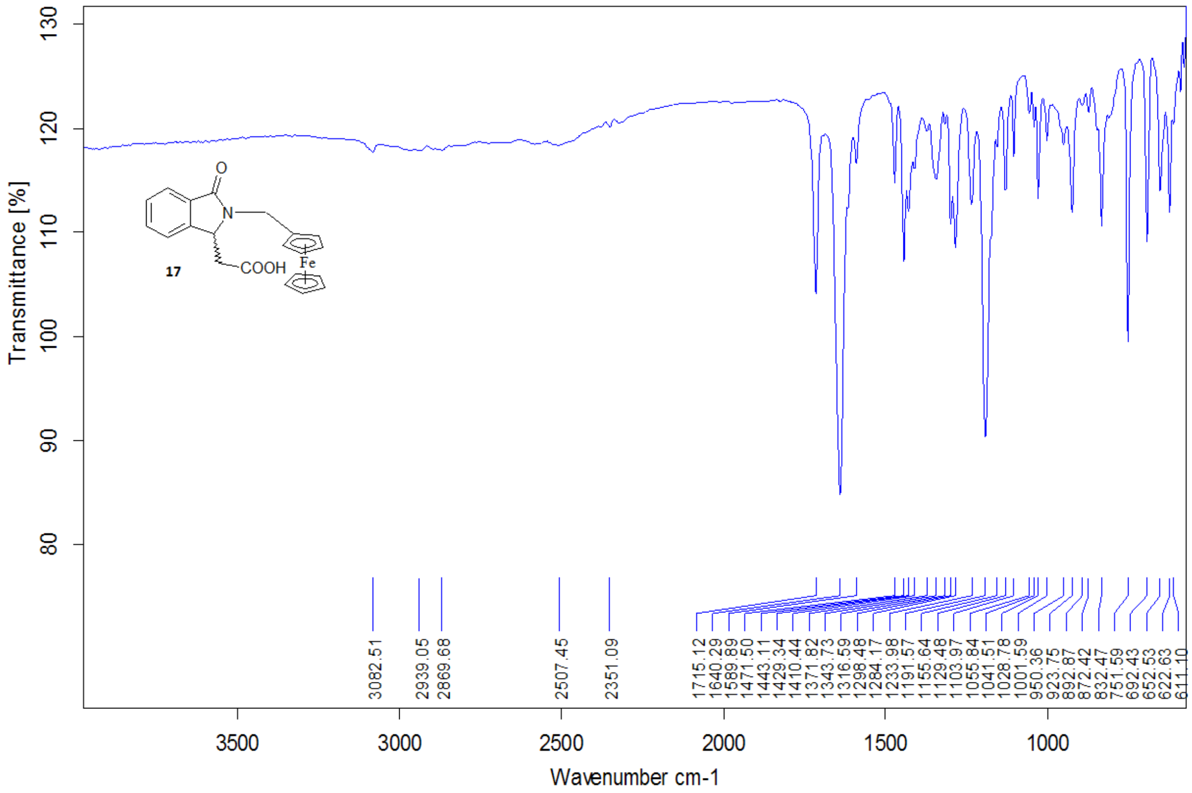

Transmittance [%]
Wavenumber cm-1
130
120
110
100
90
80
3500
3000
2500
2000
1500
1000
O
N
COOH
Fe
17
3082.51
2939.05
2869.68
2507.45
2351.09
1715.12
1640.29
1589.89
1471.50
1443.11
1429.34
1410.44
1371.82
1343.73
1316.59
1298.48
1284.17
1233.98
1191.57
1155.64
1129.48
1103.97
1055.84
1041.51
1028.78
1001.59
950.36
923.75
892.87
872.42
832.47
751.59
692.43
652.53
622.63
611.10

Figure S42: ^{1}H (in DMSO-d6) and ^{13}C (in DMSO-d6) NMR, HR-MS and IR data for compound **20**

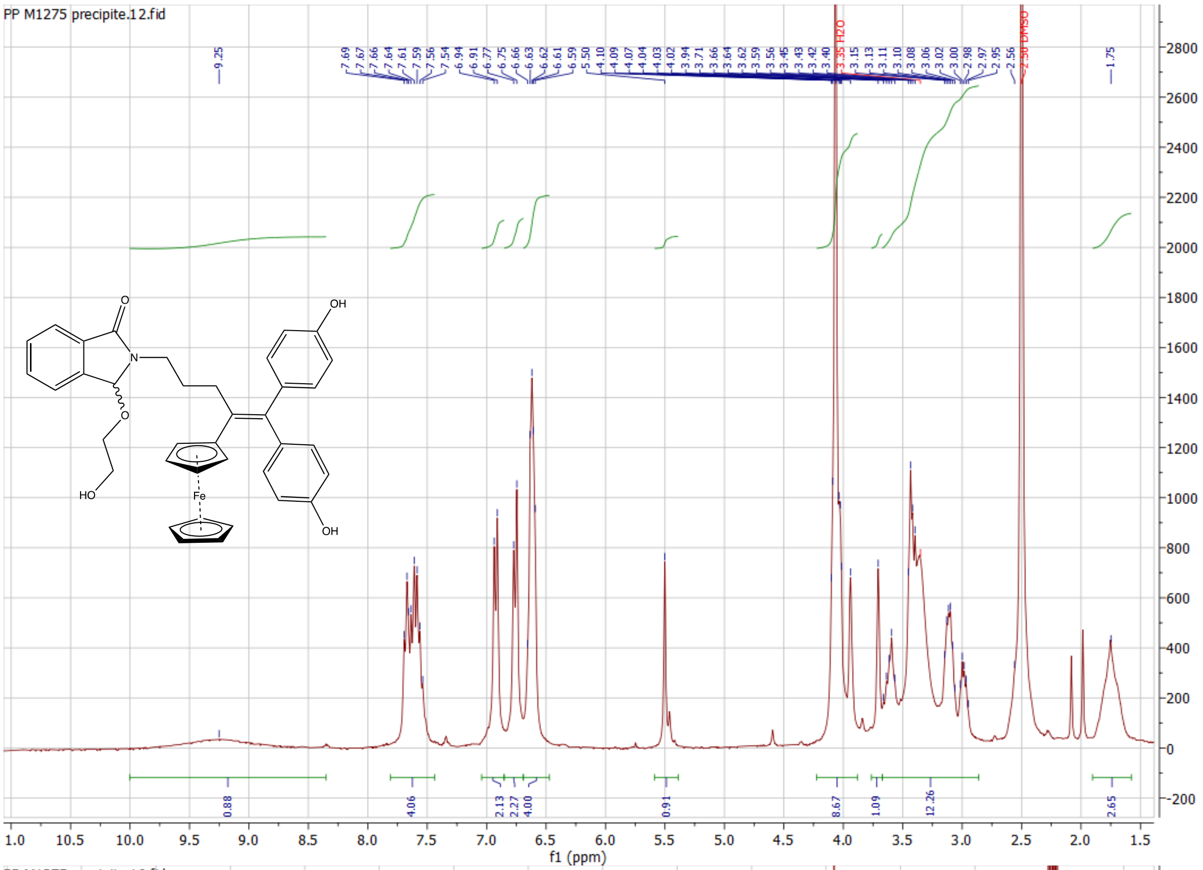

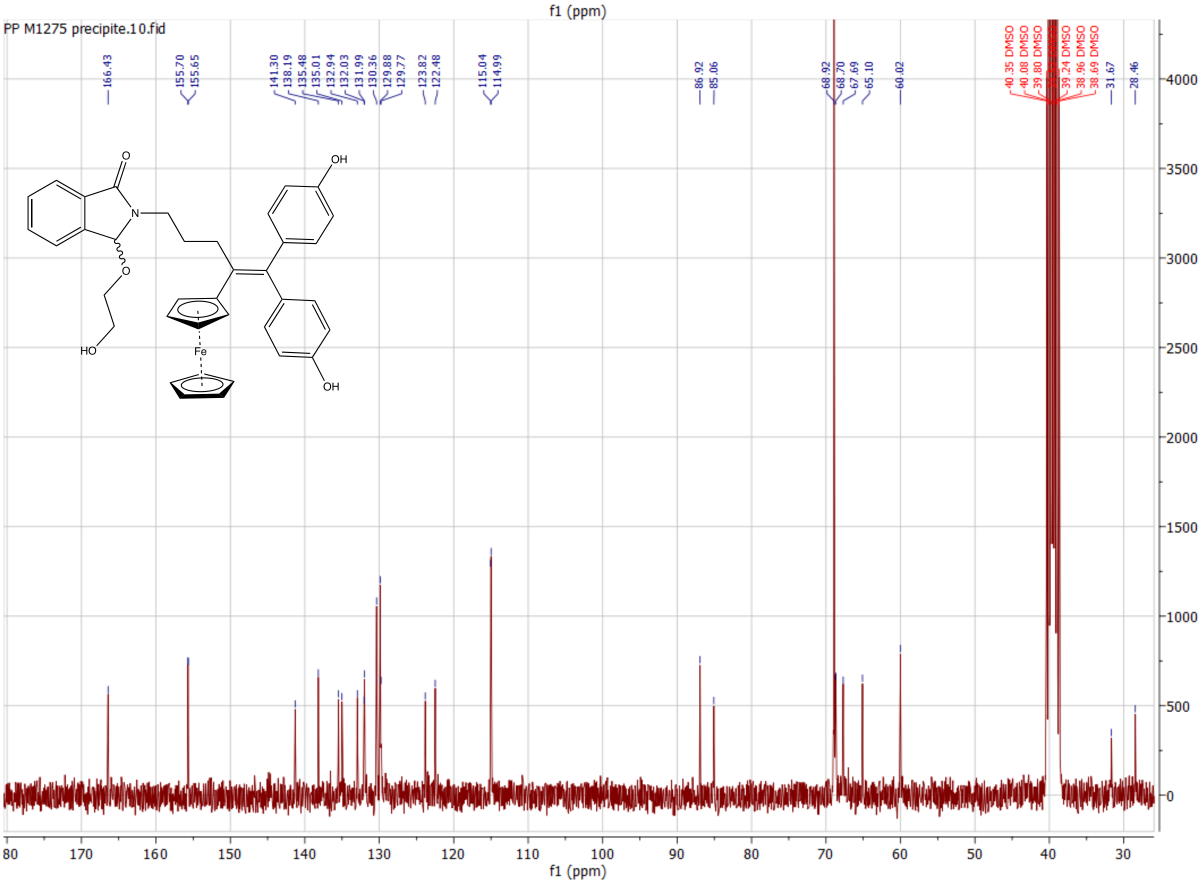

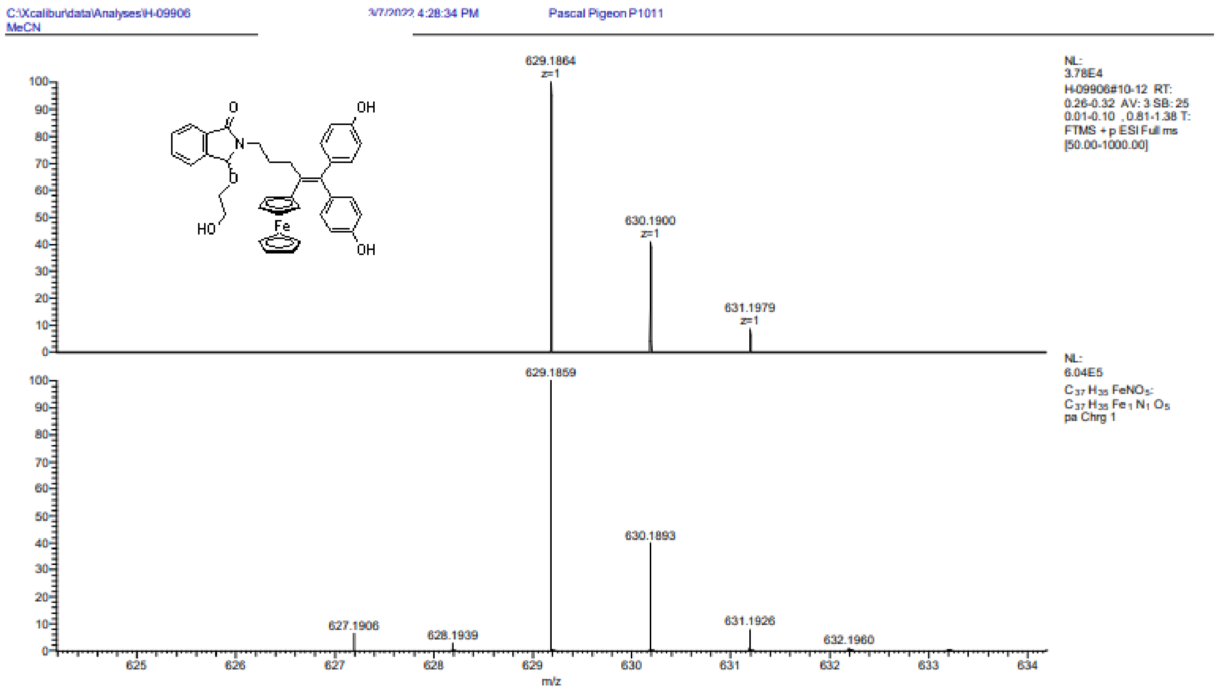

Experimental/theoretical isotopic pattern MS spectrum

Error = 0.8 ppm; Relative Intensity (%) 100

HRMS (ESI) m/z: [M]+ Calcd for C37H35FeNO5 629.1859 . Found 629.1864; (Error: 0.8 ppm).

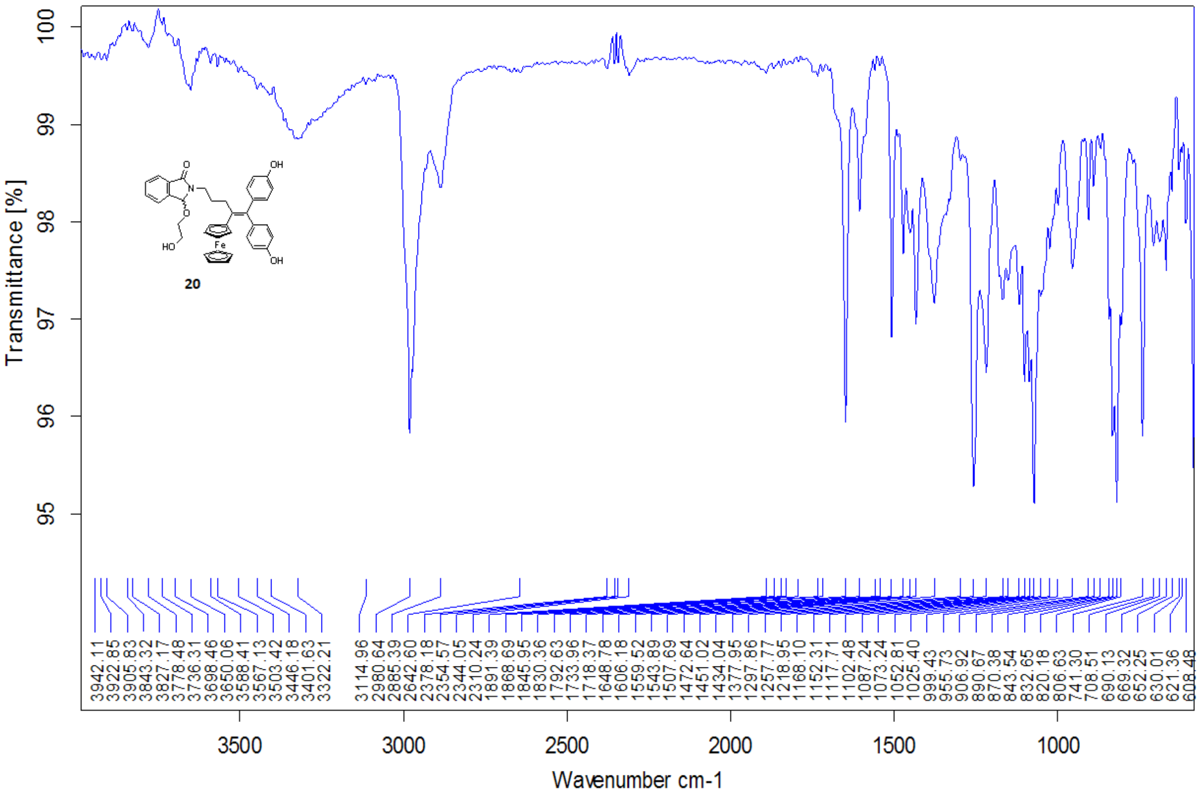

Figure S43: ^{1}H (in acetone-d$_6$), ^{13}C (in acetone-d$_6$) and COSY (in acetone-d$_6$) NMR, HR-MS and IR data for compound **21**

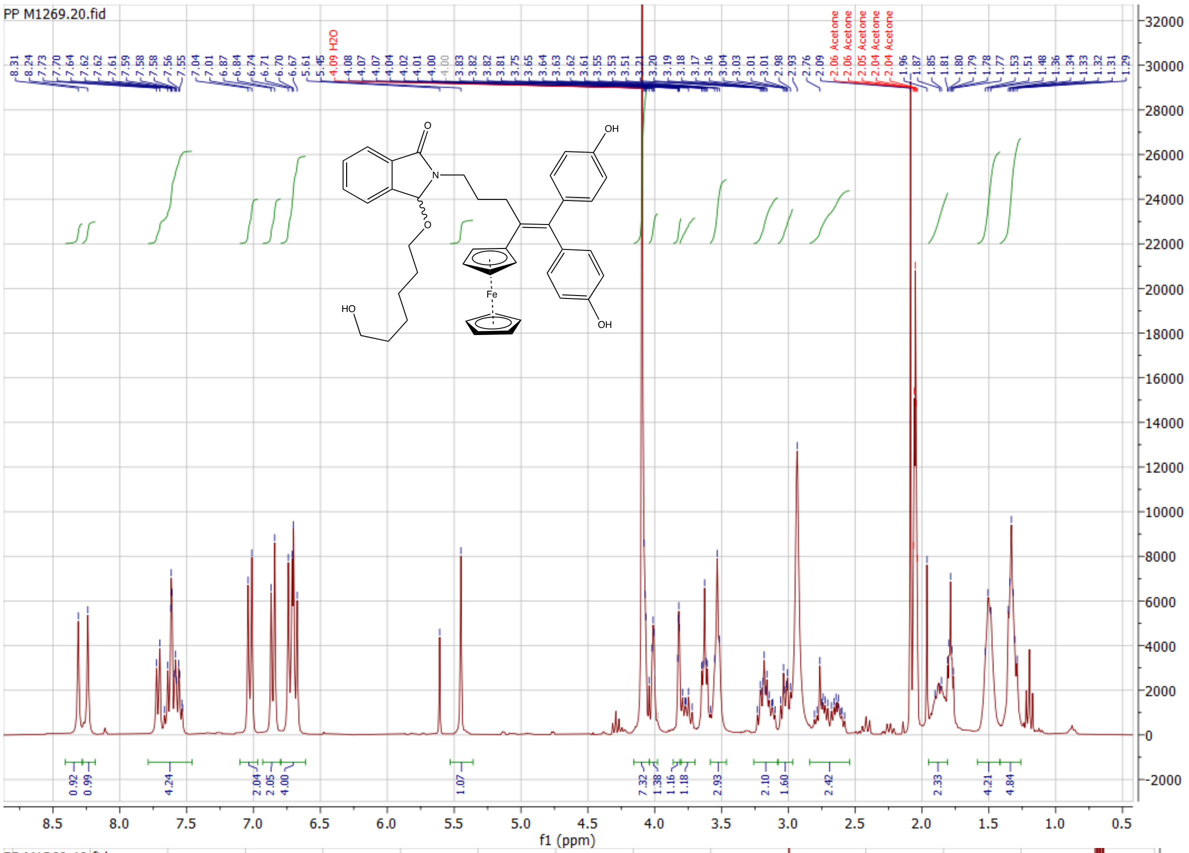

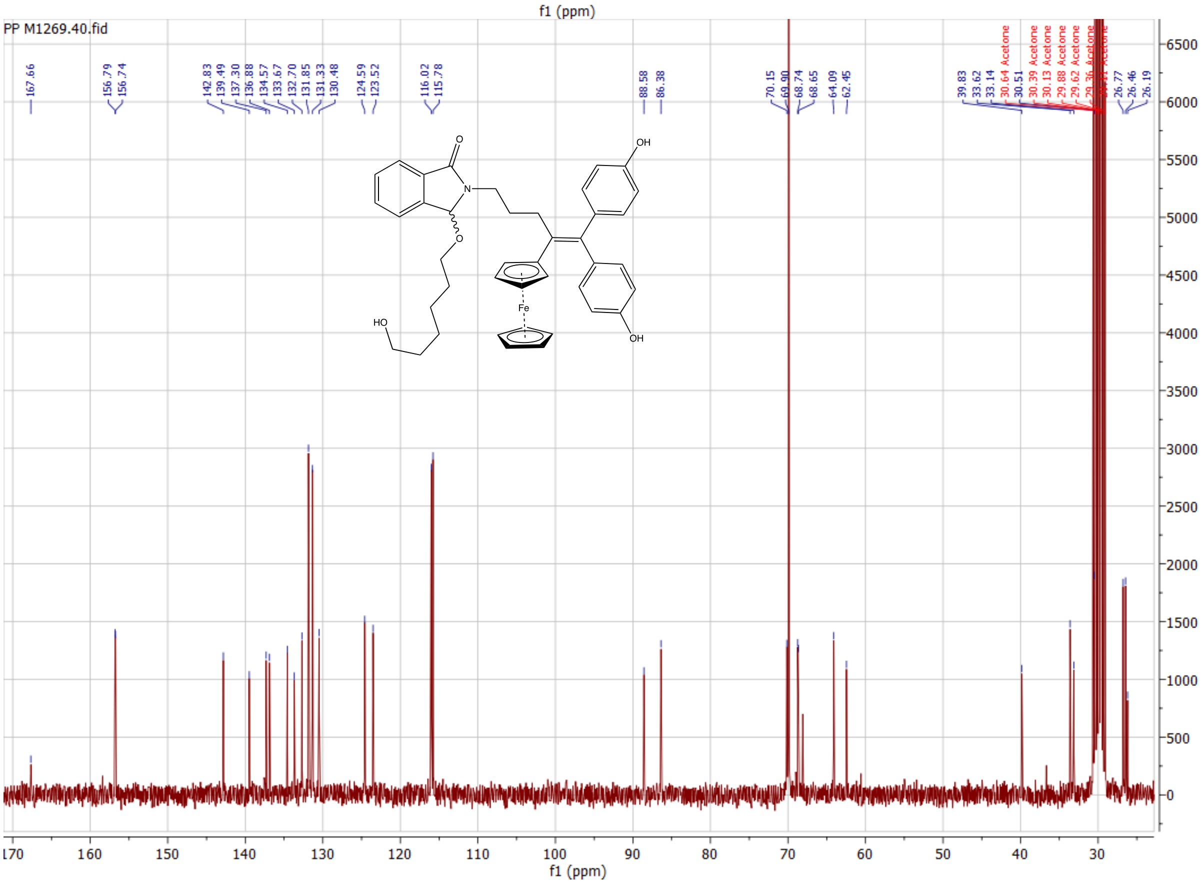

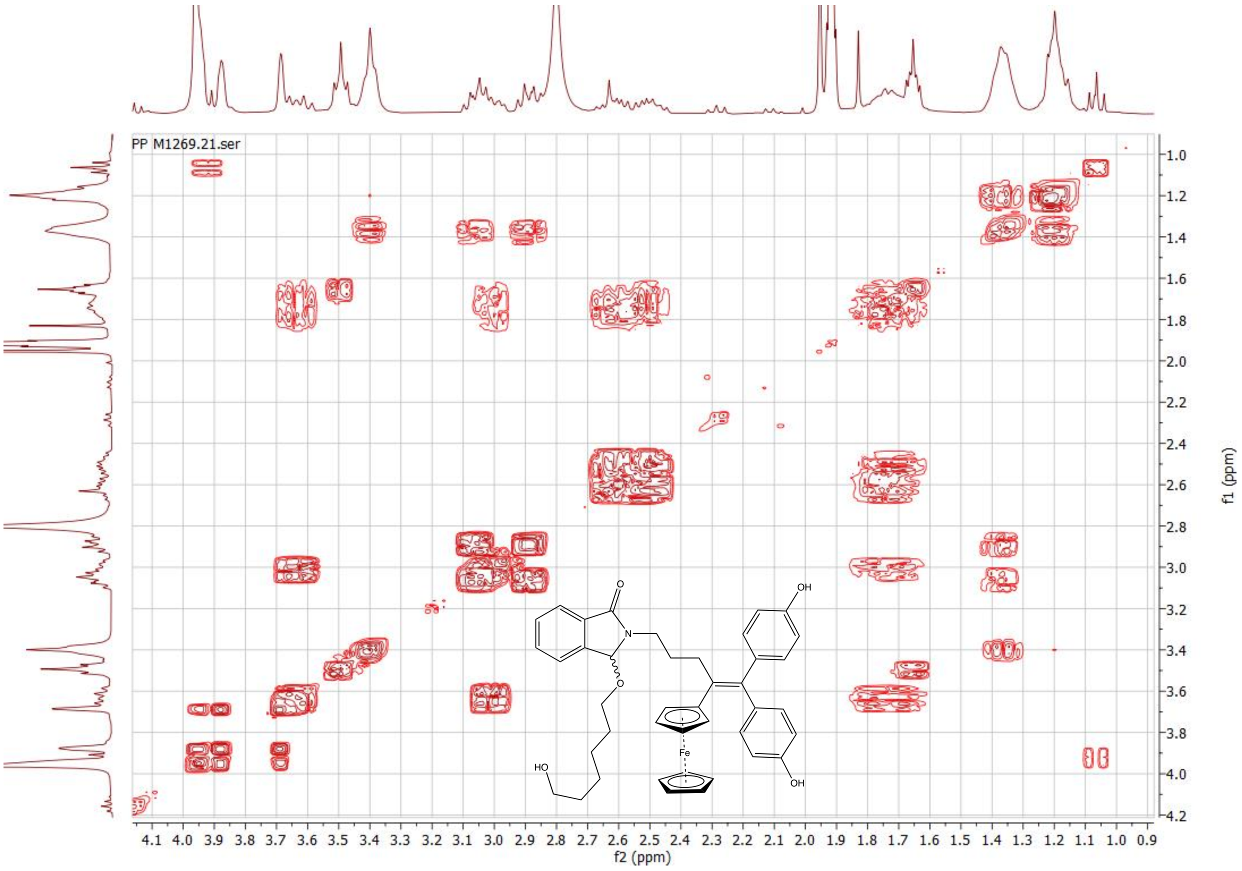

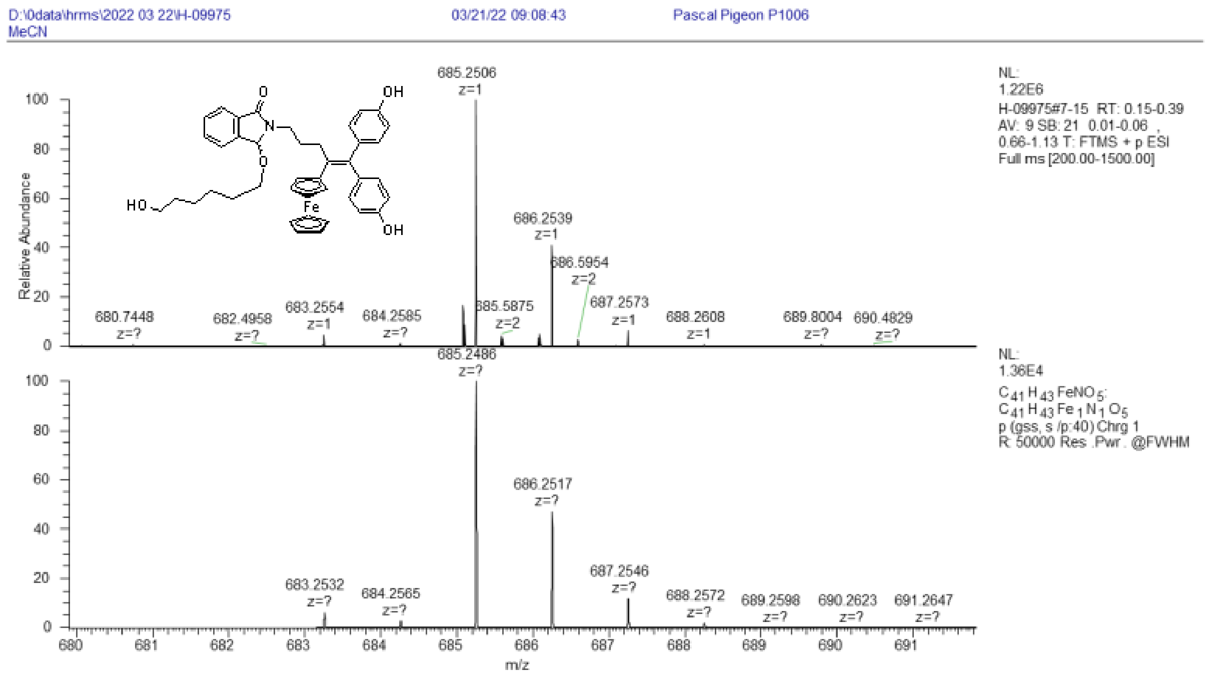

Experimental/theoretical isotopic pattern MS spectrum

Error = 2.9 ppm; Relative Intensity (%) 100

HRMS (ESI) m/z: [M]+ Calcd for C41H43FeNO5 685.2486 . Found 685.2506; (Error: 2.9 ppm).

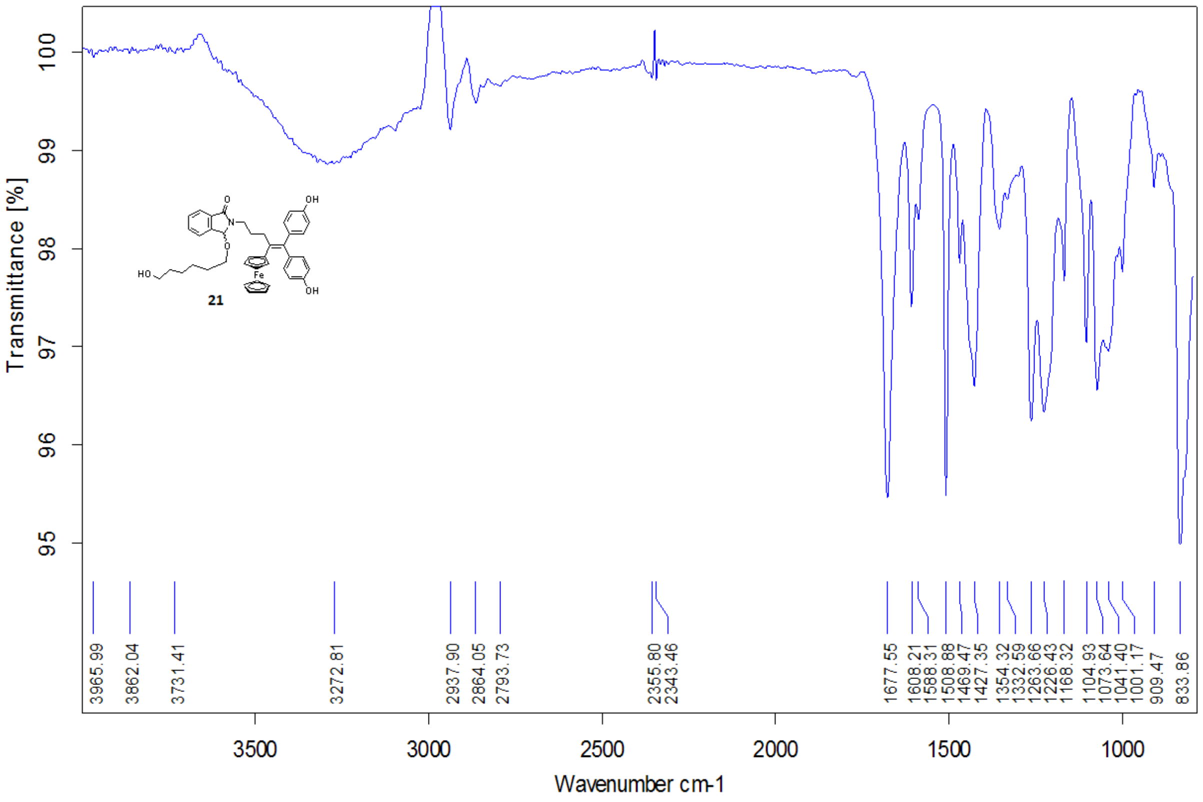
Transmittance [%]
100
99
98
97
96
95
3965.99
3862.04
3731.41
3272.81
2937.90
2864.05
2793.73
2355.80
2343.46
1677.55
1608.21
1588.31
1508.88
1469.47
1427.35
1354.32
1332.59
1263.66
1226.43
1168.32
1104.93
1073.64
1041.40
1001.17
909.47
833.86
3500
3000
2500
2000
1500
1000
Wavenumber cm-1
21

Figure 44: COSY (in acetone-d6), ^{1}H (in acetone-d6), ^{13}C (in acetone-d6) NMR, HR-MS and IR data for compound **22**

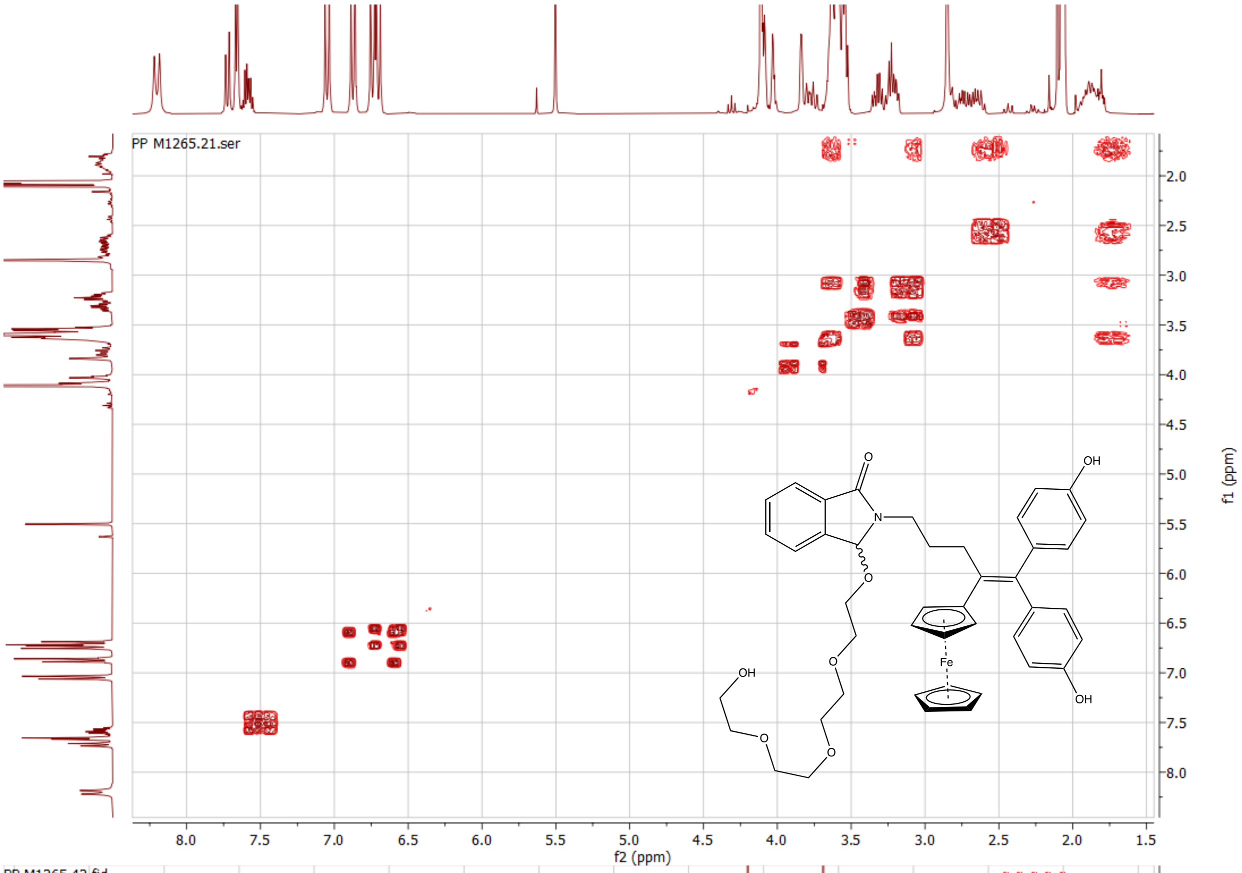

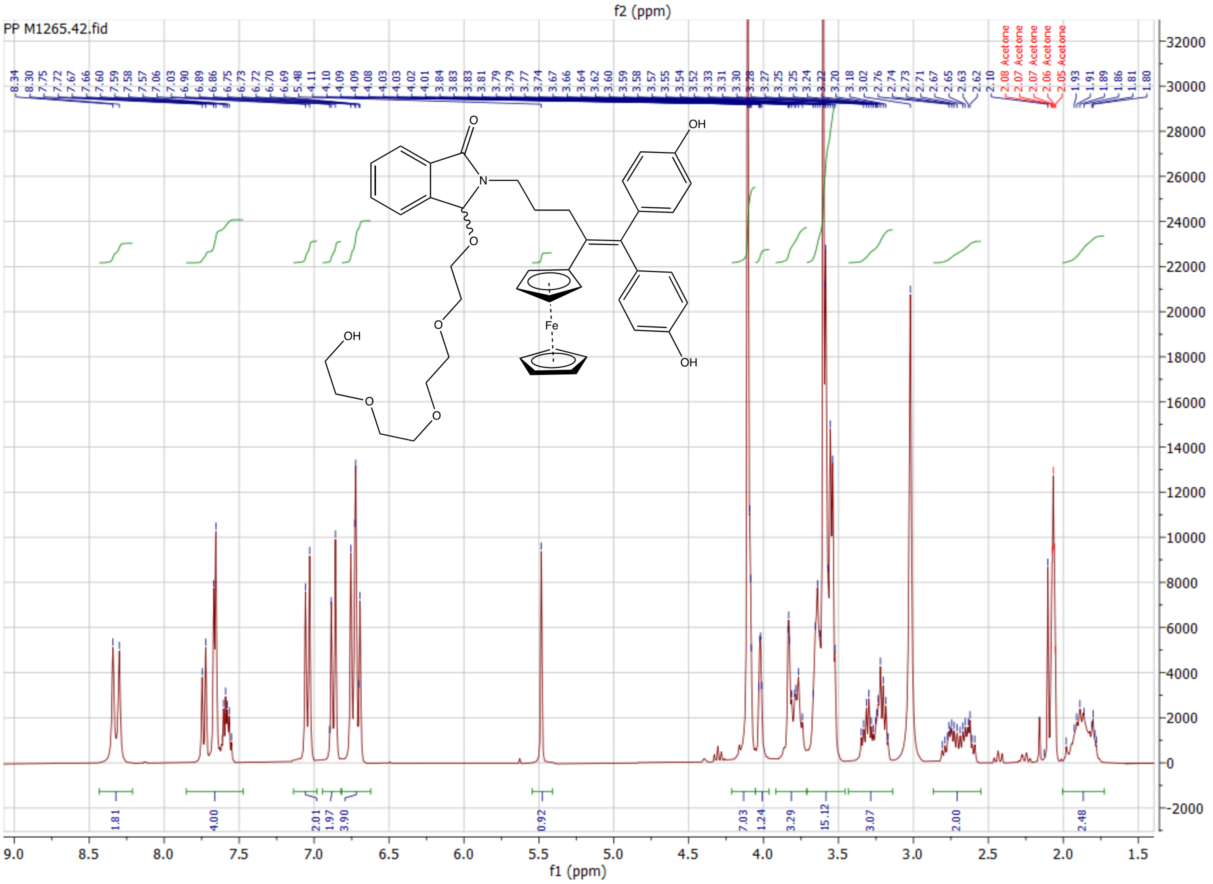

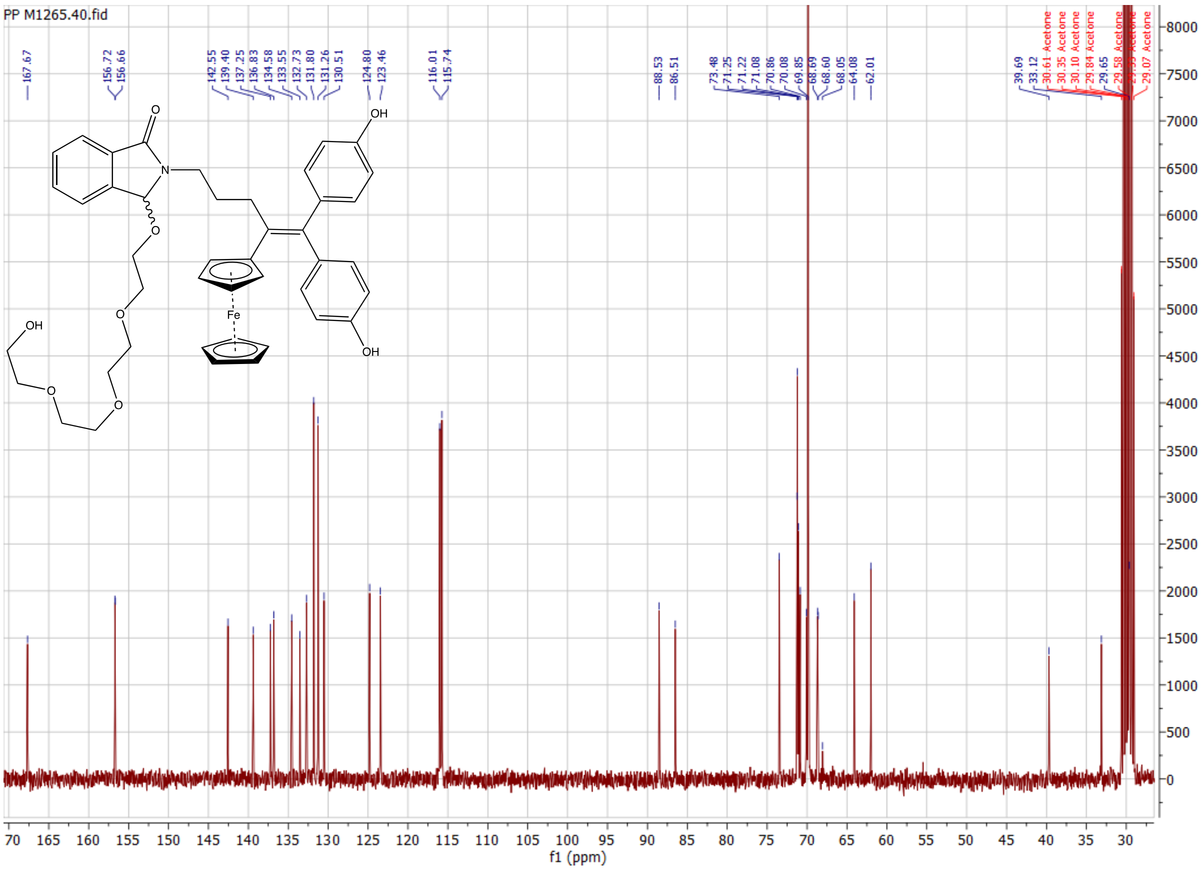

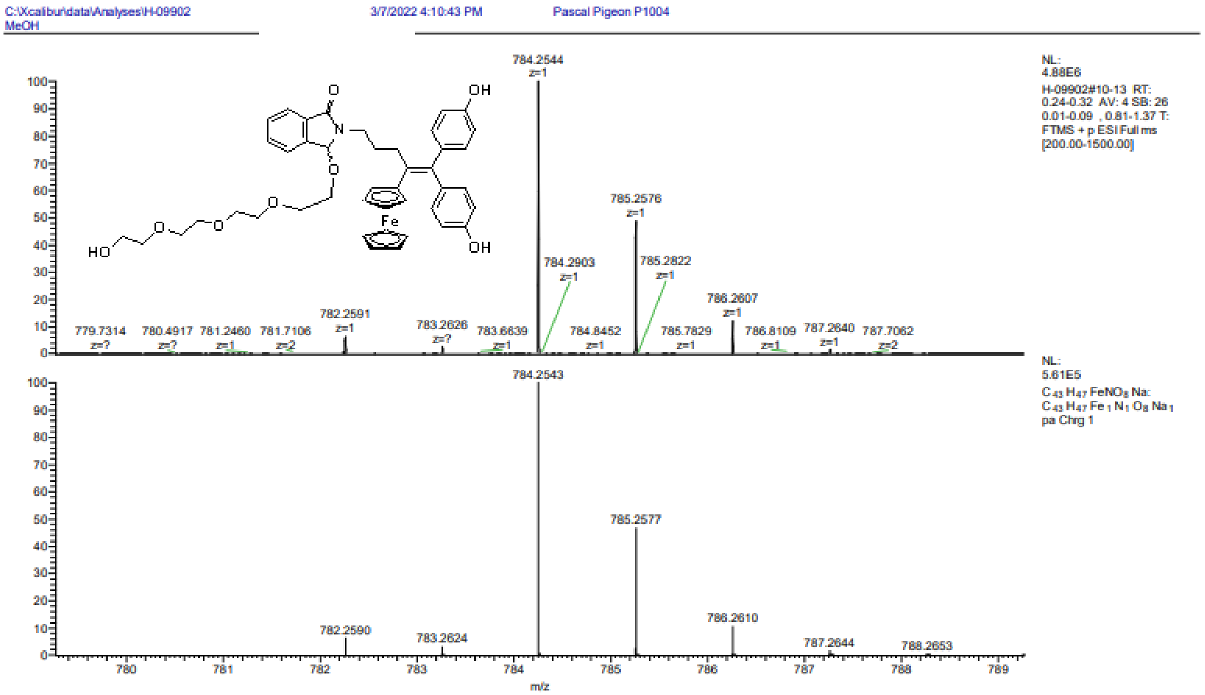

Experimental/theoretical isotopic pattern MS spectrum

Error = 0.1 ppm; Relative Intensity (%) 100

HRMS (ESI) m/z: [M+Na]+ Calcd for C43H47FeNO8Na 784.2543. Found 784.2544; (Error: 0.1 ppm).

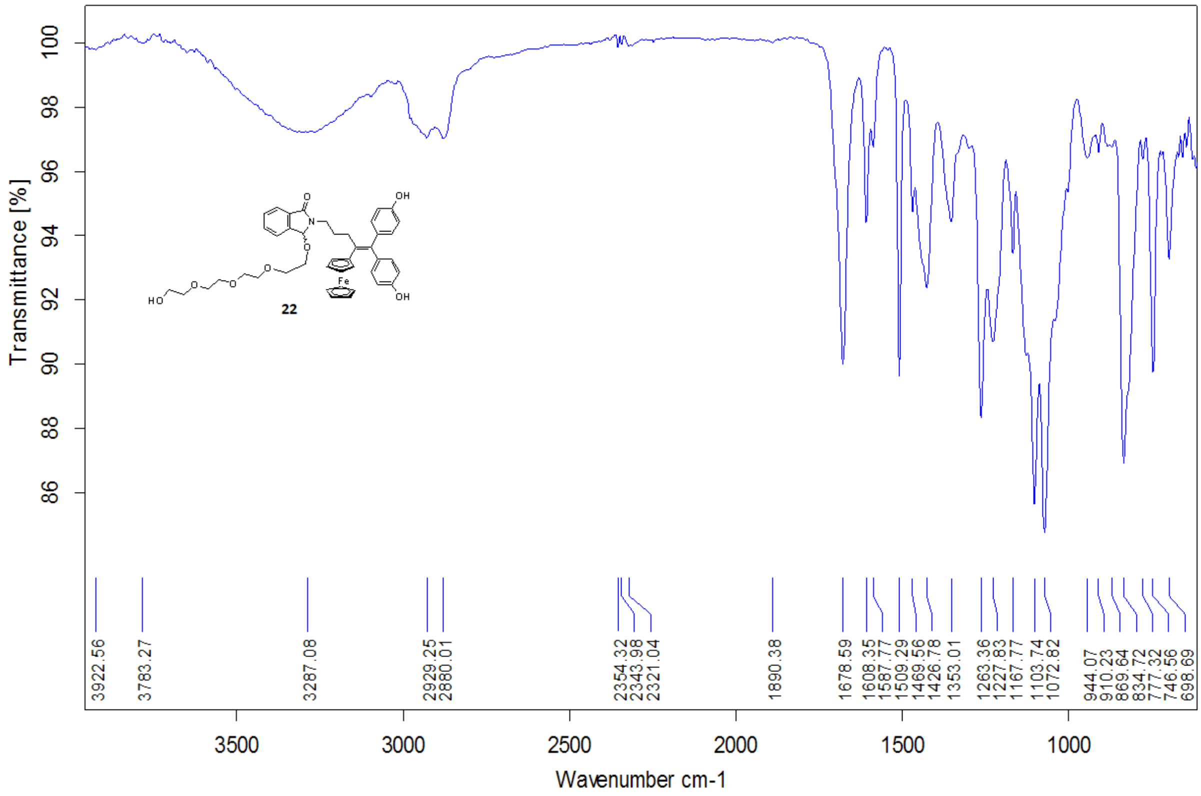

Transmittance [%]
100
98
96
94
92
90
88
86
22
OH
OH
HO
O
N
Fe
3922.56
3783.27
3287.08
2929.25
2880.01
2354.32
2343.98
2321.04
1890.38
1678.59
1608.35
1587.77
1509.29
1469.56
1426.78
1353.01
1263.36
1227.83
1167.77
1103.74
1072.82
944.07
910.23
869.64
834.72
777.32
746.56
698.69
3500
3000
2500
2000
1500
1000
Wavenumber cm-1

Figure S45: ¹H (in acetone-d₆), JMOD (in acetone-d₆), COSY (in acetone-d₆) NMR, HR-MS and IR data for compound **23**

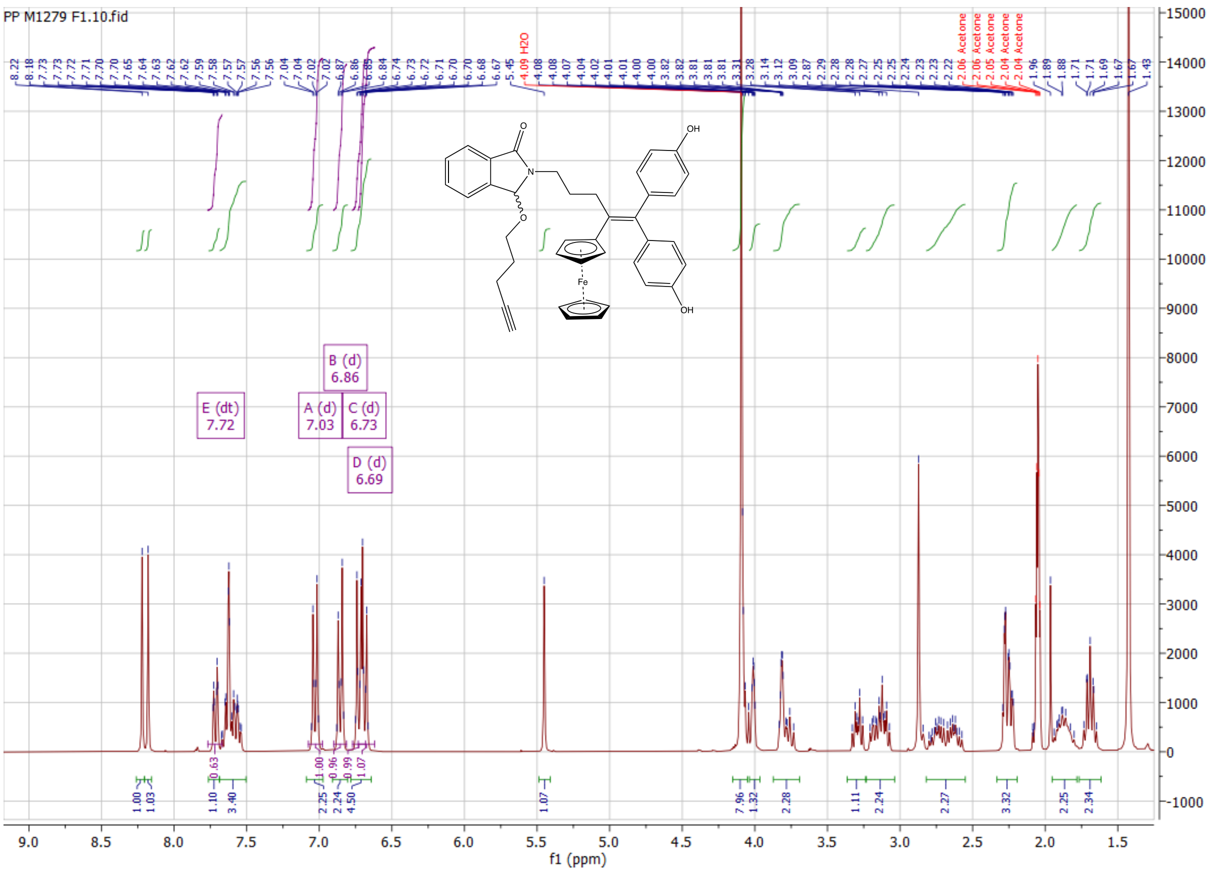

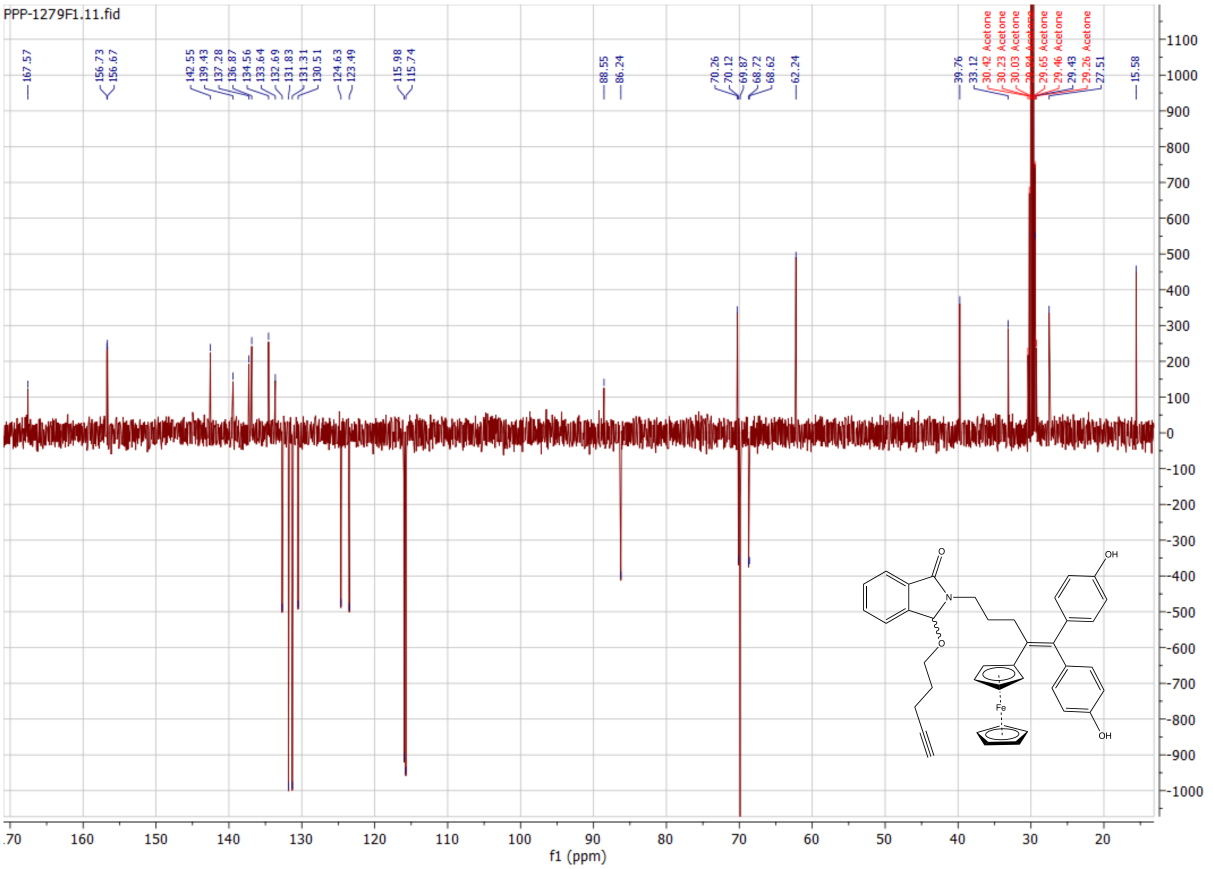

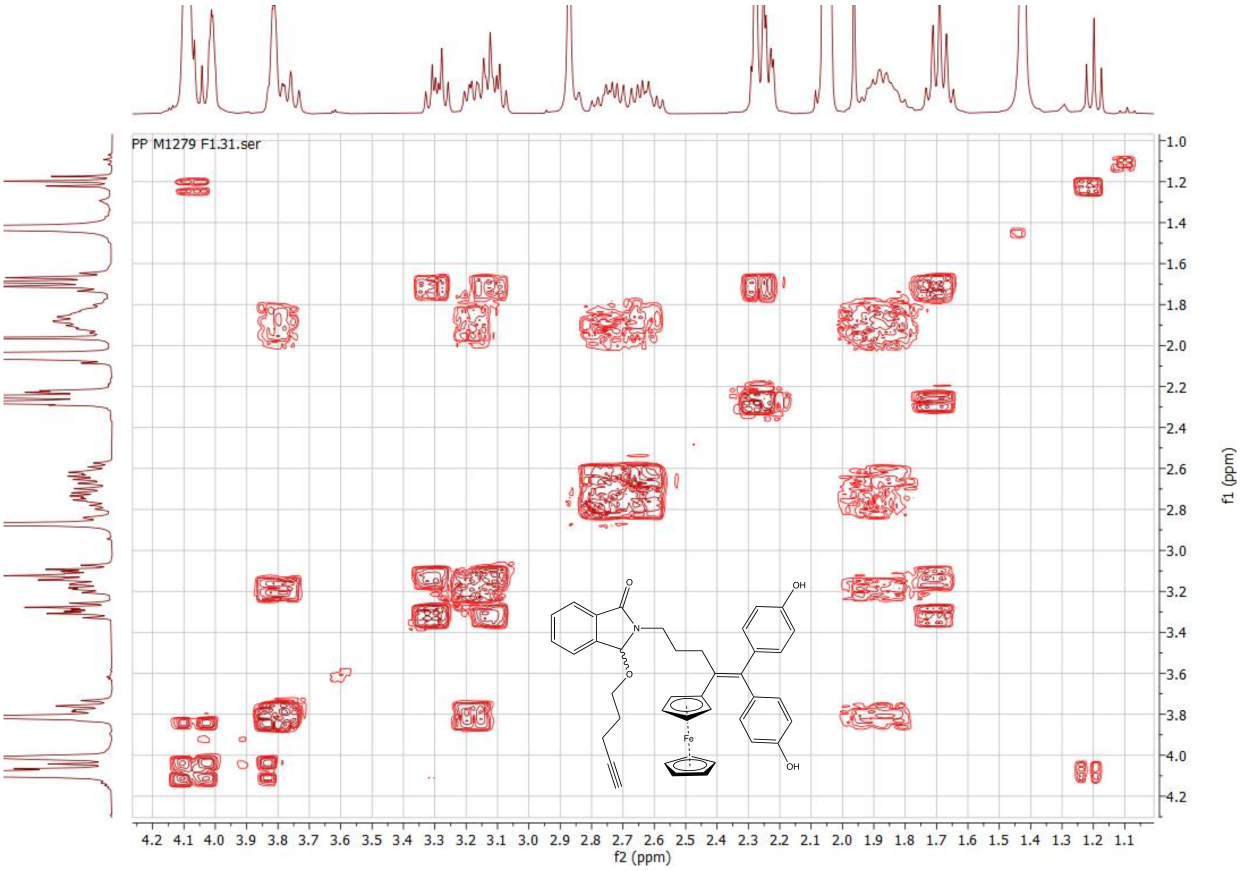

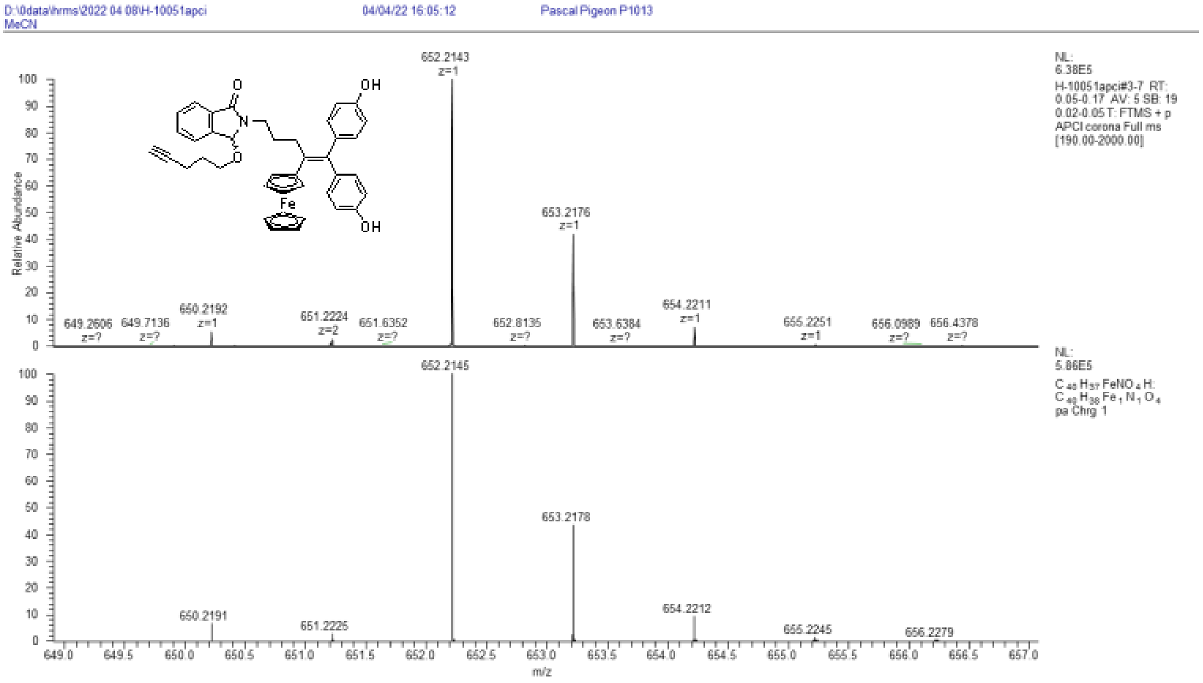

Experimental/theoretical isotopic pattern MS spectrum

Error = -0.3 ppm; Relative Intensity (%) 100

HRMS (APCI) m/z: [M+H]+ Calcd for C40H37FeNO4H 652.2145. Found 652.2143; (Error: -0.3 ppm).

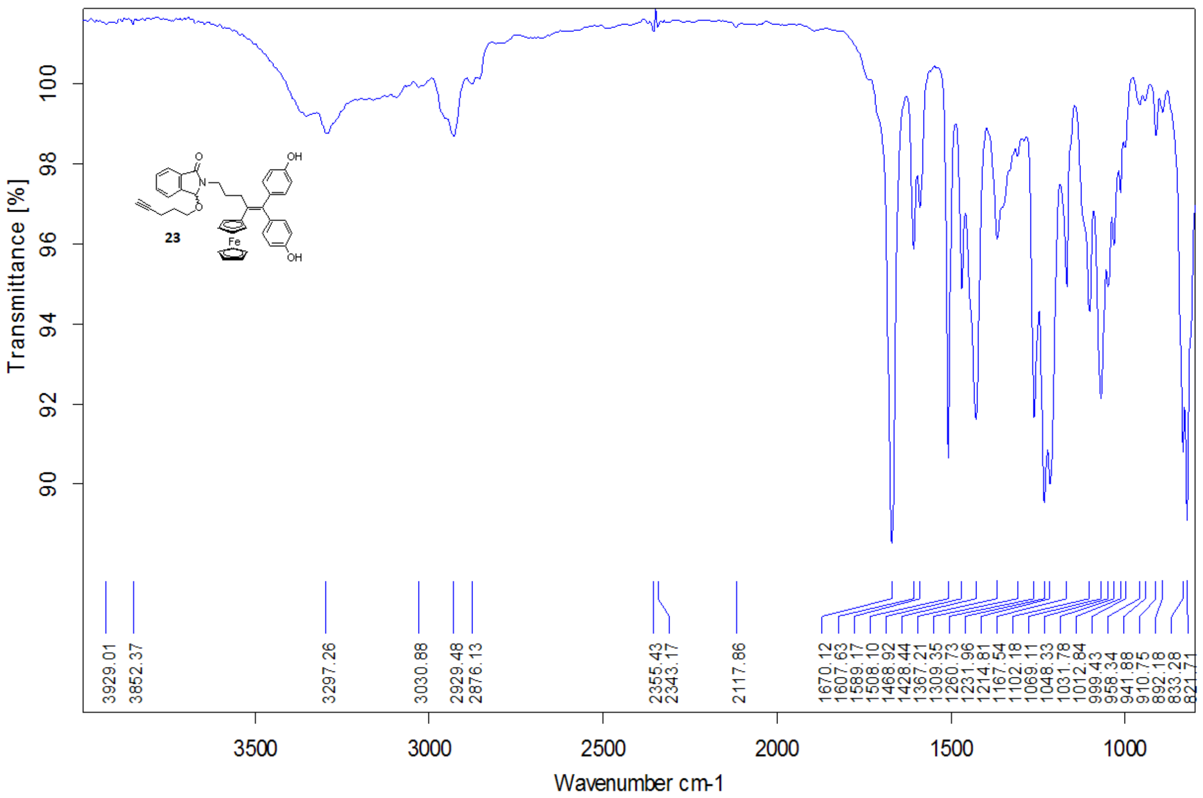

Transmittance [%]
Wavenumber cm-1
23
OH
Fe
3929.01
3852.37
3297.26
3030.88
2929.48
2876.13
2355.43
2343.17
2117.86
1670.12
1607.63
1589.17
1508.10
1468.92
1428.44
1367.21
1309.35
1260.73
1231.96
1214.81
1167.54
1102.18
1069.11
1048.33
1031.78
1012.84
999.43
958.34
941.88
910.75
892.18
833.28
821.71

Figure S46: ^{1}H (in $CDCl_3$), ^{13}C (in $CDCl_3$), COSY (in $CDCl_3$), HMQC (in $CDCl_3$) NMR, HR-MS and IR data for compound **24**

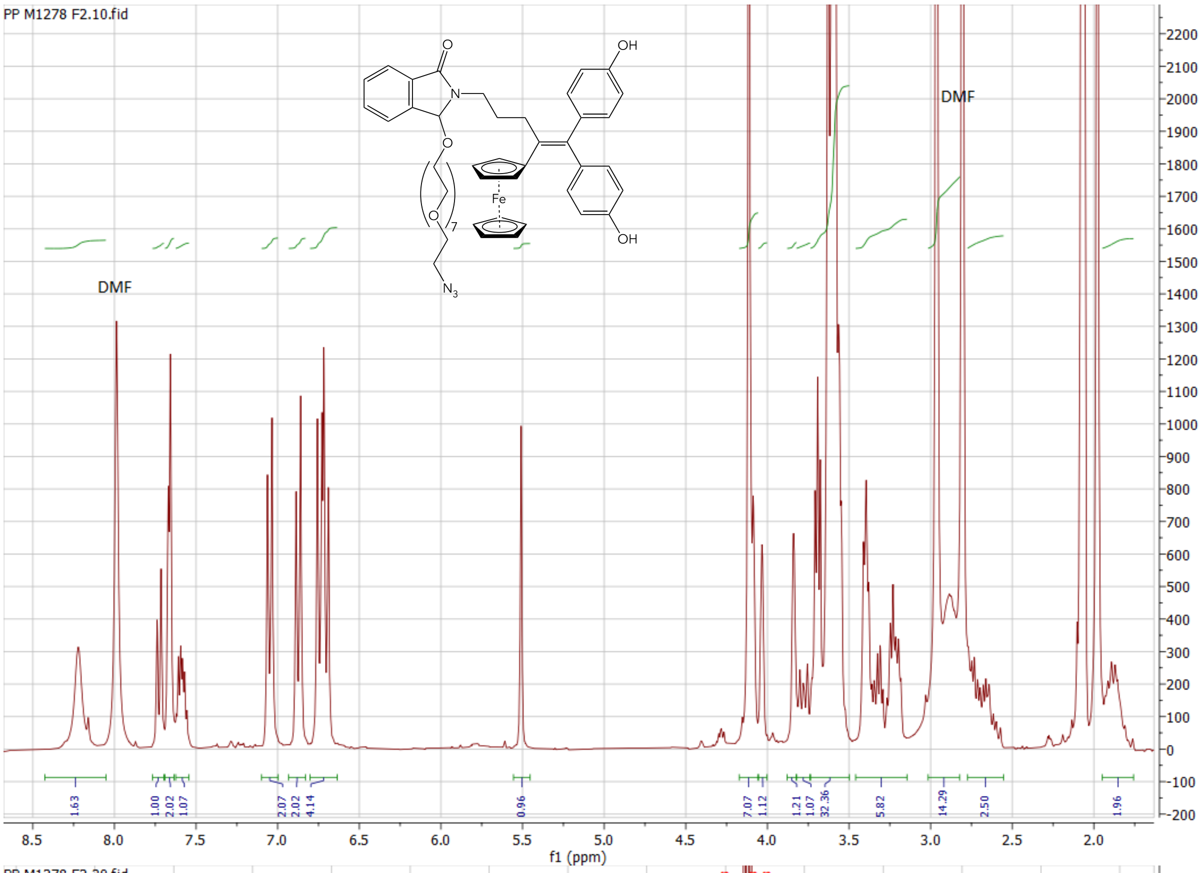

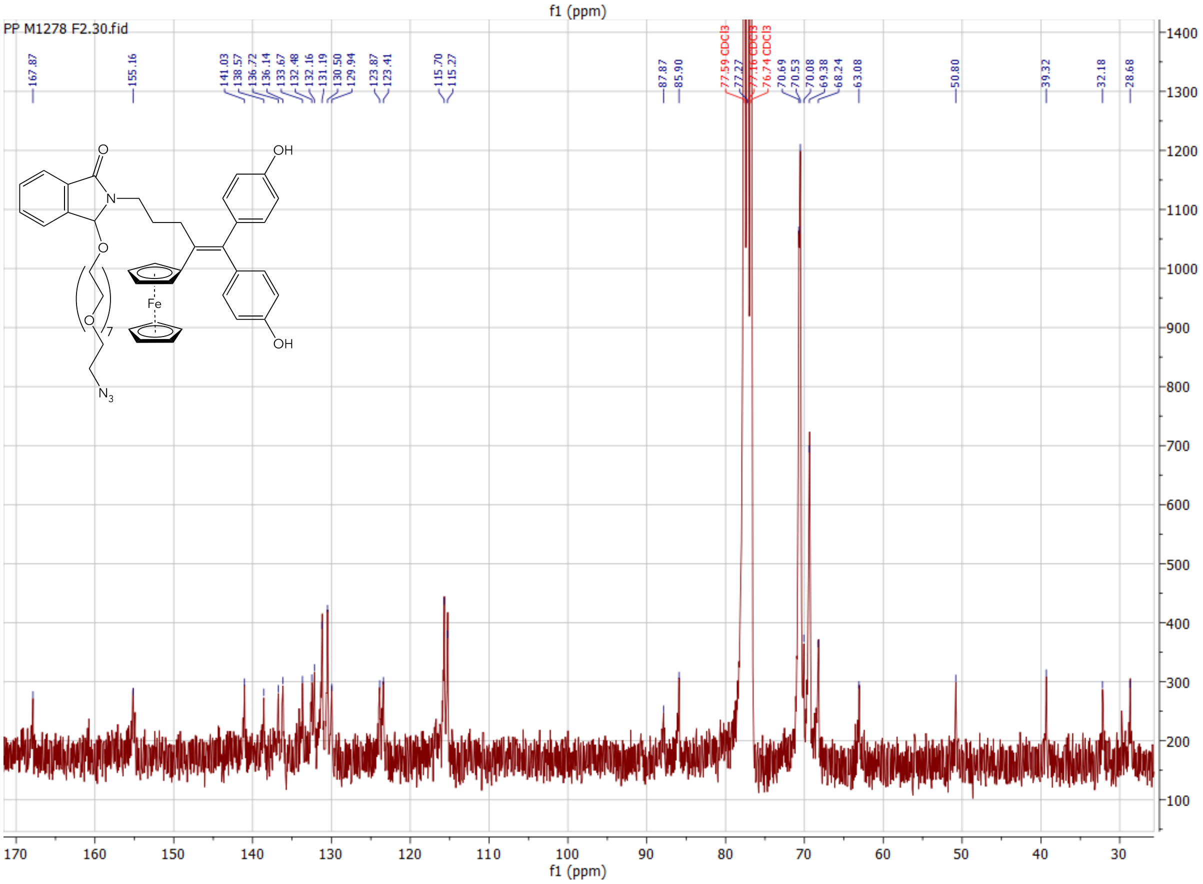

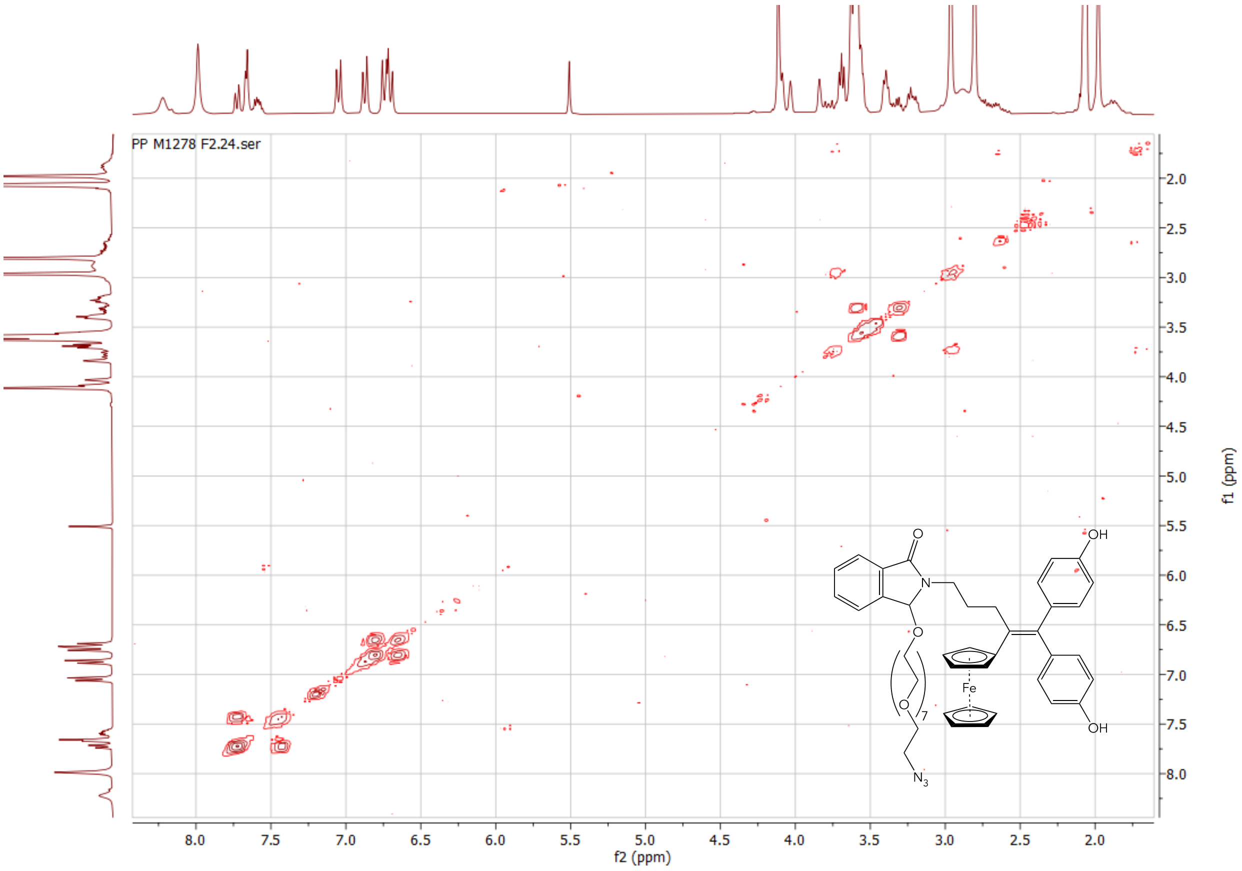
PP M1278 F2.24.ser
f2 (ppm)
f1 (ppm)
OH
OH
O
N
O
Fe
7
N3

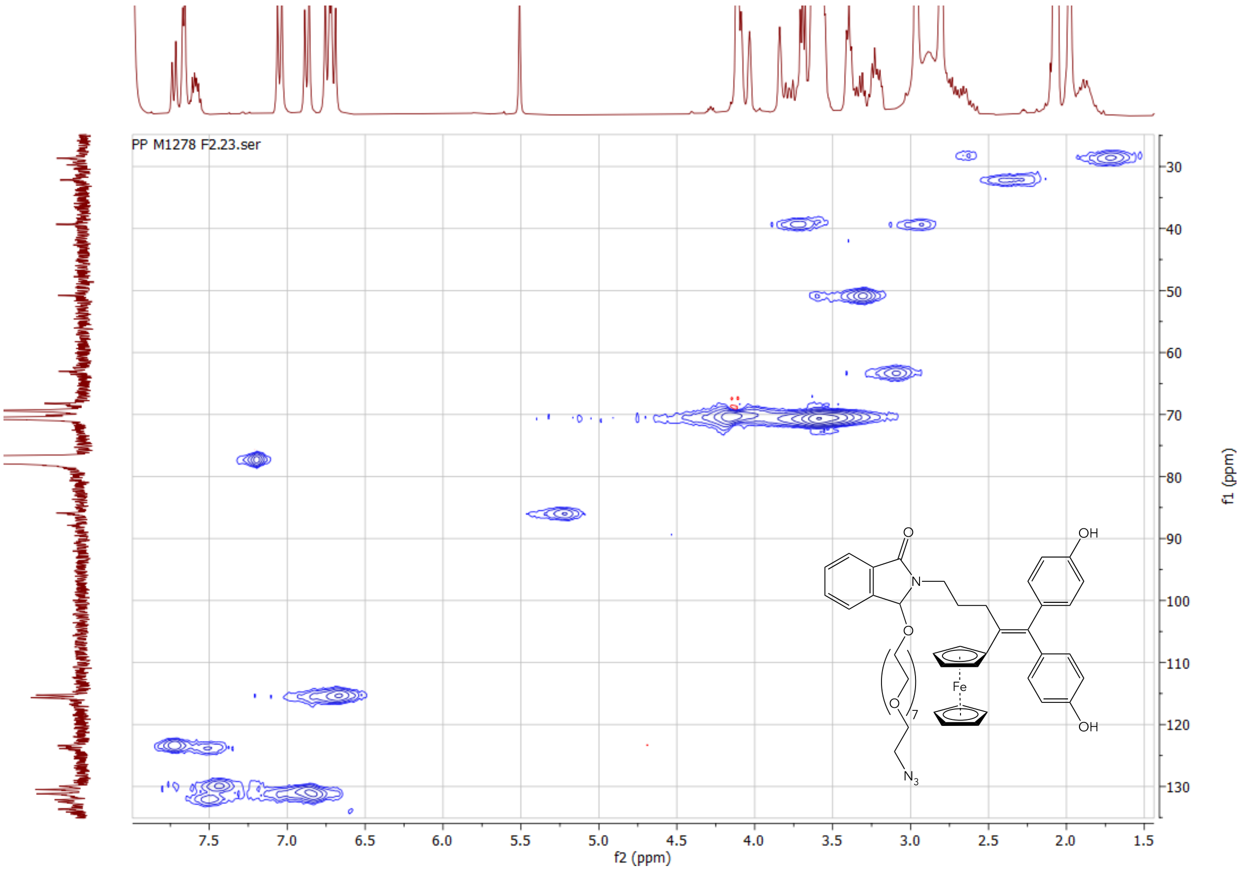
PP M1278 F2.23.ser
f2 (ppm)
f1 (ppm)
OH
OH
O
N
O
Fe
7
N3

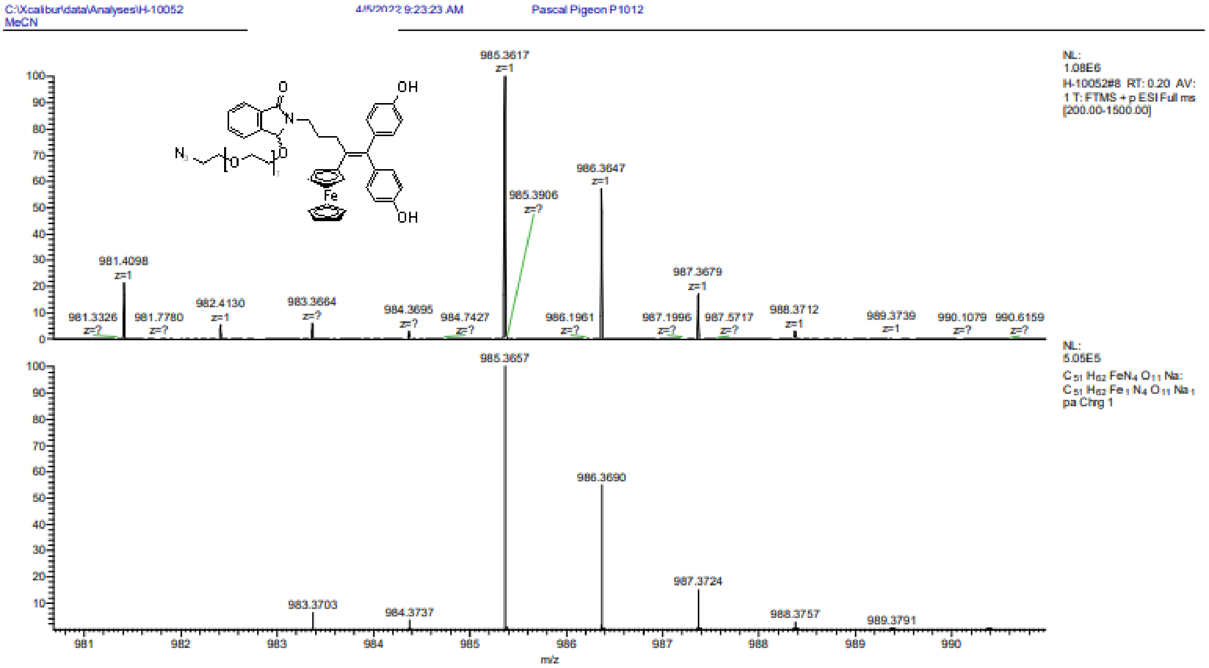

Experimental/theoretical isotopic pattern MS spectrum

Error = -4.0 ppm; Relative Intensity (%) 100

HRMS m/z: [M+Na]+ Calcd for C51H62FeN4O11Na 983.3703 Found 983.3664 ; (Error: -4.0 ppm).

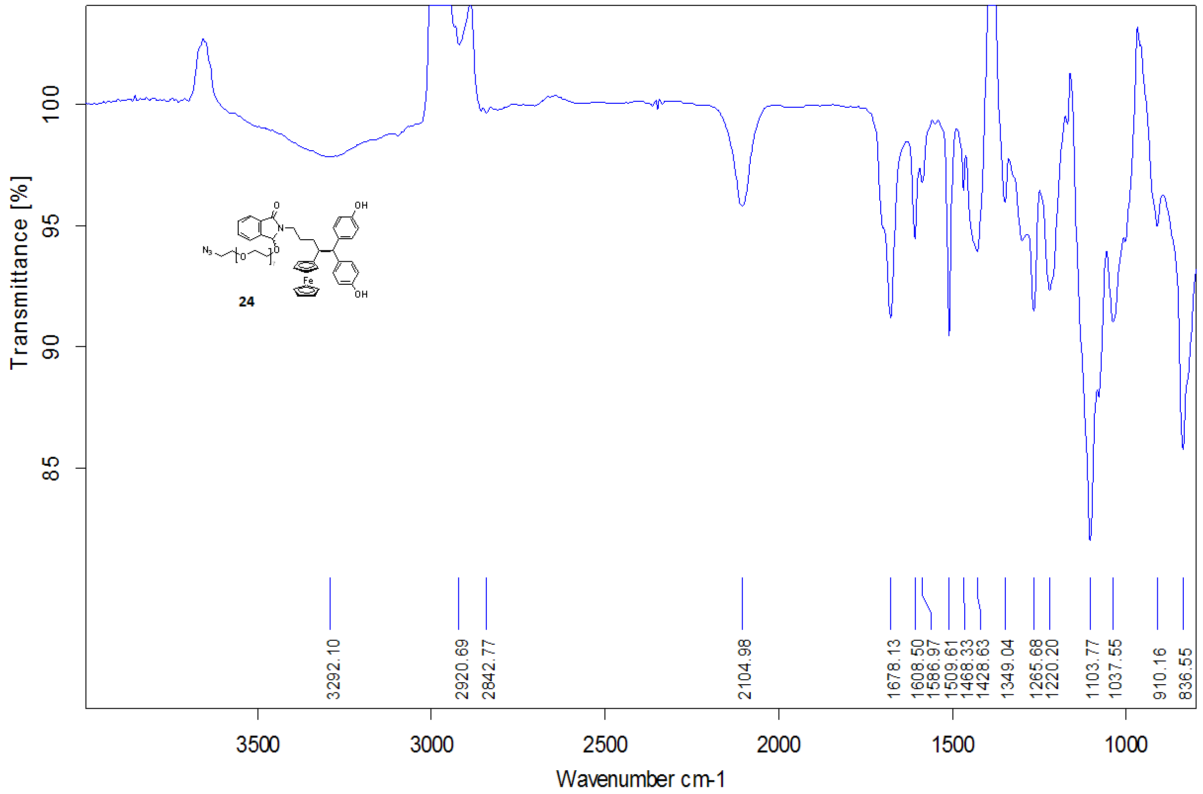

Figure S47: ^{1}H (in acetone-d_6), ^{13}C (in acetone-d_6), COSY (in acetone-d_6) NMR, HR-MS and IR data for compound **25**

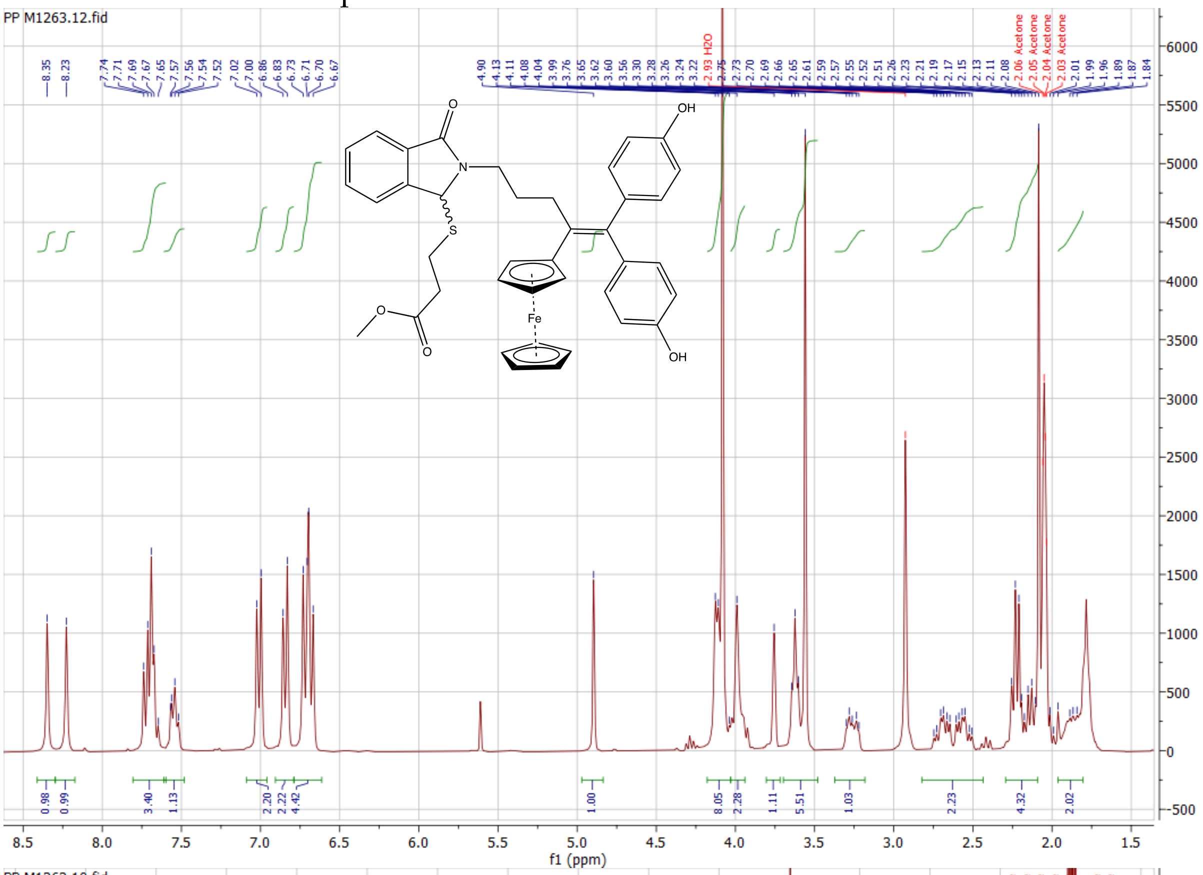

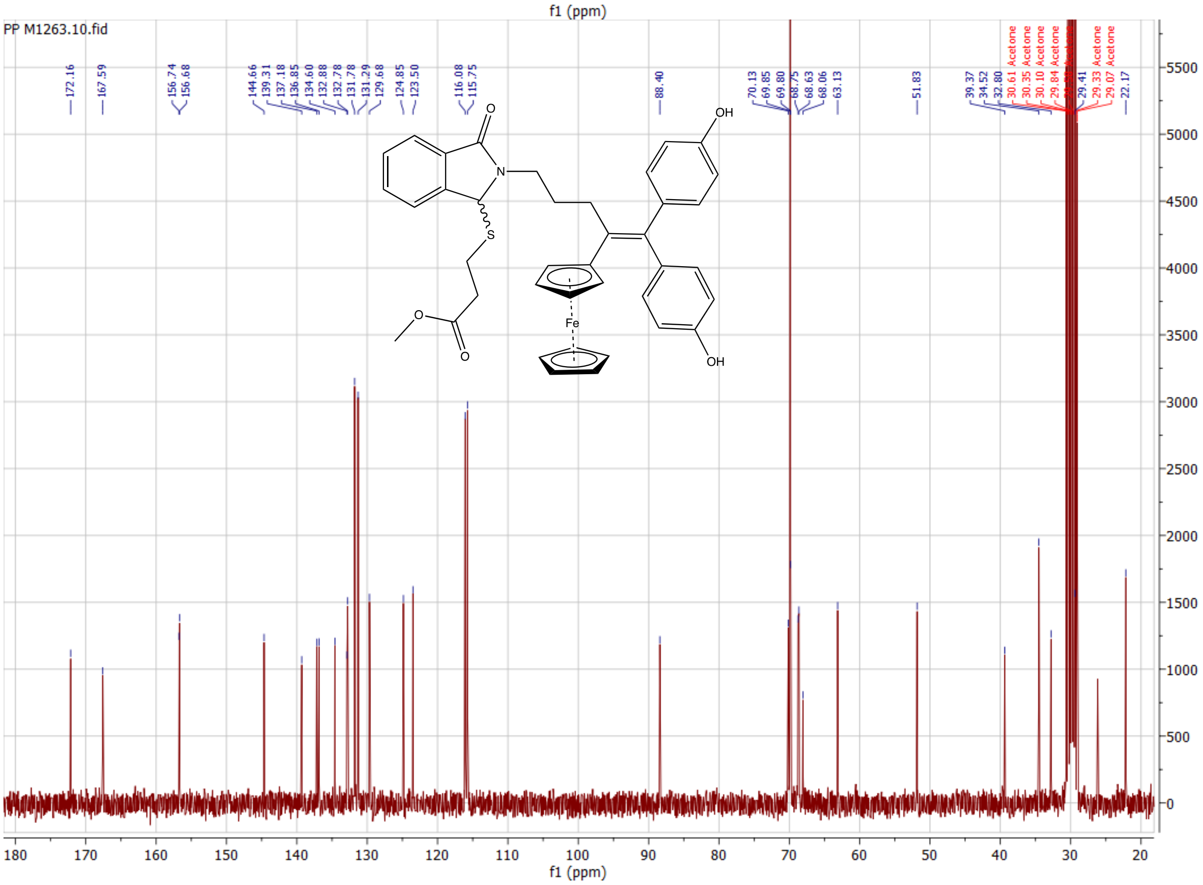

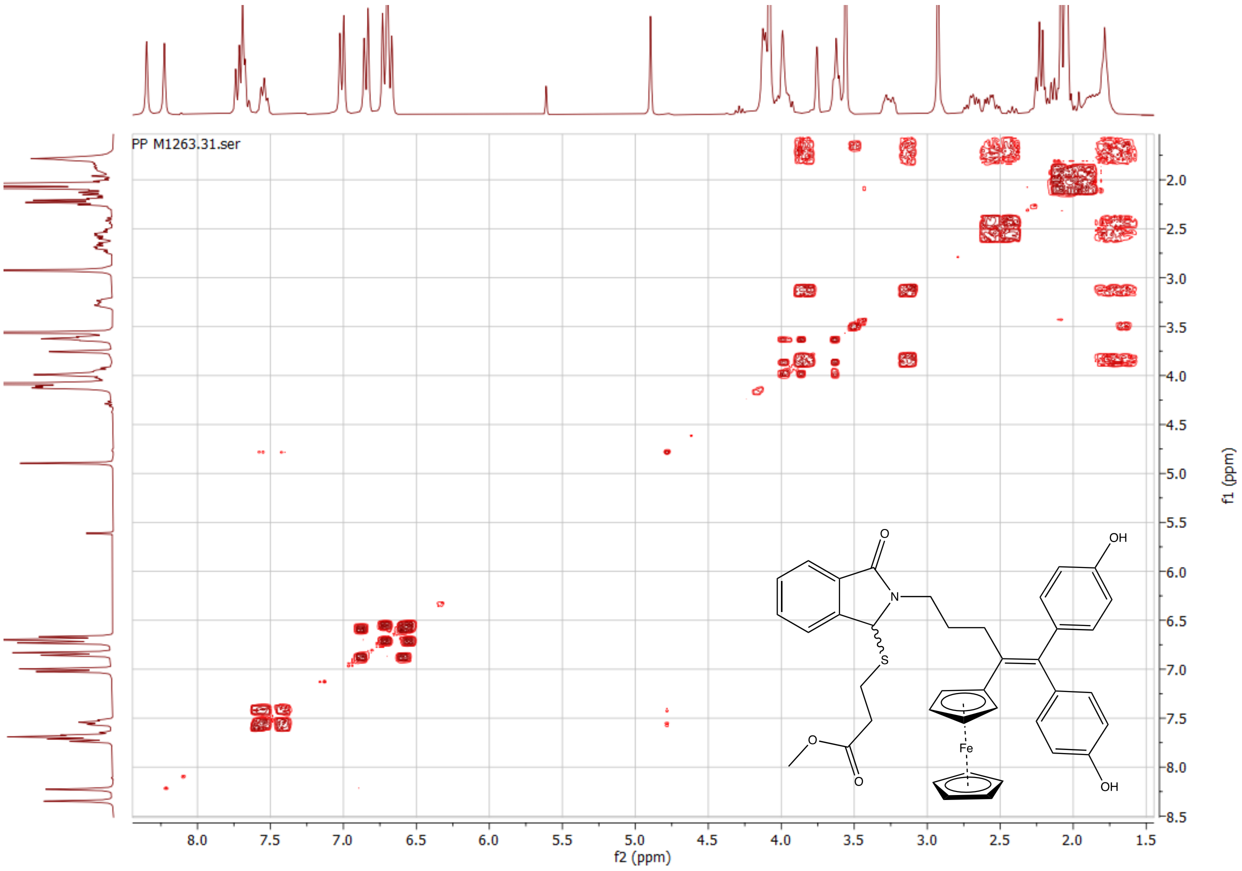

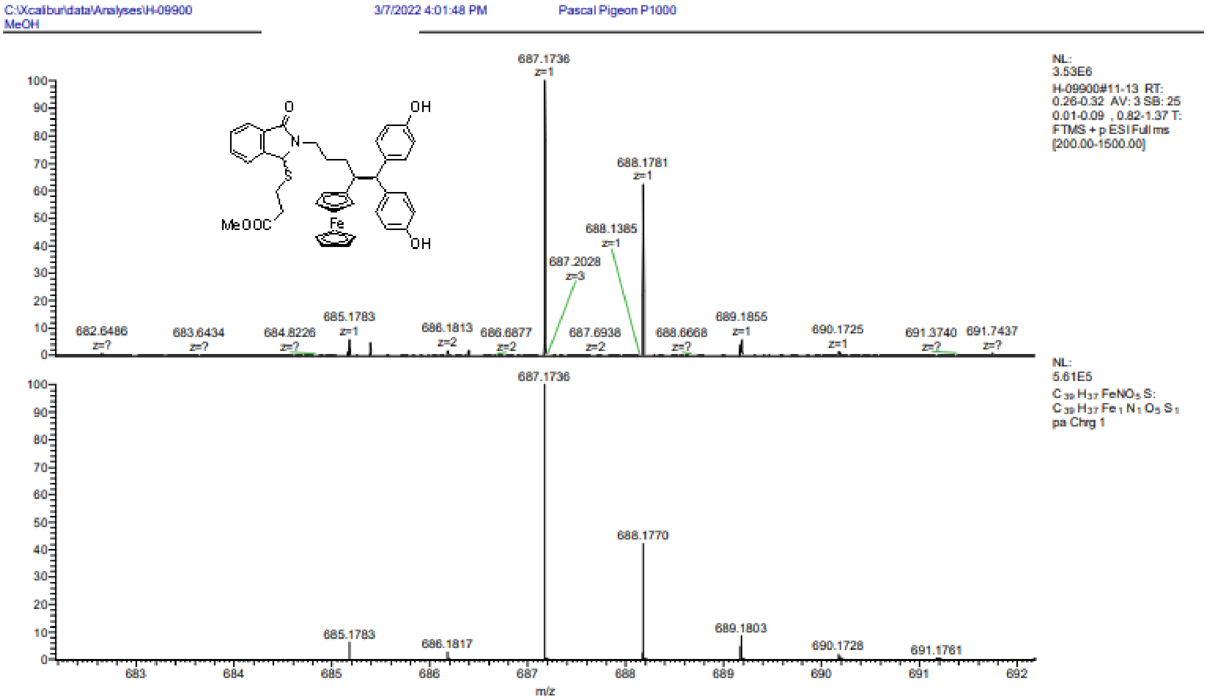

Experimental/theoretical isotopic pattern MS spectrum

Error = 0.0 ppm; Relative Intensity (%) 100

HRMS (ESI) m/z: [M]+ Calcd for C39H37FeNO5S 687.1736 . Found 687.1736; (Error: 0.0 ppm).

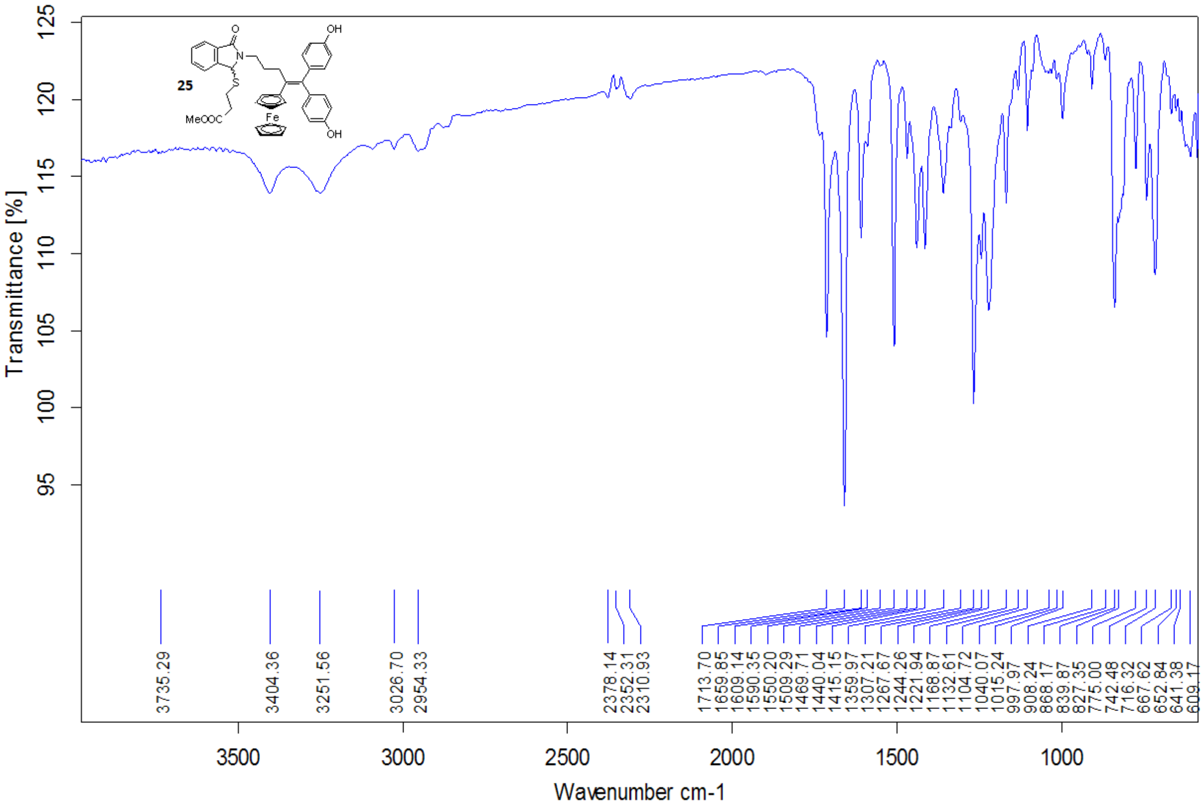

25
MeOOC
Fe
OH
Transmittance [%]
Wavenumber cm-1
3735.29
3404.36
3251.56
3026.70
2954.33
2378.14
2352.31
2310.93
1713.70
1659.85
1609.14
1590.35
1550.20
1509.29
1469.71
1440.04
1415.15
1359.97
1307.21
1267.67
1244.26
1221.94
1168.87
1132.61
1104.72
1040.07
1015.24
997.97
908.24
868.17
839.87
827.35
775.00
742.48
716.32
667.62
652.84
641.38
609.17

Figure S48: COSY (in DMSO-d6), ^{1}H (in DMSO-d6), ^{13}C (in DMSO-d6) NMR, HR-MS and IR data for compound **26**

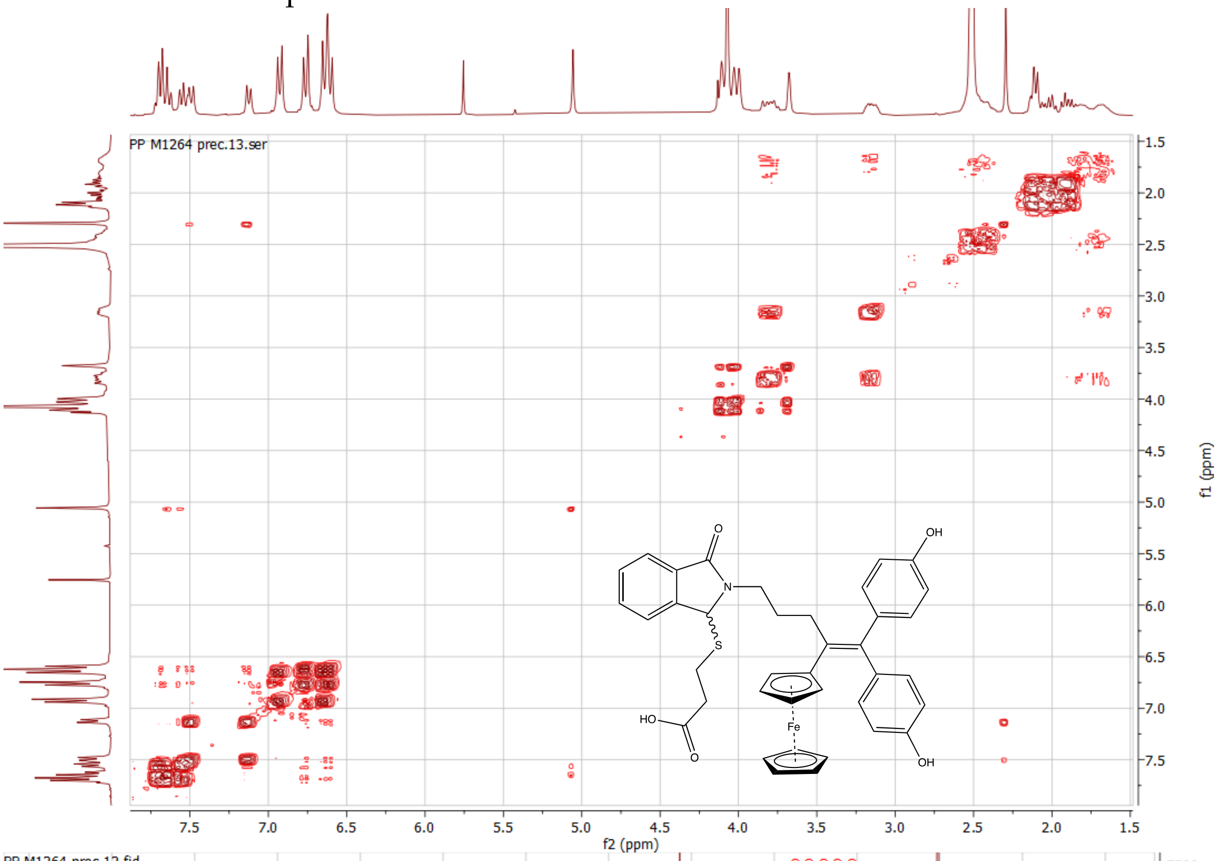

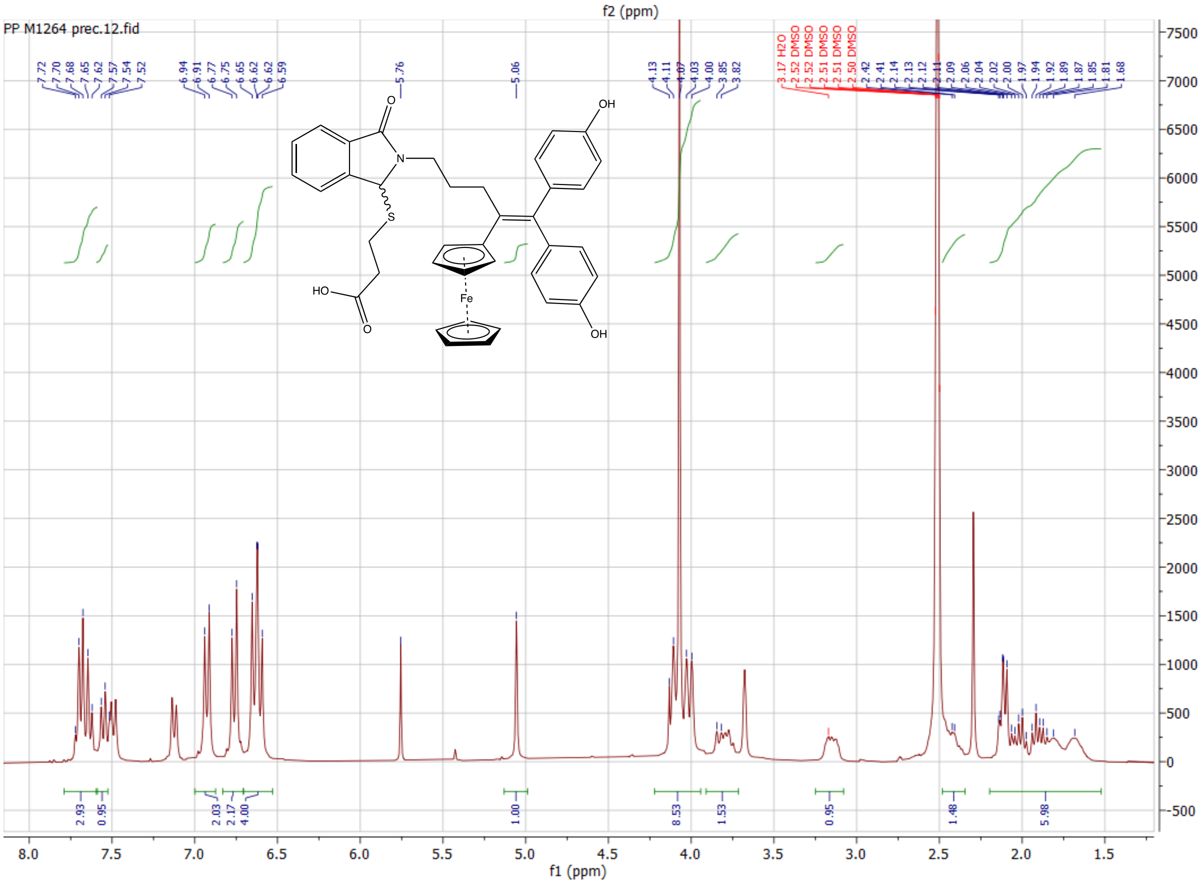

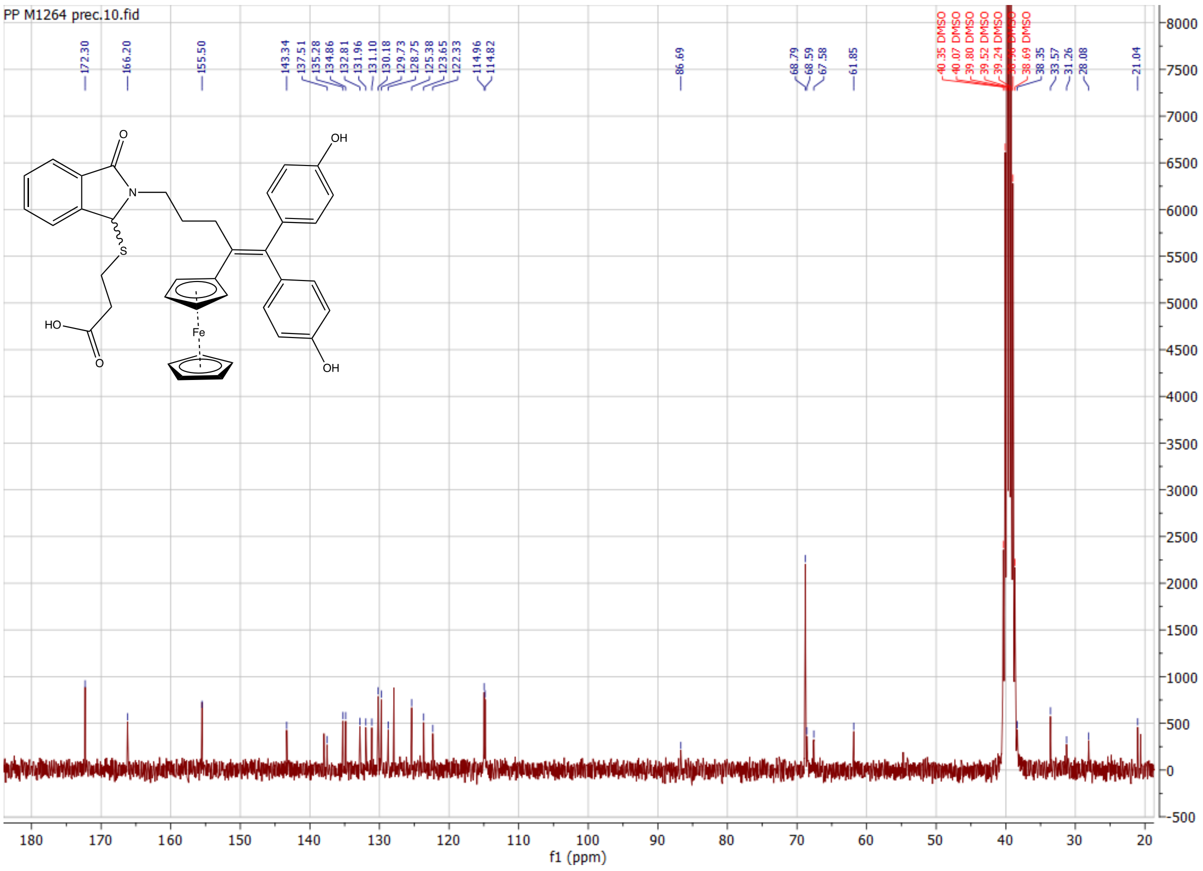

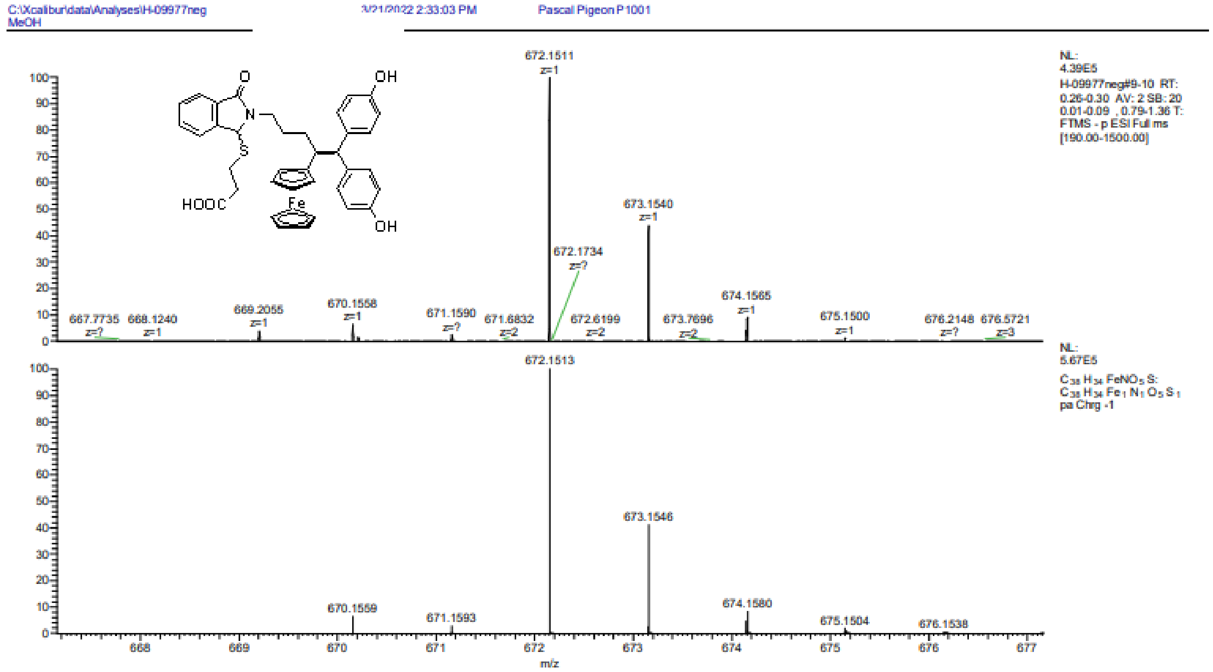

Experimental/theoretical isotopic pattern MS spectrum

Error = -0.2 ppm; Relative Intensity (%) 100

HRMS (ESI) m/z: [M] 1- Calcd for C38H34FeNO5S672.1513 . Found 672.1511; (Error: -0.2 ppm).

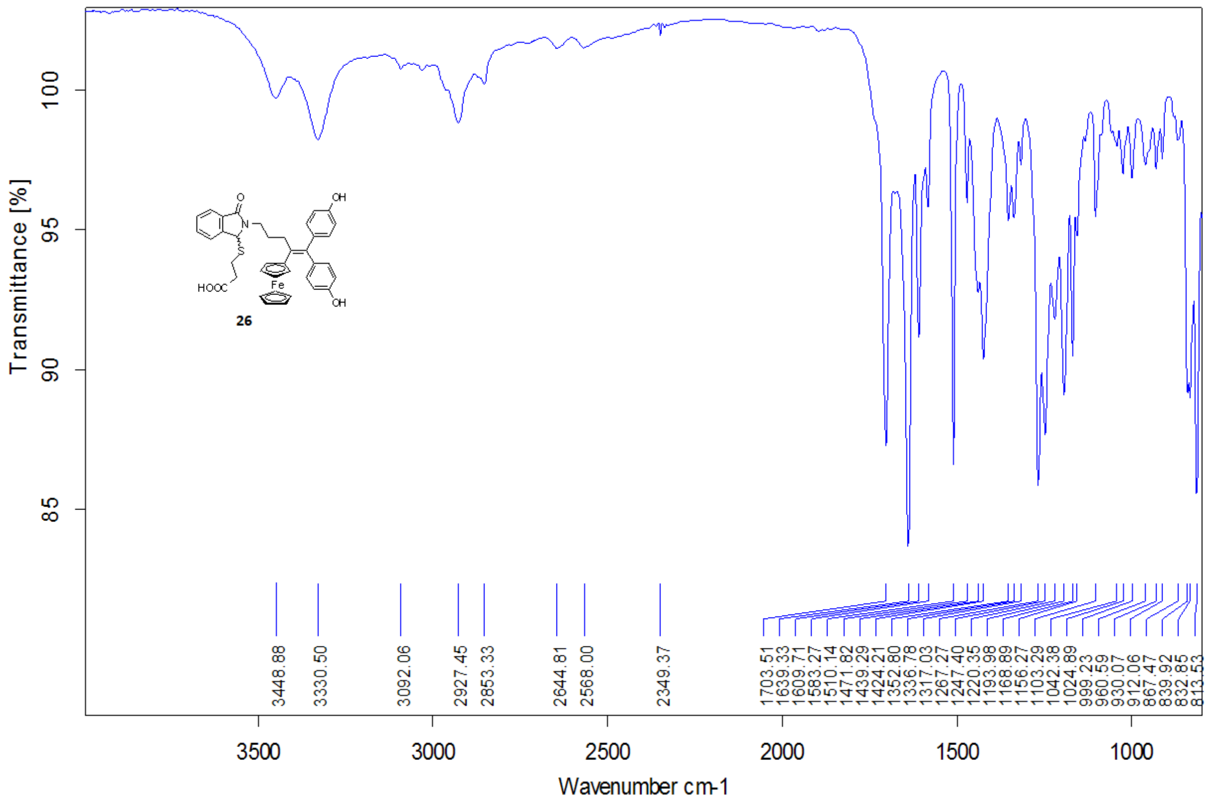

Transmittance [%]
Wavenumber cm-1
26
HOOC
Fe
OH
OH
N
O
S
3448.88
3330.50
3092.06
2927.45
2853.33
2644.81
2568.00
2349.37
1703.51
1639.33
1609.71
1583.27
1510.14
1471.82
1439.29
1424.21
1352.80
1336.78
1317.03
1267.27
1247.40
1220.35
1193.98
1168.89
1156.27
1103.29
1042.38
1024.89
999.23
960.59
930.07
912.06
867.47
839.92
832.85
813.53

Figure S49: ^{1}H (in acetone-d_6), ^{13}C (in acetone-d_6), COSY (in acetone-d_6), HMQC (in acetone-d_6) NMR, HR-MS and IR data for compound **27**

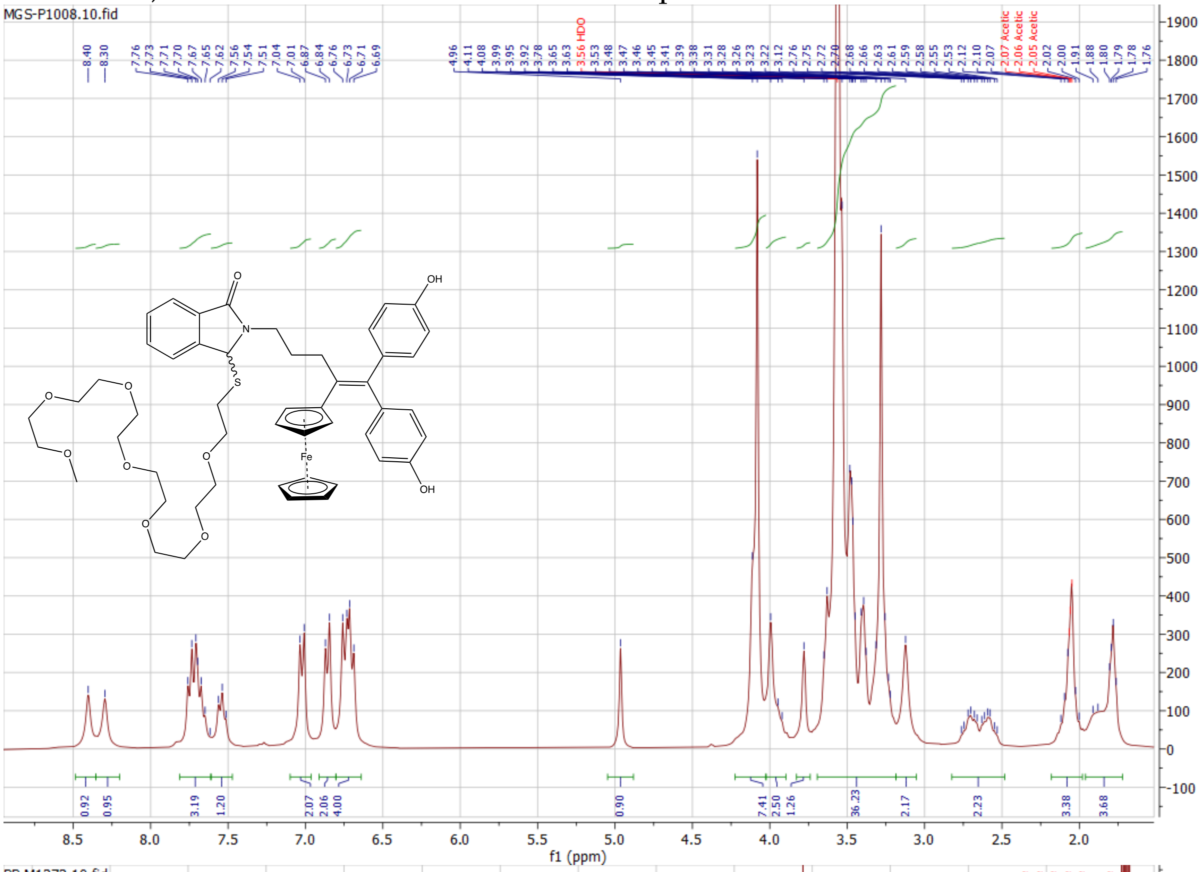

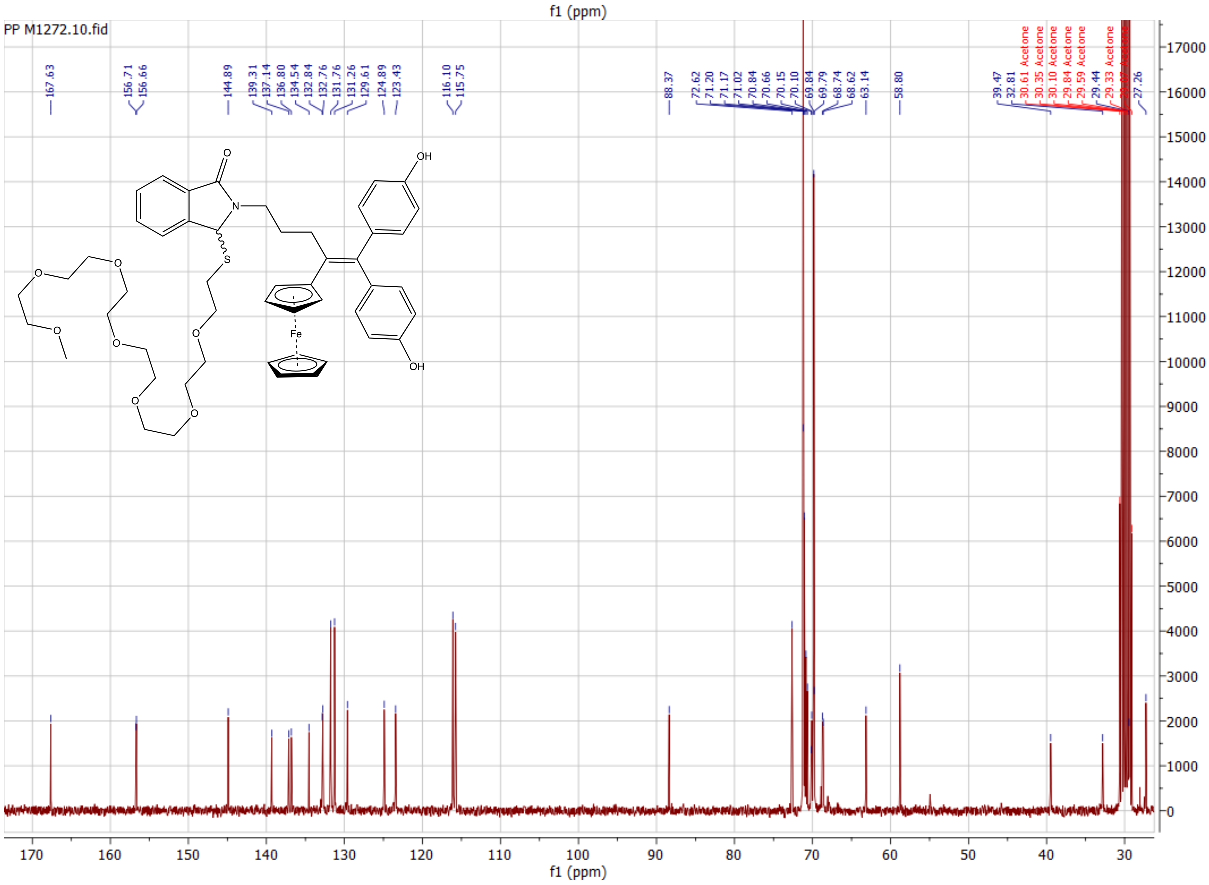

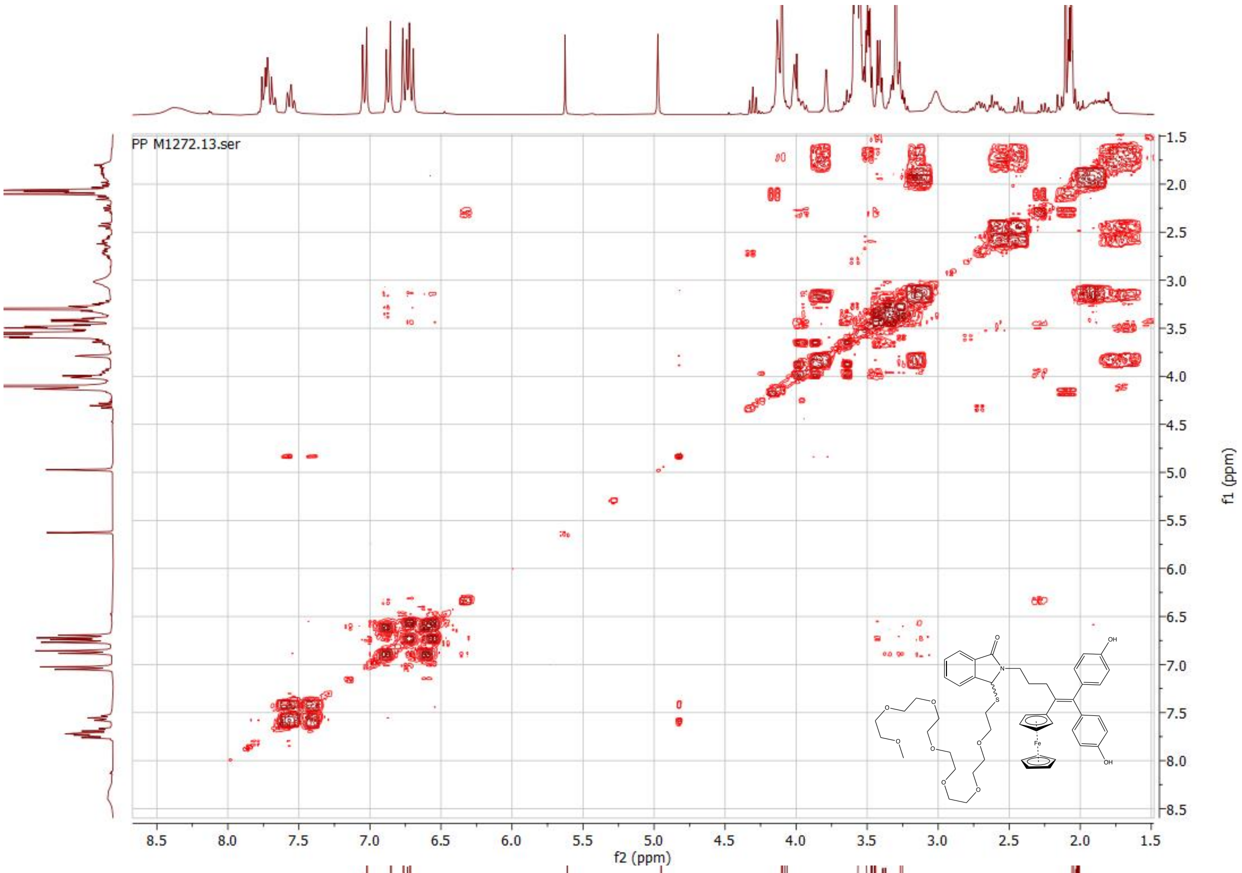
PP M1272.13.ser
f2 (ppm)
f1 (ppm)

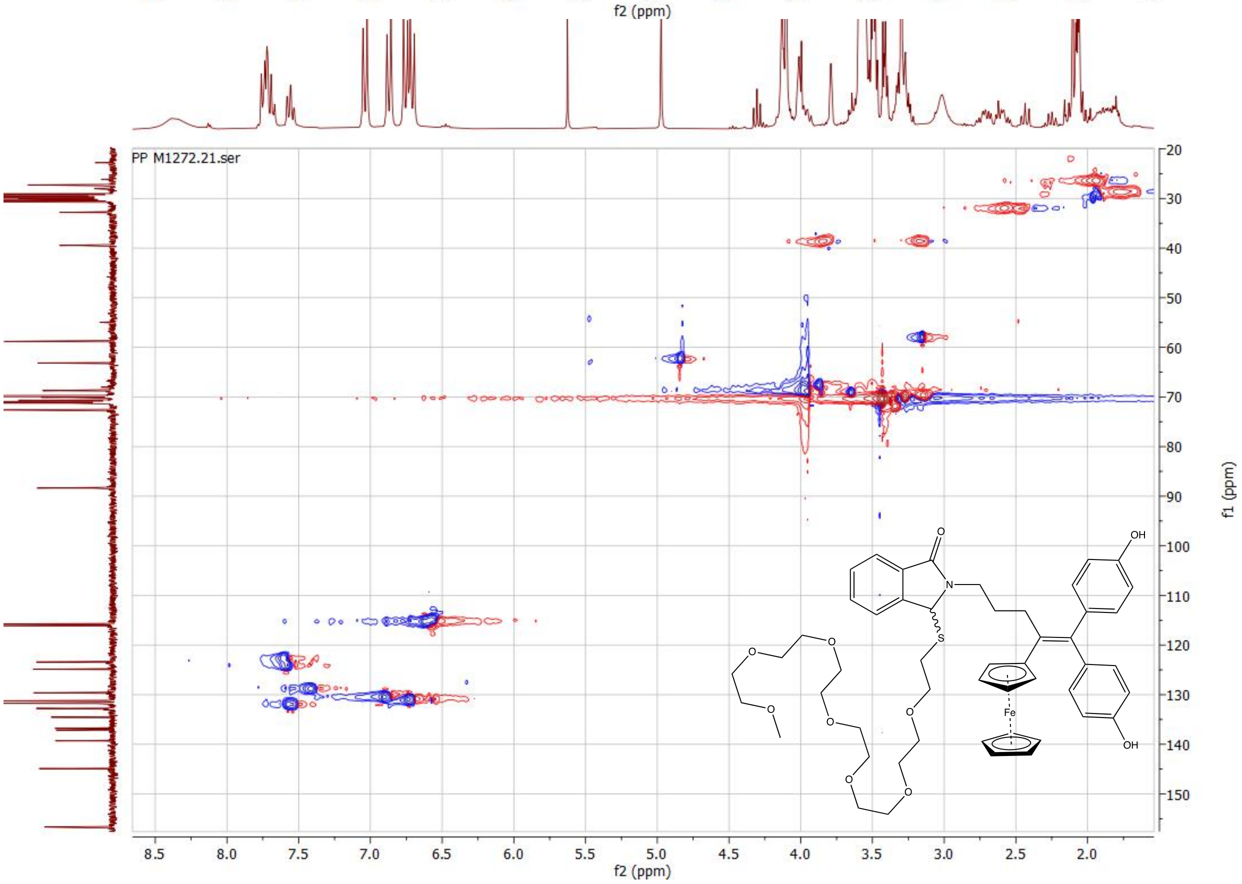
PP M1272.21.ser
f2 (ppm)
f1 (ppm)

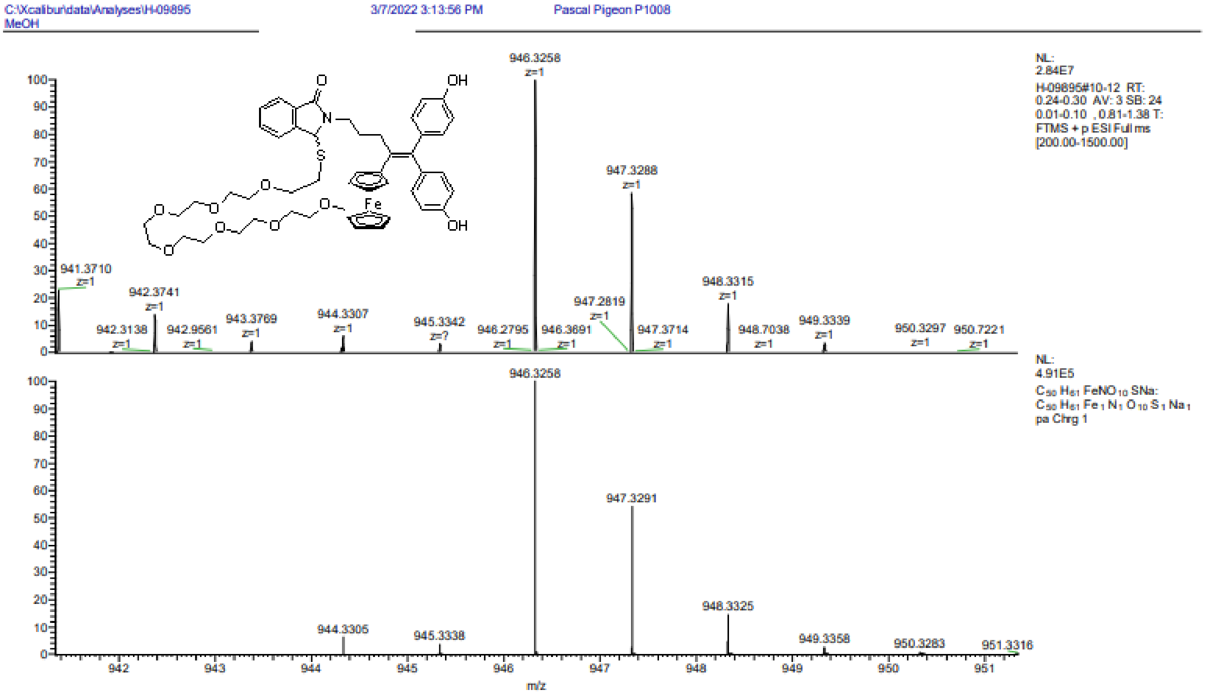

Experimental/theoretical isotopic pattern MS spectrum

Error = 0.0 ppm; Relative Intensity (%) 100

HRMS (ESI) m/z: [M+Na]+ Calcd for C50H61FeNO10SNa 946.3258. Found 946.3258; (Error: 0.0 ppm).

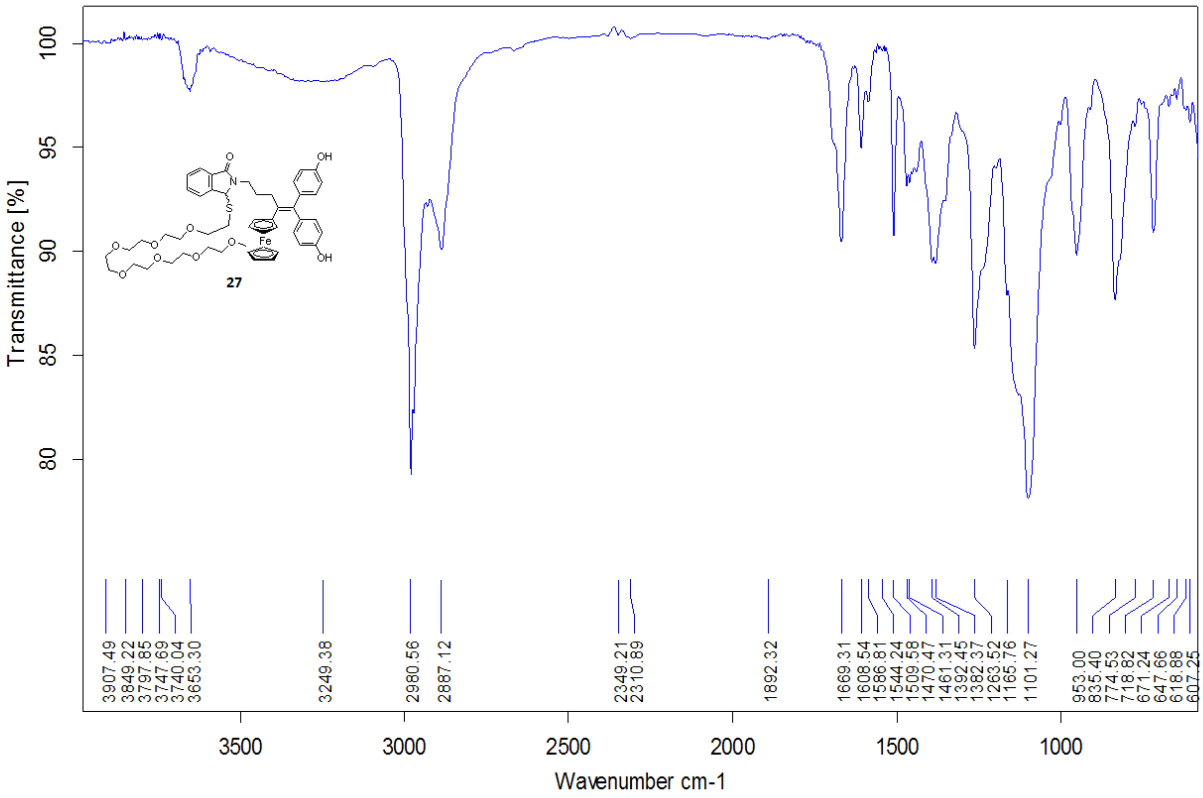

Table S1. Crystallographic data and structure refinement parameters for **15**, **23•acetone** and **25•acetone**.

**Parameters**	**15**	**23•0.5acetone**	**25•acetone**
Chemical formula	$C_{16}H_{17}FeNO$	$C_{41.5}H_{40}FeNO_{4.5}$	$C_{42}H_{43}FeNO_6S$
Formula weight (g.mol^{-1})	295.15	680.59	745.68
Crystal system	monoclinic	monoclinic	triclinic
Space group	$P2_1/c$	$P2_1/n$	P-1
Crystal size (mm^3)	0.37 x 0.27 x 0.06	0.2 x 0.1 x 0.05	0.3 x 0.2 x 0.1
Crystal color and shape	Yellow prism	Orange prism	Orange plate
a (Å)	14.6466(7)	9.8118(3)	10.7220(5)
b (Å)	7.1923(3)	17.2893(6)	13.2436(6)
c (Å)	12.0545(6)	20.2760(6)	15.3329(6)
α (Å)	90	90	97.196(3)
β (Å)	94.662(4)	91.557(2)	103.916(4)
γ (Å)	90	90	113.797(4)
Cell volume (Å^3)	1265.65(10)	3438.33(19)	1872.10(16)
T (k)	200(1)	200(1)	200
Wavelength	1.54178	1.54178	0.71073
Z	4	4	2
ϱ_{calcd} (g.cm-^{3})	1.549	1.315	1.323
μ (mm^{-1})	9.458	3.874	0.507
Scan rage (°)	$6.063 < \theta < 66.571$	$4.37 < \theta < 66.47$	$1.9360 < \theta < 32.2830$
Index ranges	$-17 \leq h \leq 16$	$-11 \leq h \leq 10$	$-14 \leq h \leq 14$
	$-5 \leq k \leq 8$	$-20 \leq k \leq 20$	$-17 \leq k \leq 17$
	$-14 \leq l \leq 14$	$-24 \leq l \leq 21$	$-20 \leq l \leq 20$
Reflections (all / independent)	9310 / 2242	26640 / 6066	25312 / 9219
Completeness (%)	99.9	99.8	99.3
R_{int}	0.0471	0.0505	0.0892
Data / parameters / restraints	2242 / 173 / 0	6066 / 455 / 27	9219 / 471 / 0
Goodness-of-fit on F^2	1.025	1.031	1.102
R1 [I>2σ(I)] (%)	3.50	3.82	6.30
wR2 (all data) (%)	9.04	9.82	16.76
Largest difference peak and hole (Å^{-3})	0.247, -0.290	0.265, -0.341	1.517, -0.588

Figure S50: Solubility studies by RP-HPLC analysis of compounds **18** (A), **20** (B) and **22** (C), **24** (D) and **27** (E)

A given amount of compounds **18**, **20** and **22** was weighted and suspended in a given volume of tested solvent (DMSO, MeOH, PBS and H_2O) to reach a theoretical concentration of 1 mM. Suspensions were sonicated for 1 min and shaken at R.T. for 18 h. The resulting solutions were filtrated (pore size 0.2 μm) and diluted to 100 μM in MeCN (for the solutions in organic solvents). HPLC analysis was performed on Nucleodur C18 column, (100 Å, 5 μm, 4.6 x 150 mm, Macherey-Nagel) using MeCN/H_2O 65:35 at 1 mL/min with detection at 302 nm (**18**, **20** and **22**) or MeCN/H_2O 55:45 at 1 mL/min with detection at 254 nm (**24** and **27**).

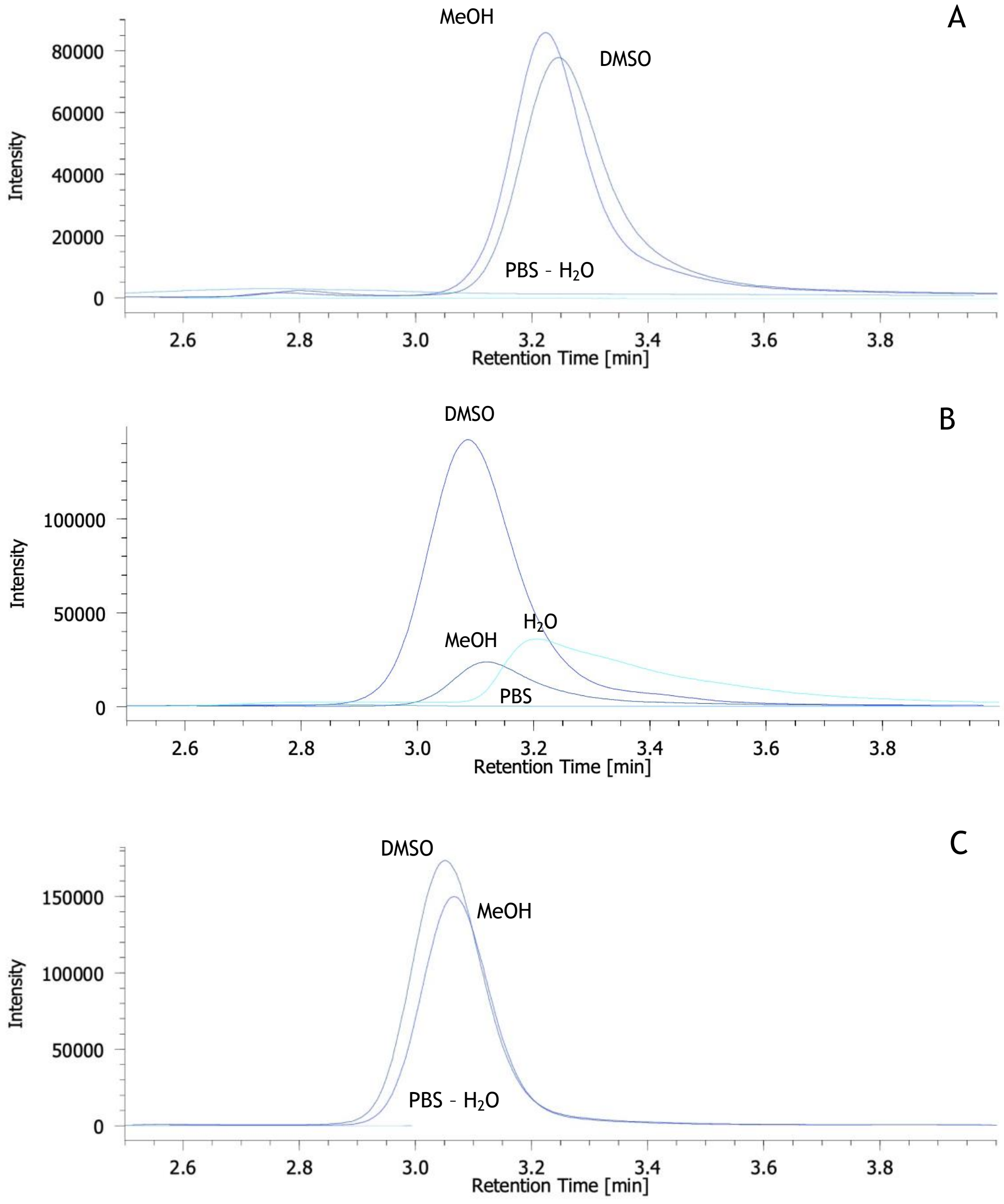

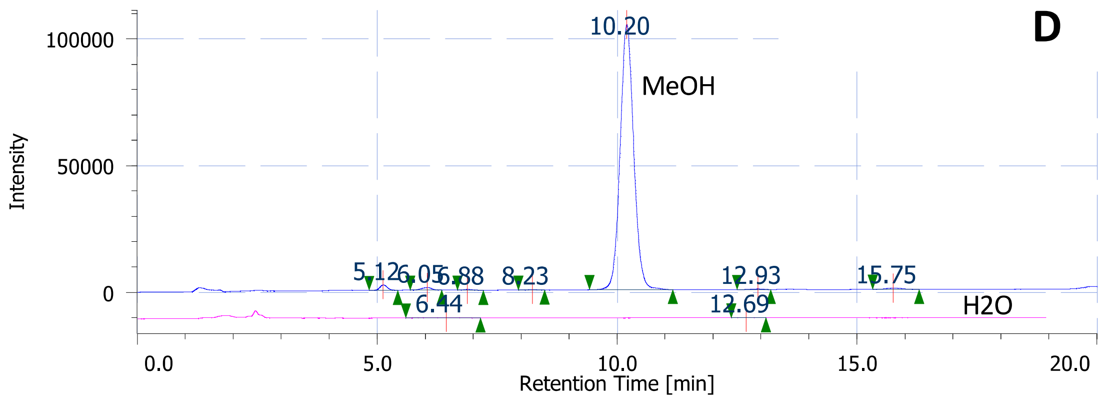
D
10.20
MeOH
H2O
5.12
6.05
6.88
8.23
6.44
12.93
12.69
15.75
Intensity
100000
50000
0
0.0
5.0
10.0
15.0
20.0
Retention Time [min]

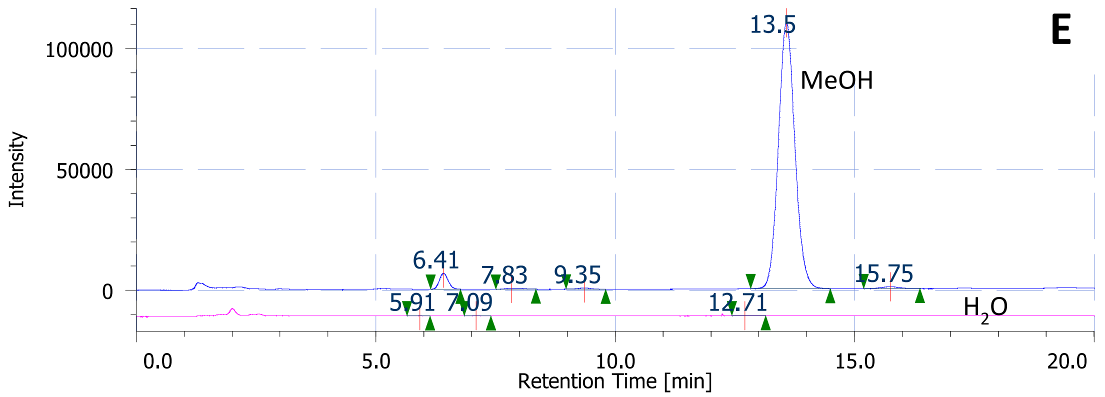
E
13.5
MeOH
H_2O
6.41
7.83
9.35
15.75
5.91
7.09
12.71
Intensity
100000
50000
0
0.0
5.0
10.0
15.0
20.0
Retention Time [min]

Figure S51. UV-vis spectra of compounds **19**, **20, 22**, **23** and **25** (50 µM) treated by HRP (46 nM) and H_2O_2 (200 µM) in 0.2 M TRIS buffer pH 8.1. Conversion to the phenolate form of the quinone methides is assessed by the appearance of the characteristic bands at ca. 570 and 360 nm.

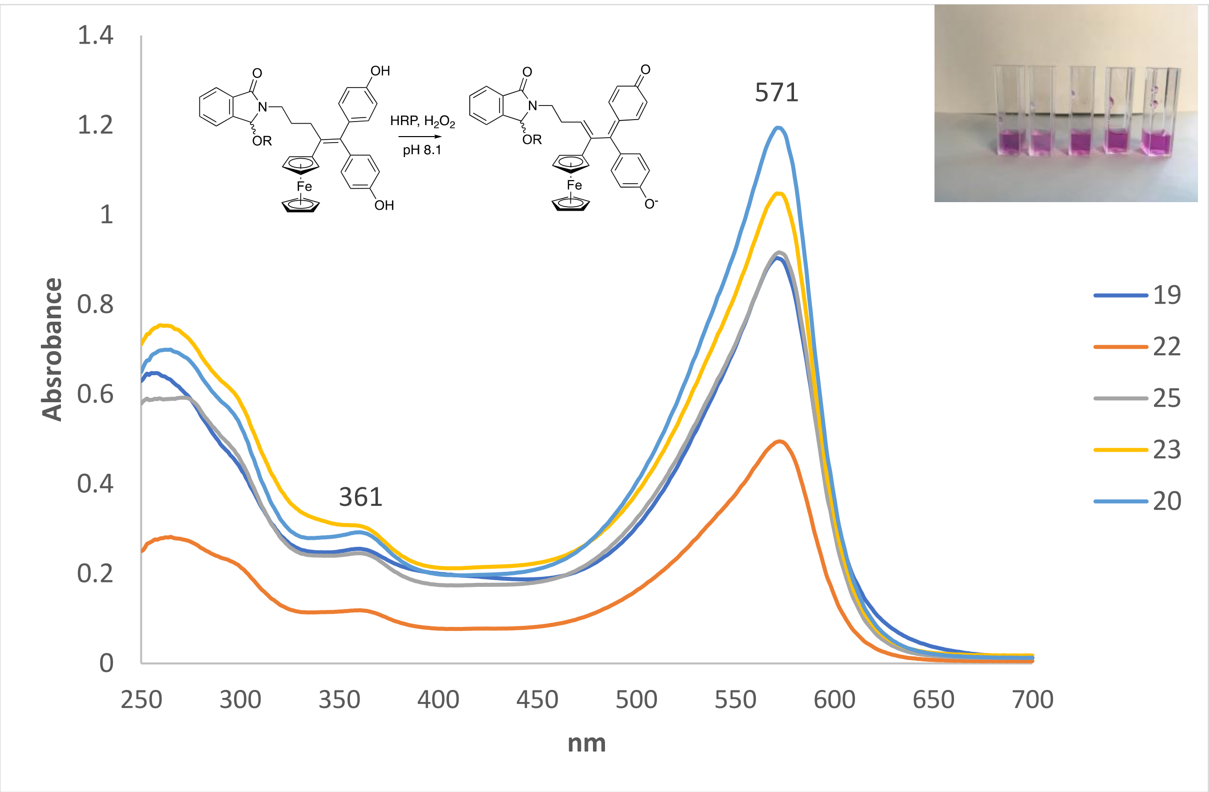

Figure S52: MTT cell viability assay for compounds **Fc-OH-TAM-3**, **3h**, **20**, **22**, **23** and **25**.

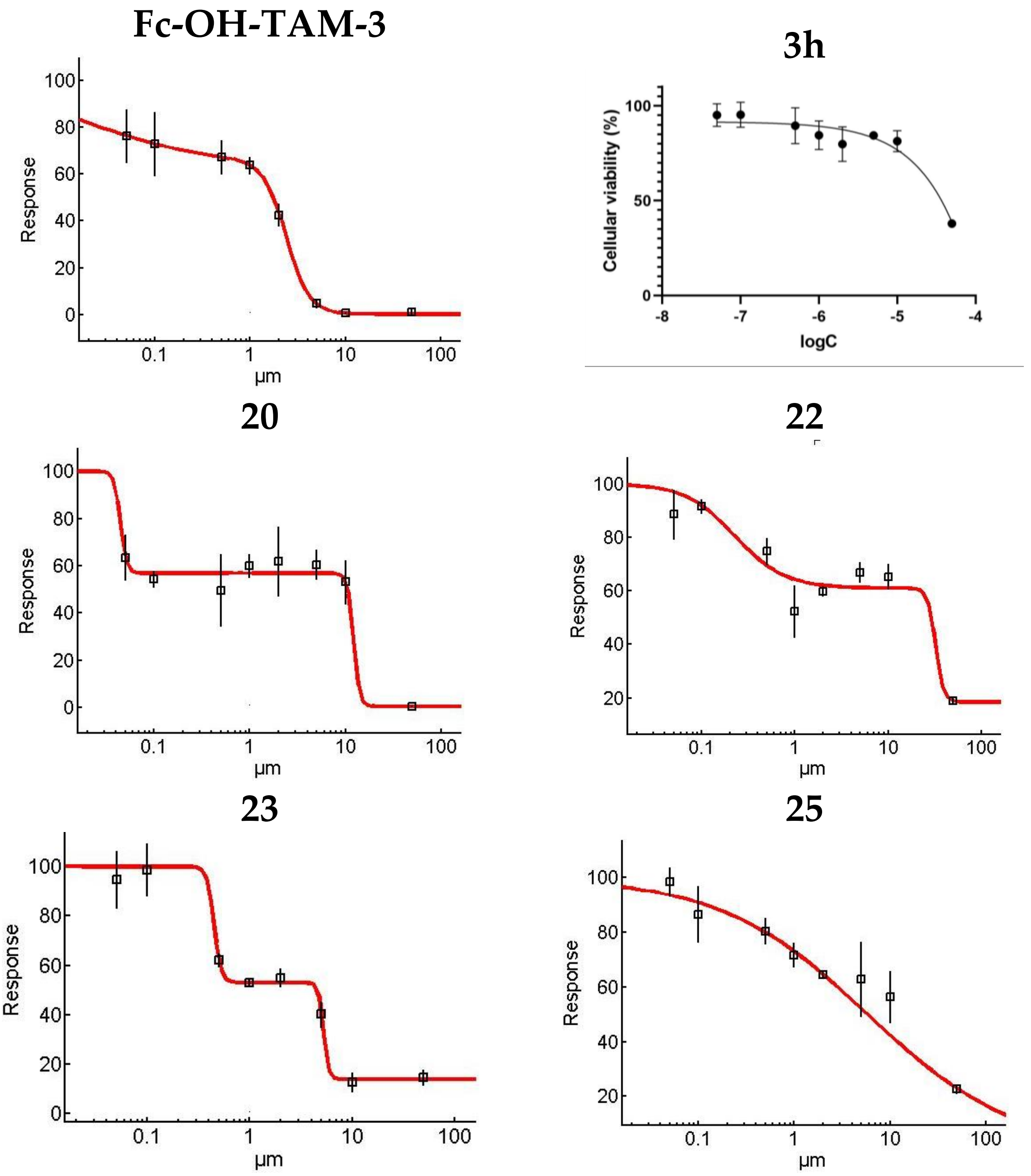

A: Fitting of dose-response curves for compounds **Fc-OH-TAM-3**, **3h**, **20**, **22**, **23**, **25** on MCF-7 cells using Dr-Fit Software (Di Veroli et al. Sci. Rep. 2015, 5, 14701) or GraphPad Prism.

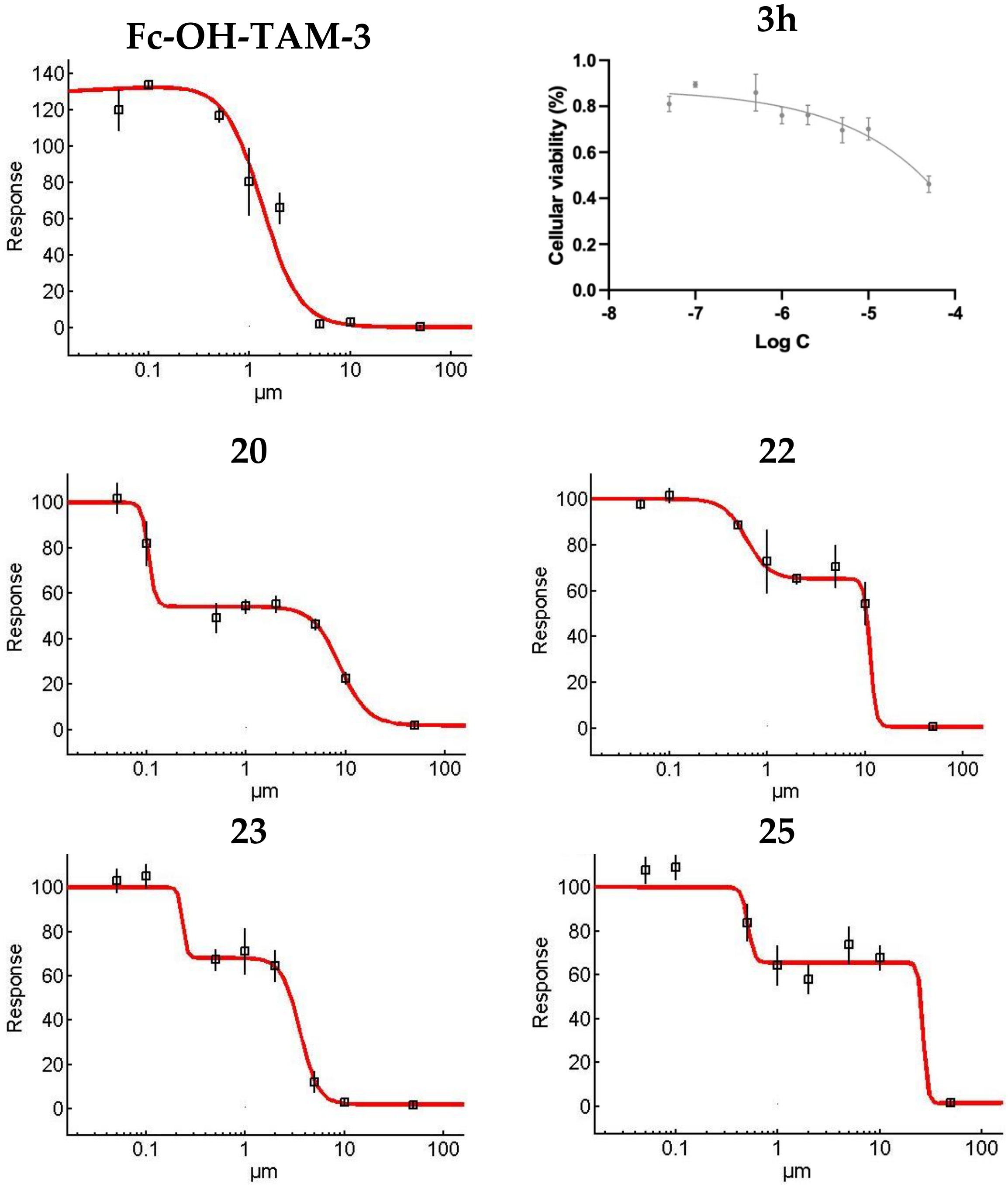

B: Fitting of dose-response curves for compounds **Fc-OH-TAM-3**, **3h**, **20**, **22**, **23**, **25** on MDA-MB-231 cells using Dr-Fit Software (Di Veroli et al. Sci. Rep. 2015, 5, 14701) or GraphPad Prism.

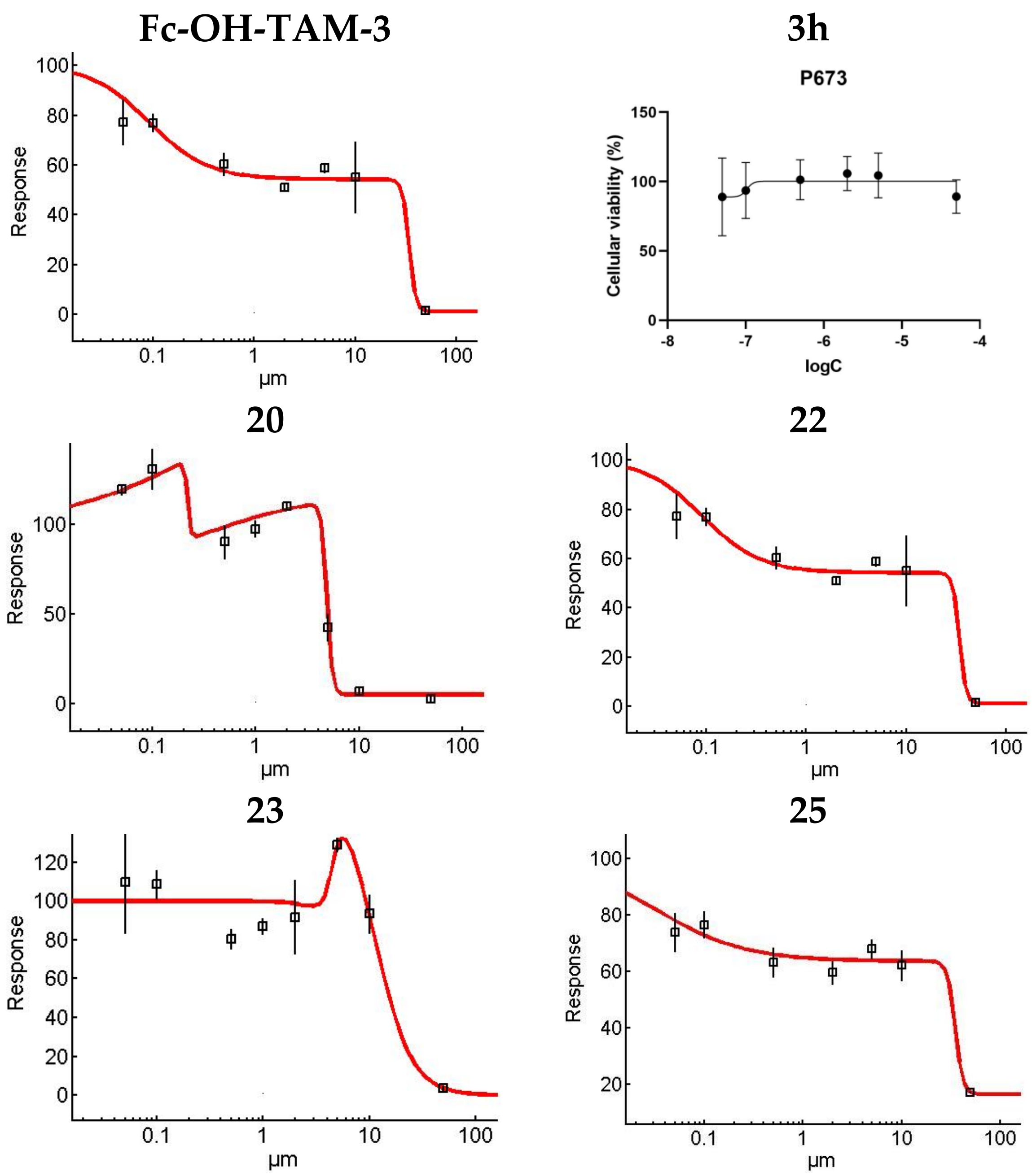

B: Fitting of dose-response curves for compounds **Fc-OH-TAM-3**, **3h**, **20**, **22**, **23**, **25** on hTERT-RPE1 cells using Dr-Fit Software (Di Veroli et al. Sci. Rep. 2015, 5, 14701) or GraphPad Prism.

Figure S53: HPLC traces for compounds **3h**, **20**, **22**, **23** and **25** (100 μM, acetonitrile, DMSO 1%; method loaded for all with acetonitrile/H_2O = 65/35, except **3h** with acetonitrile/H_2O = 75/25)

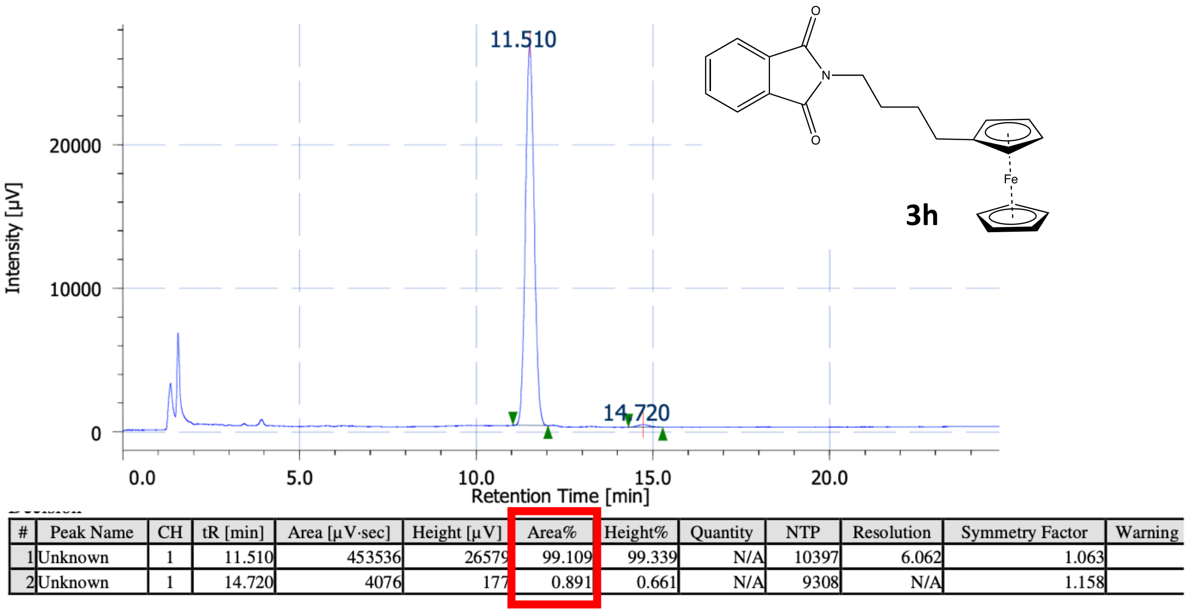

#	Peak Name	CH	tR [min]	Area [μV·sec]	Height [μV]	Area%	Height%	Quantity	NTP	Resolution	Symmetry Factor	Warning
1	Unknown	1	11.510	453536	26579	99.109	99.339	N/A	10397	6.062	1.063	
2	Unknown	1	14.720	4076	177	0.891	0.661	N/A	9308	N/A	1.158	

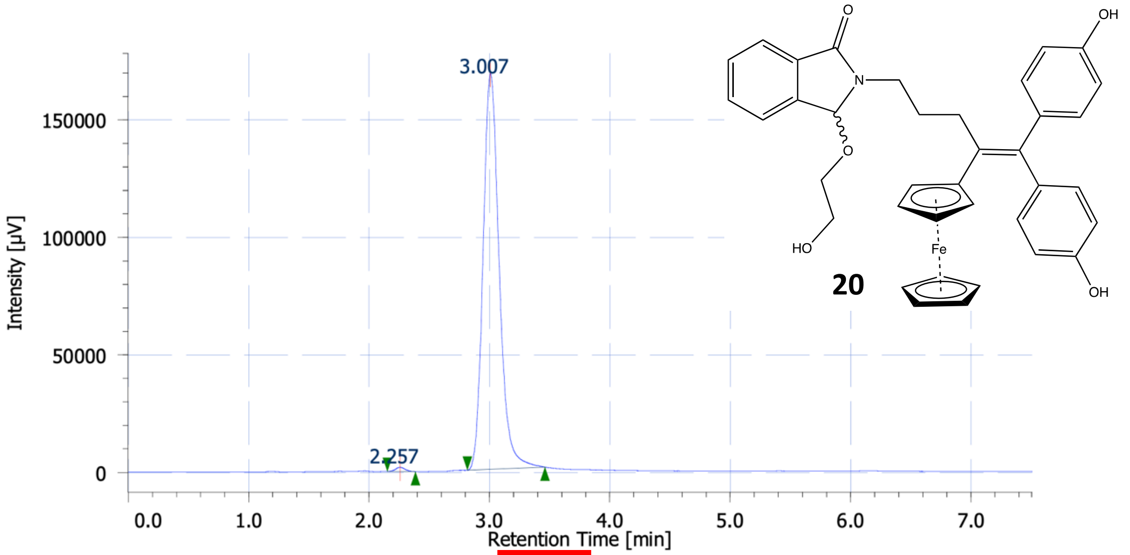

#	Peak Name	CH	tR [min]	Area [μV·sec]	Height [μV]	Area%	Height%	Quantity	NTP	Resolution	Symmetry Factor	Warning
1	Unknown	1	2.257	9980	1797	0.633	1.056	N/A	3683	3.896	1.148	
2	Unknown	1	3.007	1565580	168465	99.367	98.944	N/A	2570	N/A	1.215	

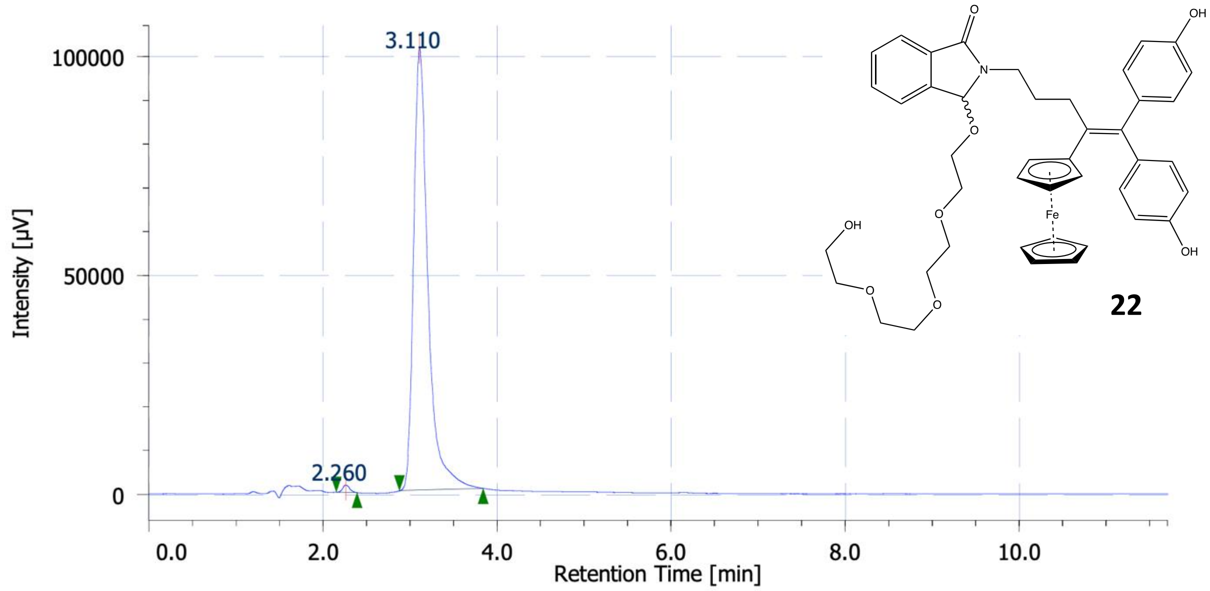

#	Peak Name	CH	tR [min]	Area [μV·sec]	Height [μV]	Area%	Height%	Quantity	NTP	Resolution	Symmetry Factor	Warning
1	Unknown	1	2.260	9469	1713	0.810	1.668	N/A	3750	4.004	1.192	
2	Unknown	1	3.110	1159459	100965	99.190	98.332	N/A	2002	N/A	1.539	

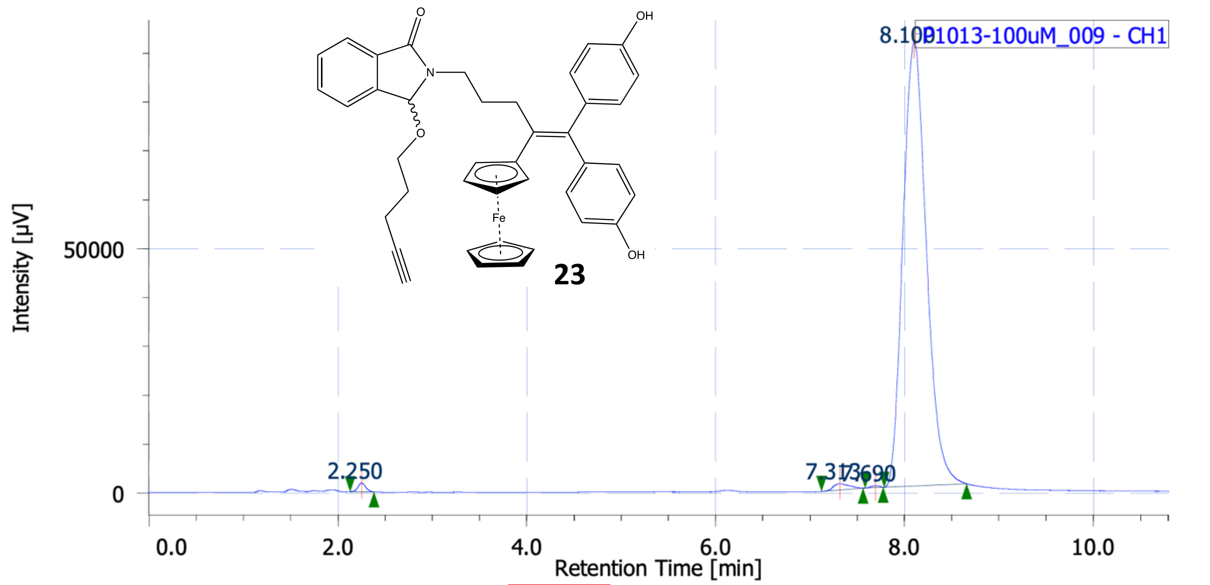

#	Peak Name	CH	tR [min]	Area [μV·sec]	Height [μV]	Area%	Height%	Quantity	NTP	Resolution	Symmetry Factor	Warning
1	Unknown	1	2.250	10286	1824	0.648	1.937	N/A	3652	21.801	1.126	
2	Unknown	1	7.313	15061	1291	0.948	1.370	N/A	8525	1.527	1.298	
3	Unknown	1	7.690	2309	373	0.145	0.396	N/A	29899	1.299	0.922	
4	Unknown	1	8.100	1560587	90692	98.259	96.297	N/A	5075	N/A	1.192	

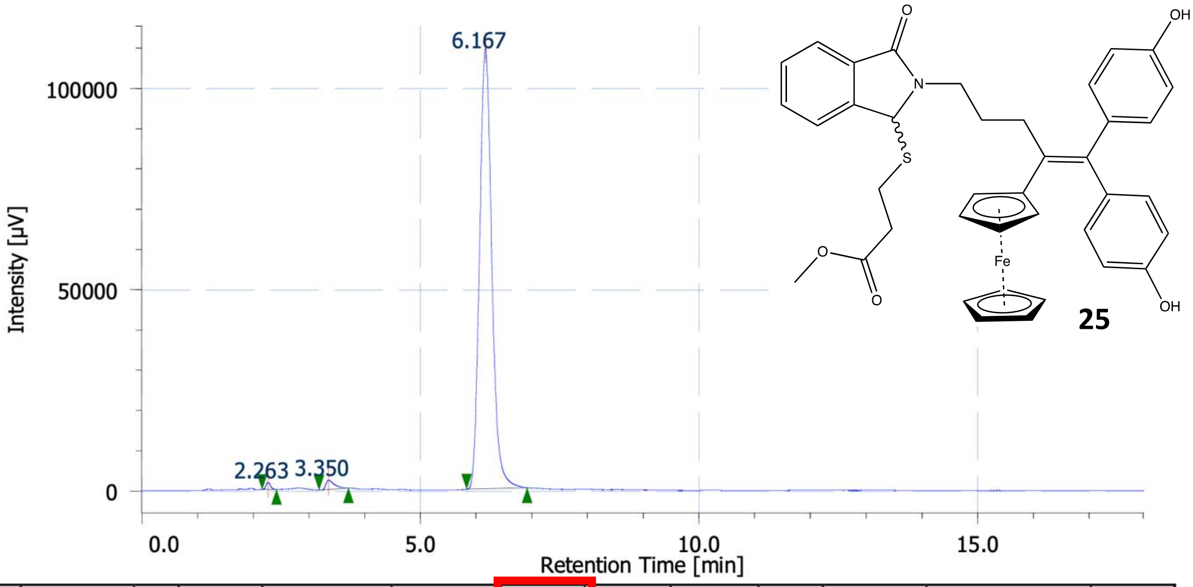

#	Peak Name	CH	tR [min]	Area [µV·sec]	Height [µV]	Area%	Height%	Quantity	NTP	Resolution	Symmetry Factor	Warning
1	Unknown	1	2.263	10131	180[illegible]	0.594	1.588	N/A	3633	5.208	1.143	
2	Unknown	1	3.350	24972	232[illegible]	1.465	2.046	N/A	2496	8.525	2.107	
3	Unknown	1	6.167	1669127	10942[illegible]	97.940	96.366	N/A	3912	N/A	1.148	
